# Effectiveness of Pharmacotherapy for Depression after Adult Traumatic Brain Injury: an Umbrella Review

**DOI:** 10.1007/s11065-022-09543-6

**Published:** 2022-06-14

**Authors:** Amelia J. Hicks, Fiona J. Clay, Amelia C. James, Malcolm Hopwood, Jennie L. Ponsford

**Affiliations:** 1 Monash-Epworth Rehabilitation Research Centre, Turner Institute for Brain and Mental Health, School of Psychological Sciences, Monash University, Ground Floor, 185-187 Hoddle St, Richmond, Melbourne, VIC 3121 Australia; 2 Department of Forensic Medicine, Monash University, Southbank, Australia; 3 Department of Psychiatry, University of Melbourne, Melbourne, Australia; 4 Professorial Psychiatry Unit, Albert Road Clinic, Department of Psychiatry, University of Melbourne, 31 Albert Road, Melbourne, Australia

**Keywords:** Traumatic brain injury, TBI, Depression, Pharmacotherapy, Umbrella review, Review

## Abstract

Symptoms of depression are common following traumatic brain injury (TBI), impacting survivors’ ability to return to work, participate in leisure activities, and placing strain on relationships. Depression symptoms post TBI are often managed with pharmacotherapy, however, there is little research evidence to guide clinical practice. There have been a number of recent systematic reviews examining pharmacotherapy for post TBI depression. The aim of this umbrella review was to synthesize systematic reviews and meta-analyses of the effectiveness of pharmacotherapy for the management of post TBI depression in adults. Eligible reviews examined any pharmacotherapy against any comparators, for the treatment of depression in adults who had sustained TBI. Seven databases were searched, with additional searching of online journals, Research Gate, Google Scholar and the TRIP Medical Database to identify published and unpublished systematic reviews and meta-analyses in English up to May 2020. A systematic review of primary studies available between March 2018 and May 2020 was also conducted. Evidence quality was assessed using Joanna Briggs Institute Critical Appraisal Instruments. The results are presented as a narrative synthesis. Twenty-two systematic reviews were identified, of which ten reviews contained a meta-analysis. No new primary studies were identified in the systematic review. There was insufficient high quality and methodologically rigorous evidence to recommend prescribing any specific drug or drug class for post TBI depression. The findings do show, however, that depression post TBI is responsive to pharmacotherapy in at least some individuals. Recommendations for primary studies, systematic reviews and advice for prescribers is provided. Review Registration PROSPERO (CRD42020184915).

**Supplementary Information:**

The online version contains supplementary material available at 10.1007/s11065-022-09543-6.

Following a traumatic brain injury (TBI), symptoms of depression are one of the most commonly reported mood changes (Gould et al., 2011a; Juengst et al., 2017; Mauri et al., 2014; Alway et al., 2016). Post-TBI depression can have a considerable impact on survivors, their families and the broader healthcare system. Post TBI depression has been associated with poorer functional outcomes (Haagsma et al., 2015; Lewis & Horn, 2017), lower employment rates, less engagement in leisure, recreation and community life, and difficulties with social relationships (Erler et al., 2019; Klyce et al., 2019), with these outcomes likely having a reciprocal and mutually exacerbating relationship with depression symptoms (Juengst et al., 2017; Haagsma et al., 2015). Post-TBI depression is associated with significant health care costs, with the estimated annual cost for military veterans with comorbid TBI and depression recently estimated at more than $1 billion USD (Dismuke-Greer et al., 2019).

Pooled prevalence rates suggest a 17% prevalence in the first year for depressive disorders, with long-term pooled prevalence estimates between 27%—43% depending on diagnostic method (Scholten et al., 2016; Osborn et al., 2014). The majority of depressive episodes occur in the first year after injury (Barker-Collo et al., 2015, 2018; Albrecht et al., 2019; Ouellet et al., 2018; Singh et al., 2019). From first occurrence, there are multiple possible trajectories of symptom evolution and resolution (Gould et al., 2011a; Barker-Collo et al., 2018; Ouellet et al., 2018; Hart et al., 2012; Bombardier et al., 2016), with some individuals experiencing gradual reduction of symptoms (Barker-Collo et al., 2015, 2018; Albrecht et al., 2019; Ouellet et al., 2018; Singh et al., 2019), while others experience little to no improvement, and even worsening of depressive symptoms over time (Alway et al., 2016; Bombardier et al., 2016; Senathi-Raja et al., 2010; Ouellet et al., 2018).

The aetiology of post-TBI depression is multi-faceted, including neurobiological mechanisms, pre-injury and comorbid personal factors, post-injury changes in functional ability, independence and participation, as well as psychological factors associated with adjustment after injury (Juengst et al., 2017). The most consistent predictor for post TBI depression is the presence of pre-injury depression or other psychiatric condition (Gould et al., 2011a; Bombardier et al., 2010; Scholten et al., 2016; Barker-Collo et al., 2015; Singh et al., 2018; Albrecht et al., 2019; Bombardier et al., 2016; Cnossen et al., 2017; Stein et al., 2019; Gould et al., 2011b). The association between TBI severity and risk of depression has been variable in studies to date; increasing TBI severity has been associated with increased (Osborn et al., 2014; Singh et al., 2018), decreased (Ouellet et al., 2018) or no association with risk of depression (Mauri et al., 2014; Singh et al., 2019; Senathi-Raja et al., 2010).

A number of neurobiological mechanisms have been implicated in post-TBI depression. Broadly, there is evidence associating post TBI mood disorders with disruption of neural circuits involved in emotional regulation (Moreno-López et al., 2016) including the prefrontal cortex, amygdala, hippocampus, insula, basal ganglia and thalamus (Jolly et al., 2019; Moreno-López et al., 2016; Jorge & Starkstein, 2005). Abnormalities in dopaminergic (Jolly et al., 2019) and glutaminergic neurotransmitter systems have been identified (Piao et al., 2019). Genetic factors may also influence a person’s vulnerability to post TBI depression (Jorge & Starkstein, 2005). Indeed, there is preliminary evidence of an association between depression post TBI and variations in a serotonin transporter gene (Failla et al., 2013), as well as the val66met polymorphism of the BDNF gene (Wang et al., 2018). Finally, there is emerging evidence for a possible role of a chronic hyperactive inflammatory system in development of depression (Fenn et al., 2014; Bodnar et al., 2018).

Depression symptoms post TBI are often managed with pharmacotherapy, however there is little methodologically rigorous research evidence to guide clinical practice and no gold standard treatment (Juengst et al., 2017). Clinical guidelines have been broadly consistent in suggesting selective serotonin reuptake inhibitors (SSRIs) as first-line treatment, with tricyclic anti-depressants (TCAs), stimulants, SNRIs (serotonin-norepinephrine reuptake inhibitor) and tetracyclic anti-depressants also suggested as options (Lamontagne et al., 2016; Marshall et al., 2012; Bayley et al., 2007; Group, 2006; Neurobehavioral Guidelines Working et al., 2006; Teasell et al., 2019; Plantier et al., 2016). Consistent with these recommendations, surveys of clinical practice reveal that SSRIs are the most frequently used medications for post TBI depression, with the most common drugs being citalopram, escitalopram and sertraline, with mirtazapine—a tetracyclic antidepressant—also commonly used (Albrecht et al., 2015).

There have been a number of recent systematic reviews examining pharmacotherapy for post TBI depression, with seven published between 2019 and 2020 alone (Beedham et al., 2020; Peppel et al., 2020; Gao et al., 2019; Liu et al., 2019; Kreitzer et al., 2019; Reyes et al., 2019; Slowinski et al., 2019). These reviews have offered little conclusive insight, with only a small subset endorsing pharmacological intervention over placebo. These reviews differ in their conduct, quality and reporting, and often have discordant results and conclusions. Given the multiple reviews on this topic, an umbrella review was deemed most appropriate (Pollock et al., 2018; Aromataris et al., 2020). This umbrella review will provide prescribers with a summary of this evidence, discussing methodological differences between reviews to highlight why conclusions have varied. This review will inform clinicians, pharmacists, allied health providers, drug regulators, policy makers, researchers and consumers as end-users on the safety and efficacy of pharmacological management of depression in individuals following a TBI.

A preliminary search (performed in May 2020) of PubMed, Cochrane Database of Systematic Reviews, CINAHL, Joanna Briggs Database of Systematic Reviews, PROSPERO, and EPISTEMONIKOS, found that there were no recent umbrella reviews or umbrella review protocols exploring our precise review objective and questions.

## Review Objective & Question

The objective of this review was to synthesize systematic reviews and meta-analyses of the effectiveness of pharmacotherapy, as compared with any other comparator, for the management of post TBI depression in adults 16 years and over. The specific review question was:

What is the current evidence for the effectiveness of pharmacotherapy for the management of depression in adults 16 years and older with mild to severe TBI?

## Methods

To ensure transparent, complete and accurate reporting, this review was conducted and reported in accordance with the Preferred Reporting Items for Systematic Review and Meta-Analysis (PRISMA) guidelines (Page et al., 2020a, b), the JBI Manual for Evidence Synthesis—Umbrella Reviews chapter (Aromataris et al., 2020), the Cochrane Handbook Overview of Reviews chapter (Pollock et al., 2018). The protocol for this review was published in JBI Evidence Synthesis (Hicks et al., 2021) and the review is registered on the PROSPERO database (CRD42020184915). There were five deviations from the protocol (Table 1).

**Table 1:** Five deviations from the Published Protocol and the Rationale for each Deviation

**Protocol**	**Deviation**	**Rationale**
**We will consider a paper a** **‘systematic review’** **if it includes** **(1) a PICO statement expressed as a study objective or a research question, (2) a comprehensive search strategy and (3) inclusion of studies against clear criteria**	We modified our criteria on what constituted a systematic review. Not all included systematic reviews fulfilled the specified criteria. (1) All studies had some form of study objective or review question. (2) A search strategy was not provided for one review (Liu et al., 2019). (3) Inclusion criteria were not clearly provided in five reviews (Liu et al., 2019; Neurobehavioral Guidelines Working et al., 2006; Plantier et al., 2016; Maksimowski & Tampi, 2016; Yue et al., 2017).	The purpose of our umbrella review is to provide a thorough summary of the reviews completed to date. We felt it was important to be flexible with our inclusion criteria to ensure we included as many of the reviews conducted on this topic to-date.
**Inclusion criteria – depression as primary outcome**	We did include reviews that examined studies for which depression was not the primary outcome (Plantier et al., 2016; Kreitzer et al., 2019; Reyes et al., 2019; Slowinski et al., 2019; Barker-Collo et al., 2013; Fann et al., 2009a). We did not extract data for these primary studies in to our review.	We include this here as a point of clarification as it was not specified in the protocol. This change does not deviate from the inclusion criteria we set out in the protocol.
**Inclusion criteria – adults 16 years and over**	It was not explicitly stated in all reviews that only primary studies of adult patients would be included For those reviews in which it was not explicitly stated in the eligibility criteria, information that the study comprised adult patients could be deciphered from the study characteristics tables or our knowledge of the included studies (Albrecht et al., 2015, 2019; Alway et al., 2016; Barker-Collo et al., 2015). Reviews that included patients less than 16 years of age were included if the findings for those aged 16 years or older were provided separately (Maksimowski & Tampi, 2016).	We include this here as a point of clarification as it was not specified in the protocol. This change does not deviate from the inclusion criteria we set out in the protocol.
**Systematic reviews of prophylactic (i.e. preventative) pharmacotherapy will be excluded**	We excluded all systematic reviews that focussed only on prophylactic pharmacotherapy. However, we did include reviews that included studies of both prophylactic pharmacotherapy and treatment pharmacotherapy. We did exclude the prophylactic studies from the reviews where possible. We retained the prophylactic studies if they were included in the only meta-analysis provided in a review. Removing the prophylactic studies in this context would have meant the review had no results.	The purpose of our umbrella review is to provide a thorough summary of the reviews completed to date. We did not wish to exclude reviews that had included prophylactic studies in their meta-analyses, as this would have resulted in excluding three reviews that we would have otherwise included. Prophylactic studies still address the question of efficacy for pharmacotherapy for depression so we felt this was valid.
**GRADE will be applied to assess the certainty of the evidence**	The GRADE approach will not be used.	Based on heterogeneity in interventions, samples, methodology and outcomes, GRADE was deemed not to be appropriate for this umbrella review.

*GRADE* Grading of Recommendations, Assessment, Development and Evaluations, *PICO* Participants, Intervention, Comparator, Outcomes

### Inclusion Criteria

Systematic reviews were selected for inclusion according to the criteria outlined below.

### Participants

Eligible reviews included studies of participants who were adults (16 years and over) of both sexes who had sustained a TBI (penetrating or non-penetrating; medically confirmed or self-report) of any cause and severity. There were no restrictions on age at injury or time since injury. Reviews of both TBI and non-TBI participants (i.e., other acquired brain injury such as stroke), were eligible if the findings from the TBI samples were presented separately or if greater than 80% of the sample was TBI. Given the age of adulthood is defined differently internationally, the minimum age of 16 was chosen. Reviews with studies in which 80% of the sample were 16 years and older were also eligible.

Participants had to present with depression of any severity as diagnosed through a standardized diagnostic interview procedure (e.g., Diagnostic and Statistical Manual of Mental Disorders criteria (DSM)) or valid rating scale. There are multiple depression rating scales that have been validated in the TBI population, including the Hospital Anxiety & Depression Scale (HADS) (Dahm et al., 2013; Schwarzbold et al., 2014), the Hamilton Rating Scale for Depression (HAM-D) (Schwarzbold et al., 2014), the Beck Depression Inventory (BDI) (Schwarzbold et al., 2014), Patient Health Questionnaire-9 (PHQ-9) (Cohen et al., 2018; Donders & Pendery, 2017), Traumatic Brain Injury Quality of Life subscale (TBI-QoL—Depression) (Cohen et al., 2018) and the Depression Anxiety Stress Scales -21 (DASS-21) (Dahm et al., 2013; Randall et al., 2017). Depression symptoms could be reported by the individual with TBI, by their clinician or other informant (e.g., family member, carer).

### Interventions

Only systematic reviews of pharmacotherapeutic interventions were considered for inclusion. The primary focus of the intervention had to be to treat depression. All pharmacotherapy interventions were eligible for inclusion, and there was no restriction on dosage, frequency, duration or follow-up. Mixed interventions (e.g., pharmacotherapy and psychological therapy) were eligible for inclusion if the data for the pharmacological intervention were reported separately. Systematic reviews of only prophylactic (i.e., preventative) pharmacotherapy were excluded. We did, however, include systematic reviews of depression treatment that also included a small number of prophylactic studies (see further explanation in Table 1).

### Comparators

Included reviews compared pharmacological interventions with all types of comparators. There were no restrictions on the type of comparator; placebo, active control (e.g., drugs within the same pharmacological class or another class), supportive, standard care or a non-pharmacological intervention were all accepted.

### Outcomes

The primary outcomes of interest were change in symptoms of depression and occurrence of harms. No secondary outcomes were included in the review. All results in the systematic reviews that were compatible with each of the primary outcomes were extracted.

### Context

All settings were eligible for inclusion; e.g., acute care, inpatient rehabilitation, outpatient rehabilitation, community.

### Studies

We included any systematic reviews (with or without meta-analyses) of the effectiveness of pharmacotherapy for post TBI depression in human adults available in English. Our criteria for a ‘systematic review’ was (1) a PICO statement expressed as a study objective or a research question, (2) a search strategy, and (3) inclusion of studies against clear criteria (but see Table 1 for deviations from this definition). Systematic reviews including both RCT and non-RCT (e.g., cohort studies, case–control studies) were included. We also considered meta-analyses that were not part of a systematic review. The following study types were excluded: systematic reviews of qualitative studies or case reports, economic evaluations, narrative reviews and primary research. Reviews focusing more broadly on psychopathology or neurobehavioral symptoms following TBI were included if the outcomes for depression were presented separately. Likewise, reviews examining pharmacotherapy for depression across many different medical conditions were included if the outcomes for the TBI sample were presented separately.

### Search Strategy

An information specialist with extensive experience in conducting systematic reviews developed and ran the search strategy. The search strategy was designed to identify both published and unpublished systematic reviews and meta-analyses, based on elements of the PICO (Population = brain injury and depression, Intervention = pharmacotherapy) and the study type. Appendix 1 provides the full search strategy.

Key words were identified by examining the titles, abstracts and search strategies of relevant published systematic reviews sourced from the Cochrane Library and Pubmed. The keywords were then added to the search strategy along with a range of Medical Subject Headings (MeSH) terms linked by Boolean operators.

The MEDLINE search strategy was peer-reviewed by the information specialist using the Peer-Review of Search Strategies checklist (PRESS) (McGowan et al., 2016) before translating the strategy for other databases and running the final searches. No date restrictions were applied.

### Information Sources

MEDLINE (Ovid SP; 1946 – May 2020) and EMBASE (Excerpta Medica Database; Ovid SP; 1974 – May 2020) were searched as they index most systematic reviews (Hartling et al., 2016). Two discipline-specific databases were also searched; PsycINFO (Ovid SP; 1967 – April 2020) and CINAHL (Cumulative Index to Nursing and Allied Health Literature; EBSCO Host; 1937 – May 2020), along with Epistemonikos, the Cochrane Database of Systematic Reviews (Cochrane Library) and PROSPERO (no date restrictions; all searched May 2020). Where a protocol was found with no accompanying published systematic review, the authors were contacted twice over a 6 week period to confirm the publication status of the systematic review. All published protocols without an accompanying systematic review are listed in Appendix 2. In addition to the database search, in May – June 2020 we also searched reference lists for included systematic reviews, online search of key journals (Neuropsychology Review; 1990 – June 2020, Brain Impairment; 2000 – June 2020; Journal of Neurotrauma; 1988 – June 2020), and searched ResearchGate, Google Scholar and the TRIP Medical Database (no date restrictions; searched June 2020). This umbrella review was last assessed as up to date in June 2020.

### Study Selection

All study screening, data extraction and methodological assessment was completed independently by two reviewers (AH, FC & AJ). Disagreements were resolved through consensus, and if required a third team member adjudicated (AH, FC & AJ).

All identified citations were uploaded into Endnote and duplicates removed. Titles and abstracts were screened against the inclusion criteria. Reviews that potentially met the inclusion criteria were retrieved in full and assessed against the inclusion criteria. Full text reviews that did not meet the inclusion criteria were excluded.

Given the purpose of this umbrella review was to present and describe the current body of systematic review evidence where overlapping reviews were identified—that is, systematic reviews containing the same primary studies—we have included both reviews. Throughout the review selection process and assessment of methodological quality, reviewers were not blinded to the journal titles, study authors or their institutions.

### Assessment of Methodological Quality

The JBI critical appraisal tool for research synthesis (Aromataris et al., 2020) was used to assess methodological quality. The tool assesses bias across nine areas (1—explicit review question; 2 – appropriate inclusion criteria; 3 – appropriate search strategy; 4—adequate search; 5 – appropriate critical appraisal; 6 – independent critical appraisal by multiple authors; 7 – minimization of data extraction errors; 8 – appropriate combination of studies; 9 – assessment of publication bias), with two final items related to review quality (10 – recommendations for policy/practise supported by reported data; 11 – appropriate directives for future research). Each item is assessed as ‘Yes’, ‘No’ or ‘Unclear’. One modification was made to this tool, adding a category of ‘Yes*’ to denote when a review fulfilled the criteria for an item, however, there were small caveats that may have introduced some minor bias. We derived an overall risk of bias judgement (low; intermediate; high) through examining performance across the 11 items, and detailed discussion to arrive at consensus, to allow for interpretation of review conclusions to be made with respect to overall study quality. No reviews were excluded based on methodological quality.

### Data Extraction

Data extraction was conducted using the standardized JBI data extraction tool (Aromataris et al., 2020). The tool was customized and piloted, with all modifications to the tool being developed and agreed upon by the review team (Aromataris et al., 2020) (data extraction form; Appendix 3). This was an iterative process with multiple versions of the tool being developed and refined. Only findings relevant to our two primary outcomes (changes in depression; occurrence of harms) were extracted. It is accepted practice to restrict attention to a subset of the evidence included in the systematic reviews (Pollock et al., 2018). Authors of systematic reviews were contacted (n = 1) to clarify missing or unclear information in their review. After data extraction, another member of the team checked all table entries for accuracy, completeness, and consistency. Extracted data is presented in a series of tables and narrative synthesis, with no quantitative re-synthesis of results (Aromataris et al., 2020).

### Systematic Review

In order to provide a complete and up-to-date reflection of the current available evidence, we also conducted a systematic review of primary studies examining effectiveness of pharmacotherapy for depression following TBI published in the last two years (March 2018 to May 2020). Although inclusion of additional primary studies within an umbrella review is as at variance with standard methodological expectations of this review format, it is an accepted practice when the existing systematic reviews are out of date (Pollock et al., 2018). The methodology for the systematic review is outlined in Appendix 4, and the full search strategy is available in Appendix 1.

## Results

### Systematic Review

A systematic review of primary studies available between March 2018 and May 2020 was conducted alongside the umbrella review.

#### Literature Search

The literature search produced 711 articles, 576 from bibliographic databases and 135 from additional sources. Title and abstract screening was completed for 625 articles after 86 duplicates were removed. Of the five articles reviewed at full-text, none were deemed eligible for inclusion in the systematic review. The PRISMA flow diagram for the systematic review is provided in Appendix 5, with further elaboration provided in Appendix 2.

As there were no primary studies deemed eligible for inclusion, we did not undertake the planned systematic review and it will not be discussed further in the results section.

### Umbrella Review

#### Literature Search

The literature search produced 499 articles, 454 from bibliographic databases and 45 from additional sources. Title and abstract screening was completed on 360 articles after 139 duplicates were removed. There were 310 articles excluded during the title and abstract screening stage.

**Fig. 1:**
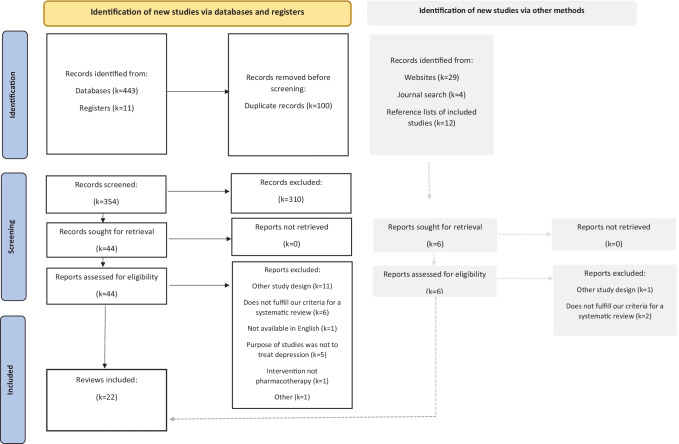
PRISMA Flow Diagram for the Systematic Review Detailing the Results of the Literature Search, Article Screening and Study Selection Process

Of the 50 articles reviewed at full-text, 28 were excluded. There were 22 systematic reviews deemed eligible for inclusion (see Appendix 6 for list of citations). Figure 1 outlines the screening process and reasons for exclusion, with further elaboration provided in Appendix 2.

#### Description of Included Reviews

Twenty-two systematic reviews published between 2004 and 2020 were included in the umbrella review. The most recent search within the systematic reviews was February 2019 (Gao et al., 2019). Nine systematic reviews provided only a narrative synthesis (Fann et al., 2009a; Guillamondegui et al., 2011; Plantier et al., 2016; Comper et al., 2005; Maksimowski & Tampi, 2016; Liu et al., 2019; Neurobehavioral Guidelines Working et al., 2006; Hardy, 2009; Deb & Crownshaw, 2004), with ten including a meta-analyses (Gao et al., 2019; Peppel et al., 2020; Kreitzer et al., 2019; Paraschakis & Katsanos, 2017; Reyes et al., 2019; Barker-Collo et al., 2013; Slowinski et al., 2019; Salter et al., 2016; Beedham et al., 2020; Wheaton et al., 2011). A further three reviews also included meta-analyses, but these combined both pharmacological and non-pharmacological interventions (Barker-Collo et al., 2013), or included other clinical populations (Rayner et al., 2010; Price et al., 2011), and as such were not extracted for this umbrella review. Five reviews were restricted to RCTs only (Peppel et al., 2020; Gao et al., 2019; Reyes et al., 2019; Paraschakis & Katsanos, 2017; Price et al., 2011), with all other reviews including other study designs such as cohort and case–control designs.

Random effects models were used for the majority of meta-analyses. One review used either a random or fixed effects model depending on the level of heterogeneity (Beedham et al., 2020), and one review did not state the model used (Wheaton et al., 2011). Heterogeneity statistics were provided for the majority of meta-analyses, with only three failing to do so (Yue et al., 2017; Salter et al., 2016; Wheaton et al., 2011). Sensitivity analyses examining the effect of individual trials on the significance of the results were performed in a limited number of reviews (n = 4/13) (Beedham et al., 2020; Kreitzer et al., 2019; Price et al., 2011; Rayner et al., 2010).

#### Primary Studies Included in Reviews

Twenty-one primary studies published between 1985 and 2017 were included across the systematic reviews (Table 2). We only extracted from primary studies that met our inclusion criteria, resulting in a total of between 1 to 15 primary studies per review (Mean = 6.41). The overlap between reviews, that is the extent to which primary studies in the reviews were the same, was determined by calculating the corrected covered area (CCA) (Pieper et al., 2014). The CCA was determined to be 0.27, corresponding to a “slight” overlap (Pieper et al., 2014). The overall sample size of TBI participants and healthy controls included in reviews was 30 to 650 participants (Mean = 214.80; Table 3).

**Table 2:**
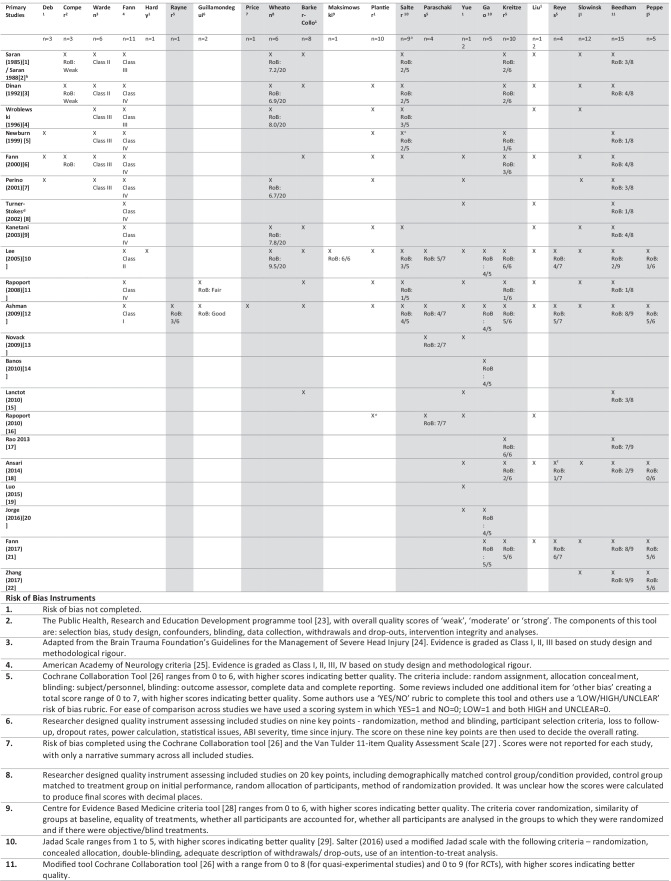
Citation Matrix Showing the Primary Studies within the 22 Included Systematic Reviews and Risk of Bias Assessment for each Primary Study

Columns in grey shading represent reviews with meta-analyses. Those columns with no shading are systematic reviews that provided a narrative synthesis only

The superscript numbers in the ‘systematic reviews’ row correspond to the risk of bias instrument used, which are listed with their corresponding number at the end of the table

Year of publication for the systematic reviews is not included due to space issues within the table. The systematic reviews have been ordered chronologically from left to right from oldest to most recently published

This table only includes primary studies from the systematic reviews that fulfilled our eligibility criteria

We only included classifications of evidence class if the classification system also included consideration of methodological rigour. Classifications of evidence class made only using study design (e.g. RCT as Class I), were not reported in this table as they do not include an assessment of methodological rigour (e.g. Plantier et al., 2016)

*RCT* randomised controlled trial, *RoB* risk of bias

^a^Salter et al. (2016) only completed methodological assessment for studies that included a comparison group

^b^Three independent authors (AH, FC & AJ) reviewed Saran (1985) and Saran (1988) and agreed these reports contain the same primary study

^c^Salter et al. (2016) did not include Newburn et al. (1999) in their meta-analysis due to insufficient data reported

^d^The findings from Turner-Stokes et al. (2002) are not included for Yue et al. (2017) and Liu et al. (2019), as these reviews did not report the findings for the TBI sample separately

^e^Plantier et al. (2016) refers to this study as ‘Rapoport (1999)’. However, all extracted details in their manuscript and the citation in their reference list is for Rapoport (2010)

^f^Reyes et al. (2019) refers to this study as ‘Ansari (2017)’. However, all extracted details in their manuscript and the citation in their reference list is for Ansari et al. (2014)

**Table 3:** Study Characteristics for the 22 Systematic Reviews Included in the Umbrella Review

**Citation**	**Key eligibility criteria for primary studies**^**a**^	**Primary Studies** **# Studies**^**b,c**^ **Study designs** **Sample Size**^**d**^	**Sample** **Sex** **Age** **% TBI**^**e**^	**Depression Entry Criteria**	**Interventions** **(number of studies)**	**Comparators**^**f**^	**Heterogeneity**^**g**^
Beedham et al. (2020) **Up to Date: January 2019** **Protocol: PROSPERO CRD42019122600** **Meta-Analysis**	Study design: RCT or quasi-experimental; depression as outcome – severity scale or remission rates Age: adults ≥ 18yrs TBI: diagnosis by clinician/ healthcare professional Depression: diagnosis using standardised criteria/ score on validated tools Intervention: TMS, pharmacotherapy or psychological	**# Studies:** 15 **Study Designs:** - 6 × RCT - 2 × non-RCT w control group - 7 × non-RCT w/out control group **Sample Size:** 504	**Sex:** NR **Age:** NR **% TBI:** 100%	**DSM MDD** III; n = 1 IV; n = 1 IV-SCID (n = 2) IV-MINI (n = 1) **HAM-D** ≥ 15 & IV-SCID (n = 1) ≥ 17 (n = 1) ≥ 18 & SCID (n = 1) ≥ 18 & DIS Interview (n = 1) **PHQ-9** ≥ 10; (n = 1) **BDI** ≥ 18 (n = 2) **Clinical interview** (n = 2)	**MAOI** Moclobemide (n = 1) **SNRI** Milnacipran (n = 1) **SSRI** Citalopram (n = 2) Sertraline (n = 6) Escitalopram (n = 1) **Stimulant** Methylphenidate (n = 2) **TCA** Amitriptyline (n = 2) **Combination Therapy** SSRI + Anti-convulsant (n = 1; Citalopram Carbamazepine)	- Comparator condition: TBI group; placebo given (n = 5) - Comparator condition: TBI group; no placebo given (n = 1) - Comparator condition: non-TBI controls; study drug given (n = 2) - No comparator condition (n = 7)	**Depression** I^2^ = 0—84%
Peppel et al. (2020) **Up to Date: September 2018** **Protocol: NR** **Meta-Analysis**	- Study design: RCT; primary outcome was depression; baseline and follow-up depression scores for intervention and control - Study aim: having depression was inclusion criterion/reducing depression was aim of study - Age: ≥ 16yrs at injury/ reported separately from other age groups - TBI: mod to sev TBI/ reported separately from other ABI - Intervention: any treatment modality - Comparator: any comparison group - Excluded: studies of mild TBI only; comorbid PTSD; preventative treatment; n < 10 total or n < 5 per group	**# Studies:** 5 **Study Designs:** - 5 × RCT **Sample Size:** 249	**Sex:** 202/249 male; 81% **Age:** **M** 32.4–49.1 **% TBI:** 100%	**NR**	**SSRI** Sertraline (n = 4) **Stimulant** Methylphenidate (n = 2)	- Comparator condition: TBI group; placebo given (n = 4) - Comparator condition: TBI group; no placebo given (n = 1)	**Depression** I^2^ = 43.26%
Gao et al. (2019) **Up to Date: February 2019** **Protocol: NR** **Meta-Analysis**	- Study design: RCT - TBI: any severity - Intervention: Sertraline - Comparator: placebo	**# Studies:** 5 **Study Designs:** - 5 × RCT **Sample Size:** 316	**Sex:** 213/316 male; 67% **Age:** **M** 33.6–54.91 **% TBI:** 100%	**NR** (n = 4) **N/A (preventative study)** (n = 1)	**SSRI** Sertraline (n = 5)	- Comparator condition: TBI group; placebo given (n = 5)	**Depression** I^2^ = 4% **Harms** I^2^ = 0–64%
Kreitzer et al. (2019) **Up to Date: September 2017** **Protocol: NR** **Meta-Analysis**	-Study design: prospective studies - TBI: any severity - Depression: MDD - Intervention: any Anti-depressant - Excluded: anti-depressant intervention for MDD refractory to other first-line agents; same cohort from prior study	**# Studies:** 10 **Study Designs:** - 5 × RCT - 5 × not specified **Sample Size:** 336	**Sex:** NR **Age:** NR **% TBI:** 100%	**NR**	**MAOI** Moclobemide (n = 1) Phenelzine (n = 1) **SSRI** Escitalopram (n-1) Citalopram (n = 1) Sertraline (n = 5) **Stimulant** Methylphenidate (n = 1) **TCA** Amitriptyline (n = 2)	- Comparator condition: TBI group; placebo given (n = 4) - Comparator condition: TBI group; no placebo given (n = 1) - Comparator condition: non-TBI controls; study drug given (n = 2) - No comparator condition (n = 3)	**Depression** I^2^ = 17%
Liu et al. (2019) **Up to Date: NR** **Protocol: NR** **Narrative Synthesis**	**NR**	**# Studies:** 12 **Study Designs:** - 3 × RCT - 6 X non-RCT w control group - 3 X non-RCT w/out control group **Sample Size:** 317^ h^	**Sex:** NR **Age:** NR **% TBI:** Unclear^i^	**NR**	**SNRI** Milnacipran (n = 1) **SSRI** Citalopram (n = 2) Sertraline (n = 6) **Stimulant** Methylphenidate (n = 1) **TCA** Amitriptyline (n = 2) Desipramine (n = 1)	- Comparator condition: TBI group; placebo given (n = 5) - Comparator condition: TBI group; no placebo given (n = 1) - Comparator condition: non-TBI controls; study drug given (n = 2) - No comparator condition (n = 4)	N/A – no meta- analysis
Reyes et al. (2019) **Up to Date: NR** **Protocol: NR** **Meta-Analysis**	- Study design: RCT; depression as a primary or secondary outcome - Age: ≥ 18yrs - TBI: documented LoC/radiological evidence - Depression: diagnosed w MDD using standardized diagnostic criteria - Intervention: Sertraline - Excluded: patients with diagnosis of bipolar disorder, psychotic disorder, substance abuse disorder, bereavement, suicidal ideations or intent; taking anti-depressants; undergoing psychotherapy; serious medical illness; history of allergy/adverse reaction to study drug; pregnant or breastfeeding; prophylactic studies	**# Studies:** 4 **Study Designs:** - 4 × RCT **Sample Size:** 224^j^	**Sex:** 181/224 male; 80.1% **Age**^k^**:** **M** 33.6 – 49.1 **% TBI:** 100%	**DSM-IV &** HAM-D ≥ 18 (n = 1) HAM-D ≥ 15 (n = 1) BDI > 18 (n = 1) **PHQ-9** (n = 1)	**SSRI** Sertraline (n = 4)	- Comparator condition: TBI group; placebo given (n = 3) - Comparator condition: TBI group; no placebo given (n = 1)	**Depression** I^2^ = 84 – 98%
Slowinski et al. (2019) **Up to Date: NR** **Protocol: NR** **Meta-Analysis**	Study design: depression as a primary or secondary outcome Age: > 18yrs TBI: any severity Depression: any; must have depression diagnosis at baseline Intervention: any Setting: any	**# Studies:** 12 **Study Designs:** - 2 × Non-RCT w control group - 3 × Non-RCT w/out control group - 7 × Clinical trial w control group (unclear if RCT) **Sample Size:** Unclear^l^	**Sex:** NR **Age:** NR **% TBI:** 100%	**DSM (no further info provided)** (n = 11) **PHQ-9** (n = 1)	**MAOI** Phenelzine (n = 1) **SNRI** Milnacipran (n = 1) **SSRI** Citalopram (n = 1) Sertraline (n = 5) **Stimulant** Methylphenidate (n = 2) **TCA** Amitriptyline (n = 2) Desipramine (n = 1) **Combination Therapy** SSRI + Anti-convulsant (n = 1; Citalopram Carbamazepine)	- Comparator condition: TBI group; placebo given (n = 6) - Comparator condition: TBI group; no placebo given (n = 1) - Comparator condition: non-TBI controls; study drug given (n = 2) - No comparator condition (n = 3)	**Depression** I^2^ = 81.45–91.94%
Paraschakis and Katsanos (2017) **Up to Date: August 2017** **Protocol: NR** **Meta-Analysis**	- Study design: RCT; HAM-D as outcome measure - Age: adult - TBI: any - Depression: major/mod depression, adjustment disorder w depressive symptoms, dysthymic disorder - Intervention: anti-depressants - Comparator: placebo	**# Studies:** 4 **Study Designs:** - 4 × RCT **Sample Size:** 181	**Sex:** 123/181 male; 68% **Age:** **M** 34.5 – 47.7 **% TBI:** 100%	**DSM-IV** n = 1 & HAM-D ≥ 18 (n = 1) & HAM-D ≥ 16 (n = 1) **N/A (preventative study)** (n = 1)	**SSRI** Citalopram (n = 1) Sertraline (n = 3)	- Comparator condition: TBI group; placebo given (n = 4)	**Depression** I^2^ = 0%
Yue et al. (2017) **Up to Date:** **September 2016** **Protocol: NR** **Meta-Analysis & Narrative Synthesis**^**m**^	**NR**	**# Studies:** 12 **Study Designs:** - 6 × RCT - 6 × Non-RCT w/out control group **Sample Size:** 650	**Sex:** 80/650 male; 570/650 NR **Age:** NR **% TBI:** 96.6%^n^	**DSM-IV MDD/ MDE** (n = 4) **BDI (cut-off ns)** (n = 1) **MDD (diagnosis method ns)** (n = 1) **NR** (n = 6) **N/A (preventative study)** (n = 2)	**SSRI** Sertraline (n = 7) Citalopram (n = 5)	- Comparator condition: TBI group; placebo given (n = 5) - Comparator condition: TBI group; no placebo given (n = 1) - No comparator condition (n = 6)	**NR**
Maksimowksi and Tampi (2016) **Up to Date: December 2015** **Protocol: NR** **Narrative Synthesis**	**NR**	**# Studies:** 1 **Study Designs:** - 1 × RCT **Sample Size:** 30	**Sex:** NR **Age:** M 34.8 **% TBI:** 100%	**NR**	**Stimulant** Methylphenidate (n = 1)^o^	- Comparator condition: TBI group; placebo given (n = 1)	N/A – no meta- analysis
Plantier et al. (2016) **Up to Date: June 2015** **Protocol: NR** **Narrative Synthesis**	Study Aim: to treat behavioural disorders post-TBI Exclusion: participants in acute recovery phase in ICU; interventions to improve cognition/ stimulate recovery; absent/insufficient participants w TBI	**# Studies:** 10 **Study Designs:** - 4 × RCT - 1 × Non-RCT w control group - 5 × Non-RCT w/out control group **Sample Size:** 308	**Sex:** 4/295 male; 291/295 NR **Age:** n = 1; R 28–74 n = 9; NR **% TBI:** 100%	**NR**	**MAOI** Moclobemide (n = 1) **SNRI** Milnacipran (n = 1) **SSRI** Citalopram (n = 2) Sertraline (n = 3) **Stimulant** Methylphenidate (n = 1) **TCA** Amitriptyline (n = 1) Desipramine (n = 1) **Combination Therapy** SSRI + Anti-convulsant (n = 1; Citalopram Carbamazepine)	- Comparator condition: TBI group; placebo given (n = 4) - Comparator condition: non-TBI controls; study drug given (n = 1) - No comparator condition (n = 5)	N/A – no meta- analysis
Salter et al. (2016) **Up to Date: October 2014** **Protocol: NR** **Meta-Analysis**	Study design: clinical trial Age: adults TBI: any severity; presence of TBI made w comparison to NINDS Common Data Elements definition Depression: diagnosis using standardised criteria/ score on validated tools; must have depression diagnosis at baseline Intervention: any pharmacotherapy	**# Studies:** 9 **Study Designs:** - 3 × RCT - 2 × Non-RCT w control group - 4 × Non-RCT w/out control group **Sample Size:** 245	**Sex:** NR **Age: M** 28.5 – 58.3 **% TBI:** 100%	**DSM** III-R (n = 1) III-R MDD (n = 1) III MDD & HAM-D > 17 (n = 1) III-R MDD & HAM-D ≥ 18 (n = 1) IV MDE (n = 1) IV MDE & HAM-D > 18 (n = 1) IV MDE/MDD (n = 1) **BDI** ≥ 18 (n = 1) **NR** (n = 1)	**MAOI** Moclobemide (n = 1) Phenelzine (n = 1) **SNRI** Milnacipran (n = 1) **SSRI** Citalopram (n = 1) Sertraline (n = 3) **Stimulant** Methylphenidate (n = 1) **TCA** Amitriptyline (n = 2) Desipramine (n = 1)	- Comparator condition: TBI group; placebo given (n = 3) - Comparator condition: non-TBI controls; study drug given (n = 2) - No comparator condition (n = 4)	**NR**
Barker-Collo et al. (2013) **Up to Date: NR** **Protocol: NR** **Meta-Analysis**^**p**^	Study Design: depression/depressive symptoms as an outcome measure; pre- and post-test assessment Age: adult TBI: mTBI (LoC ≤ 30 min/PTA < 24 h) Intervention: pharmacological or non-pharmacological	**# Studies:** 8 **Study Designs:** - 2 × RCT - 2 × Non-RCT w control group - 4 × Non-RCT w/out control group **Sample Size:** 195	**Sex:** 181/224 male; 80.1% **Age**^q^**:** **M** 33.6 – 49.1 **% TBI:** 100%	**MDD & HAM-D ≥ 18** n = 1 **DSM** III MDD- Feighner (1972) criteria (n = 1) III MDD & HAM-D > 17 (n = 1) III-R MDD & HAM-D > 17 (n = 1) IV MDE (n = 1) IV MDE or ‘minor depression’ (n = 1) IV MDD (n = 2) IV Diagnosis ns (n = 1)	**SNRI** Milnacipran (n = 1) **SSRI** Sertraline (n = 3) Citalopram (n = 2) **Stimulant** Methylphenidate (n = 1) **TCA** Amitriptyline (n = 2)	- Comparator condition: TBI group; placebo given (n = 2) - Comparator condition: non-TBI controls; study drug given (n = 2) - No comparator condition (n = 4)	**Depression** I^2^ = 71.1–86.7%^r^
Guillamondegui et al. (2011) **Up to Date: May 2010** **Protocol: NR** **Narrative Synthesis**	- Study design: RCT, cohort w comparison, case control, case series; n ≥ 50 - Age: ≥ 16yrs - TBI: any severity; sustained as adult - Depression: any severity; diagnostic information required - Setting: study conducted in a ‘developed nation’ - Excluded: penetrating TBI; self-report depression	**# Studies:** 2 **Study Designs:** - 1 × RCT - 1 × Non-RCT w/out control group **Sample Size:** 132	**Sex:** NR **Age:** **M** 39.7 – 51.5 **% TBI:** 100%	**DSM** **IV MDE & HAM-D ≥ 18** (n = 1) **IV MDD (5/9 symptoms and depressed mood or anhedonia)** (n = 1)	**SSRI** Citalopram (n = 1) Sertraline (n = 1)	- Comparator condition: TBI group; placebo given (n = 1) - No comparator condition (n = 1)	N/A – no meta- analysis
Price et al. (2011) **Up to Date:** **August 2009** **Protocol: NR** **Meta-Analysis**	Study design: RCT; depression primary outcome Age: adult TBI: any neurological disease w biological underpinning Depression: MDD, adjustment disorder, dysthymic disorder; diagnosis using standardised criteria/score on validated tools Intervention: antidepressant Comparator: placebo Excluded: dementia; MCI	**# Studies:** 1 **Study Designs:** - 1 × RCT **Sample Size:** 41	**Sex:** NR **Age:** NR **% TBI:** 100%	**NR**	**SSRI** Sertraline (n = 1)	- Comparator condition: TBI group; placebo given (n = 1)	**Depression** I^2^ = 78%^s^
Wheaton et al. (2011) **Up to Date: April 2010** **Protocol: NR** **Meta-Analysis**	Study Design: treatment and control group; outcomes measures of cognition/behaviour; sufficient data provided to calculate ES Age: ≥ 16yrs/M-1SD ≥ 16yrs TBI: non-penetrating Intervention: any pharmacology; administered ≥ 4wks post TBI Excluded: previous TBI, pre-existing neurological/psychiatric disorder, substance abuse history	**# Studies:** 6 **Study Designs:** - 2 × RCT - 2 x non-RCT w control group - 4 x non-RCT w/out control group **Sample Size:** Unclear^t^	**Sex:** NR **Age:** M 29.10 – 42 **% TBI:** 100%	**NR**	**MAOI** Phenelzine (n = 1) **SNRI** Milnacipran (n = 1) **SSRI** Citalopram (n = 1) Sertraline (n = 1) **Stimulant** Methylphenidate (n = 1) **TCA** Amitriptyline (n = 2) Desipramine (n = 1)	- Comparator condition: TBI group; placebo given (n = 2) - Comparator condition: non-TBI controls; study drug given (n = 2) - No comparator condition (n = 2)	**NR**
Rayner et al. (2010) **Up to Date: December 2009** **Protocol: Yes** **Meta-Analysis**	Study Design: RCT (cluster and cross-over were eligible); depression as primary outcome Age: > 18yrs Depression: MDD, adjustment disorder, dysthymic disorder using standardised criteria/ score on validated tools; any severity Intervention: anti-depressant prescribed for depression Comparator: placebo	**# Studies:** 1 **Study Designs:** - 1 × RCT **Sample Size:** 52	**Sex:** 22/52 male; 42% **Age: M** 46.8 – 51.5 **% TBI:** 100%	**NR**	**SSRI** Sertraline (n = 1)	- Comparator condition: TBI group; placebo given (n = 1)	**Depression** I^2^ = 54.07–78.27%^u^
Fann et al. (2009a) **Up to Date: NR** **Protocol: NR** **Narrative Synthesis**	- Study design: depression as a primary or secondary outcome; quantitative pre/post scores provided - TBI: any severity; reported separately from other ABI - Intervention: any treatment modality	**# Studies:** 11 **Study Designs:** - 2 × RCT - 3 × Non-RCT w control group - 6 × Non-RCT w/out control group **Sample Size:** 243	**Sex:** 13/243 male; 230/243 NR **Age:** NR **% TBI:** 100%	**DSM MDD** III (n = 2) III-R (n = 3) IV (n = 5) **HAM-D** > 17 (n = 3) ≥ 17 (n = 1) ≥ 18 (n = 1)	**MAOI** Moclobemide (n = 1) Phenelzine (n = 1) **SNRI** Milnacipran (n = 1) **SSRI** Citalopram (n = 1) Sertraline (n = 4) **Stimulant** Methylphenidate (n = 1) **TCA** Amitriptyline (n = 2) Desipramine (n = 1) **Combination Therapy** SSRI + Anti-convulsant (n = 1; Citalopram Carbamazepine)	- Comparator condition: TBI group; placebo given (n = 3) - Comparator condition: non-TBI controls; study drug given (n = 2) - No comparator condition (n = 6)	N/A – no meta- analysis
Hardy (2009) **Up to Date:** **July 2008** **Protocol: NR** **Narrative Synthesis**	Study design: controlled trials, case series, case reports Age: older adults TBI: any Depression: any	**# Studies:** 1 **Study Designs:** - 1 × RCT **Sample Size:** 30	**Sex:** NR **Age:** M 34yrs **% TBI:** 100%	**NR**	**Stimulant** Methylphenidate (n = 1)	- Comparator condition: TBI group; placebo given n = 1	N/A – no meta- analysis
Warden et al. (2006) **Up to Date:** **October 2004** **Protocol: NR** **Narrative Synthesis**	**NR**	**# Studies:** 6 **Study Designs:** - 1 × RCT - 2 X non-RCT w control group - 3 X non-RCT w/out control group **Sample Size:** 119	**Sex:** NR **Age:** NR **% TBI:** 100%	**DSM** III (n = 1) III-R (n = 2) IV (n = 1) **NR** (n = 2)	**SSRI** Sertraline (n = 1) **TCA** Amitriptyline (n = 2) Desipramine (n = 1) **MAOI** Moclobemide (n = 1) **Combination Therapy** SSRI + Anti-convulsant (n = 1; Citalopram Carbamazepine)	- Comparator condition: TBI group; placebo given (n = 1) - Comparator condition: non-TBI controls; study drug given (n = 2) - No comparator condition (n = 3)	N/A – no meta- analysis
Comper et al. (2005) **Up to Date: 2003** **Protocol: NR** **Narrative Synthesis**	Age: 16-65yrs TBI: mTBI; mixed severity with mTBI separately reported; ≤ 5yrs post injury Intervention: any in human TBI populations Excluded: case series and case studies	**# Studies:** 3 **Study Designs:** - 2 × Non-RCT w control group - 1 × Non-RCT w/out control group **Sample Size:** 63	**Sex:** 47/63 male; 74.6% **Age: M** 30.7 – 44.2 **% TBI:** 100%	**NR**	**MAOI** Phenelzine (n = 1) **SSRI** Sertraline (n = 1) **TCA** Amitriptyline (n = 2)	- Comparator condition: non-TBI controls; study drug given (n = 2) - No comparator condition (n = 1)	N/A – no meta- analysis
Deb and Crownshaw (2004) **Up to Date: January 2003** **Protocol: NR** **Narrative Synthesis**	Age: majority of sample > 16yrs TBI: any Depression: any Intervention: any drug that may affect behaviour directly or indirectly Excluded: studies using non-psychotropic drugs acute post injury	**# Studies:** 3 **Study Designs:** - 1 × non-RCT w control group - 1 × non-RCT w/out control group - 1 × Unclear **Sample Size:** 61	**Sex:** NR **Age:** M 27 – 41.9^v^ (NR; n = 1) **% TBI:** 100%	**DSM** III-R MDD (n = 1) **NR** (n = 2)	**SSRI** Sertraline (n = 1) **MAOI** Moclobemide (n = 1) **Combination Therapy** SSRI + Anti-convulsant (n = 1; Citalopram Carbamazepine)	- No comparator condition (n = 3)	N/A – no meta- analysis

Non-RCT w/out control group includes open trials, cohort studies, case series

*ABI* Acquired Brain Injury, *BDI* Beck Depression Inventory, *DSM* Diagnostic and Statistical Manual of Mental Disorders (III – 3^rd^ Edition; III-R – 3^rd^ Edition, Revised; IV – 4^th^ Edition; IV-SCID – Structured Clinical Interview for DSM-IV; IV-MINI – Mini-International Neuropsychiatric Interview), *ES* Effect Size, *HAM-D* Hamilton Rating Scale for Depression, *ICU* Intensive Care Unit, *LoC* Loss of Consciousness, *M* Mean, *MAOI* Monoamine Oxidase Inhibitors, *MCI* Mild Cognitive Impairment, *MDD* Major Depressive Disorder, *MDE* Major Depressive Episode, *mTBI* Mild Traumatic Brain Injury, *n* Number, *NINDS* National Institute of Neurological Disorders and Stroke, *PHQ-9* Patient Health Questionnaire (9 Questions), *SNRI* Serotonin and Norepinephrine Reuptake Inhibitors, *NR* Not Reported, *N/A* Not Applicable, *PTA* Post-Traumatic Amnesia, *PTSD* Post-Traumatic Stress Disorder, *R* Range, *RCT* Randomized Controlled Trial, *SD* Standard Deviation, *SSRI* Selective Serotonin Reuptake Inhibitor, *TBI* Traumatic Brain Injury, *TCA* Tricyclic Antidepressant, *TMS* Transcranial Magnetic Stimulation

^a^Eligibility criteria had to be clearly provided in the methods section of the review

^b^The number of studies does not always equate to the number of interventions as some studies used multiple intervention groups

^c^Only primary studies that met the umbrella review’s inclusion criteria were extracted from each systematic review

^d^This refers to the overall sample size; including both the treatment group and control group

^e^% of participants included in the extracted data that have TBI (as opposed to other non-TBI ABI)

^f^The category of ‘no comparator condition’ is used for single arm study such as cohort study

^g^We have only extracted the I^2^ value as this was consistently reported across reviews. Some reviews reported other heterogeneity statistics that are not included in the table

^h^Liu et al. (2019) only included the number of cases in the primary studies (i.e. did not report the number of healthy control participants)

^i^Liu et al. (2019) included the entire mixed ABI sample from Turner-Stokes et al. (2002). Data from the TBI group was not reported separately

^j^We have extracted the sample size as stated in the abstract of this paper and Table 1. However, it is noted that the overall sample size should be 223. There were three studies with loss to follow-up. For two studies (Ashman et al., 2009; Fann et al., 2017), all participants enrolled in the study have been included in Table 1 and counted in the overall sample size. In contrast, for the remaining study (Ansari et al., 2014) with loss to follow-up, only those who completed the study have been included. As such, if the 9 people lost to follow-up are included in the overall sample size, this brings the sample size to 233. However, the Lee et al. (2005) study’s sample size should only be 20 participants as only 20 participants received sertraline or placebo interventions, with the remaining 10 participants receiving methylphenidate and therefore not being included in this review. With removal of these 10 participants, this bring the sample size back down to 223

^k^Ansari et al. (2014) was not included in calculating participant average age, as review extracted frequency of age brackets with no M or SD provided

^l^Slowinski et al. (2019) only included the number of cases in the primary studies (i.e. did not report the number of healthy control participants)

^m^Yue et al. (2017) included 12 studies. Two studies were included in a meta-analysis and the remaining 10 studies were only included in a narrative synthesis

^n^Yue et al. (2017) included the entire mixed ABI sample from Turner-Stokes et al. (2002). As Turner-Stokes et al. (2002) was not included in their meta-analysis, this did not impact the meta-analysis findings

^o^Maksimowski and Tampi (2016) also made reference to the SSRI sertraline as the one included study (Lee et al., 2005) had two treatment arms; methylphenidate and sertraline. However, given the focus of their review was on stimulants, and the sertraline intervention group was considered another comparator group by Maksimowksi and Tampi (2016), we have only included methylphenidate in our extraction

^p^Barker-Collo et al. (2013) pooled pharmacological and non-pharmacological interventions in their meta-analyses, with no separate meta-analyses conducted for pharmacological interventions only. As such, no pooled estimates could be extracted from this review

^q^Ansari et al. (2014) not included in calculating participant average age, as review extracted frequency of age brackets with no M or SD provided

^s^The meta-analysis in Price et al. (2011) included other neurological disorders. This heterogeneity figure is therefore not specific to the TBI study only

^t^Wheaton et al. (2011) only included the sample size for the treatment group in Supplemental Table B. Given there were two RCTs with a control group it is not clear what the overall sample size is

^u^The meta-analyses in Rayner et al. (2010) included other physical illnesses. These heterogeneity figures are therefore not specific to the TBI study only

^v^The average age reported here from the Deb and Crownshaw (2004) review is based on only two of the three studies. No details about age were provided for the third study

The reviews provided varying amounts of descriptive details about the primary studies with respect to comparators, participants and outcome measures (Table 3). Studies that included a group comparison usually included a placebo condition as the comparator. However, there were a small number of studies that used a control condition without a placebo or used non-TBI controls (i.e., ‘healthy controls’ with depression) as the comparator condition. Participant characteristics (i.e., gender and age) were often not reported or only partially reported (n = 14/22). Where sex was reported, the samples consistently included more males than females. Half of the reviews provided information about the depression entry criteria required in the primary studies (n = 11/22). There was much variation both in the measures used and the cut-off values within measures. The HAM-D or HAM-D 21 were the most common measurement tools used within the primary studies. Other popular tools included the BPRS, CGI, BDI/ BDI-II and PHQ-9. The majority of reviews included primary studies with samples of any TBI severity (14/22). One review restricted included studies to those with moderate to severe TBI only, and two reviews only included mild TBI. Five reviews did not provide this information.

### Search Strategy

The majority of reviews (n = 20/22) provided detailed information on their search strategy (Appendix 7) and the date upon which their search was last assessed as up-to-date (n = 16/22). The reviews searched between one and seven databases (M = 4.04), with most reviews restricting their search to English language publications (n = 15/22). Although almost all reviews provided details of supplementary searches (n = 19/22) (e.g., clinical trial registries, hand searching journals), only a small number included a search for unpublished literature (n = 8/22).

### Interventions

Six drug classes (MAOIs, TCAs, SSRIs, SNRIs, stimulants and anti-convulsants) and 10 individual drugs were examined across the 22 reviews (Table 3). The majority of reviews did not specify the follow up time point (n = 15/22), and only one study in the reviews examined outcomes post drug cessation, with outcomes assessed 7 days and again at 21 days post intervention (Newburn et al., 1999).

### Measurement of Harms and Drop Outs

Harms were not mentioned in half of the reviews (n = 11/22) (Hardy, 2009; Deb and Crownshaw, 2004; Barker-Collo et al., 2013; Peppel et al., 2020; Comper et al., 2005; Wheaton et al., 2011; Rayner et al., 2010; Beedham et al., 2020; Kreitzer et al., 2019; Paraschakis & Katsanos, 2017; Slowinski et al., 2019). Only one review conducted a meta-analysis for harms data (Gao et al., 2019), and one review conducted a tolerability analysis (Price et al., 2011). Six reviews reported on study drop-outs where possible (i.e., where this was reported in the primary studies) (Guillamondegui et al., 2011; Salter et al., 2016; Price et al., 2011; Neurobehavioral Guidelines Working et al., 2006; Plantier et al., 2016; Reyes et al., 2019). Of these, three commented on the reasons for drop outs for at least some studies – noting where this was due to adverse events (Guillamondegui et al., 2011; Neurobehavioral Guidelines Working et al., 2006; Plantier et al., 2016).

### Findings

The following section summarises the meta-analyses’ findings across reviews for citalopram, sertraline, methylphenidate and amitriptyline, as well as for the drug classes SSRIs and TCAs (Table 4 provides detailed information about the study designs, samples, intervention and findings including effect sizes). The colours used in Table 4 refers to the methodological assessment: green—‘low’ risk of bias, yellow—‘intermediate’ risk of bias, red—‘high’ risk of bias. The findings from six primary studies examining drugs not included in any meta-analyses (or only included in meta-analyses that pooled across drug classes) are then briefly discussed (Appendix 8).

In interpreting the meta-analysis summaries below it is important to understand the distinction between meta-analyses using either a ‘pre-post’ or ‘control comparison’ design. ‘Pre-post’ data is from single group studies that have compared change in score pre-intervention to post-intervention (i.e., without a control comparison group). ‘Control comparison’ data is from studies using two independent groups—a treatment and control group.

### SSRIs – Sertraline, Citalopram & Escitalopram

#### Depression

Three meta-analyses pooled findings across studies examining either sertraline, citalopram or escitalopram. Only the pre-post meta-analysis found in favour of SSRIs, and reported a large effect size for the difference in depression scores from pre to post intervention in samples of mild to severe TBI. The two control comparison meta-analyses failed to find a significant effect in mild to moderate TBI (Beedham et al., 2020) and mild to severe TBI (Paraschakis & Katsanos, 2017). Although one of the control comparison meta-analysis reviews did have an intermediate risk of bias, the other review was assessed as low risk of bias.

The efficacy of sertraline on depression scores was examined in twelve meta-analyses reported within seven reviews (Beedham et al., 2020; Peppel et al., 2020; Gao et al., 2019; Reyes et al., 2019; Slowinski et al., 2019; Yue et al., 2017; Paraschakis & Katsanos, 2017). Broadly, three of the meta-analyses found a significant impact of sertraline on depression (Beedham et al., 2020; Slowinski et al., 2019; Yue et al., 2017), with moderate to large effect sizes. The remaining eight meta-analyses failed to find a significant effect of sertraline on depression (Gao et al., 2019; Reyes et al., 2019; Paraschakis & Katsanos, 2017). Peppel et al. (2020) found conflicting results using the same four studies in control comparison meta-analyses. The only point of difference was the measures used, with meta-analyses in favour of sertraline including data from the HAM-D and PHQ-9, and the meta-analyses not finding in favour of sertraline using HAM-D, PHQ-9, BDI-II and SCL-20.

Both pre-post meta-analyses (n = 2/2) found in favour of sertraline. In comparison, the majority of control-comparison meta-analyses (n = 8/10) did not find in favour of sertraline. These meta-analyses were from reviews with a low risk of bias (n = 6/8) or intermediate risk of bias (n = 2/8). Of the two control-comparison meta-analyses that were in favour of sertraline, one had a high risk of bias, with the other review assigned a low risk of bias. Many of the reviews included participants across the spectrum of severity and time since injury, with no pattern identified between these factors and response to sertraline.

Two citalopram studies including mild to moderate TBI were combined in a pre-post meta-analysis from a review with low risk of bias showing a significant improvement in depression scores with a large effect size (Beedham et al., 2020).

#### Harms

For sertraline, a control-comparison meta-analysis from a review of moderate to severe TBI with a low risk of bias showed that the risk of harms was not greater in the treatment group for diarrhoea, dizziness, dry mouth and nausea/vomiting. Further information provided in narrative summaries confirmed this, summarising from primary studies that although the treatment group did report greater intestinal gas, agitation, decreased libido, gastrointestinal palpitations and sweating, this was not significantly greater than that reported by the control group (Reyes et al., 2019; Fann et al., 2009a). No meta-analytic or quantitative data were provided for harms relating to citalopram. Further information provided in narrative summaries stated that common side effects of citalopram included decreased libido, dry mouth, nausea, sedation and diarrhoea (Fann et al., 2009a; Plantier et al., 2016).

#### Concluding Statements

When pooled across individual drugs, control-comparison meta-analyses did not find favourable results for SSRIs. The single meta-analysis with positive findings for SSRIs in mild to severe TBI was of a high quality, however, used a pre-post design. The weight of the higher quality evidence from control comparison meta-analyses fails to show sertraline as effective for depression following mild to severe TBI based on the evidence collected to date. There were promising results from one high quality meta-analysis showing no greater risk of harms in individuals with moderate to severe TBI given sertraline compared to placebo. One review with low risk of bias provided support for citalopram following mild to moderate TBI. However, this was from a pre-post analysis with no control group. These meta-analyses included participants across the spectrum of severity and time since injury. Overall, discordant conclusions between analyses could not be clearly accounted for by any differences in injury severity. Further, given most meta-analyses included the full spectrum from mild to severe TBI or included only a subset of severity with no comparison between severity groups, no insights could be gained regarding the impact of TBI severity on drug effectiveness.

### Stimulants – Methylphenidate

#### Depression

Four meta-analyses in two reviews with low risk of bias provided mostly favourable data with large effect sizes for the use of methylphenidate for post TBI depression (Beedham et al., 2020; Peppel et al., 2020). The majority of participants across all analyses had sustained a mild to moderate injury and were early post injury; however, these details were not consistently provided. Both pre-post and control comparison analyses using HAM-D showed methylphenidate to result in significantly reduced depression scores. Notably, the single meta-analysis not in favour of methylphenidate differed only in the measures used – using BDI for depression scores as opposed to HAM-D.

#### Harms

No data on harms from stimulant use were provided.

#### Concluding Statements

The meta-analyses from two high quality reviews provide promising evidence for methylphenidate. However, it is of concern that the findings were not robust to the measures used to assess depression. Further, the total pool of participants used in the meta-analyses was quite small (n = 28–56). These findings are limited to survivors of mild to moderate injuries in the early phase post injury.

### TCAs – Amitriptyline & Desipramine

#### Depression

A pre-post meta-analysis of three studies from a review with low risk of bias examining amitriptyline and desipramine treatment found a significant improvement in depression scores following mild to moderate TBI with a large effect size (Salter et al., 2016). Two pre-post meta-analyses of amitriptyline also found a significant impact of the drug on depression scores, with both reporting large effect sizes in samples of mild and ‘minor’ TBI (Beedham et al., 2020; Wheaton et al., 2011).

#### Harms

No meta-analytic or quantitative data were provided for harms relating to amitriptyline. Information was provided in narrative summaries for desipramine, noting the occurrence of seizures and manic episodes (Fann et al., 2009a; Plantier et al., 2016; Neurobehavioral Guidelines Working et al., 2006).

#### Concluding Statements

Although all meta-analyses for TCAs produced positive findings, these were for pre-post studies only and in small samples (n = 23 – 58). Further, one of these reviews was judged to have a high risk of bias. The lack of any harms data for amitriptyline and occurrence of harms for those taking desipramine reinforces hesitation in considering this drug. Any conclusions from these reviews could only be generalized to those who have sustained mild to moderate injuries and are early post injury.

### Pharmacotherapy – SSRIs & Stimulants

#### Depression

Three reviews, all with low risk of bias, provided control-comparison meta-analyses pooling across SSRIs and stimulants (Peppel et al., 2020; Kreitzer et al., 2019; Salter et al., 2016). Two of these meta-analyses concluded in favour of pharmacotherapy and reported moderate to large effect sizes, with the third meta-analysis failing to find such evidence. All meta-analyses included participants across the spectrum of severity and time since injury, and used a similar combination of measures to assess depression (HAM-D, PHQ-9, and MADRS). Meta-regression showed no significant difference between sertraline and methylphenidate (Peppel et al., 2020).

#### Harms

No data on harms was provided.

#### Concluding Statements

It is difficult to draw conclusions from studies that have grouped across drug classes, as it is unclear whether one or both of the drug classes is associated with the positive effect. Further, as these drug classes were not provided as combination therapy in any of the primary studies, conclusions cannot be drawn about using these drug classes as co-interventions. We recommend referring to the conclusions above about each of these drug classes independently.

### Pharmacotherapy – Multiple Drug Classes

#### Depression

Three reviews with intermediate to low risk of bias provided five meta-analyses pooling across multiple drug classes to examine the effects of pharmacotherapy more broadly on post TBI depression (Beedham et al., 2020; Slowinski et al., 2019; Salter et al., 2016). The single control-comparison meta-analysis from a review with intermediate risk of bias was the only analysis to conclude not in favour of pharmacotherapy. The TBI severity in that meta-analysis was not reported. The four meta-analyses finding in favour of pharmacotherapy reported moderate to large effect sizes, were all pre-post analyses and were drawn from reviews with intermediate (n = 2) and low (n = 2) risk of bias. TBI severity was only provided for two of these meta-analyses, for which one was mild to moderate and the one was mild to severe.

#### Harms

No data on harms was provided.

#### Concluding Statements

There is some evidence from reasonably high quality reviews that pharmacotherapy may be effective for post TBI depression. However, all these meta-analyses were pre-post designs with no control comparison group. Indeed, the single control-comparison meta-analysis, drawn from an intermediate quality review, did not find in favour of pharmacotherapy. As stated above, it is difficult to draw any conclusions from reviews that pool across drug classes—beyond that post-TBI depression appears to be responsive to pharmacotherapeutic intervention. This does not, however, provide any specific guidance for clinicians. We recommend referring to the conclusions above about each of these drug classes independently.

### Other drugs

There were six drugs that were either included in meta-analyses that pooled across drug classes or were not included in any meta-analyses. We provide brief details from the primary studies examining these drugs below (Appendix 8). Given this evidence is drawn from single primary studies, it should be given considerably less weight then the meta-analyses findings summarised above.

*Phenelzine (MAOI)* treatment was not associated with a significant change in HAM-D scores over 4 weeks in 22 survivors of mild TBI (time since injury unclear) (Saran, 1985). No harms data provided.

*Desipramine (TCA)* treatment was associated with a significant reduction in scores compared to placebo on a researcher generated affect/mood scale in a total of 10 survivors (6 TG; 4 CG) of TBI sustained an average of 1.5 years previously (injury severity was moderate or less) (Wroblewski et al., 1996). Two participants withdrew due to seizures and mania, with two further participants reporting action tremors and mild seizures but remaining in the trial.

*Moclobemide (MAOI)* treatment was associated with a mean reduction in HAM-D scores of 80.79% in 26 survivors of TBI (injury severity and time since injury not reported) (Newburn et al., 1999). Twenty four adverse events were reported by 14 subjects, with five drop-outs due to adverse events.

A combination of *Citalopram (SSRI)* and *Carbamazepine (Anti-Convulsant)* was associated with a significant reduction in BPRS scores over 12 weeks in 20 survivors of a moderate to severe TBI an average of 4.6 to 34.6 months post injury (Perino et al., 2001). No harms data was provided.

*Milnacipran (SNRI)* treatment was associated with a significant improvement in HAM-D scores over 6 weeks, with a 66.7% response rate and 44.4% remission rate in 10 survivors of TBI sustained an average of 152.8 days prior (injury severity was unclear). One participant withdrew due to nausea.

*Escitalopram (SSRI)* treatment was associated with a reduction in MADRS scores over 12 weeks in 14 TBI survivors (injury severity and time since injury were not reported) (Rao, 2013). No harms were reported by participants receiving the treatment.

### Quality and Risk of Bias

#### Financial Support & Conflicts of Interest

Twelve reviews reported receiving financial support for the conduct of their review (Fann et al., 2009a; Peppel et al., 2020; Guillamondegui et al., 2011; Plantier et al., 2016; Comper et al., 2005; Rayner et al., 2010; Price et al., 2011; Beedham et al., 2020; Wheaton et al., 2011; Liu et al., 2019; Neurobehavioral Guidelines Working et al., 2006; Hardy, 2009), however, no support was received from any pharmaceutical companies. The majority of reviews (n = 14/22) declared no conflicts of interest (Fann et al., 2009a; Gao et al., 2019; Guillamondegui et al., 2011; Paraschakis & Katsanos, 2017; Reyes et al., 2019; Barker-Collo et al., 2013; Plantier et al., 2016; Rayner et al., 2010; Salter et al., 2016; Yue et al., 2017; Beedham et al., 2020; Wheaton et al., 2011; Maksimowski & Tampi, 2016; Liu et al., 2019). Five reviews did not report on whether there were conflicts of interest (Comper et al., 2005; Slowinski et al., 2019; Neurobehavioral Guidelines Working et al., 2006; Hardy, 2009; Deb & Crownshaw, 2004).

#### Protocols

Only one study had pre-published a protocol for their systematic review (Beedham et al., 2020). However, the authors did not address whether there were any deviations from the protocol. Comparison between the protocol and review (completed by two study authors independently and in duplicate; AH & AJ), identified only one change: searching of clinical trials registries was completed in the review but not stipulated in the protocol.

#### Methodological Assessment of Primary Studies

Seven systematic reviews did not complete a methodological assessment of their included primary studies (Deb & Crownshaw, 2004; Hardy, 2009; Barker-Collo et al., 2013; Plantier et al., 2016; Yue et al., 2017; Liu et al., 2019; Slowinski et al., 2019) (Table 2). Ten different risk of bias tools were used across the other studies (description of tools and adjudications provided in Table 2). For most studies, the risk of bias assigned across reviews was reasonably consistent (e.g., Dinan and Mobayed (1992), Newburn et al. (1999), Fann et al. (2017)). However, for the other studies, there was considerable variation in the risk of bias assigned (for example see entries for Lee et al. (2005), Ashman et al. (2009); Table 2). No studies conducted a quality appraisal to assess the confidence in their findings (e.g., GRADE).

#### Methodological Assessment of the Systematic Reviews

The JBI critical appraisal tool for research synthesis (Aromataris et al., 2020) was used to appraise the risk of bias and methodological rigour in each review (Table 5). Expanded rationale for all methodological assessments are provided in Appendix 9.

**Table 4:**
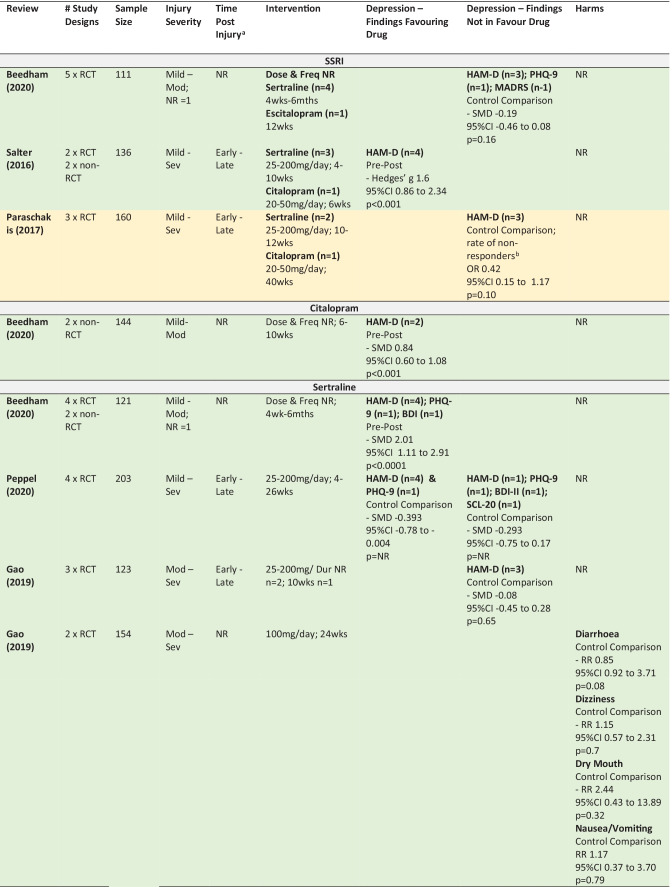

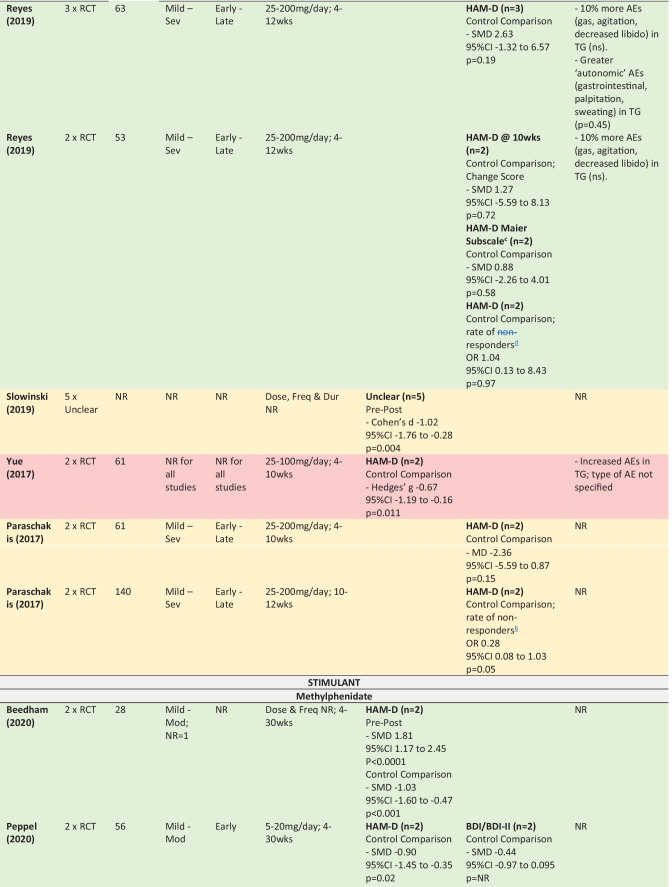

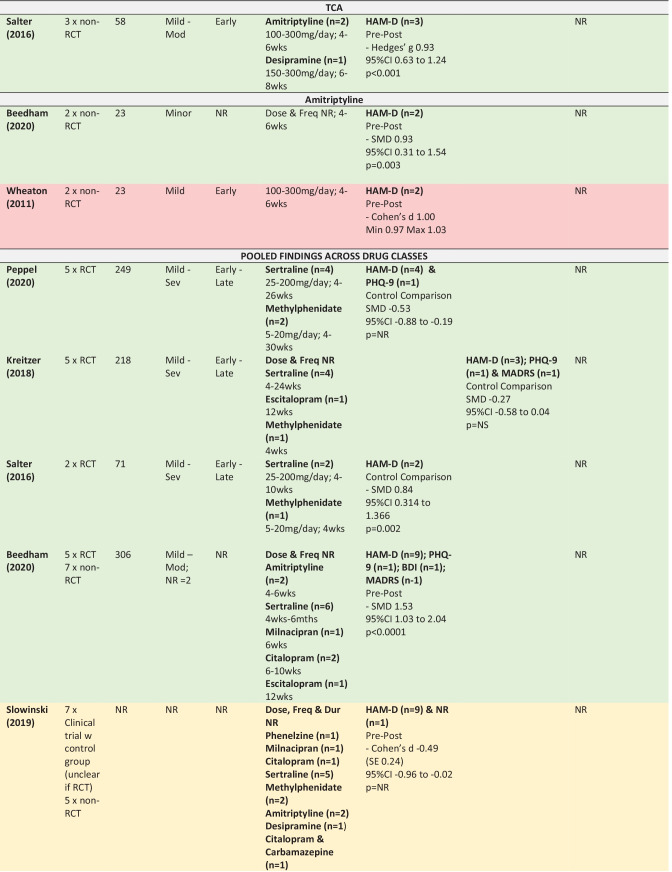

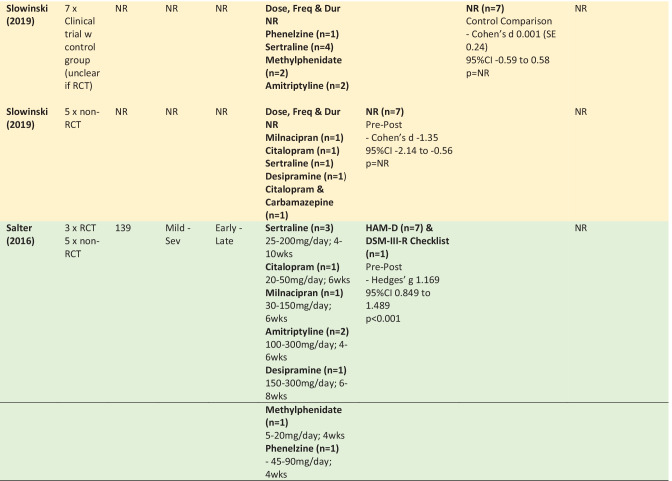
Summary of Meta-Analysis Findings from the 10 Meta-Analyses in the Umbrella Review

*BDI* Beck Depression Inventory, *BDI-II* Beck Depression Inventory – Second Edition, *DSM-III-R *Psychiatry Diagnostic & Statistical Manual of Mental Disorders–3rd Edition Revised, Freq. – Frequency, *HAM-D* Hamilton Rating Scale for Depression, *MADRS* Montgomery-Asberg Depression Rating Scale, *Mod.* Moderate, *Mths.* Months, *NR* Not reported, *NS* Not significant, *OR* Odds Ratio, *PHQ-9* Patient Health Questionnaire, *RCT* Randomised Controlled Trial, *SCL-20* Symptom Checklist – 20, *Sev.* Severe, *SMD* Standard Mean Difference, *Wks.* Weeks, 95% CI – 95% Confidence Interval

Colours refer to the methodological assessment; green—‘low’ risk of bias, yellow—‘intermediate’ risk of bias, red—‘high’ risk of bias

‘Pre-post’ data is from single group studies that have compared change in scores pre-intervention to post-intervention. ‘Control comparison’ data is from studies using two independent groups. Most ‘control comparison’ analyses examined differences in post-treatment outcomes between the treatment and control groups. A smaller number of reviews compared group differences in pre to post-intervention change – this is signified in the table by the word ‘change’

Where we have not provided p values – this is because they were not provided in the systematic review. At times, the review did state in the narrative text that the p value was not significant. Where this occurs we have recorded ‘NS’ for the p value

Salter et al. (2016) only reported harms in their discussion section and did not include them in their findings section

Barker-Collo et al. (2013), Price et al. (2011) and Rayner et al. (2010) are not included in the above table as they did not provide separate pooled estimates for studies examining pharmacotherapy for depression post TBI

Kreitzer et al. (2019) performed a second meta-analysis that examined change in depression scores from pre to post treatment. As this meta-analysis included a study that did not meet eligibility criteria for the current review (Horsfield et al., 2002), the pooled estimate could not be extracted

Effects sizes were interpreted as follows 0.2 ‘small’, 0.5 ‘moderate’ and 0.8 ‘large’ (Cohen, 1988)

^a^Time post injury categorised as: ≤ 1 year ‘Early’; > 1 year to 5 years ‘Mid’; > 5 years ‘Late’

^b^No definition of ‘non-responders’ provided

^c^The HAM-D Maier subscale measures: 1 (depressed mood), 2 (feelings of guilt), 7 (work and activities), 9 (agitation), 10 (anxiety/psychic), 11 (anxiety – somatic), 14 (genital symptoms)

^d^Responders defined as: decrease in final HAM-D score of more than 50%

**Table 5:** Risk of Bias Judgements for the 22 Systematic Reviews Included in the Umbrella Review

	**REVIEW VALIDITY**									**REVIEW QUALITY**		**TOTAL**
**Review**	**Clear question**	**Appropriate inclusion criteria**	**Appropriate search strategy**	**Adequate search sources and resources**	**Appropriate critical appraisal**	**Critical appraisal by ≥ 2 authors**	**Methods to minimize data extraction errors**	**Appropriate methods to combine studies**	**Publication bias assessed**	**Recommendations for policy/ practise supported**	**Appropriate directed for future research**	
Beedham et al. (2020)	Y	Y	Y	Y	Y	Y	Y*	Y*	Y	Y	Y	Low Y = 9; Y* = 2; N = 0; U = 0
Peppel et al. (2020)	Y	Y*	Y	Y	Y	Y	Y	Y	N	Y*	Y	Low Y = 8; Y* = 2; N = 1; U = 0
Gao et al. (2019)	Y	N	N	Y	Y	Y	Y	Y	N	Y	Y	Low Y = 8; Y* = 0; N = 3; U = 0
Kreitzer et al. (2019)	Y	U	Y	N	Y	Y	Y	Y	N	Y	Y	Low Y = 8; Y* = 0; N = 2; U = 1
Liu et al. (2019)	N	N	N	N	N	N	N	Y*	N	Y	Y	High Y = 2; Y* = 1; N = 8; U = 0
Reyes et al. (2019)	Y	Y	Y	Y	Y	Y	Y	Y*	N	Y	Y	Low Y = 9; Y* = 1; N = 1; U = 0
Slowinski et al. (2019)	Y	Y*	Y	Y	N	N	N	Y	Y	Y	Y	Intermediate Y = 7; Y* = 1; N = 3; U = 0
Paraschakis and Katsanos (2017)	Y	Y	Y	N	Y	Y	N	Y	N	N	Y	Intermediate Y = 7; Y* = 0; N = 4; U = 0
Yue et al. (2017)	Y	N	Y	N	N	N	N	Y*	N	Y	Y	High Y = 4; Y* = 1; N = 6; U = 0
Maksimowski and Tampi (2016)	N	N	Y	Y	Y	N	N	Y	N	Y	Y	Intermediate Y = 6; Y* = 0; N = 5; U = 0
Plantier et al. (2016)	Y	N	Y	N	N	N	U	Y	N	Y	N	High Y = 4; Y* = 0; N = 6; U = 1
Salter et al. (2016)	Y	Y	Y	Y	Y*	Y	Y	Y*	Y	Y	Y	Low Y = 9; Y* = 2; N = 0; U = 0
Barker-Collo et al. (2013)	N	Y	Y	Y	N	N	U	Y	Y	Y	Y	Intermediate Y = 7; Y* = 0; N = 3; U = 1
Guillamondegui et al. (2011)	Y	Y	Y	Y	U	Y	Y	Y	N	Y	Y	Low Y = 9; Y* = 0; N = 1; U = 1
Price et al. (2011)	Y	Y	Y	Y	Y	U	Y	Y	N	Y	N	Low Y = 8; Y* = 0; N = 2; U = 1
Wheaton et al. (2011)	Y	Y*	Y	N	U	N	N	Y*	Y	Y*	Y	High Y = 4; Y* = 3; N = 3; U = 1
Rayner et al. (2010)	Y	Y	Y	Y	Y	Y	Y	Y	Y	Y*	Y	Low Y = 10; Y* = 1; N = 0; U = 0
Fann et al. (2009a, b)	Y	Y*	Y	Y	Y	N	Y	Y	N	Y	Y	Low Y = 8; Y* = 1; N = 2; U = 0
Hardy (2009)	Y	Y	Y	Y	N	N	N	Y	N	Y	Y	Intermediate Y = 7; Y* = 0; N = 4; U = 0
Warden et al. (2006)	N	N	Y	N	Y	Y	Y	Y	N	U	Y	Intermediate Y = 6; Y* = 0; N = 4; U = 1
Comper et al. (2005)	Y	Y	Y	Y	U	Y	Y	Y	N	Y	Y	Low Y = 9; Y* = 0; N = 1; U = 1
Deb and Crownshaw (2004)	Y	Y	Y	Y	N	N	N	Y	N	Y	N	Intermediate Y = 6; Y* = 0; N = 5; U = 0
Total performance on each item across reviews	Y: 18 Y*: 0 U: 0 N: 4	Y: 11 Y*: 4 U: 1 N: 6	Y: 20 Y*: 0 U: 0 N: 2	Y: 15 Y*: 0 U: 0 N: 7	Y: 11 Y*: 1 U: 3 N: 7	Y: 11 Y*: 0 U: 1 N: 10	Y: 11 Y*: 1 U: 2 N: 8	Y: 16 Y*: 6 U: 0 N: 0	Y: 6 Y*: 0 U: 0 N: 16	Y: 17 Y*: 3 U: 1 N: 1	Y: 19 Y*: 0 U: 0 N: 3	

Y* is used to denote when a review fulfilled the criteria for an item, however, there were small caveats that may have introduced some minor bias. The explanation for each caveat is provided in Appendix 9

We derived an overall risk of bias judgement (low; intermediate; high) through examining performance across the 11 items, and detailed discussion to arrive at consensus

*N* No, *U* Unclear, *Y* Yes

Based on the scores across the 11 items, we classified 11 reviews as having a low risk of bias (Beedham et al., 2020; Peppel et al., 2020; Gao et al., 2019; Kreitzer et al., 2019; Reyes et al., 2019; Salter et al., 2016; Guillamondegui et al., 2011; Price et al., 2011; Rayner et al., 2010; Fann et al., 2009a; Comper et al., 2005), seven reviews as having an intermediate risk of bias (Slowinski et al., 2019; Paraschakis & Katsanos, 2017; Maksimowski & Tampi, 2016; Barker-Collo et al., 2013; Hardy, 2009; Neurobehavioral Guidelines Working et al., 2006; Deb & Crownshaw, 2004), and four as having a high risk of bias (Liu et al., 2019; Yue et al., 2017; Plantier et al., 2016; Wheaton et al., 2011). Across reviews, the most consistent area of bias was failure to assess for publication bias (6/22 included some assessment for publication bias). Other common areas of methodological weakness included unclear inclusion criteria, poor critical appraisal and lack of or insufficient methods to minimize data extraction errors. Areas of methodological rigour included appropriate and extensive search strategies and clear and explicit review questions.

A number of meta-analyses had high heterogeneity (I^2^ > 75%; (Higgins et al., 2003)). Furthermore, confidence intervals were not provided for the I^2^ statistic, this is important as evidence suggests that even for point estimates of 0% the confidence intervals can be wide and often exceed 50% (Ioannidis et al., 2007). Inconsistency across studies reduces the confidence of recommendations about treatment and should be explicitly addressed in reviews. Ideally, authors may conduct sub-group analyses and meta-regressions to explore heterogeneity, however, this may not be possible if the primary studies have not provided sufficient detail of study characteristics (to be used as independent variables in these analyses; e.g., drug dose, TBI severity).

## Discussion

We synthesized systematic reviews and meta-analyses on the effectiveness of pharmacotherapy for the management of post TBI depression in adults 16 years and over. Twenty-two reviews met inclusion criteria for the review. Six drug classes (SSRIs, TCAs, MAOIs, SNRIs, stimulants and anti-convulsants) and 10 different drugs were examined. Harms were not mentioned in half the reviews. We conclude that there is insufficient high quality and methodologically rigorous evidence to recommend prescribing any specific drug or drug class for post TBI depression. The findings do show, however, that depression post TBI is responsive to pharmacotherapy in at least some individuals. Possible reasons for the varied findings are discussed, along with recommendations for both prescribers and researchers.

### Main Findings

#### Change in Depression Scores

SSRIs have been the most extensively studied pharmacotherapeutic intervention for post TBI depression. The weight of the higher quality evidence did not find in favour of SSRIs and sertraline specifically as effective for post TBI depression across the spectrum of severity and time since injury. Although one review did report positive results for citalopram, the strength of this evidence is low given the pre-post analyses with no control group.

There have been considerably fewer published studies of TCAs and stimulants, and the possibility for publication bias in these findings must be acknowledged. Given the preliminary evidence to date, albeit from small sample sizes and studies of varied methodological quality, is mostly favourable for methylphenidate, TCAs and amitriptyline specifically, further trials of these drugs for post TBI depression seems appropriate.

The majority of meta-analyses that pooled across drug classes concluded in favour of treatment. However, the utility of such analyses is queried given it is unclear which drugs specifically were associated with the positive effect. Further, given many of these meta-analyses were pre-post designs with no control comparison group, it is not possible to control for natural recovery over time. Of those drugs that were only described in a single primary study, positive findings were reported for desipramine (TCA), moclobemide (MAOI), combination therapy of citalopram (SSRI) and carbamazepine (anti-convulsant), milnacipran (SNRI) and escitalopram (SSRI), with no significant effect of phenelzine (MAOI) found. No recommendations can be drawn from these findings, with further studies needed to allow for meta-analyses.

#### Harms

Comprehensive reporting of harms was only available in one high quality meta-analysis of sertraline, which showed no greater risk of harms in those given sertraline compared to placebo (Gao et al., 2019). These findings should be considered in the context of what is known about SSRI side effects from a substantial number of studies in non-TBI populations in which gastrointestinal issues, weight gain, sleep issues and sexual dysfunction are commonly reported (Carvalho et al., 2016; Ferguson, 2001). Prescribers must also consider the potentially deleterious effects of SSRIs on cognition and agitation when used in conjunction with other psychotropic agents (Yue et al., 2017).

Data on harms is also of particular importance for drugs such as methylphenidate and TCAs given the known potential to lower seizure threshold (Wroblewski et al., 1990; Barker-Collo et al., 2013), and the association of methylphenidate with anxiety, irritability, insomnia, reduced appetite and increases in heart rate and blood pressure (Kimko et al., 1999).

### Factors that may have Impacted Findings

#### Injury Severity

A clearer picture of depression pharmacotherapy may be achieved by stratifying pooled results by TBI severity. Most meta-analyses examined across the spectrum from mild to severe TBI, and did not provide sub-analyses comparing injury severity groups. Given the association between injury severity and post TBI depression remains unclear (Osborn et al., 2014; Singh et al., 2018; Ouellet et al., 2018; Mauri et al., 2014; Singh et al., 2019; Senathi-Raja et al., 2010), it is important to include stratified analyses where possible. When analyses are pooled across TBI severities, an overall non-significant finding may obscure a treatment effect specific to one TBI severity group. Alternatively, an overall significant result may be driven by treatment efficacy in only one severity sub-group from which concluding efficacy in other injury severities may be misleading. At a biological level, it is conceivable that medication metabolism may be impacted by injury severity, due to factors such as greater neuronal damage and disturbance to neurotransmitter systems, as well as greater disruption to cerebral blood flow and the blood brain barrier (Levine, 2013; Lo et al., 2001). Finally, the overall treatment plan for depressive symptoms after moderate to severe TBI may differ from that of mild TBI in whom symptoms are more likely to resolve over time (Barker-Collo et al., 2018; Theadom et al., 2018). As such, for individuals with mild TBI, evaluating the balance between possible symptom reduction and possible harms from a trial of pharmacotherapy is likely to be different.

#### TBI Specific Outcome Measure

Use of TBI-appropriate measures is also critical. It is likely that the use of sub-optimal measures in the primary studies contributed to mixed findings. A clear impact of this was seen in the evidence for sertraline and methylphenidate, in which the measures used changed the meta-analyses conclusions (Peppel et al., 2020). As discussed above, measures which include TBI sequalae may overstate depression at baseline, and underestimate change over time, as the TBI related sequalae are unlikely to be impacted by the pharmacotherapy (Peppel et al., 2020).

Measures such as the HADS, which purposefully omit items which are likely to overlap with symptoms of common medical disorders, are recommended as they may provide a more accurate means of detecting post TBI depression (Barker-Collo et al., 2015; Singh et al., 2018). Despite this, the HADS was not used in any primary study. Rather, the HAM-D was the most common measurement tool used within the primary studies, despite evidence suggesting it is not ideal for TBI samples and less responsive to treatment than other depression measures (Caplan et al., 2016).

#### Depression Entry Criteria

The lack of robust response to medications identified in some reviews may be related to participant’s low depression severity at baseline. The most common measures used, DSM criteria and HAM-D scores, may have inflated baseline depression scores due to overlap in symptoms of depression with common sequalae of TBI (Barker-Collo et al., 2015), resulting in samples with lower rates of actual depression symptoms. The cut-off values used may also have been too low. A patient level meta-analysis showed that HAM-D scores needed to be greater than 25 at baseline for the treatment to show a clinically meaningful difference (Fournier et al., 2010). No primary studies in the current review had depression entry criteria of HAM-D over 25, with the highest cut-off score being 18. This is of concern given evidence in non-TBI samples that depression severity is strongly associated with response to anti-depressant medication, with the most robust effects found in those with more severe depression (Fournier et al., 2010).

#### Depression Measurement

The measurement and conceptualisation of depression within the primary studies may have also introduced bias. The included depression measurement tools were all multi-dimensional and include a diverse set of symptoms. Research suggests that the overlap in symptoms captured on common depression rating scales is low (Fried, 2016). This may lead to research results idiosyncratic to the scales used (Fried, 2016), and poor correspondence between depression measures within the same individuals. There is some evidence of this issue within TBI samples, with research showing the HADS does not strongly correspond with clinical diagnosis of depression on DSM-IV-TR (Whelan-Goodinson et al., 2009).

The heterogeneity in depression scales reflects the diversity of clinical opinions regarding what depression is (Fried, 2016). Given this diversity, the idea of depression as a homogenous concept has been questioned, and it has been suggested that depression could be better understood by examining individual symptoms (Fried et al., 2016). This new approach could provide a more sensitive examination of drug efficacy by evaluating treatment response at the individual symptom level or to more homogenous symptom dimensions (Fried, 2016; Fried et al., 2016; Fornaro et al., 2021). Re-analysis of anti-depressant trials in non-TBI populations have indeed shown that single item/ symptom end points are more sensitive to treatment effect than sum scores from rating scales (Hieronymus et al., 2016). For example, a re-analysis of SSRI trials shows that while 18 out of 32 comparisons (56%) failed to separate active drug from placebo at week 6 with respect to reduction in HAM-D, only 3 out of 32 comparisons (9%) were negative when depressed mood was used as an effect parameter (Hieronymus et al., 2016). Notably, depressed mood has been identified as the most frequent MDD symptoms at 6 and 12 months post injury (Gould et al., 2011a), suggesting this symptom may be a sensitive marker for anti-depressant treatment efficacy in TBI samples.

#### Placebo Effect

The non-significant results for the control-comparison meta-analyses may have been contributed to, in part, by a placebo effect in the control group. Such placebo effects have been seen in other recent TBI pharmacotherapy studies (Hammond et al., 2014, 2015, 2018) and are common in depression trials more broadly. Factors driving the placebo effect include therapeutic alliance, participation in a research study, anxiety reduction and hope, as well as placebo neurobiology including top-down cortical regulation, reward system activation and dopaminergic and serotoninergic neurotransmission (Polich et al., 2018).

It has been suggested that TBI survivors may be highly responsive to placebos, through both neurobiological pathways and psychosocial factors (Polich et al., 2018). Interpersonal factors and access to healthcare providers may be particularly salient for TBI survivors who may be experiencing social isolation particularly in the chronic post injury period where access to services may have reduced (Polich et al., 2018; Hammond et al., 2015; Lefkovits et al., 2020). From a neurobiological perspective, there is overlap in some of the dysregulated systems post TBI that are targeted in pharmacological treatment and those implicated in the placebo response, including the dopaminergic and serotonergic pathways (Polich et al., 2018). As a result, those taking a placebo may experience similar activation of these systems, while for those in the treatment group, placebo effects may either act alone to drive symptom improvement, or act synergistically with the active drug to promote an even greater effect (Polich et al., 2018).

#### Length of Treatment

The variation in intervention duration may have confounded response to treatment and impacted the combined results. Indeed, one of the meta-analyses included in this review found that length of treatment was significantly associated with change in depression symptom severity, and suggested that greater reductions in depressive symptomatology might have been observed if treatment periods had been prolonged (Salter et al., 2016).

### Recommendations for Research

#### Recommendations for Primary Studies

To allow more robust control-comparison meta-analyses to be conducted, primary studies must include a control group. Although control-comparison analyses are not without limitations due to the placebo effect, the findings from pre-post analyses cannot be reliably discerned from natural change over time. Recruitment and maintenance of patients is always an issue, but consistency in research protocols will allow for more precise meta-analyses across smaller studies. Taken further, the option of prospective meta-analyses, in which different teams of researchers work together to conduct a set of studies addressing the same question, and synthesize the results once all studies are completed, could be explored (Thomas et al., 2019).

Researchers and clinicians should carefully consider the item content of the measures used for study entry and outcome assessment. Ideally, measures such as the HADS that omit items that are likely to overlap with TBI related symptoms should be used. Examining single items from these measures, such as those focussed on depressed mood specifically, may be more sensitive to treatment efficacy. Furthermore, other clinically meaningful outcome measures such as quality of life and functional status should also be included.

Standardized reporting of non-responders, partial responses (i.e., 25–50% improvement on a standard symptoms scale), full responses (i.e., > 50% improvement) and remission (i.e., absence of symptoms) would also facilitate easier comparison across studies. Inadequate symptom improvement (i.e., partial responses or no responses) to anti-depressant medication in non-TBI samples are common (Corey-Lisle et al., 2004; Fournier et al., 2010; Kirsch et al., 2008; Xiao et al., 2018), and it is important to understand which TBI survivors may be at-risk. In the cases of partial or non-response to medications, drug augmentation could be trialled as part of the study design. This would enhance the clinical applicability of findings, given that drug augmentation following partial or non-response is a common clinical pathway (Fredman et al., 2000; Gaynes et al., 2008), which has not been explored in the TBI literature. There are a number of meta-analyses in non-TBI samples examining the efficacy, acceptability and tolerability of augmentation agents for treatment resistant depression from which guidance could be sort while TBI specific evidence builds (Strawbridge et al., 2019; Zhou et al., 2015).

Following cessation of intervention, it is also important to assess the maintenance of treatment effects and rates of relapse for specific anti-depressant drugs. Based on preliminary evidence in TBI samples (Rapoport et al., 2010) and substantial evidence in non-TBI samples (Keller et al., 1992; Gaynes et al., 2008; Ramana et al., 1995), it would be expected that a number of patients will experience relapse and recurrence of symptoms after a single medication trial. Indeed, for most non-TBI patients research suggests remission will require repeated trials of sufficiently sustained anti-depressants, with only a minority of patients entering long-term remission after one medication trial (Gaynes et al., 2008). Relapse prevention management has been examined in TBI samples by providing continuation therapy with citalopram following remission of symptoms (Rapoport et al., 2010). There was, however, no significant impact on relapse prevention. Further research on effective relapse prevention strategies in TBI populations are required.

Studies should include examination of known relapse risk factors in non-TBI populations (e.g., comorbid anxiety, age of onset, neuroticism, greater initial severity of depression; (Buckman et al., 2018; Ramana et al., 1995) as well exploring possible TBI-specific factors. Following up participants may help to produce predictive models so those with higher relapse propensity can be more actively managed. This is important as evidence indicates that risk of depressive recurrence and treatment resistance in non-TBI samples increases as the illness becomes more highly recurrent (Keller et al., 1992; Gaynes et al., 2008). Finally, longer follow-up periods post drug cessation would allow for more accurate understanding of drug tolerance, and the longevity and burden of adverse events.

#### Recommendations for Systematic Reviews

Generalizability of results from reviews would be improved by including clear details of participant characteristics including age, gender, injury severity and time since injury. Analyses should be stratified by injury severity where possible. Although it is acknowledged that depressive disorders may occur after TBI of any severity, metabolism of medication may be impacted by injury severity (Levine, 2013; Lo et al., 2001).

It is of critical importance for primary studies to measure harms, and for systematic reviews to include these as part of their primary outcomes. Harms were not mentioned in half of the reviews, and only six commented on study-drop outs, which is an important indicator of drug acceptability. Although harms data from non-TBI populations can provide useful preliminary guidance, harms must be studied within TBI populations given the possible impact of abnormal brain function on the metabolism of drugs (Levine, 2013; Lo et al., 2001; Waldron-Perrine et al., 2008). Harms may also be more burdensome for TBI survivors, due to the overlap in TBI related sequalae and common side effects of anti-depressants such as sleep problems and sexual dysfunction (Castriotta et al., 2007; Mathias & Alvaro, 2012; Hibbard et al., 2000; Ponsford, 2003; Downing et al., 2013; Ferguson, 2001).

Finally, the utility of broad reviews that include meta-analyses combining medical conditions or both pharmacological and non-pharmacological treatments is unclear, and indeed may contribute further methodological ambiguity to the interpretation of pharmacotherapy intervention.

### Advice to Prescribers

#### Pharmacotherapy

Although the findings of this umbrella review do not provide support for any specific drug class, they do show that post TBI depression is a treatment responsive condition in at least some individuals. Lack of significant findings may have been contributed to by small pooled samples, the outcome measures used, short treatment duration, low methodological quality and low severity of depression at baseline. While the evidence base develops for specific drugs, this umbrella review suggests that a trial of anti-depressants may be sensible with careful monitoring of harms objective assessment of depressive symptoms, and discontinuation if no benefit is observed. The selection of which anti-depressant to prescribe should be made considering the likelihood of responsiveness to the treatment and vulnerability to the adverse events associated with that drug for each individual (Carvalho et al., 2016). Once a patient has been started on an anti-depressant they may benefit from an alternative or adjunctive medication if the agent prescribed first does not achieve a depression remission (Silverberg & Panenka, 2019).

One final consideration is the length of treatment. Research in non-TBI populations has shown that ongoing anti-depressant use may only be appropriate for people with high risk of relapse, with the optimal treatment period not yet known for those deemed at low risk of relapse (Geddes et al., 2003). Treatment duration must also be balanced with the risk of adverse events, with non-TBI evidence suggesting that greater duration of treatment with anti-depressants is associated with longevity of adverse events (Carvalho et al., 2016). On the other hand, premature discontinuation of therapy may give the impression of less than optimal response to treatment in an individual who might otherwise show treatment gains. This may be particularly problematic in TBI samples who may require different medication management (i.e., different dosage, frequency) to achieve a treatment response as compared non-TBI samples (Morgan et al., 2012; Dinan & Mobayed, 1992). Adequate follow-up with symptom monitoring and drug augmentation as required is recommended.

### Other Intervention Possibilities

Given the multi-faceted aetiology of post TBI depression, the value of providing multi-modal treatment should be further explored, with pharmacotherapy forming one part of a comprehensive biopsychosocial response to depression treatment (Fann et al., 2009a). In light of promising meta-analyses findings for psychotherapy in post ABI depression (Stalder-Lüthy et al., 2013), a combination of these modalities may be prudent. Evidence in non-TBI populations has found that a combination of pharmacotherapy and psychotherapy was more effective in achieving a treatment response than pharmacotherapy alone (Cuijpers et al., 2020). Psychotherapy also has the additional benefit of being able to focus on issues that may be having a bi-directional impact on depression post TBI such as fatigue, changes to identity and issues with social relationships and return to work. Exercise interventions and rTMS (Repetitive Transcranial Magnetic Stimulation) also have a growing evidence base mostly in non-TBI populations, and may be particularly helpful in cases of medication refractory depression (Hu et al., 2020; Hoy et al., 2019; Fann et al., 2009b).

### Limitations

Our review was limited to publications in English only, resulting in one review being excluded (Richard et al., 2003). This review was published in 2003, and as such it is unlikely to contain papers not captured in the 22 reviews included in this umbrella review that were all published after this date. Seven of the reviews did not restrict their search to English language only, suggesting that any relevant primary studies not available in English would have been included in those reviews.

### Conclusion

Debilitating and pervasive symptoms of depression often develop following TBI, and greatly disrupt the lives of survivors and their families. In the absence of a stong evidence base for any specific drug, tentative trials of anti-depressant medication weighing vulnerability to risk factors seems appropriate. To progress the evidence base, primary studies should use a control-comparison design, TBI appropriate measures of depression and symptom-level analysis, and include a follow-up post intervention cessation. Finally, measurement and reporting of harms in both primary studies and systematic reviews is critical to understand the tolerability of commonly used drugs in this population.

## Supplementary Information

Below is the link to the electronic supplementary material.

Supplementary file1 (DOCX 36 KB)

Supplementary file2 (DOCX 44 KB)

Supplementary file3 (DOCX 28 KB)

Supplementary file4 (DOCX 16 KB)

Supplementary file5 (DOCX 44 KB)

Supplementary file6 (DOCX 20 KB)

Supplementary file7 (DOCX 27 KB)

Supplementary file8 (DOCX 29 KB)

Supplementary file9 (DOCX 88 KB)

## Data Availability

The data extraction form has been provided as an appendices.
