# Supplementary material for: Effectiveness of Pharmacotherapy for Depression after Adult Traumatic Brain Injury: an Umbrella Review
Source: Neuropsychol Rev. 2022 Jun 14;33(2):393–431. doi: 10.1007/s11065-022-09543-6 (PMC10148771; doi:10.1007/s11065-022-09543-6)
Supplement: Supplementary file 1 — Supplementary file1 (DOCX 36 KB) [file 11065_2022_9543_MOESM1_ESM.docx]

**Appendix 1**

**Full search strategies searches for the umbrella review and systematic review**

**Full Search Strategy for the Umbrella Review**

**Existing Systematic Review Search Filters Added to Current Search**

CINAHL: <http://libguides.sph.uth.tmc.edu/search_filters/cinahl_filters>

Embase, MEDLINE, and PsycINFO: <https://www.cadth.ca/resources/finding-evidence/strings-attached-cadths-database-search-filters#syst>

**Appendix 1: Search Strategies**

**A. CINAHL**

Advanced Search

Limitters - Exclude MEDLINE records

( ( (MH "Brain Injuries") OR (MH "Brain Concussion") OR (MH "Left Hemisphere Injuries") OR (MH "Right Hemisphere Injuries") OR (MH "Pneumocephalus") OR (MH "Head Injuries") ) OR TI ( ((Brain OR Cerebr* OR Crani* OR "Crushing Skull" OR "Diffuse Axonal" OR Head OR Hemisphere*) N1 (Injur* OR Trauma*)) OR ((Cerebr* OR Crani* OR Head) N1 (Lesion* OR Wound*)) OR ((Posttraumatic OR Traumatic) N1 (Encephalopath*)) OR ((Traumatic) N1 (Brain OR Cerebr*)) OR Concuss* OR DAI OR DAIs OR Pneumocephalus OR TBI OR TBIs ) OR AB ( ((Brain OR Cerebr* OR Crani* OR "Crushing Skull" OR "Diffuse Axonal" OR Head OR Hemisphere*) N1 (Injur* OR Trauma*)) OR ((Cerebr* OR Crani* OR Head) N1 (Lesion* OR Wound*)) OR ((Posttraumatic OR Traumatic) N1 (Encephalopath*)) OR ((Traumatic) N1 (Brain OR Cerebr*)) OR Concuss* OR DAI OR DAIs OR Pneumocephalus OR TBI OR TBIs ) ) AND ( ( [MH "Adrenergic Beta-Antagonists"] OR [MH "Antianxiety Agents"] OR [MH Anticonvulsants] OR [MH "Antidepressive Agents"] OR [MH "Antipsychotic Agents"] OR [MH Atomoxetine] OR [MH "Antianxiety Agents, Benzodiazepine"] OR [MH "Clorazepate Dipotassium"] OR [MH "Desvenlafaxine Succinate"] OR [MH "Duloxetine Hydrochloride"] OR [MH "Lithium Carbonate"] OR [MH Paliperidone] OR [MH Quetiapine] OR [MH Venlafaxine] OR [MH Alprazolam] OR [MH Amitriptyline] OR [MH Amoxapine] OR [MH Aripiprazole] OR [MH Atenolol] OR [MH Benztropine] OR [MH Bupropion] OR [MH Buspirone] OR [MH Carbamazepine] OR [MH Chlordiazepoxide] OR [MH Chlorpromazine] OR [MH Citalopram] OR [MH Clomipramine] OR [MH Clonazepam] OR [MH Clozapine] OR [MH Desipramine] OR [MH Dextroamphetamine] OR [MH Diazepam] OR [MH Doxepin] OR [MH Droperidol] OR [MH Estazolam] OR [MH Eszopiclone] OR [MH Flunitrazepam] OR [MH Fluoxetine] OR [MH Fluphenazine] OR [MH "Fluvoxamine Maleate"] OR [MH Haloperidol] OR [MH Imipramine] OR [MH Lithium] OR [MH Lorazepam] OR [MH Loxapine] OR [MH Methylphenidate] OR [MH Midazolam] OR [MH "Molindone Hydrochloride"] OR [MH Nortriptyline] OR [MH Oxazepam] OR [MH Paroxetine] OR [MH Phenobarbital] OR [MH Pindolol] OR [MH Pregabalin] OR [MH Prochlorperazine] OR [MH Promethazine] OR [MH Propranolol] OR [MH Risperidone] OR [MH Selegiline] OR [MH "Sertraline Hydrochloride"] OR [MH Temazepam] OR [MH Thioridazine] OR [MH Thiothixene] OR [MH Trazodone] OR [MH Triazolam] OR [MH "Trifluoperazine Hydrochloride"] OR [MH "Valproic Acid"] ) OR TI ( ((Adrenergic) N1 Beta N2 (Antagonist OR Block*)) OR Anti Anxiety OR Anti Convuls* OR Anti Depress* OR Anti Epilep* OR Anti Psychotic* OR Antianxiety OR Anticonvuls* OR Antidepress* OR Antiepilep* OR Antipsychotic* OR Anxiolytic* OR Benzodiazepine* OR (Beta N1 Block*) OR (Beta N1 Adrenergic N2 Block*) OR Thymoanaleptic* OR Thymoleptic* OR Agomelatine OR "S 20098" OR S20098 OR Thymanax OR Valdoxan OR "AGO 178" OR AGO178 OR Alprazolam OR Alprazolan OR "Apo Alpraz" OR ApoAlpraz OR Cassadan OR "D 65MT" OR D65MT OR Xanax OR Tafil OR Trankimazin OR "Novo Alprazol" OR NovoAlprazol OR "Nu Alpraz" OR NuAlpraz OR Ralozam OR "U-31,889" OR "U31,889" OR Alprox OR Esparon OR Kalma OR Amisulpride OR Sultopride OR Barnetil OR "DAN 2163" OR Solian OR "LIN 1418" OR Amitriptyline OR Amineurin OR Amitrip OR Amitriptylin OR Amitrol OR Tryptine OR ApoAmitriptyline OR Damilen OR Domical OR Laroxyl OR Endep OR Lentizol OR Novoprotect OR Saroten OR Sarotex OR Syneudon OR Triptafen OR Tryptizol OR Tryptanol OR Elavil OR Anapsique OR Amoxapine OR Desmethylloxapine OR "CL 67,772" OR "CL67,772" OR Demolox OR Asendin OR Defanyl OR Asendis OR Aripiprazole OR Aripiprazol OR "OPC 14597" OR Abilify OR Asenapine OR Saphris OR "OR G 5222" OR Atenolol OR Tenormine OR Tenormin OR "ICI 66082" OR ICI66082 OR Atomoxetine OR Tomoxetine OR Strattera OR "LY 139603" OR Benztropine OR Benzatropine OR Bensylate OR PMSBenztropine OR Cogentin OR Cogentinol OR Methylbenztropine OR ApoBenztropine OR Brexpiprazole OR Bromazepam OR BromaLich OR "Bromaz 1A Pharma" OR Bromazanil OR "Bromazep von CT" OR Durazanil OR Lexotan OR Lexotanil OR Lexatin OR Lexomil OR "Ro 5-3350" OR "Ro 53350" OR Anxyrex OR Bupropion OR Amfebutamone OR Zyntabac OR Quomen OR Wellbutrin OR Zyban OR Buspirone OR "MJ 9022 1" OR MJ90221 OR Neurosine OR Busp OR Anxut OR Buspar OR Bespar OR Carbamazepine OR Tegretol OR Carbazepin OR Epitol OR Finlepsin OR Neurotol OR Amizepine OR Cariprazine OR "RGH 188" OR Chlordiazepoxide OR Methaminodiazepoxide OR Librium OR Chlozepid OR Elenium OR Chlorpromazine OR Thorazine OR Aminazine OR Largactil OR Chlordelazine OR Contomin OR Fenactil OR Propaphenin OR Chlorazine OR Citalopram OR Cytalopram OR "Lu 10 171" OR Lu10171 OR Escitalopram OR Lexapro OR Clobazam OR "HR 376" OR Onfi OR "LM 2717" OR Frisium OR Urbanyl OR Clomipramine OR Chlomipramine OR Chlorimipramine OR Hydiphen OR Anafranil OR Clonazepam OR "Ro 5 4023" OR "Ro 54023" OR Antelepsin OR Rivotril OR Clopenthixol OR Zuclopenthixol OR Cisordinol OR Clorazepate OR Chlorazepate OR Tranxene OR Tranxilium OR "4306 CB" OR Clozapine OR Clozaril OR Leponex OR Desipramine OR Desmethylimipramine OR Demethylimipramine OR Norpramin OR Pertofrane OR Pertrofran OR Pertofran OR Petylyl OR Desvenlafaxine OR "O Desmethylvenlafaxine" OR "WY 45,233" OR "WY 45,233" OR "WY45,233" OR "WY 45233" OR WY45233 OR Pristiq OR Dextroamphetamine OR Dexamphetamine OR Dexamfetamine OR "Dextro Amphetamine" OR "D Amphetamine" OR Dexedrine OR DextroStat OR Oxydess OR Diazepam OR Diazemuls OR Faustan OR Valium OR Seduxen OR Sibazon OR Stesolid OR Apaurin OR Relanium OR "Valproic Acid" OR Divalproex OR "Propylisopropylacetic Acid" OR "2 Propylpentanoic Acid" OR Convulsofin OR Depakene OR Depakine OR Depakote OR Vupral OR Valproate OR Ergenyl OR "Dipropyl Acetate" OR Domperidone OR Domperidon OR Domidon OR Gastrocure OR Motilium OR Nauzelin OR Peridys OR "R 33,812" OR "R33,812" OR "R 33812" OR R33812 OR Dothiepin OR Dosulepin OR Prothiaden OR Doxepin OR Deptran OR Desidox OR Doneurin OR Doxepia OR Espadox OR Mareen OR Prudoxin OR Quitaxon OR Sinequan OR Sinquan OR Zonalon OR Xepin OR Aponal OR ApoDoxepin OR Droperidol OR Inapsine OR Dehidrobenzperidol OR Dehydrobenzperidol OR Droleptan OR Duloxetine OR "LY 248686" OR LY248686 OR "LY 227942" OR LY227942 OR Cymbalta OR Estazolam OR Tasedan OR ProSom OR "D 40TA" OR D40TA OR Nuctalon OR Eszopiclone OR Lunesta OR Estorra OR Flunitrazepam OR Fluridrazepam OR Flunibeta OR Flunimerck OR Fluninoc OR Rohypnol OR Rohipnol OR Narcozep OR "Flunizep von CT" OR "RO 5 4200" OR RO54200 OR Fluoxetine OR Fluoxetin OR "Lilly 110140" OR Lilly110140 OR Sarafem OR Prozac OR Flupenthixol OR Flupentixol OR Emergil OR Fluanxol OR Fluphenazine OR Flufenazin OR Lyogen OR Prolixin OR Fluvoxamine OR Fluvoxadura OR Fluvoxamin OR Fluvoxamina OR Luvox OR Fevarin OR Floxyfral OR Dumirox OR Faverin OR Desiflu OR "DU 23000" OR DU23000 OR Guanfacine OR Tenex OR Lon798 OR "BS 100 141" OR BS100141 OR Estulic OR Haloperidol OR Haldol OR Iloperidone OR Zomaril OR Fanapt OR "HP 873" OR Imipramine OR Imizin OR Norchlorimipramine OR Imidobenzyle OR Tofranil OR Melipramine OR Pryleugan OR Janimine OR Isocarboxazid OR Lamotrigine OR Crisomet OR Lamictal OR Lamiktal OR "BW 430C" OR Labileno OR Methotrimeprazine OR Levomepromazine OR Levopromazine OR Levomeprazin OR Tisercin OR Tizercine OR Tizertsin OR Lithium OR Dilithium OR Lithane OR Lithobid OR Lithonate OR "CP-15,467 61" OR "CP15,46761" OR Micalith OR "NSC 16895" OR NSC16895 OR Priadel OR "Quilinorm Retard" OR Quilinormretard OR Eskalith OR Lithotabs OR Lorazepam OR Ativan OR Temesta OR "Orfidal Wyeth" OR Donix OR Duralozam OR Durazolam OR Idalprem OR Laubeel OR "Lorazep von CT" OR "Novo Lorazem" OR NovoLorazem OR "Nu Loraz" OR NuLoraz OR Sedicepan OR Sinestron OR Somagerol OR Tolid OR "WY 4036" OR WY4036 OR ApoLorazepam OR Loxapine OR Cloxazepine OR Oxilapine OR Loxitane OR Loxipine OR Loxapinsuccinate OR "CL 71,563" OR "CL71,563" OR Lurasidone OR "SM 13496" OR SM13496 OR "SM-13,496" OR "SM13,496" OR Latuda OR Methylphenidate OR Metadate OR Equasym OR Methylin OR Concerta OR Phenidylate OR Ritalin OR Ritaline OR Tsentedrin OR Centedrin OR Daytrana OR Mianserin OR Tolvon OR Lerivon OR Org GB 94 OR Midazolam OR Dormicum OR Versed OR "Ro 21 3981" OR "Ro 213981" OR Milnacipran OR Midalcipran OR Levomilnacipran OR Savella OR "F 2207" OR Ixel OR Mirtazapine OR "6 Azamianserin" OR Esmirtazapine OR Remeron OR Remergil OR Zispin OR Norset OR Rexer OR "Org 50081" OR " OR G 3770" OR Moclobemide OR Moclobamide OR Arima OR Aurorix OR Manerix OR Moclamine OR Aurorex OR Deprenorm OR Feraken OR Moclobemid OR Moclobeta OR Moclodura OR Moclonorm OR Rimoc OR "Ro 11 1163" OR Modafinil OR Benzhydrylsulfinylacetamide OR "CRL 40476" OR Vigil OR Provigil OR Sparlon OR Alertec OR Modiodal OR Molindone OR Moban OR Nefazodone OR Rulivan OR Serzone OR Dutonin OR Nefadar OR Menfazona OR Nitrazepam OR Nitrodiazepam OR "Dormo Puren" OR Eatan OR Imadorm OR Imeson OR Mogadon OR Nitrazadon OR Nitrazep OR Novanox OR Radedorm OR Remnos OR Serenade OR Somnite OR Alodorm OR Dormalon OR Nortriptyline OR Desmethylamitriptylin OR Desitriptyline OR Aventyl OR Paxtibi OR Allegron OR Norfenazin OR Pamelor OR Nortrilen OR Olanzapine OR Zolafren OR "LY 170052" OR Zyprexa OR "LY 170053" OR Oxazepam OR Serax OR Tazepam OR Adumbran OR Oxcarbazepine OR Timox OR Trileptal OR "GP 47680" OR Paliperidone OR "9 OH Risperidone" OR "9 Hydroxy Risperidone" OR "9 Hydroxyrisperidone" OR Invega OR "R 76477" OR R76477 OR Paroxetine OR "BRL 29060" OR BRL29060 OR "FG 7051" OR FG7051 OR Seroxat OR Paxil OR Aropax OR Periciazine OR Propericiazine OR Pericyazine OR Neuleptil OR Neuleptyl OR Aolept OR Phenelzine OR "Beta Phenylethylhydrazine" OR "2 Phenethylhydrazine" OR Fenelzin OR Phenethylhydrazine OR Nardelzine OR Nardil OR Phenobarbital OR Phenobarbitone OR "Phenylethylbarbituric Acid" OR Phenemal OR Phenylbarbital OR Hysteps OR Luminal OR Gardenal OR Pindolol OR Prindolol OR Visken OR "LB 46" OR LB46 OR Prazepam OR Lysanxia OR Reapam OR Centrax OR Demetrin OR Pregabalin OR "3 Isobutyl GABA" OR Lyrica OR "CI 1008" OR CI1008 OR Prochlorperazine OR Compazine OR Promazine OR Sparine OR Sinophenin OR Protactyl OR Promethazine OR Prometazin OR Proazamine OR Rumergan OR Diprazin OR Phenergan OR Phenargan OR Phensedyl OR Pipolfen OR Pipolphen OR Promet OR Prothazin OR Pyrethia OR Remsed OR Atosil OR Diphergan OR Propranolol OR Propanolol OR Inderal OR Avlocardyl OR "AY 20694" OR AY20694 OR Rexigen OR Dexpropranolol OR Dociton OR Obsidan OR Obzidan OR Anaprilin OR Anapriline OR Betadren OR Protriptyline OR Vivactil OR Quetiapine OR "ICI 204,636" OR "ICI 204636" OR ICI204636 OR Seroquel OR Reboxetine OR Vestra OR Remoxipride OR "FLA 731" OR FLA731 OR Risperidone OR Risperdal OR Risperidal OR "R 64,766" OR "R64,766" OR "R 64766" OR R64766 OR Selegiline OR Selegyline OR "L Deprenyl" OR "E 250" OR E250 OR Eldepryl OR Emsam OR Zelapar OR Deprenil OR Deprenalin OR Yumex OR Jumex OR Humex OR Deprenyl OR Sertindole OR Serlect OR "Lu 23 174" OR Serdolect OR Sertraline OR Zoloft OR Altruline OR Lustral OR Aremis OR Besitran OR Sealdin OR Gladem OR Sulpiride OR Sulperide OR Arminol OR Deponerton OR Meresa OR Desisulpid OR Digton OR Dogmatil OR Dolmatil OR Eglonyl OR Ekilid OR Guastil OR Lebopride OR Neogama OR Pontiride OR Psicocen OR Sulp OR Sulpitil OR Sulpivert OR Sulpor OR Synedil OR Tepavil OR Aiglonyl OR Temazepam OR Hydroxydiazepam OR Methyloxazepam OR Signopam OR Tenox OR "WY 3917" OR WY3917 OR Dasuen OR Euhypnos OR Levanxol OR "Norkotral Tema" OR Normison OR Nocturne OR Temtabs OR Normitab OR Nortem OR Planum OR "Pronervon T" OR Remestan OR Restoril OR "Ro 5 5345" OR Ro55345 OR "SaH 47 603" OR "SaH 47603" OR Temaze OR "Temazep von CT" OR Thioridazine OR ApoThioridazine OR Meleril OR Melleril OR Melleryl OR Mellaril OR Melleretten OR Melzine OR Thiozine OR Sonapax OR Thioridazineneurazpharm OR Aldazine OR Rideril OR Thiothixene OR Tiotixene OR Navane OR Topiramate OR USL255 OR "McN 4853" OR Topamax OR Epitomax OR Tranylcypromine OR "Trans 2 Phenylcyclopropylamine" OR Jatrosom OR Transamine OR Parnate OR Trazodone OR Tradozone OR "AF 1161" OR AF1161 OR Deprax OR Desyrel OR Molipaxin OR Trittico OR Thombran OR "Trazodon Hexal" OR "Trazodon Neuraxpharm" OR Trazon OR Triazolam OR "U 33,030" OR "U33,030" OR Halcion OR Trilam OR "Apo Triazo" OR Trifluoperazine OR Trifluoroperazine OR Trifluperazine OR Eskazine OR Flupazine OR Terfluzine OR Triftazin OR Stelazine OR Trimipramine OR Trimeprimine OR Herphonal OR Trimineurin OR NovoTripramine OR Rhotrimine OR Stangyl OR Surmontil OR Trimidura OR Trimineurin OR Trimipramin OR "Apo Trimip" OR ApoTrimip OR Eldoral OR Venlafaxine OR "Wy 45030" OR Wy45030 OR "Wy 45,030" OR "Wy45,030" OR Effexor OR Trevilor OR Vandral OR Efexor OR Dobupal OR Vigabatrin OR "Gamma Vinyl GABA" OR "Gamma Vinyl Gamma Aminobutyric Acid" OR Sabril OR Sabrilex OR Vortioxetine OR Brintellix OR "Lu AA21004" OR LuAA21004 OR Zaleplon OR "SKP 1041" OR Sonata OR Zelepion OR Starnoc OR "CL 284,846" OR "CL284,846" OR "CL 284846" OR "L 846" OR Ziprasidone OR Ziprazidone OR "CP 88,059" OR "CP 88059" OR Zolpidem OR Amsic OR Bikalm OR Dalparan OR "SL 80.0750" OR "SL 800750 23 N" OR Stilnoct OR Stilnox OR Zodormdura OR Zoldem OR Zolirin OR "Zolpi Lich" OR Zolpinox OR Zolpimist OR Ambien OR Zopiclone OR Zop OR Zopicalma OR Zopiclodura OR Zopiclon OR Zopitan OR Zorclone OR Imovane OR Ximovan OR Zimovane OR Limovan OR Optidorm OR Rhovane OR "RP 27 267" OR Siaten OR Somnosan OR Zileze OR Zimoclone OR "Zopi Puren" OR Zopicalm OR Zotepine OR Zoleptil OR Nipolept OR Zuclopenthixol OR Zuclopentixol OR Clopixol OR Zuclopenthixole OR Acuphase ) OR AB ( ((Adrenergic) N1 Beta N2 (Antagonist OR Block*)) OR Anti Anxiety OR Anti Convuls* OR Anti Depress* OR Anti Epilep* OR Anti Psychotic* OR Antianxiety OR Anticonvuls* OR Antidepress* OR Antiepilep* OR Antipsychotic* OR Anxiolytic* OR Benzodiazepine* OR (Beta N1 Block*) OR (Beta N1 Adrenergic N2 Block*) OR Thymoanaleptic* OR Thymoleptic* OR Agomelatine OR "S 20098" OR S20098 OR Thymanax OR Valdoxan OR "AGO 178" OR AGO178 OR Alprazolam OR Alprazolan OR "Apo Alpraz" OR ApoAlpraz OR Cassadan OR "D 65MT" OR D65MT OR Xanax OR Tafil OR Trankimazin OR "Novo Alprazol" OR NovoAlprazol OR "Nu Alpraz" OR NuAlpraz OR Ralozam OR "U-31,889" OR "U31,889" OR Alprox OR Esparon OR Kalma OR Amisulpride OR Sultopride OR Barnetil OR "DAN 2163" OR Solian OR "LIN 1418" OR Amitriptyline OR Amineurin OR Amitrip OR Amitriptylin OR Amitrol OR Tryptine OR ApoAmitriptyline OR Damilen OR Domical OR Laroxyl OR Endep OR Lentizol OR Novoprotect OR Saroten OR Sarotex OR Syneudon OR Triptafen OR Tryptizol OR Tryptanol OR Elavil OR Anapsique OR Amoxapine OR Desmethylloxapine OR "CL 67,772" OR "CL67,772" OR Demolox OR Asendin OR Defanyl OR Asendis OR Aripiprazole OR Aripiprazol OR "OPC 14597" OR Abilify OR Asenapine OR Saphris OR "OR G 5222" OR Atenolol OR Tenormine OR Tenormin OR "ICI 66082" OR ICI66082 OR Atomoxetine OR Tomoxetine OR Strattera OR "LY 139603" OR Benztropine OR Benzatropine OR Bensylate OR PMSBenztropine OR Cogentin OR Cogentinol OR Methylbenztropine OR ApoBenztropine OR Brexpiprazole OR Bromazepam OR BromaLich OR "Bromaz 1A Pharma" OR Bromazanil OR "Bromazep von CT" OR Durazanil OR Lexotan OR Lexotanil OR Lexatin OR Lexomil OR "Ro 5-3350" OR "Ro 53350" OR Anxyrex OR Bupropion OR Amfebutamone OR Zyntabac OR Quomen OR Wellbutrin OR Zyban OR Buspirone OR "MJ 9022 1" OR MJ90221 OR Neurosine OR Busp OR Anxut OR Buspar OR Bespar OR Carbamazepine OR Tegretol OR Carbazepin OR Epitol OR Finlepsin OR Neurotol OR Amizepine OR Cariprazine OR "RGH 188" OR Chlordiazepoxide OR Methaminodiazepoxide OR Librium OR Chlozepid OR Elenium OR Chlorpromazine OR Thorazine OR Aminazine OR Largactil OR Chlordelazine OR Contomin OR Fenactil OR Propaphenin OR Chlorazine OR Citalopram OR Cytalopram OR "Lu 10 171" OR Lu10171 OR Escitalopram OR Lexapro OR Clobazam OR "HR 376" OR Onfi OR "LM 2717" OR Frisium OR Urbanyl OR Clomipramine OR Chlomipramine OR Chlorimipramine OR Hydiphen OR Anafranil OR Clonazepam OR "Ro 5 4023" OR "Ro 54023" OR Antelepsin OR Rivotril OR Clopenthixol OR Zuclopenthixol OR Cisordinol OR Clorazepate OR Chlorazepate OR Tranxene OR Tranxilium OR "4306 CB" OR Clozapine OR Clozaril OR Leponex OR Desipramine OR Desmethylimipramine OR Demethylimipramine OR Norpramin OR Pertofrane OR Pertrofran OR Pertofran OR Petylyl OR Desvenlafaxine OR "O Desmethylvenlafaxine" OR "WY 45,233" OR "WY 45,233" OR "WY45,233" OR "WY 45233" OR WY45233 OR Pristiq OR Dextroamphetamine OR Dexamphetamine OR Dexamfetamine OR "Dextro Amphetamine" OR "D Amphetamine" OR Dexedrine OR DextroStat OR Oxydess OR Diazepam OR Diazemuls OR Faustan OR Valium OR Seduxen OR Sibazon OR Stesolid OR Apaurin OR Relanium OR "Valproic Acid" OR Divalproex OR "Propylisopropylacetic Acid" OR "2 Propylpentanoic Acid" OR Convulsofin OR Depakene OR Depakine OR Depakote OR Vupral OR Valproate OR Ergenyl OR "Dipropyl Acetate" OR Domperidone OR Domperidon OR Domidon OR Gastrocure OR Motilium OR Nauzelin OR Peridys OR "R 33,812" OR "R33,812" OR "R 33812" OR R33812 OR Dothiepin OR Dosulepin OR Prothiaden OR Doxepin OR Deptran OR Desidox OR Doneurin OR Doxepia OR Espadox OR Mareen OR Prudoxin OR Quitaxon OR Sinequan OR Sinquan OR Zonalon OR Xepin OR Aponal OR ApoDoxepin OR Droperidol OR Inapsine OR Dehidrobenzperidol OR Dehydrobenzperidol OR Droleptan OR Duloxetine OR "LY 248686" OR LY248686 OR "LY 227942" OR LY227942 OR Cymbalta OR Estazolam OR Tasedan OR ProSom OR "D 40TA" OR D40TA OR Nuctalon OR Eszopiclone OR Lunesta OR Estorra OR Flunitrazepam OR Fluridrazepam OR Flunibeta OR Flunimerck OR Fluninoc OR Rohypnol OR Rohipnol OR Narcozep OR "Flunizep von CT" OR "RO 5 4200" OR RO54200 OR Fluoxetine OR Fluoxetin OR "Lilly 110140" OR Lilly110140 OR Sarafem OR Prozac OR Flupenthixol OR Flupentixol OR Emergil OR Fluanxol OR Fluphenazine OR Flufenazin OR Lyogen OR Prolixin OR Fluvoxamine OR Fluvoxadura OR Fluvoxamin OR Fluvoxamina OR Luvox OR Fevarin OR Floxyfral OR Dumirox OR Faverin OR Desiflu OR "DU 23000" OR DU23000 OR Guanfacine OR Tenex OR Lon798 OR "BS 100 141" OR BS100141 OR Estulic OR Haloperidol OR Haldol OR Iloperidone OR Zomaril OR Fanapt OR "HP 873" OR Imipramine OR Imizin OR Norchlorimipramine OR Imidobenzyle OR Tofranil OR Melipramine OR Pryleugan OR Janimine OR Isocarboxazid OR Lamotrigine OR Crisomet OR Lamictal OR Lamiktal OR "BW 430C" OR Labileno OR Methotrimeprazine OR Levomepromazine OR Levopromazine OR Levomeprazin OR Tisercin OR Tizercine OR Tizertsin OR Lithium OR Dilithium OR Lithane OR Lithobid OR Lithonate OR "CP-15,467 61" OR "CP15,46761" OR Micalith OR "NSC 16895" OR NSC16895 OR Priadel OR "Quilinorm Retard" OR Quilinormretard OR Eskalith OR Lithotabs OR Lorazepam OR Ativan OR Temesta OR "Orfidal Wyeth" OR Donix OR Duralozam OR Durazolam OR Idalprem OR Laubeel OR "Lorazep von CT" OR "Novo Lorazem" OR NovoLorazem OR "Nu Loraz" OR NuLoraz OR Sedicepan OR Sinestron OR Somagerol OR Tolid OR "WY 4036" OR WY4036 OR ApoLorazepam OR Loxapine OR Cloxazepine OR Oxilapine OR Loxitane OR Loxipine OR Loxapinsuccinate OR "CL 71,563" OR "CL71,563" OR Lurasidone OR "SM 13496" OR SM13496 OR "SM-13,496" OR "SM13,496" OR Latuda OR Methylphenidate OR Metadate OR Equasym OR Methylin OR Concerta OR Phenidylate OR Ritalin OR Ritaline OR Tsentedrin OR Centedrin OR Daytrana OR Mianserin OR Tolvon OR Lerivon OR Org GB 94 OR Midazolam OR Dormicum OR Versed OR "Ro 21 3981" OR "Ro 213981" OR Milnacipran OR Midalcipran OR Levomilnacipran OR Savella OR "F 2207" OR Ixel OR Mirtazapine OR "6 Azamianserin" OR Esmirtazapine OR Remeron OR Remergil OR Zispin OR Norset OR Rexer OR "Org 50081" OR " OR G 3770" OR Moclobemide OR Moclobamide OR Arima OR Aurorix OR Manerix OR Moclamine OR Aurorex OR Deprenorm OR Feraken OR Moclobemid OR Moclobeta OR Moclodura OR Moclonorm OR Rimoc OR "Ro 11 1163" OR Modafinil OR Benzhydrylsulfinylacetamide OR "CRL 40476" OR Vigil OR Provigil OR Sparlon OR Alertec OR Modiodal OR Molindone OR Moban OR Nefazodone OR Rulivan OR Serzone OR Dutonin OR Nefadar OR Menfazona OR Nitrazepam OR Nitrodiazepam OR "Dormo Puren" OR Eatan OR Imadorm OR Imeson OR Mogadon OR Nitrazadon OR Nitrazep OR Novanox OR Radedorm OR Remnos OR Serenade OR Somnite OR Alodorm OR Dormalon OR Nortriptyline OR Desmethylamitriptylin OR Desitriptyline OR Aventyl OR Paxtibi OR Allegron OR Norfenazin OR Pamelor OR Nortrilen OR Olanzapine OR Zolafren OR "LY 170052" OR Zyprexa OR "LY 170053" OR Oxazepam OR Serax OR Tazepam OR Adumbran OR Oxcarbazepine OR Timox OR Trileptal OR "GP 47680" OR Paliperidone OR "9 OH Risperidone" OR "9 Hydroxy Risperidone" OR "9 Hydroxyrisperidone" OR Invega OR "R 76477" OR R76477 OR Paroxetine OR "BRL 29060" OR BRL29060 OR "FG 7051" OR FG7051 OR Seroxat OR Paxil OR Aropax OR Periciazine OR Propericiazine OR Pericyazine OR Neuleptil OR Neuleptyl OR Aolept OR Phenelzine OR "Beta Phenylethylhydrazine" OR "2 Phenethylhydrazine" OR Fenelzin OR Phenethylhydrazine OR Nardelzine OR Nardil OR Phenobarbital OR Phenobarbitone OR "Phenylethylbarbituric Acid" OR Phenemal OR Phenylbarbital OR Hysteps OR Luminal OR Gardenal OR Pindolol OR Prindolol OR Visken OR "LB 46" OR LB46 OR Prazepam OR Lysanxia OR Reapam OR Centrax OR Demetrin OR Pregabalin OR "3 Isobutyl GABA" OR Lyrica OR "CI 1008" OR CI1008 OR Prochlorperazine OR Compazine OR Promazine OR Sparine OR Sinophenin OR Protactyl OR Promethazine OR Prometazin OR Proazamine OR Rumergan OR Diprazin OR Phenergan OR Phenargan OR Phensedyl OR Pipolfen OR Pipolphen OR Promet OR Prothazin OR Pyrethia OR Remsed OR Atosil OR Diphergan OR Propranolol OR Propanolol OR Inderal OR Avlocardyl OR "AY 20694" OR AY20694 OR Rexigen OR Dexpropranolol OR Dociton OR Obsidan OR Obzidan OR Anaprilin OR Anapriline OR Betadren OR Protriptyline OR Vivactil OR Quetiapine OR "ICI 204,636" OR "ICI 204636" OR ICI204636 OR Seroquel OR Reboxetine OR Vestra OR Remoxipride OR "FLA 731" OR FLA731 OR Risperidone OR Risperdal OR Risperidal OR "R 64,766" OR "R64,766" OR "R 64766" OR R64766 OR Selegiline OR Selegyline OR "L Deprenyl" OR "E 250" OR E250 OR Eldepryl OR Emsam OR Zelapar OR Deprenil OR Deprenalin OR Yumex OR Jumex OR Humex OR Deprenyl OR Sertindole OR Serlect OR "Lu 23 174" OR Serdolect OR Sertraline OR Zoloft OR Altruline OR Lustral OR Aremis OR Besitran OR Sealdin OR Gladem OR Sulpiride OR Sulperide OR Arminol OR Deponerton OR Meresa OR Desisulpid OR Digton OR Dogmatil OR Dolmatil OR Eglonyl OR Ekilid OR Guastil OR Lebopride OR Neogama OR Pontiride OR Psicocen OR Sulp OR Sulpitil OR Sulpivert OR Sulpor OR Synedil OR Tepavil OR Aiglonyl OR Temazepam OR Hydroxydiazepam OR Methyloxazepam OR Signopam OR Tenox OR "WY 3917" OR WY3917 OR Dasuen OR Euhypnos OR Levanxol OR "Norkotral Tema" OR Normison OR Nocturne OR Temtabs OR Normitab OR Nortem OR Planum OR "Pronervon T" OR Remestan OR Restoril OR "Ro 5 5345" OR Ro55345 OR "SaH 47 603" OR "SaH 47603" OR Temaze OR "Temazep von CT" OR Thioridazine OR ApoThioridazine OR Meleril OR Melleril OR Melleryl OR Mellaril OR Melleretten OR Melzine OR Thiozine OR Sonapax OR Thioridazineneurazpharm OR Aldazine OR Rideril OR Thiothixene OR Tiotixene OR Navane OR Topiramate OR USL255 OR "McN 4853" OR Topamax OR Epitomax OR Tranylcypromine OR "Trans 2 Phenylcyclopropylamine" OR Jatrosom OR Transamine OR Parnate OR Trazodone OR Tradozone OR "AF 1161" OR AF1161 OR Deprax OR Desyrel OR Molipaxin OR Trittico OR Thombran OR "Trazodon Hexal" OR "Trazodon Neuraxpharm" OR Trazon OR Triazolam OR "U 33,030" OR "U33,030" OR Halcion OR Trilam OR "Apo Triazo" OR Trifluoperazine OR Trifluoroperazine OR Trifluperazine OR Eskazine OR Flupazine OR Terfluzine OR Triftazin OR Stelazine OR Trimipramine OR Trimeprimine OR Herphonal OR Trimineurin OR NovoTripramine OR Rhotrimine OR Stangyl OR Surmontil OR Trimidura OR Trimineurin OR Trimipramin OR "Apo Trimip" OR ApoTrimip OR Eldoral OR Venlafaxine OR "Wy 45030" OR Wy45030 OR "Wy 45,030" OR "Wy45,030" OR Effexor OR Trevilor OR Vandral OR Efexor OR Dobupal OR Vigabatrin OR "Gamma Vinyl GABA" OR "Gamma Vinyl Gamma Aminobutyric Acid" OR Sabril OR Sabrilex OR Vortioxetine OR Brintellix OR "Lu AA21004" OR LuAA21004 OR Zaleplon OR "SKP 1041" OR Sonata OR Zelepion OR Starnoc OR "CL 284,846" OR "CL284,846" OR "CL 284846" OR "L 846" OR Ziprasidone OR Ziprazidone OR "CP 88,059" OR "CP 88059" OR Zolpidem OR Amsic OR Bikalm OR Dalparan OR "SL 80.0750" OR "SL 800750 23 N" OR Stilnoct OR Stilnox OR Zodormdura OR Zoldem OR Zolirin OR "Zolpi Lich" OR Zolpinox OR Zolpimist OR Ambien OR Zopiclone OR Zop OR Zopicalma OR Zopiclodura OR Zopiclon OR Zopitan OR Zorclone OR Imovane OR Ximovan OR Zimovane OR Limovan OR Optidorm OR Rhovane OR "RP 27 267" OR Siaten OR Somnosan OR Zileze OR Zimoclone OR "Zopi Puren" OR Zopicalm OR Zotepine OR Zoleptil OR Nipolept OR Zuclopenthixol OR Zuclopentixol OR Clopixol OR Zuclopenthixole OR Acuphase ) ) AND ( ( (MH "Depression") OR (MH "Depression, Reactive") OR (MH "Dysthymic Disorder") OR (MH "Self-Rating Depression Scale") OR (MH "Beck Depression Inventory, Revised Edition") OR (MH "Center for Epidemiological Studies Depression Scale") OR (MH "Hamilton Rating Scale for Depression") OR (MH "Dysthymic Disorder") OR (MH "Cyclothymic Disorder") OR (MH "Affective Disorders") OR (MH "Affective Disorders, Psychotic") ) OR TI ( "Affective Disorder" or "Affective Disorders" or "Affective Psychoses" or "Affective Psychosis" or Cyclothymic or Depress* or Dysthym* or "Mood Disorder" or "Mood Disorders" ) OR AB ( "Affective Disorder" or "Affective Disorders" or "Affective Psychoses" or "Affective Psychosis" or Cyclothymic or Depress* or Dysthym* or "Mood Disorder" or "Mood Disorders" ) ) AND ((TI (systematic* n3 review*)) or (AB (systematic* n3 review*)) or (TI (systematic* n3 bibliographic*)) or (AB (systematic* n3 bibliographic*)) or (TI (systematic* n3 literature)) or (AB (systematic* n3 literature)) or (TI (comprehensive* n3 literature)) or (AB (comprehensive* n3 literature)) or (TI (comprehensive* n3 bibliographic*)) or (AB (comprehensive* n3 bibliographic*)) or (TI (integrative n3 review)) or (AB (integrative n3 review)) or (JN “Cochrane Database of Systematic Reviews”) or (TI (information n2 synthesis)) or (TI (data n2 synthesis)) or (AB (information n2 synthesis)) or (AB (data n2 synthesis)) or (TI (data n2 extract*)) or (AB (data n2 extract*)) or (TI (medline or pubmed or psyclit or cinahl or (psycinfo not “psycinfo database”) or “web of science” or scopus or embase)) or (AB (medline or pubmed or psyclit or cinahl or (psycinfo not “psycinfo database”) or “web of science” or scopus or embase)) or (MH “Systematic Review”) or (MH “Meta Analysis”) or (TI (meta-analy* or metaanaly*)) or (AB (meta-analy* or metaanaly*)))

**B. Cochrane Database of Systematic Reviews**

([mh "Brain Hemorrhage, Traumatic"] OR [mh ^"Brain Injuries"] OR [mh ^"Brain Injury, Chronic"] OR [mh ^"Cerebral Hemorrhage, Traumatic"] OR [mh ^"Cerebrovascular Trauma"] OR [mh ^"Craniocerebral Trauma"] OR [mh ^"Diffuse Axonal Injury"] OR [mh "Head Injuries, Closed"] OR [mh ^"Head Injuries, Penetrating"] OR [mh "Intracranial Hemorrhage, Traumatic"] OR [mh Pneumocephalus] OR (((Brain OR Cerebr* OR Crani* OR "Crushing Skull" OR "Diffuse Axonal" OR Head OR Hemisphere*) NEAR/1 (Injur* OR Trauma*)) OR ((Cerebr* OR Crani* OR Head) NEAR/1 (Lesion* OR Wound*)) OR ((Posttraumatic OR Traumatic) NEAR/1 Encephalopath*) OR (Traumatic NEAR/1 (Brain OR Cerebr*)) OR Concuss* OR DAI OR DAIs OR Pneumocephalus OR TBI OR TBIs):ti,ab) AND ([mh "Adrenergic Beta-Antagonists"] or [mh "Anti-Anxiety Agents"] or [mh Anticonvulsants] or [mh "Antidepressive Agents"] or [mh "Antipsychotic Agents"] or [mh "Atomoxetine Hydrochloride"] or [mh Benzodiazepines] or [mh "Clorazepate Dipotassium"] or [mh "Desvenlafaxine Succinate"] or [mh "Duloxetine Hydrochloride"] or [mh "Lithium Carbonate"] or [mh "Lurasidone Hydrochloride"] or [mh "Paliperidone Palmitate"] or [mh "Quetiapine Fumarate"] or [mh "Venlafaxine Hydrochloride"] or [mh Alprazolam] or [mh Amitriptyline] or [mh Amoxapine] or [mh Aripiprazole] or [mh Atenolol] or [mh Benztropine] or [mh Bromazepam] or [mh Bupropion] or [mh Buspirone] or [mh Carbamazepine] or [mh Chlordiazepoxide] or [mh Chlorpromazine] or [mh Citalopram] or [mh Clomipramine] or [mh Clonazepam] or [mh Clopenthixol] or [mh Clozapine] or [mh Desipramine] or [mh Dextroamphetamine] or [mh Diazepam] or [mh Domperidone] or [mh Dothiepin] or [mh Doxepin] or [mh Droperidol] or [mh Estazolam] or [mh Eszopiclone] or [mh Flunitrazepam] or [mh Fluoxetine] or [mh Flupenthixol] or [mh Fluphenazine] or [mh Fluvoxamine] or [mh Guanfacine] or [mh Haloperidol] or [mh Imipramine] or [mh Isocarboxazid] or [mh Lithium] or [mh Lorazepam] or [mh Loxapine] or [mh Methotrimeprazine] or [mh Methylphenidate] or [mh Mianserin] or [mh Midazolam] or [mh Moclobemide] or [mh Molindone] or [mh Nitrazepam] or [mh Nortriptyline] or [mh Oxazepam] or [mh Paroxetine] or [mh Phenelzine] or [mh Phenobarbital] or [mh Pindolol] or [mh Prazepam] or [mh Pregabalin] or [mh Prochlorperazine] or [mh Promazine] or [mh Promethazine] or [mh Propranolol] or [mh Protriptyline] or [mh Remoxipride] or [mh Risperidone] or [mh Selegiline] or [mh Sertraline] or [mh Sulpiride] or [mh Temazepam] or [mh Thioridazine] or [mh Thiothixene] or [mh Tranylcypromine] or [mh Trazodone] or [mh Triazolam] or [mh Trifluoperazine] or [mh Trimipramine] or [mh "Valproic Acid"] or [mh Vigabatrin] OR ((Adrenergic NEAR/1 Beta NEAR/2 (Antagonist OR Block*)) OR Anti Anxiety OR Anti Convuls* OR Anti Depress* OR Anti Epilep* OR Anti Psychotic* OR Antianxiety OR Anticonvuls* OR Antidepress* OR Antiepilep* OR Antipsychotic* OR Anxiolytic* OR Benzodiazepine* OR (Beta NEAR/1 Block*) OR (Beta NEAR/1 Adrenergic NEAR/2 Block*) OR Thymoanaleptic* OR Thymoleptic* OR Agomelatine OR "S 20098" OR S20098 OR Thymanax OR Valdoxan OR "AGO 178" OR AGO178 OR Alprazolam OR Alprazolan OR "Apo Alpraz" OR ApoAlpraz OR Cassadan OR "D 65MT" OR D65MT OR Xanax OR Tafil OR Trankimazin OR "Novo Alprazol" OR NovoAlprazol OR "Nu Alpraz" OR NuAlpraz OR Ralozam OR "U-31,889" OR "U31,889" OR Alprox OR Esparon OR Kalma OR Amisulpride OR Sultopride OR Barnetil OR "DAN 2163" OR Solian OR "LIN 1418" OR Amitriptyline OR Amineurin OR Amitrip OR Amitriptylin OR Amitrol OR Tryptine OR ApoAmitriptyline OR Damilen OR Domical OR Laroxyl OR Endep OR Lentizol OR Novoprotect OR Saroten OR Sarotex OR Syneudon OR Triptafen OR Tryptizol OR Tryptanol OR Elavil OR Anapsique OR Amoxapine OR Desmethylloxapine OR "CL 67,772" OR "CL67,772" OR Demolox OR Asendin OR Defanyl OR Asendis OR Aripiprazole OR Aripiprazol OR "OPC 14597" OR Abilify OR Asenapine OR Saphris OR "OR G 5222" OR Atenolol OR Tenormine OR Tenormin OR "ICI 66082" OR ICI66082 OR Atomoxetine OR Tomoxetine OR Strattera OR "LY 139603" OR Benztropine OR Benzatropine OR Bensylate OR PMSBenztropine OR Cogentin OR Cogentinol OR Methylbenztropine OR ApoBenztropine OR Brexpiprazole OR Bromazepam OR BromaLich OR "Bromaz 1A Pharma" OR Bromazanil OR "Bromazep von CT" OR Durazanil OR Lexotan OR Lexotanil OR Lexatin OR Lexomil OR "Ro 5-3350" OR "Ro 53350" OR Anxyrex OR Bupropion OR Amfebutamone OR Zyntabac OR Quomen OR Wellbutrin OR Zyban OR Buspirone OR "MJ 9022 1" OR MJ90221 OR Neurosine OR Busp OR Anxut OR Buspar OR Bespar OR Carbamazepine OR Tegretol OR Carbazepin OR Epitol OR Finlepsin OR Neurotol OR Amizepine OR Cariprazine OR "RGH 188" OR Chlordiazepoxide OR Methaminodiazepoxide OR Librium OR Chlozepid OR Elenium OR Chlorpromazine OR Thorazine OR Aminazine OR Largactil OR Chlordelazine OR Contomin OR Fenactil OR Propaphenin OR Chlorazine OR Citalopram OR Cytalopram OR "Lu 10 171" OR Lu10171 OR Escitalopram OR Lexapro OR Clobazam OR "HR 376" OR Onfi OR "LM 2717" OR Frisium OR Urbanyl OR Clomipramine OR Chlomipramine OR Chlorimipramine OR Hydiphen OR Anafranil OR Clonazepam OR "Ro 5 4023" OR "Ro 54023" OR Antelepsin OR Rivotril OR Clopenthixol OR Zuclopenthixol OR Cisordinol OR Clorazepate OR Chlorazepate OR Tranxene OR Tranxilium OR "4306 CB" OR Clozapine OR Clozaril OR Leponex OR Desipramine OR Desmethylimipramine OR Demethylimipramine OR Norpramin OR Pertofrane OR Pertrofran OR Pertofran OR Petylyl OR Desvenlafaxine OR "O Desmethylvenlafaxine" OR "WY 45,233" OR "WY 45,233" OR "WY45,233" OR "WY 45233" OR WY45233 OR Pristiq OR Dextroamphetamine OR Dexamphetamine OR Dexamfetamine OR "Dextro Amphetamine" OR "D Amphetamine" OR Dexedrine OR DextroStat OR Oxydess OR Diazepam OR Diazemuls OR Faustan OR Valium OR Seduxen OR Sibazon OR Stesolid OR Apaurin OR Relanium OR "Valproic Acid" OR Divalproex OR "Propylisopropylacetic Acid" OR "2 Propylpentanoic Acid" OR Convulsofin OR Depakene OR Depakine OR Depakote OR Vupral OR Valproate OR Ergenyl OR "Dipropyl Acetate" OR Domperidone OR Domperidon OR Domidon OR Gastrocure OR Motilium OR Nauzelin OR Peridys OR "R 33,812" OR "R33,812" OR "R 33812" OR R33812 OR Dothiepin OR Dosulepin OR Prothiaden OR Doxepin OR Deptran OR Desidox OR Doneurin OR Doxepia OR Espadox OR Mareen OR Prudoxin OR Quitaxon OR Sinequan OR Sinquan OR Zonalon OR Xepin OR Aponal OR ApoDoxepin OR Droperidol OR Inapsine OR Dehidrobenzperidol OR Dehydrobenzperidol OR Droleptan OR Duloxetine OR "LY 248686" OR LY248686 OR "LY 227942" OR LY227942 OR Cymbalta OR Estazolam OR Tasedan OR ProSom OR "D 40TA" OR D40TA OR Nuctalon OR Eszopiclone OR Lunesta OR Estorra OR Flunitrazepam OR Fluridrazepam OR Flunibeta OR Flunimerck OR Fluninoc OR Rohypnol OR Rohipnol OR Narcozep OR "Flunizep von CT" OR "RO 5 4200" OR RO54200 OR Fluoxetine OR Fluoxetin OR "Lilly 110140" OR Lilly110140 OR Sarafem OR Prozac OR Flupenthixol OR Flupentixol OR Emergil OR Fluanxol OR Fluphenazine OR Flufenazin OR Lyogen OR Prolixin OR Fluvoxamine OR Fluvoxadura OR Fluvoxamin OR Fluvoxamina OR Luvox OR Fevarin OR Floxyfral OR Dumirox OR Faverin OR Desiflu OR "DU 23000" OR DU23000 OR Guanfacine OR Tenex OR Lon798 OR "BS 100 141" OR BS100141 OR Estulic OR Haloperidol OR Haldol OR Iloperidone OR Zomaril OR Fanapt OR "HP 873" OR Imipramine OR Imizin OR Norchlorimipramine OR Imidobenzyle OR Tofranil OR Melipramine OR Pryleugan OR Janimine OR Isocarboxazid OR Lamotrigine OR Crisomet OR Lamictal OR Lamiktal OR "BW 430C" OR Labileno OR Methotrimeprazine OR Levomepromazine OR Levopromazine OR Levomeprazin OR Tisercin OR Tizercine OR Tizertsin OR Lithium OR Dilithium OR Lithane OR Lithobid OR Lithonate OR "CP-15,467 61" OR "CP15,46761" OR Micalith OR "NSC 16895" OR NSC16895 OR Priadel OR "Quilinorm Retard" OR Quilinormretard OR Eskalith OR Lithotabs OR Lorazepam OR Ativan OR Temesta OR "Orfidal Wyeth" OR Donix OR Duralozam OR Durazolam OR Idalprem OR Laubeel OR "Lorazep von CT" OR "Novo Lorazem" OR NovoLorazem OR "Nu Loraz" OR NuLoraz OR Sedicepan OR Sinestron OR Somagerol OR Tolid OR "WY 4036" OR WY4036 OR ApoLorazepam OR Loxapine OR Cloxazepine OR Oxilapine OR Loxitane OR Loxipine OR Loxapinsuccinate OR "CL 71,563" OR "CL71,563" OR Lurasidone OR "SM 13496" OR SM13496 OR "SM-13,496" OR "SM13,496" OR Latuda OR Methylphenidate OR Metadate OR Equasym OR Methylin OR Concerta OR Phenidylate OR Ritalin OR Ritaline OR Tsentedrin OR Centedrin OR Daytrana OR Mianserin OR Tolvon OR Lerivon OR Org GB 94 OR Midazolam OR Dormicum OR Versed OR "Ro 21 3981" OR "Ro 213981" OR Milnacipran OR Midalcipran OR Levomilnacipran OR Savella OR "F 2207" OR Ixel OR Mirtazapine OR "6 Azamianserin" OR Esmirtazapine OR Remeron OR Remergil OR Zispin OR Norset OR Rexer OR "Org 50081" OR " OR G 3770" OR Moclobemide OR Moclobamide OR Arima OR Aurorix OR Manerix OR Moclamine OR Aurorex OR Deprenorm OR Feraken OR Moclobemid OR Moclobeta OR Moclodura OR Moclonorm OR Rimoc OR "Ro 11 1163" OR Modafinil OR Benzhydrylsulfinylacetamide OR "CRL 40476" OR Vigil OR Provigil OR Sparlon OR Alertec OR Modiodal OR Molindone OR Moban OR Nefazodone OR Rulivan OR Serzone OR Dutonin OR Nefadar OR Menfazona OR Nitrazepam OR Nitrodiazepam OR "Dormo Puren" OR Eatan OR Imadorm OR Imeson OR Mogadon OR Nitrazadon OR Nitrazep OR Novanox OR Radedorm OR Remnos OR Serenade OR Somnite OR Alodorm OR Dormalon OR Nortriptyline OR Desmethylamitriptylin OR Desitriptyline OR Aventyl OR Paxtibi OR Allegron OR Norfenazin OR Pamelor OR Nortrilen OR Olanzapine OR Zolafren OR "LY 170052" OR Zyprexa OR "LY 170053" OR Oxazepam OR Serax OR Tazepam OR Adumbran OR Oxcarbazepine OR Timox OR Trileptal OR "GP 47680" OR Paliperidone OR "9 OH Risperidone" OR "9 Hydroxy Risperidone" OR "9 Hydroxyrisperidone" OR Invega OR "R 76477" OR R76477 OR Paroxetine OR "BRL 29060" OR BRL29060 OR "FG 7051" OR FG7051 OR Seroxat OR Paxil OR Aropax OR Periciazine OR Propericiazine OR Pericyazine OR Neuleptil OR Neuleptyl OR Aolept OR Phenelzine OR "Beta Phenylethylhydrazine" OR "2 Phenethylhydrazine" OR Fenelzin OR Phenethylhydrazine OR Nardelzine OR Nardil OR Phenobarbital OR Phenobarbitone OR "Phenylethylbarbituric Acid" OR Phenemal OR Phenylbarbital OR Hysteps OR Luminal OR Gardenal OR Pindolol OR Prindolol OR Visken OR "LB 46" OR LB46 OR Prazepam OR Lysanxia OR Reapam OR Centrax OR Demetrin OR Pregabalin OR "3 Isobutyl GABA" OR Lyrica OR "CI 1008" OR CI1008 OR Prochlorperazine OR Compazine OR Promazine OR Sparine OR Sinophenin OR Protactyl OR Promethazine OR Prometazin OR Proazamine OR Rumergan OR Diprazin OR Phenergan OR Phenargan OR Phensedyl OR Pipolfen OR Pipolphen OR Promet OR Prothazin OR Pyrethia OR Remsed OR Atosil OR Diphergan OR Propranolol OR Propanolol OR Inderal OR Avlocardyl OR "AY 20694" OR AY20694 OR Rexigen OR Dexpropranolol OR Dociton OR Obsidan OR Obzidan OR Anaprilin OR Anapriline OR Betadren OR Protriptyline OR Vivactil OR Quetiapine OR "ICI 204,636" OR "ICI 204636" OR ICI204636 OR Seroquel OR Reboxetine OR Vestra OR Remoxipride OR "FLA 731" OR FLA731 OR Risperidone OR Risperdal OR Risperidal OR "R 64,766" OR "R64,766" OR "R 64766" OR R64766 OR Selegiline OR Selegyline OR "L Deprenyl" OR "E 250" OR E250 OR Eldepryl OR Emsam OR Zelapar OR Deprenil OR Deprenalin OR Yumex OR Jumex OR Humex OR Deprenyl OR Sertindole OR Serlect OR "Lu 23 174" OR Serdolect OR Sertraline OR Zoloft OR Altruline OR Lustral OR Aremis OR Besitran OR Sealdin OR Gladem OR Sulpiride OR Sulperide OR Arminol OR Deponerton OR Meresa OR Desisulpid OR Digton OR Dogmatil OR Dolmatil OR Eglonyl OR Ekilid OR Guastil OR Lebopride OR Neogama OR Pontiride OR Psicocen OR Sulp OR Sulpitil OR Sulpivert OR Sulpor OR Synedil OR Tepavil OR Aiglonyl OR Temazepam OR Hydroxydiazepam OR Methyloxazepam OR Signopam OR Tenox OR "WY 3917" OR WY3917 OR Dasuen OR Euhypnos OR Levanxol OR "Norkotral Tema" OR Normison OR Nocturne OR Temtabs OR Normitab OR Nortem OR Planum OR "Pronervon T" OR Remestan OR Restoril OR "Ro 5 5345" OR Ro55345 OR "SaH 47 603" OR "SaH 47603" OR Temaze OR "Temazep von CT" OR Thioridazine OR ApoThioridazine OR Meleril OR Melleril OR Melleryl OR Mellaril OR Melleretten OR Melzine OR Thiozine OR Sonapax OR Thioridazineneurazpharm OR Aldazine OR Rideril OR Thiothixene OR Tiotixene OR Navane OR Topiramate OR USL255 OR "McN 4853" OR Topamax OR Epitomax OR Tranylcypromine OR "Trans 2 Phenylcyclopropylamine" OR Jatrosom OR Transamine OR Parnate OR Trazodone OR Tradozone OR "AF 1161" OR AF1161 OR Deprax OR Desyrel OR Molipaxin OR Trittico OR Thombran OR "Trazodon Hexal" OR "Trazodon Neuraxpharm" OR Trazon OR Triazolam OR "U 33,030" OR "U33,030" OR Halcion OR Trilam OR "Apo Triazo" OR Trifluoperazine OR Trifluoroperazine OR Trifluperazine OR Eskazine OR Flupazine OR Terfluzine OR Triftazin OR Stelazine OR Trimipramine OR Trimeprimine OR Herphonal OR Trimineurin OR NovoTripramine OR Rhotrimine OR Stangyl OR Surmontil OR Trimidura OR Trimineurin OR Trimipramin OR "Apo Trimip" OR ApoTrimip OR Eldoral OR Venlafaxine OR "Wy 45030" OR Wy45030 OR "Wy 45,030" OR "Wy45,030" OR Effexor OR Trevilor OR Vandral OR Efexor OR Dobupal OR Vigabatrin OR "Gamma Vinyl GABA" OR "Gamma Vinyl Gamma Aminobutyric Acid" OR Sabril OR Sabrilex OR Vortioxetine OR Brintellix OR "Lu AA21004" OR LuAA21004 OR Zaleplon OR "SKP 1041" OR Sonata OR Zelepion OR Starnoc OR "CL 284,846" OR "CL284,846" OR "CL 284846" OR "L 846" OR Ziprasidone OR Ziprazidone OR "CP 88,059" OR "CP 88059" OR Zolpidem OR Amsic OR Bikalm OR Dalparan OR "SL 80.0750" OR "SL 800750 23 N" OR Stilnoct OR Stilnox OR Zodormdura OR Zoldem OR Zolirin OR "Zolpi Lich" OR Zolpinox OR Zolpimist OR Ambien OR Zopiclone OR Zop OR Zopicalma OR Zopiclodura OR Zopiclon OR Zopitan OR Zorclone OR Imovane OR Ximovan OR Zimovane OR Limovan OR Optidorm OR Rhovane OR "RP 27 267" OR Siaten OR Somnosan OR Zileze OR Zimoclone OR "Zopi Puren" OR Zopicalm OR Zotepine OR Zoleptil OR Nipolept OR Zuclopenthixol OR Zuclopentixol OR Clopixol OR Zuclopenthixole OR Acuphase):ti,ab) AND ([mh Depression] or [mh ^"Mood Disorders"] or [mh ^"Depressive Disorder"] or [mh "Depressive Disorder, Major"] or [mh "Dysthymic Disorder"] or [mh "Depressive Disorder, Treatment‐Resistant"] or [mh "Affective Disorders, Psychotic"] or [mh "Cyclothymic Disorder"] or ("Affective Disorder" or "Affective Disorders" or "Affective Psychoses" or "Affective Psychosis" or Cyclothymic or Depress* or Dysthym* or "Mood Disorder" or "Mood Disorders"):ti,ab)

**C. Embase 1980 to 2020 Week 18**

1. Brain Concussion/ OR Brain Injury/ OR Diffuse Axonal Injury/ OR Head Injury/ OR Pneumocephalus/ OR Traumatic Brain Injury/ OR (((Brain OR Cerebr$ OR Crani$ OR Crushing Skull OR Diffuse Axonal OR Head OR Hemisphere?) adj1 (Injur$ OR Trauma$)) OR ((Cerebr$ OR Crani$ OR Head) adj (Lesion? OR Wound?)) OR ((Posttraumatic OR Traumatic) adj Encephalopath$) OR (Traumatic adj (Brain OR Cerebr$)) OR Concuss$ OR DAI OR DAIs OR Pneumocephalus OR TBI OR TBIs).ti,ab.
2. Anxiolytic Agent/ OR Anticonvulsive Agent/ OR Antidepressant Agent/ OR Neuroleptic Agent/ OR Benzodiazepine Derivative/ OR Beta Adrenergic Receptor Blocking Agent/ OR Agomelatine/ OR Alprazolam/ OR Amisulpride/ OR Amitriptyline/ OR Amoxapine/ OR Aripiprazole/ OR Atenolol/ OR Atomoxetine/ OR Benztropine/ OR Brexpiprazole/ OR Bromazepam/ OR Bupropion/ OR Buspirone/ OR Carbamazepine/ OR Cariprazine/ OR Chlordiazepoxide/ OR Chlorpromazine/ OR Citalopram/ OR Clobazam/ OR Clomipramine/ OR Clonazepam/ OR Clopenthixol/ OR Clorazepate/ OR Clozapine/ OR Desipramine/ OR Desvenlafaxine/ OR Dexamphetamine/ OR Diazepam/ OR Domperidone/ OR Dosulepin/ OR Doxepin/ OR Droperidol/ OR Duloxetine/ OR Estazolam/ OR Eszopiclone/ OR Flunitrazepam/ OR Fluoxetine/ OR Flupentixol/ OR Fluphenazine/ OR Fluvoxamine/ OR Guanfacine/ OR Haloperidol/ OR Iloperidone/ OR Imipramine/ OR Isocarboxazid/ OR Lamotrigine/ OR Levomepromazine/ OR Lithium/ OR Lithium Carbonate/ OR Lorazepam/ OR Loxapine/ OR Lurasidone/ OR Methylphenidate/ OR Mianserin/ OR Midazolam/ OR Milnacipran/ OR Mirtazapine/ OR Moclobemide/ OR Modafinil/ OR Molindone/ OR Nefazodone/ OR Nitrazepam/ OR Nortriptyline/ OR Olanzapine/ OR Oxazepam/ OR Oxcarbazepine/ OR Paliperidone/ OR Paroxetine/ OR Periciazine/ OR Phenelzine/ OR Phenobarbital/ OR Pindolol/ OR Prazepam/ OR Pregabalin/ OR Prochlorperazine/ OR Promazine/ OR Promethazine/ OR Propranolol/ OR Protriptyline/ OR Quetiapine/ OR Reboxetine/ OR Remoxipride/ OR Risperidone/ OR Selegiline/ OR Sertindole/ OR Sertraline/ OR Sulpiride/ OR Temazepam/ OR Thioridazine/ OR Tiotixene/ OR Topiramate/ OR Tranylcypromine/ OR Trazodone/ OR Triazolam/ OR Trifluoperazine/ OR Trimipramine/ OR Valproic Acid/ OR Venlafaxine/ OR Vigabatrin/ OR Vortioxetine/ OR Zaleplon/ OR Ziprasidone/ OR Zolpidem/ OR Zopiclone/ OR Zotepine/ OR Zuclopenthixol/ OR Zuclopenthixol Acetate/ OR Zuclopenthixol Decanoate/ OR ((Adrenergic adj Beta adj2 (Antagonist OR Block$)) OR Anti Anxiety OR Anti Convuls$ OR Anti Depress$ OR Anti Epilep$ OR Anti Psychotic? OR Antianxiety OR Anticonvuls$ OR Antidepress$ OR Antiepilep$ OR Antipsychotic$ OR Anxiolytic$ OR Benzodiazepine$ OR (Beta adj Block$) OR (Beta adj1 Adrenergic adj2 Block$) OR Thymoanaleptic$ OR Thymoleptic$ OR Agomelatine OR "S 20098" OR S20098 OR Thymanax OR Valdoxan OR "AGO 178" OR AGO178 OR Alprazolam OR Alprazolan OR "Apo Alpraz" OR ApoAlpraz OR Cassadan OR "D 65MT" OR D65MT OR Xanax OR Tafil OR Trankimazin OR "Novo Alprazol" OR NovoAlprazol OR "Nu Alpraz" OR NuAlpraz OR Ralozam OR "U-31,889" OR "U31,889" OR Alprox OR Esparon OR Kalma OR Amisulpride OR Sultopride OR Barnetil OR "DAN 2163" OR Solian OR "LIN 1418" OR Amitriptyline OR Amineurin OR Amitrip OR Amitriptylin OR Amitrol OR Tryptine OR ApoAmitriptyline OR Damilen OR Domical OR Laroxyl OR Endep OR Lentizol OR Novoprotect OR Saroten OR Sarotex OR Syneudon OR Triptafen OR Tryptizol OR Tryptanol OR Elavil OR Anapsique OR Amoxapine OR Desmethylloxapine OR "CL 67,772" OR "CL67,772" OR Demolox OR Asendin OR Defanyl OR Asendis OR Aripiprazole OR Aripiprazol OR "OPC 14597" OR Abilify OR Asenapine OR Saphris OR "OR G 5222" OR Atenolol OR Tenormine OR Tenormin OR "ICI 66082" OR ICI66082 OR Atomoxetine OR Tomoxetine OR Strattera OR "LY 139603" OR Benztropine OR Benzatropine OR Bensylate OR PMSBenztropine OR Cogentin OR Cogentinol OR Methylbenztropine OR ApoBenztropine OR Brexpiprazole OR Bromazepam OR BromaLich OR "Bromaz 1A Pharma" OR Bromazanil OR "Bromazep von CT" OR Durazanil OR Lexotan OR Lexotanil OR Lexatin OR Lexomil OR "Ro 5-3350" OR "Ro 53350" OR Anxyrex OR Bupropion OR Amfebutamone OR Zyntabac OR Quomen OR Wellbutrin OR Zyban OR Buspirone OR "MJ 9022 1" OR MJ90221 OR Neurosine OR Busp OR Anxut OR Buspar OR Bespar OR Carbamazepine OR Tegretol OR Carbazepin OR Epitol OR Finlepsin OR Neurotol OR Amizepine OR Cariprazine OR "RGH 188" OR Chlordiazepoxide OR Methaminodiazepoxide OR Librium OR Chlozepid OR Elenium OR Chlorpromazine OR Thorazine OR Aminazine OR Largactil OR Chlordelazine OR Contomin OR Fenactil OR Propaphenin OR Chlorazine OR Citalopram OR Cytalopram OR "Lu 10 171" OR Lu10171 OR Escitalopram OR Lexapro OR Clobazam OR "HR 376" OR Onfi OR "LM 2717" OR Frisium OR Urbanyl OR Clomipramine OR Chlomipramine OR Chlorimipramine OR Hydiphen OR Anafranil OR Clonazepam OR "Ro 5 4023" OR "Ro 54023" OR Antelepsin OR Rivotril OR Clopenthixol OR Zuclopenthixol OR Cisordinol OR Clorazepate OR Chlorazepate OR Tranxene OR Tranxilium OR "4306 CB" OR Clozapine OR Clozaril OR Leponex OR Desipramine OR Desmethylimipramine OR Demethylimipramine OR Norpramin OR Pertofrane OR Pertrofran OR Pertofran OR Petylyl OR Desvenlafaxine OR "O Desmethylvenlafaxine" OR "WY 45,233" OR "WY 45,233" OR "WY45,233" OR "WY 45233" OR WY45233 OR Pristiq OR Dextroamphetamine OR Dexamphetamine OR Dexamfetamine OR "Dextro Amphetamine" OR "D Amphetamine" OR Dexedrine OR DextroStat OR Oxydess OR Diazepam OR Diazemuls OR Faustan OR Valium OR Seduxen OR Sibazon OR Stesolid OR Apaurin OR Relanium OR "Valproic Acid" OR Divalproex OR "Propylisopropylacetic Acid" OR "2 Propylpentanoic Acid" OR Convulsofin OR Depakene OR Depakine OR Depakote OR Vupral OR Valproate OR Ergenyl OR "Dipropyl Acetate" OR Domperidone OR Domperidon OR Domidon OR Gastrocure OR Motilium OR Nauzelin OR Peridys OR "R 33,812" OR "R33,812" OR "R 33812" OR R33812 OR Dothiepin OR Dosulepin OR Prothiaden OR Doxepin OR Deptran OR Desidox OR Doneurin OR Doxepia OR Espadox OR Mareen OR Prudoxin OR Quitaxon OR Sinequan OR Sinquan OR Zonalon OR Xepin OR Aponal OR ApoDoxepin OR Droperidol OR Inapsine OR Dehidrobenzperidol OR Dehydrobenzperidol OR Droleptan OR Duloxetine OR "LY 248686" OR LY248686 OR "LY 227942" OR LY227942 OR Cymbalta OR Estazolam OR Tasedan OR ProSom OR "D 40TA" OR D40TA OR Nuctalon OR Eszopiclone OR Lunesta OR Estorra OR Flunitrazepam OR Fluridrazepam OR Flunibeta OR Flunimerck OR Fluninoc OR Rohypnol OR Rohipnol OR Narcozep OR "Flunizep von CT" OR "RO 5 4200" OR RO54200 OR Fluoxetine OR Fluoxetin OR "Lilly 110140" OR Lilly110140 OR Sarafem OR Prozac OR Flupenthixol OR Flupentixol OR Emergil OR Fluanxol OR Fluphenazine OR Flufenazin OR Lyogen OR Prolixin OR Fluvoxamine OR Fluvoxadura OR Fluvoxamin OR Fluvoxamina OR Luvox OR Fevarin OR Floxyfral OR Dumirox OR Faverin OR Desiflu OR "DU 23000" OR DU23000 OR Guanfacine OR Tenex OR Lon798 OR "BS 100 141" OR BS100141 OR Estulic OR Haloperidol OR Haldol OR Iloperidone OR Zomaril OR Fanapt OR "HP 873" OR Imipramine OR Imizin OR Norchlorimipramine OR Imidobenzyle OR Tofranil OR Melipramine OR Pryleugan OR Janimine OR Isocarboxazid OR Lamotrigine OR Crisomet OR Lamictal OR Lamiktal OR "BW 430C" OR Labileno OR Methotrimeprazine OR Levomepromazine OR Levopromazine OR Levomeprazin OR Tisercin OR Tizercine OR Tizertsin OR Lithium OR Dilithium OR Lithane OR Lithobid OR Lithonate OR "CP-15,467 61" OR "CP15,46761" OR Micalith OR "NSC 16895" OR NSC16895 OR Priadel OR "Quilinorm Retard" OR Quilinormretard OR Eskalith OR Lithotabs OR Lorazepam OR Ativan OR Temesta OR "Orfidal Wyeth" OR Donix OR Duralozam OR Durazolam OR Idalprem OR Laubeel OR "Lorazep von CT" OR "Novo Lorazem" OR NovoLorazem OR "Nu Loraz" OR NuLoraz OR Sedicepan OR Sinestron OR Somagerol OR Tolid OR "WY 4036" OR WY4036 OR ApoLorazepam OR Loxapine OR Cloxazepine OR Oxilapine OR Loxitane OR Loxipine OR Loxapinsuccinate OR "CL 71,563" OR "CL71,563" OR Lurasidone OR "SM 13496" OR SM13496 OR "SM-13,496" OR "SM13,496" OR Latuda OR Methylphenidate OR Metadate OR Equasym OR Methylin OR Concerta OR Phenidylate OR Ritalin OR Ritaline OR Tsentedrin OR Centedrin OR Daytrana OR Mianserin OR Tolvon OR Lerivon OR Org GB 94 OR Midazolam OR Dormicum OR Versed OR "Ro 21 3981" OR "Ro 213981" OR Milnacipran OR Midalcipran OR Levomilnacipran OR Savella OR "F 2207" OR Ixel OR Mirtazapine OR "6 Azamianserin" OR Esmirtazapine OR Remeron OR Remergil OR Zispin OR Norset OR Rexer OR "Org 50081" OR " OR G 3770" OR Moclobemide OR Moclobamide OR Arima OR Aurorix OR Manerix OR Moclamine OR Aurorex OR Deprenorm OR Feraken OR Moclobemid OR Moclobeta OR Moclodura OR Moclonorm OR Rimoc OR "Ro 11 1163" OR Modafinil OR Benzhydrylsulfinylacetamide OR "CRL 40476" OR Vigil OR Provigil OR Sparlon OR Alertec OR Modiodal OR Molindone OR Moban OR Nefazodone OR Rulivan OR Serzone OR Dutonin OR Nefadar OR Menfazona OR Nitrazepam OR Nitrodiazepam OR "Dormo Puren" OR Eatan OR Imadorm OR Imeson OR Mogadon OR Nitrazadon OR Nitrazep OR Novanox OR Radedorm OR Remnos OR Serenade OR Somnite OR Alodorm OR Dormalon OR Nortriptyline OR Desmethylamitriptylin OR Desitriptyline OR Aventyl OR Paxtibi OR Allegron OR Norfenazin OR Pamelor OR Nortrilen OR Olanzapine OR Zolafren OR "LY 170052" OR Zyprexa OR "LY 170053" OR Oxazepam OR Serax OR Tazepam OR Adumbran OR Oxcarbazepine OR Timox OR Trileptal OR "GP 47680" OR Paliperidone OR "9 OH Risperidone" OR "9 Hydroxy Risperidone" OR "9 Hydroxyrisperidone" OR Invega OR "R 76477" OR R76477 OR Paroxetine OR "BRL 29060" OR BRL29060 OR "FG 7051" OR FG7051 OR Seroxat OR Paxil OR Aropax OR Periciazine OR Propericiazine OR Pericyazine OR Neuleptil OR Neuleptyl OR Aolept OR Phenelzine OR "Beta Phenylethylhydrazine" OR "2 Phenethylhydrazine" OR Fenelzin OR Phenethylhydrazine OR Nardelzine OR Nardil OR Phenobarbital OR Phenobarbitone OR "Phenylethylbarbituric Acid" OR Phenemal OR Phenylbarbital OR Hysteps OR Luminal OR Gardenal OR Pindolol OR Prindolol OR Visken OR "LB 46" OR LB46 OR Prazepam OR Lysanxia OR Reapam OR Centrax OR Demetrin OR Pregabalin OR "3 Isobutyl GABA" OR Lyrica OR "CI 1008" OR CI1008 OR Prochlorperazine OR Compazine OR Promazine OR Sparine OR Sinophenin OR Protactyl OR Promethazine OR Prometazin OR Proazamine OR Rumergan OR Diprazin OR Phenergan OR Phenargan OR Phensedyl OR Pipolfen OR Pipolphen OR Promet OR Prothazin OR Pyrethia OR Remsed OR Atosil OR Diphergan OR Propranolol OR Propanolol OR Inderal OR Avlocardyl OR "AY 20694" OR AY20694 OR Rexigen OR Dexpropranolol OR Dociton OR Obsidan OR Obzidan OR Anaprilin OR Anapriline OR Betadren OR Protriptyline OR Vivactil OR Quetiapine OR "ICI 204,636" OR "ICI 204636" OR ICI204636 OR Seroquel OR Reboxetine OR Vestra OR Remoxipride OR "FLA 731" OR FLA731 OR Risperidone OR Risperdal OR Risperidal OR "R 64,766" OR "R64,766" OR "R 64766" OR R64766 OR Selegiline OR Selegyline OR "L Deprenyl" OR "E 250" OR E250 OR Eldepryl OR Emsam OR Zelapar OR Deprenil OR Deprenalin OR Yumex OR Jumex OR Humex OR Deprenyl OR Sertindole OR Serlect OR "Lu 23 174" OR Serdolect OR Sertraline OR Zoloft OR Altruline OR Lustral OR Aremis OR Besitran OR Sealdin OR Gladem OR Sulpiride OR Sulperide OR Arminol OR Deponerton OR Meresa OR Desisulpid OR Digton OR Dogmatil OR Dolmatil OR Eglonyl OR Ekilid OR Guastil OR Lebopride OR Neogama OR Pontiride OR Psicocen OR Sulp OR Sulpitil OR Sulpivert OR Sulpor OR Synedil OR Tepavil OR Aiglonyl OR Temazepam OR Hydroxydiazepam OR Methyloxazepam OR Signopam OR Tenox OR "WY 3917" OR WY3917 OR Dasuen OR Euhypnos OR Levanxol OR "Norkotral Tema" OR Normison OR Nocturne OR Temtabs OR Normitab OR Nortem OR Planum OR "Pronervon T" OR Remestan OR Restoril OR "Ro 5 5345" OR Ro55345 OR "SaH 47 603" OR "SaH 47603" OR Temaze OR "Temazep von CT" OR Thioridazine OR ApoThioridazine OR Meleril OR Melleril OR Melleryl OR Mellaril OR Melleretten OR Melzine OR Thiozine OR Sonapax OR Thioridazineneurazpharm OR Aldazine OR Rideril OR Thiothixene OR Tiotixene OR Navane OR Topiramate OR USL255 OR "McN 4853" OR Topamax OR Epitomax OR Tranylcypromine OR "Trans 2 Phenylcyclopropylamine" OR Jatrosom OR Transamine OR Parnate OR Trazodone OR Tradozone OR "AF 1161" OR AF1161 OR Deprax OR Desyrel OR Molipaxin OR Trittico OR Thombran OR "Trazodon Hexal" OR "Trazodon Neuraxpharm" OR Trazon OR Triazolam OR "U 33,030" OR "U33,030" OR Halcion OR Trilam OR "Apo Triazo" OR Trifluoperazine OR Trifluoroperazine OR Trifluperazine OR Eskazine OR Flupazine OR Terfluzine OR Triftazin OR Stelazine OR Trimipramine OR Trimeprimine OR Herphonal OR Trimineurin OR NovoTripramine OR Rhotrimine OR Stangyl OR Surmontil OR Trimidura OR Trimineurin OR Trimipramin OR "Apo Trimip" OR ApoTrimip OR Eldoral OR Venlafaxine OR "Wy 45030" OR Wy45030 OR "Wy 45,030" OR "Wy45,030" OR Effexor OR Trevilor OR Vandral OR Efexor OR Dobupal OR Vigabatrin OR "Gamma Vinyl GABA" OR "Gamma Vinyl Gamma Aminobutyric Acid" OR Sabril OR Sabrilex OR Vortioxetine OR Brintellix OR "Lu AA21004" OR LuAA21004 OR Zaleplon OR "SKP 1041" OR Sonata OR Zelepion OR Starnoc OR "CL 284,846" OR "CL284,846" OR "CL 284846" OR "L 846" OR Ziprasidone OR Ziprazidone OR "CP 88,059" OR "CP 88059" OR Zolpidem OR Amsic OR Bikalm OR Dalparan OR "SL 80.0750" OR "SL 800750 23 N" OR Stilnoct OR Stilnox OR Zodormdura OR Zoldem OR Zolirin OR "Zolpi Lich" OR Zolpinox OR Zolpimist OR Ambien OR Zopiclone OR Zop OR Zopicalma OR Zopiclodura OR Zopiclon OR Zopitan OR Zorclone OR Imovane OR Ximovan OR Zimovane OR Limovan OR Optidorm OR Rhovane OR "RP 27 267" OR Siaten OR Somnosan OR Zileze OR Zimoclone OR "Zopi Puren" OR Zopicalm OR Zotepine OR Zoleptil OR Nipolept OR Zuclopenthixol OR Zuclopentixol OR Clopixol OR Zuclopenthixole OR Acuphase).ti,ab.
3. Depression/ or Adolescent Depression/ or Agitated Depression/ or Atypical Depression/ or Chronic Depression/ or Depressive Psychosis/ or Dysthymia/ or Major Depression/ or Minor Depression/ or "Mixed Anxiety and Depression"/ or "Mixed Depression and Dementia"/ or "Mixed Mania and Depression"/ or Reactive Depression/ or Recurrent Brief Depression/ or Subsyndromal Depression/ or Treatment Resistant Depression/ or Cyclothymia/ or Exp Depression Assessment/ or Mood Disorder/ or Affective Psychosis/ or Major Affective Disorder/ or Minor Affective Disorder/ or ("Affective Disorder" or "Affective Disorders" or "Affective Psychoses" or "Affective Psychosis" or Cyclothymic or Depress* or Dysthym* or "Mood Disorder" or "Mood Disorders").ti,ab.
4. meta-analysis.pt. or meta-analysis/ or systematic review/ or meta-analysis as topic/ or "meta analysis (topic)"/ or "systematic review (topic)"/ or exp technology assessment, biomedical/ or (((systematic* adj3 (review* or overview*)) or (methodologic* adj3 (review* or overview*))) or ((quantitative adj3 (review* or overview* or synthes*)) or (research adj3 (integrati* or overview*))) or ((integrative adj3 (review* or overview*)) or (collaborative adj3 (review* or overview*)) or (pool* adj3 analy*)) or (data synthes* or data extraction* or data abstraction*) or (handsearch* or hand search*) or (mantel haenszel or peto or der simonian or dersimonian or fixed effect* or latin square*) or (met analy* or metanaly* or technology assessment* or HTA or HTAs or technology overview* or technology appraisal*) or (meta regression* or metaregression*) or (comparative adj3 (efficacy or effectiveness)) or (outcomes research or relative effectiveness) or ((indirect or indirect treatment or mixed-treatment) adj comparison*)).ti,ab,kw. or (meta-analy* or metaanaly* or systematic review* or biomedical technology assessment* or bio-medical technology assessment*).mp,hw. or (medline or cochrane or pubmed or medlars or embase or cinahl).ti,ab,hw. Or (cochrane or (health adj2 technology assessment) or evidence report).jw.
5. 1 AND 2 AND 3 AND 4

**D. Epistemonikos**

title:((Brain OR Cerebr* OR Cranial OR Cranio* OR "Diffuse Axonal" OR Head OR Hemisphere* OR Trauma* OR Posttrauma* OR Concuss*) AND (Affective OR Cyclothymic OR Depression OR Depressive OR Dysthymic OR Mood))

Publication type: Systematic Review

**E. Ovid MEDLINE(R) ALL 1946 to May 01, 2020**

1. Exp Brain Hemorrhage, Traumatic/ OR Brain Injuries/ OR Brain Injury, Chronic/ OR Cerebral Hemorrhage, Traumatic/ OR Cerebrovascular Trauma/ OR Craniocerebral Trauma/ OR Diffuse Axonal Injury/ OR Exp Head Injuries, Closed/ OR Head Injuries, Penetrating/ OR Exp Intracranial Hemorrhage, Traumatic/ OR Exp Pneumocephalus/ OR (((Brain OR Cerebr$ OR Crani$ OR Crushing Skull OR Diffuse Axonal OR Head OR Hemisphere?) adj1 (Injur$ OR Trauma$)) OR ((Cerebr$ OR Crani$ OR Head) adj (Lesion? OR Wound?)) OR ((Posttraumatic OR Traumatic) adj Encephalopath$) OR (Traumatic adj (Brain OR Cerebr$)) OR Concuss$ OR DAI OR DAIs OR Pneumocephalus OR TBI OR TBIs).ti,ab.
2. "Anti-Anxiety Agents"/ OR "Anticonvulsants"/ OR "Antidepressive Agents"/ OR "Antipsychotic Agents"/ OR "Benzodiazepines"/ OR "Adrenergic Beta-Antagonists"/ OR Alprazolam/ OR Amitriptyline/ OR Amoxapine/ OR Aripiprazole/ OR Atenolol/ OR Atomoxetine Hydrochloride/ OR Benztropine/ OR Bromazepam/ OR Bupropion/ OR Buspirone/ OR Carbamazepine/ OR Chlordiazepoxide/ OR Chlorpromazine/ OR Citalopram/ OR Clomipramine/ OR Clonazepam/ OR Clopenthixol/ OR Clorazepate Dipotassium/ OR Clozapine/ OR Desipramine/ OR Desvenlafaxine Succinate/ OR Dextroamphetamine/ OR Diazepam/ OR Valproic Acid/ OR Domperidone/ OR Dothiepin/ OR Doxepin/ OR Droperidol/ OR Duloxetine Hydrochloride/ OR Estazolam/ OR Eszopiclone/ OR Flunitrazepam/ OR Fluoxetine/ OR Flupenthixol/ OR Fluphenazine/ OR Fluvoxamine/ OR Guanfacine/ OR Haloperidol/ OR Imipramine/ OR Isocarboxazid/ OR Methotrimeprazine/ OR Lithium/ OR Lithium Carbonate/ OR Lorazepam/ OR Loxapine/ OR Lurasidone Hydrochloride/ OR Methylphenidate/ OR Mianserin/ OR Midazolam/ OR Moclobemide/ OR Molindone/ OR Nitrazepam/ OR Nortriptyline/ OR Oxazepam/ OR Paliperidone Palmitate/ OR Paroxetine/ OR Phenelzine/ OR Phenobarbital/ OR Pindolol/ OR Prazepam/ OR Pregabalin/ OR Prochlorperazine/ OR Promazine/ OR Promethazine/ OR Propranolol/ OR Protriptyline/ OR Quetiapine Fumarate/ OR Remoxipride/ OR Risperidone/ OR Selegiline/ OR Sertraline/ OR Sulpiride/ OR Temazepam/ OR Thioridazine/ OR Thiothixene/ OR Tranylcypromine/ OR Trazodone/ OR Triazolam/ OR Trifluoperazine/ OR Trimipramine/ OR Venlafaxine Hydrochloride/ OR Vigabatrin/ OR ((Adrenergic adj Beta adj2 (Antagonist OR Block$)) OR Anti Anxiety OR Anti Convuls$ OR Anti Depress$ OR Anti Epilep$ OR Anti Psychotic? OR Antianxiety OR Anticonvuls$ OR Antidepress$ OR Antiepilep$ OR Antipsychotic$ OR Anxiolytic$ OR Benzodiazepine$ OR (Beta adj Block$) OR (Beta adj1 Adrenergic adj2 Block$) OR Thymoanaleptic$ OR Thymoleptic$ OR Agomelatine OR "S 20098" OR S20098 OR Thymanax OR Valdoxan OR "AGO 178" OR AGO178 OR Alprazolam OR Alprazolan OR "Apo Alpraz" OR ApoAlpraz OR Cassadan OR "D 65MT" OR D65MT OR Xanax OR Tafil OR Trankimazin OR "Novo Alprazol" OR NovoAlprazol OR "Nu Alpraz" OR NuAlpraz OR Ralozam OR "U-31,889" OR "U31,889" OR Alprox OR Esparon OR Kalma OR Amisulpride OR Sultopride OR Barnetil OR "DAN 2163" OR Solian OR "LIN 1418" OR Amitriptyline OR Amineurin OR Amitrip OR Amitriptylin OR Amitrol OR Tryptine OR ApoAmitriptyline OR Damilen OR Domical OR Laroxyl OR Endep OR Lentizol OR Novoprotect OR Saroten OR Sarotex OR Syneudon OR Triptafen OR Tryptizol OR Tryptanol OR Elavil OR Anapsique OR Amoxapine OR Desmethylloxapine OR "CL 67,772" OR "CL67,772" OR Demolox OR Asendin OR Defanyl OR Asendis OR Aripiprazole OR Aripiprazol OR "OPC 14597" OR Abilify OR Asenapine OR Saphris OR "OR G 5222" OR Atenolol OR Tenormine OR Tenormin OR "ICI 66082" OR ICI66082 OR Atomoxetine OR Tomoxetine OR Strattera OR "LY 139603" OR Benztropine OR Benzatropine OR Bensylate OR PMSBenztropine OR Cogentin OR Cogentinol OR Methylbenztropine OR ApoBenztropine OR Brexpiprazole OR Bromazepam OR BromaLich OR "Bromaz 1A Pharma" OR Bromazanil OR "Bromazep von CT" OR Durazanil OR Lexotan OR Lexotanil OR Lexatin OR Lexomil OR "Ro 5-3350" OR "Ro 53350" OR Anxyrex OR Bupropion OR Amfebutamone OR Zyntabac OR Quomen OR Wellbutrin OR Zyban OR Buspirone OR "MJ 9022 1" OR MJ90221 OR Neurosine OR Busp OR Anxut OR Buspar OR Bespar OR Carbamazepine OR Tegretol OR Carbazepin OR Epitol OR Finlepsin OR Neurotol OR Amizepine OR Cariprazine OR "RGH 188" OR Chlordiazepoxide OR Methaminodiazepoxide OR Librium OR Chlozepid OR Elenium OR Chlorpromazine OR Thorazine OR Aminazine OR Largactil OR Chlordelazine OR Contomin OR Fenactil OR Propaphenin OR Chlorazine OR Citalopram OR Cytalopram OR "Lu 10 171" OR Lu10171 OR Escitalopram OR Lexapro OR Clobazam OR "HR 376" OR Onfi OR "LM 2717" OR Frisium OR Urbanyl OR Clomipramine OR Chlomipramine OR Chlorimipramine OR Hydiphen OR Anafranil OR Clonazepam OR "Ro 5 4023" OR "Ro 54023" OR Antelepsin OR Rivotril OR Clopenthixol OR Zuclopenthixol OR Cisordinol OR Clorazepate OR Chlorazepate OR Tranxene OR Tranxilium OR "4306 CB" OR Clozapine OR Clozaril OR Leponex OR Desipramine OR Desmethylimipramine OR Demethylimipramine OR Norpramin OR Pertofrane OR Pertrofran OR Pertofran OR Petylyl OR Desvenlafaxine OR "O Desmethylvenlafaxine" OR "WY 45,233" OR "WY 45,233" OR "WY45,233" OR "WY 45233" OR WY45233 OR Pristiq OR Dextroamphetamine OR Dexamphetamine OR Dexamfetamine OR "Dextro Amphetamine" OR "D Amphetamine" OR Dexedrine OR DextroStat OR Oxydess OR Diazepam OR Diazemuls OR Faustan OR Valium OR Seduxen OR Sibazon OR Stesolid OR Apaurin OR Relanium OR "Valproic Acid" OR Divalproex OR "Propylisopropylacetic Acid" OR "2 Propylpentanoic Acid" OR Convulsofin OR Depakene OR Depakine OR Depakote OR Vupral OR Valproate OR Ergenyl OR "Dipropyl Acetate" OR Domperidone OR Domperidon OR Domidon OR Gastrocure OR Motilium OR Nauzelin OR Peridys OR "R 33,812" OR "R33,812" OR "R 33812" OR R33812 OR Dothiepin OR Dosulepin OR Prothiaden OR Doxepin OR Deptran OR Desidox OR Doneurin OR Doxepia OR Espadox OR Mareen OR Prudoxin OR Quitaxon OR Sinequan OR Sinquan OR Zonalon OR Xepin OR Aponal OR ApoDoxepin OR Droperidol OR Inapsine OR Dehidrobenzperidol OR Dehydrobenzperidol OR Droleptan OR Duloxetine OR "LY 248686" OR LY248686 OR "LY 227942" OR LY227942 OR Cymbalta OR Estazolam OR Tasedan OR ProSom OR "D 40TA" OR D40TA OR Nuctalon OR Eszopiclone OR Lunesta OR Estorra OR Flunitrazepam OR Fluridrazepam OR Flunibeta OR Flunimerck OR Fluninoc OR Rohypnol OR Rohipnol OR Narcozep OR "Flunizep von CT" OR "RO 5 4200" OR RO54200 OR Fluoxetine OR Fluoxetin OR "Lilly 110140" OR Lilly110140 OR Sarafem OR Prozac OR Flupenthixol OR Flupentixol OR Emergil OR Fluanxol OR Fluphenazine OR Flufenazin OR Lyogen OR Prolixin OR Fluvoxamine OR Fluvoxadura OR Fluvoxamin OR Fluvoxamina OR Luvox OR Fevarin OR Floxyfral OR Dumirox OR Faverin OR Desiflu OR "DU 23000" OR DU23000 OR Guanfacine OR Tenex OR Lon798 OR "BS 100 141" OR BS100141 OR Estulic OR Haloperidol OR Haldol OR Iloperidone OR Zomaril OR Fanapt OR "HP 873" OR Imipramine OR Imizin OR Norchlorimipramine OR Imidobenzyle OR Tofranil OR Melipramine OR Pryleugan OR Janimine OR Isocarboxazid OR Lamotrigine OR Crisomet OR Lamictal OR Lamiktal OR "BW 430C" OR Labileno OR Methotrimeprazine OR Levomepromazine OR Levopromazine OR Levomeprazin OR Tisercin OR Tizercine OR Tizertsin OR Lithium OR Dilithium OR Lithane OR Lithobid OR Lithonate OR "CP-15,467 61" OR "CP15,46761" OR Micalith OR "NSC 16895" OR NSC16895 OR Priadel OR "Quilinorm Retard" OR Quilinormretard OR Eskalith OR Lithotabs OR Lorazepam OR Ativan OR Temesta OR "Orfidal Wyeth" OR Donix OR Duralozam OR Durazolam OR Idalprem OR Laubeel OR "Lorazep von CT" OR "Novo Lorazem" OR NovoLorazem OR "Nu Loraz" OR NuLoraz OR Sedicepan OR Sinestron OR Somagerol OR Tolid OR "WY 4036" OR WY4036 OR ApoLorazepam OR Loxapine OR Cloxazepine OR Oxilapine OR Loxitane OR Loxipine OR Loxapinsuccinate OR "CL 71,563" OR "CL71,563" OR Lurasidone OR "SM 13496" OR SM13496 OR "SM-13,496" OR "SM13,496" OR Latuda OR Methylphenidate OR Metadate OR Equasym OR Methylin OR Concerta OR Phenidylate OR Ritalin OR Ritaline OR Tsentedrin OR Centedrin OR Daytrana OR Mianserin OR Tolvon OR Lerivon OR "Org GB 94" OR Midazolam OR Dormicum OR Versed OR "Ro 21 3981" OR "Ro 213981" OR Milnacipran OR Midalcipran OR Levomilnacipran OR Savella OR "F 2207" OR Ixel OR Mirtazapine OR "6 Azamianserin" OR Esmirtazapine OR Remeron OR Remergil OR Zispin OR Norset OR Rexer OR "Org 50081" OR " OR G 3770" OR Moclobemide OR Moclobamide OR Arima OR Aurorix OR Manerix OR Moclamine OR Aurorex OR Deprenorm OR Feraken OR Moclobemid OR Moclobeta OR Moclodura OR Moclonorm OR Rimoc OR "Ro 11 1163" OR Modafinil OR Benzhydrylsulfinylacetamide OR "CRL 40476" OR Vigil OR Provigil OR Sparlon OR Alertec OR Modiodal OR Molindone OR Moban OR Nefazodone OR Rulivan OR Serzone OR Dutonin OR Nefadar OR Menfazona OR Nitrazepam OR Nitrodiazepam OR "Dormo Puren" OR Eatan OR Imadorm OR Imeson OR Mogadon OR Nitrazadon OR Nitrazep OR Novanox OR Radedorm OR Remnos OR Serenade OR Somnite OR Alodorm OR Dormalon OR Nortriptyline OR Desmethylamitriptylin OR Desitriptyline OR Aventyl OR Paxtibi OR Allegron OR Norfenazin OR Pamelor OR Nortrilen OR Olanzapine OR Zolafren OR "LY 170052" OR Zyprexa OR "LY 170053" OR Oxazepam OR Serax OR Tazepam OR Adumbran OR Oxcarbazepine OR Timox OR Trileptal OR "GP 47680" OR Paliperidone OR "9 OH Risperidone" OR "9 Hydroxy Risperidone" OR "9 Hydroxyrisperidone" OR Invega OR "R 76477" OR R76477 OR Paroxetine OR "BRL 29060" OR BRL29060 OR "FG 7051" OR FG7051 OR Seroxat OR Paxil OR Aropax OR Periciazine OR Propericiazine OR Pericyazine OR Neuleptil OR Neuleptyl OR Aolept OR Phenelzine OR "Beta Phenylethylhydrazine" OR "2 Phenethylhydrazine" OR Fenelzin OR Phenethylhydrazine OR Nardelzine OR Nardil OR Phenobarbital OR Phenobarbitone OR "Phenylethylbarbituric Acid" OR Phenemal OR Phenylbarbital OR Hysteps OR Luminal OR Gardenal OR Pindolol OR Prindolol OR Visken OR "LB 46" OR LB46 OR Prazepam OR Lysanxia OR Reapam OR Centrax OR Demetrin OR Pregabalin OR "3 Isobutyl GABA" OR Lyrica OR "CI 1008" OR CI1008 OR Prochlorperazine OR Compazine OR Promazine OR Sparine OR Sinophenin OR Protactyl OR Promethazine OR Prometazin OR Proazamine OR Rumergan OR Diprazin OR Phenergan OR Phenargan OR Phensedyl OR Pipolfen OR Pipolphen OR Promet OR Prothazin OR Pyrethia OR Remsed OR Atosil OR Diphergan OR Propranolol OR Propanolol OR Inderal OR Avlocardyl OR "AY 20694" OR AY20694 OR Rexigen OR Dexpropranolol OR Dociton OR Obsidan OR Obzidan OR Anaprilin OR Anapriline OR Betadren OR Protriptyline OR Vivactil OR Quetiapine OR "ICI 204,636" OR "ICI 204636" OR ICI204636 OR Seroquel OR Reboxetine OR Vestra OR Remoxipride OR "FLA 731" OR FLA731 OR Risperidone OR Risperdal OR Risperidal OR "R 64,766" OR "R64,766" OR "R 64766" OR R64766 OR Selegiline OR Selegyline OR "L Deprenyl" OR "E 250" OR E250 OR Eldepryl OR Emsam OR Zelapar OR Deprenil OR Deprenalin OR Yumex OR Jumex OR Humex OR Deprenyl OR Sertindole OR Serlect OR "Lu 23 174" OR Serdolect OR Sertraline OR Zoloft OR Altruline OR Lustral OR Aremis OR Besitran OR Sealdin OR Gladem OR Sulpiride OR Sulperide OR Arminol OR Deponerton OR Meresa OR Desisulpid OR Digton OR Dogmatil OR Dolmatil OR Eglonyl OR Ekilid OR Guastil OR Lebopride OR Neogama OR Pontiride OR Psicocen OR Sulp OR Sulpitil OR Sulpivert OR Sulpor OR Synedil OR Tepavil OR Aiglonyl OR Temazepam OR Hydroxydiazepam OR Methyloxazepam OR Signopam OR Tenox OR "WY 3917" OR WY3917 OR Dasuen OR Euhypnos OR Levanxol OR "Norkotral Tema" OR Normison OR Nocturne OR Temtabs OR Normitab OR Nortem OR Planum OR "Pronervon T" OR Remestan OR Restoril OR "Ro 5 5345" OR Ro55345 OR "SaH 47 603" OR "SaH 47603" OR Temaze OR "Temazep von CT" OR Thioridazine OR ApoThioridazine OR Meleril OR Melleril OR Melleryl OR Mellaril OR Melleretten OR Melzine OR Thiozine OR Sonapax OR Thioridazineneurazpharm OR Aldazine OR Rideril OR Thiothixene OR Tiotixene OR Navane OR Topiramate OR USL255 OR "McN 4853" OR Topamax OR Epitomax OR Tranylcypromine OR "Trans 2 Phenylcyclopropylamine" OR Jatrosom OR Transamine OR Parnate OR Trazodone OR Tradozone OR "AF 1161" OR AF1161 OR Deprax OR Desyrel OR Molipaxin OR Trittico OR Thombran OR "Trazodon Hexal" OR "Trazodon Neuraxpharm" OR Trazon OR Triazolam OR "U 33,030" OR "U33,030" OR Halcion OR Trilam OR "Apo Triazo" OR Trifluoperazine OR Trifluoroperazine OR Trifluperazine OR Eskazine OR Flupazine OR Terfluzine OR Triftazin OR Stelazine OR Trimipramine OR Trimeprimine OR Herphonal OR Trimineurin OR NovoTripramine OR Rhotrimine OR Stangyl OR Surmontil OR Trimidura OR Trimineurin OR Trimipramin OR "Apo Trimip" OR ApoTrimip OR Eldoral OR Venlafaxine OR "Wy 45030" OR Wy45030 OR "Wy 45,030" OR "Wy45,030" OR Effexor OR Trevilor OR Vandral OR Efexor OR Dobupal OR Vigabatrin OR "Gamma Vinyl GABA" OR "Gamma Vinyl Gamma Aminobutyric Acid" OR Sabril OR Sabrilex OR Vortioxetine OR Brintellix OR "Lu AA21004" OR LuAA21004 OR Zaleplon OR "SKP 1041" OR Sonata OR Zelepion OR Starnoc OR "CL 284,846" OR "CL284,846" OR "CL 284846" OR "L 846" OR Ziprasidone OR Ziprazidone OR "CP 88,059" OR "CP 88059" OR Zolpidem OR Amsic OR Bikalm OR Dalparan OR "SL 80.0750" OR "SL 800750 23 N" OR Stilnoct OR Stilnox OR Zodormdura OR Zoldem OR Zolirin OR "Zolpi Lich" OR Zolpinox OR Zolpimist OR Ambien OR Zopiclone OR Zop OR Zopicalma OR Zopiclodura OR Zopiclon OR Zopitan OR Zorclone OR Imovane OR Ximovan OR Zimovane OR Limovan OR Optidorm OR Rhovane OR "RP 27 267" OR Siaten OR Somnosan OR Zileze OR Zimoclone OR "Zopi Puren" OR Zopicalm OR Zotepine OR Zoleptil OR Nipolept OR Zuclopenthixol OR Zuclopentixol OR Clopixol OR Zuclopenthixole OR Acuphase).ti,ab.
3. Depression/ or Mood Disorders/ or Depressive Disorder/ or "Depressive Disorder, Major"/ or Dysthymic Disorder/ or "Depressive Disorder, Treatment‐Resistant"/ or "Affective Disorders, Psychotic"/ or Cyclothymic Disorder/ or ("Affective Disorder" or "Affective Disorders" or "Affective Psychoses" or "Affective Psychosis" or Cyclothymic or Depress* or Dysthym* or "Mood Disorder" or "Mood Disorders").ti,ab.
4. meta-analysis.pt. or meta-analysis/ or systematic review/ or meta-analysis as topic/ or "meta analysis (topic)"/ or "systematic review (topic)"/ or exp technology assessment, biomedical/ or (((systematic* adj3 (review* or overview*)) or (methodologic* adj3 (review* or overview*))) or ((quantitative adj3 (review* or overview* or synthes*)) or (research adj3 (integrati* or overview*))) or ((integrative adj3 (review* or overview*)) or (collaborative adj3 (review* or overview*)) or (pool* adj3 analy*)) or (data synthes* or data extraction* or data abstraction*) or (handsearch* or hand search*) or (mantel haenszel or peto or der simonian or dersimonian or fixed effect* or latin square*) or (met analy* or metanaly* or technology assessment* or HTA or HTAs or technology overview* or technology appraisal*) or (meta regression* or metaregression*) or (comparative adj3 (efficacy or effectiveness)) or (outcomes research or relative effectiveness) or ((indirect or indirect treatment or mixed-treatment) adj comparison*)).ti,ab,kf,kw. or (meta-analy* or metaanaly* or systematic review* or biomedical technology assessment* or bio-medical technology assessment*).mp,hw. or (medline or cochrane or pubmed or medlars or embase or cinahl).ti,ab,hw. Or (cochrane or (health adj2 technology assessment) or evidence report).jw.
5. 1 AND 2 AND 3 AND 4

**F. PROSPERO**

Search 1: Trauma Depression (5 Results)

Search 2: Trauma Depressive (1 Result)

Search 3: Traumatic Depression (1 Result)

Search 4: Traumatic Depressive (1 Result)

Search 5: Posttrauma Depression (0 Results)

Search 6: Posttrauma Depressive (0 Results)

Search 7: Posttraumatic Depression (1 Result)

Search 8: Posttraumatic Depressive (0 Result)

Search 9: TBI Depression (2 Results)

Search 10: TBI Depressive (0 Result)

**G. APA PsycInfo 1806 to April Week 4 2020**

1. Exp Head Injuries/ OR Exp Traumatic Brain Injury/ OR (((Brain OR Cerebr$ OR Crani$ OR Crushing Skull OR Diffuse Axonal OR Head OR Hemisphere?) adj1 (Injur$ OR Trauma$)) OR ((Cerebr$ OR Crani$ OR Head) adj (Lesion? OR Wound?)) OR ((Posttraumatic OR Traumatic) adj Encephalopath$) OR (Traumatic adj (Brain OR Cerebr$)) OR Concuss$ OR DAI OR DAIs OR Pneumocephalus OR TBI OR TBIs).ti,ab.
2. Tranquilizing Drugs/ OR Anticonvulsive Drugs/ OR Antidepressant Drugs/ OR Neuroleptic Drugs/ OR Benzodiazepines/ OR Adrenergic Blocking Drugs/ OR Alprazolam/ OR Amitriptyline/ OR Aripiprazole/ OR Atomoxetine/ OR Bupropion/ OR Buspirone/ OR Carbamazepine/ OR Chlordiazepoxide/ OR Chlorpromazine/ OR Citalopram/ OR Chlorimipramine/ OR Clonazepam/ OR Clozapine/ OR Desipramine/ OR Dextroamphetamine/ OR Diazepam/ OR Doxepin/ OR Flunitrazepam/ OR Fluoxetine/ OR Fluphenazine/ OR Fluvoxamine/ OR Haloperidol/ OR Imipramine/ OR Isocarboxazid/ OR Lithium/ OR Lithium Carbonate/ OR Lorazepam/ OR Loxapine/ OR Methylphenidate/ OR Mianserin/ OR Midazolam/ OR Moclobemide/ OR Molindone/ OR Nitrazepam/ OR Nortriptyline/ OR Olanzapine/ OR Oxazepam/ OR Paroxetine/ OR Phenelzine/ OR Phenobarbital/ OR Pregabalin/ OR Prochlorperazine/ OR Promazine/ OR Promethazine/ OR Propranolol/ OR Quetiapine/ OR Risperidone/ OR Sertraline/ OR Sulpiride/ OR Thioridazine/ OR Thiothixene/ OR Tranylcypromine/ OR Trazodone/ OR Triazolam/ OR Trifluoperazine/ OR Valproic Acid/ OR Venlafaxine/ OR ((Adrenergic adj Beta adj2 (Antagonist OR Block$)) OR Anti Anxiety OR Anti Convuls$ OR Anti Depress$ OR Anti Epilep$ OR Anti Psychotic? OR Antianxiety OR Anticonvuls$ OR Antidepress$ OR Antiepilep$ OR Antipsychotic$ OR Anxiolytic$ OR Benzodiazepine$ OR (Beta adj Block$) OR (Beta adj1 Adrenergic adj2 Block$) OR Thymoanaleptic$ OR Thymoleptic$ OR Agomelatine OR "S 20098" OR S20098 OR Thymanax OR Valdoxan OR "AGO 178" OR AGO178 OR Alprazolam OR Alprazolan OR "Apo Alpraz" OR ApoAlpraz OR Cassadan OR "D 65MT" OR D65MT OR Xanax OR Tafil OR Trankimazin OR "Novo Alprazol" OR NovoAlprazol OR "Nu Alpraz" OR NuAlpraz OR Ralozam OR "U-31,889" OR "U31,889" OR Alprox OR Esparon OR Kalma OR Amisulpride OR Sultopride OR Barnetil OR "DAN 2163" OR Solian OR "LIN 1418" OR Amitriptyline OR Amineurin OR Amitrip OR Amitriptylin OR Amitrol OR Tryptine OR ApoAmitriptyline OR Damilen OR Domical OR Laroxyl OR Endep OR Lentizol OR Novoprotect OR Saroten OR Sarotex OR Syneudon OR Triptafen OR Tryptizol OR Tryptanol OR Elavil OR Anapsique OR Amoxapine OR Desmethylloxapine OR "CL 67,772" OR "CL67,772" OR Demolox OR Asendin OR Defanyl OR Asendis OR Aripiprazole OR Aripiprazol OR "OPC 14597" OR Abilify OR Asenapine OR Saphris OR "OR G 5222" OR Atenolol OR Tenormine OR Tenormin OR "ICI 66082" OR ICI66082 OR Atomoxetine OR Tomoxetine OR Strattera OR "LY 139603" OR Benztropine OR Benzatropine OR Bensylate OR PMSBenztropine OR Cogentin OR Cogentinol OR Methylbenztropine OR ApoBenztropine OR Brexpiprazole OR Bromazepam OR BromaLich OR "Bromaz 1A Pharma" OR Bromazanil OR "Bromazep von CT" OR Durazanil OR Lexotan OR Lexotanil OR Lexatin OR Lexomil OR "Ro 5-3350" OR "Ro 53350" OR Anxyrex OR Bupropion OR Amfebutamone OR Zyntabac OR Quomen OR Wellbutrin OR Zyban OR Buspirone OR "MJ 9022 1" OR MJ90221 OR Neurosine OR Busp OR Anxut OR Buspar OR Bespar OR Carbamazepine OR Tegretol OR Carbazepin OR Epitol OR Finlepsin OR Neurotol OR Amizepine OR Cariprazine OR "RGH 188" OR Chlordiazepoxide OR Methaminodiazepoxide OR Librium OR Chlozepid OR Elenium OR Chlorpromazine OR Thorazine OR Aminazine OR Largactil OR Chlordelazine OR Contomin OR Fenactil OR Propaphenin OR Chlorazine OR Citalopram OR Cytalopram OR "Lu 10 171" OR Lu10171 OR Escitalopram OR Lexapro OR Clobazam OR "HR 376" OR Onfi OR "LM 2717" OR Frisium OR Urbanyl OR Clomipramine OR Chlomipramine OR Chlorimipramine OR Hydiphen OR Anafranil OR Clonazepam OR "Ro 5 4023" OR "Ro 54023" OR Antelepsin OR Rivotril OR Clopenthixol OR Zuclopenthixol OR Cisordinol OR Clorazepate OR Chlorazepate OR Tranxene OR Tranxilium OR "4306 CB" OR Clozapine OR Clozaril OR Leponex OR Desipramine OR Desmethylimipramine OR Demethylimipramine OR Norpramin OR Pertofrane OR Pertrofran OR Pertofran OR Petylyl OR Desvenlafaxine OR "O Desmethylvenlafaxine" OR "WY 45,233" OR "WY 45,233" OR "WY45,233" OR "WY 45233" OR WY45233 OR Pristiq OR Dextroamphetamine OR Dexamphetamine OR Dexamfetamine OR "Dextro Amphetamine" OR "D Amphetamine" OR Dexedrine OR DextroStat OR Oxydess OR Diazepam OR Diazemuls OR Faustan OR Valium OR Seduxen OR Sibazon OR Stesolid OR Apaurin OR Relanium OR "Valproic Acid" OR Divalproex OR "Propylisopropylacetic Acid" OR "2 Propylpentanoic Acid" OR Convulsofin OR Depakene OR Depakine OR Depakote OR Vupral OR Valproate OR Ergenyl OR "Dipropyl Acetate" OR Domperidone OR Domperidon OR Domidon OR Gastrocure OR Motilium OR Nauzelin OR Peridys OR "R 33,812" OR "R33,812" OR "R 33812" OR R33812 OR Dothiepin OR Dosulepin OR Prothiaden OR Doxepin OR Deptran OR Desidox OR Doneurin OR Doxepia OR Espadox OR Mareen OR Prudoxin OR Quitaxon OR Sinequan OR Sinquan OR Zonalon OR Xepin OR Aponal OR ApoDoxepin OR Droperidol OR Inapsine OR Dehidrobenzperidol OR Dehydrobenzperidol OR Droleptan OR Duloxetine OR "LY 248686" OR LY248686 OR "LY 227942" OR LY227942 OR Cymbalta OR Estazolam OR Tasedan OR ProSom OR "D 40TA" OR D40TA OR Nuctalon OR Eszopiclone OR Lunesta OR Estorra OR Flunitrazepam OR Fluridrazepam OR Flunibeta OR Flunimerck OR Fluninoc OR Rohypnol OR Rohipnol OR Narcozep OR "Flunizep von CT" OR "RO 5 4200" OR RO54200 OR Fluoxetine OR Fluoxetin OR "Lilly 110140" OR Lilly110140 OR Sarafem OR Prozac OR Flupenthixol OR Flupentixol OR Emergil OR Fluanxol OR Fluphenazine OR Flufenazin OR Lyogen OR Prolixin OR Fluvoxamine OR Fluvoxadura OR Fluvoxamin OR Fluvoxamina OR Luvox OR Fevarin OR Floxyfral OR Dumirox OR Faverin OR Desiflu OR "DU 23000" OR DU23000 OR Guanfacine OR Tenex OR Lon798 OR "BS 100 141" OR BS100141 OR Estulic OR Haloperidol OR Haldol OR Iloperidone OR Zomaril OR Fanapt OR "HP 873" OR Imipramine OR Imizin OR Norchlorimipramine OR Imidobenzyle OR Tofranil OR Melipramine OR Pryleugan OR Janimine OR Isocarboxazid OR Lamotrigine OR Crisomet OR Lamictal OR Lamiktal OR "BW 430C" OR Labileno OR Methotrimeprazine OR Levomepromazine OR Levopromazine OR Levomeprazin OR Tisercin OR Tizercine OR Tizertsin OR Lithium OR Dilithium OR Lithane OR Lithobid OR Lithonate OR "CP-15,467 61" OR "CP15,46761" OR Micalith OR "NSC 16895" OR NSC16895 OR Priadel OR "Quilinorm Retard" OR Quilinormretard OR Eskalith OR Lithotabs OR Lorazepam OR Ativan OR Temesta OR "Orfidal Wyeth" OR Donix OR Duralozam OR Durazolam OR Idalprem OR Laubeel OR "Lorazep von CT" OR "Novo Lorazem" OR NovoLorazem OR "Nu Loraz" OR NuLoraz OR Sedicepan OR Sinestron OR Somagerol OR Tolid OR "WY 4036" OR WY4036 OR ApoLorazepam OR Loxapine OR Cloxazepine OR Oxilapine OR Loxitane OR Loxipine OR Loxapinsuccinate OR "CL 71,563" OR "CL71,563" OR Lurasidone OR "SM 13496" OR SM13496 OR "SM-13,496" OR "SM13,496" OR Latuda OR Methylphenidate OR Metadate OR Equasym OR Methylin OR Concerta OR Phenidylate OR Ritalin OR Ritaline OR Tsentedrin OR Centedrin OR Daytrana OR Mianserin OR Tolvon OR Lerivon OR Org GB 94 OR Midazolam OR Dormicum OR Versed OR "Ro 21 3981" OR "Ro 213981" OR Milnacipran OR Midalcipran OR Levomilnacipran OR Savella OR "F 2207" OR Ixel OR Mirtazapine OR "6 Azamianserin" OR Esmirtazapine OR Remeron OR Remergil OR Zispin OR Norset OR Rexer OR "Org 50081" OR " OR G 3770" OR Moclobemide OR Moclobamide OR Arima OR Aurorix OR Manerix OR Moclamine OR Aurorex OR Deprenorm OR Feraken OR Moclobemid OR Moclobeta OR Moclodura OR Moclonorm OR Rimoc OR "Ro 11 1163" OR Modafinil OR Benzhydrylsulfinylacetamide OR "CRL 40476" OR Vigil OR Provigil OR Sparlon OR Alertec OR Modiodal OR Molindone OR Moban OR Nefazodone OR Rulivan OR Serzone OR Dutonin OR Nefadar OR Menfazona OR Nitrazepam OR Nitrodiazepam OR "Dormo Puren" OR Eatan OR Imadorm OR Imeson OR Mogadon OR Nitrazadon OR Nitrazep OR Novanox OR Radedorm OR Remnos OR Serenade OR Somnite OR Alodorm OR Dormalon OR Nortriptyline OR Desmethylamitriptylin OR Desitriptyline OR Aventyl OR Paxtibi OR Allegron OR Norfenazin OR Pamelor OR Nortrilen OR Olanzapine OR Zolafren OR "LY 170052" OR Zyprexa OR "LY 170053" OR Oxazepam OR Serax OR Tazepam OR Adumbran OR Oxcarbazepine OR Timox OR Trileptal OR "GP 47680" OR Paliperidone OR "9 OH Risperidone" OR "9 Hydroxy Risperidone" OR "9 Hydroxyrisperidone" OR Invega OR "R 76477" OR R76477 OR Paroxetine OR "BRL 29060" OR BRL29060 OR "FG 7051" OR FG7051 OR Seroxat OR Paxil OR Aropax OR Periciazine OR Propericiazine OR Pericyazine OR Neuleptil OR Neuleptyl OR Aolept OR Phenelzine OR "Beta Phenylethylhydrazine" OR "2 Phenethylhydrazine" OR Fenelzin OR Phenethylhydrazine OR Nardelzine OR Nardil OR Phenobarbital OR Phenobarbitone OR "Phenylethylbarbituric Acid" OR Phenemal OR Phenylbarbital OR Hysteps OR Luminal OR Gardenal OR Pindolol OR Prindolol OR Visken OR "LB 46" OR LB46 OR Prazepam OR Lysanxia OR Reapam OR Centrax OR Demetrin OR Pregabalin OR "3 Isobutyl GABA" OR Lyrica OR "CI 1008" OR CI1008 OR Prochlorperazine OR Compazine OR Promazine OR Sparine OR Sinophenin OR Protactyl OR Promethazine OR Prometazin OR Proazamine OR Rumergan OR Diprazin OR Phenergan OR Phenargan OR Phensedyl OR Pipolfen OR Pipolphen OR Promet OR Prothazin OR Pyrethia OR Remsed OR Atosil OR Diphergan OR Propranolol OR Propanolol OR Inderal OR Avlocardyl OR "AY 20694" OR AY20694 OR Rexigen OR Dexpropranolol OR Dociton OR Obsidan OR Obzidan OR Anaprilin OR Anapriline OR Betadren OR Protriptyline OR Vivactil OR Quetiapine OR "ICI 204,636" OR "ICI 204636" OR ICI204636 OR Seroquel OR Reboxetine OR Vestra OR Remoxipride OR "FLA 731" OR FLA731 OR Risperidone OR Risperdal OR Risperidal OR "R 64,766" OR "R64,766" OR "R 64766" OR R64766 OR Selegiline OR Selegyline OR "L Deprenyl" OR "E 250" OR E250 OR Eldepryl OR Emsam OR Zelapar OR Deprenil OR Deprenalin OR Yumex OR Jumex OR Humex OR Deprenyl OR Sertindole OR Serlect OR "Lu 23 174" OR Serdolect OR Sertraline OR Zoloft OR Altruline OR Lustral OR Aremis OR Besitran OR Sealdin OR Gladem OR Sulpiride OR Sulperide OR Arminol OR Deponerton OR Meresa OR Desisulpid OR Digton OR Dogmatil OR Dolmatil OR Eglonyl OR Ekilid OR Guastil OR Lebopride OR Neogama OR Pontiride OR Psicocen OR Sulp OR Sulpitil OR Sulpivert OR Sulpor OR Synedil OR Tepavil OR Aiglonyl OR Temazepam OR Hydroxydiazepam OR Methyloxazepam OR Signopam OR Tenox OR "WY 3917" OR WY3917 OR Dasuen OR Euhypnos OR Levanxol OR "Norkotral Tema" OR Normison OR Nocturne OR Temtabs OR Normitab OR Nortem OR Planum OR "Pronervon T" OR Remestan OR Restoril OR "Ro 5 5345" OR Ro55345 OR "SaH 47 603" OR "SaH 47603" OR Temaze OR "Temazep von CT" OR Thioridazine OR ApoThioridazine OR Meleril OR Melleril OR Melleryl OR Mellaril OR Melleretten OR Melzine OR Thiozine OR Sonapax OR Thioridazineneurazpharm OR Aldazine OR Rideril OR Thiothixene OR Tiotixene OR Navane OR Topiramate OR USL255 OR "McN 4853" OR Topamax OR Epitomax OR Tranylcypromine OR "Trans 2 Phenylcyclopropylamine" OR Jatrosom OR Transamine OR Parnate OR Trazodone OR Tradozone OR "AF 1161" OR AF1161 OR Deprax OR Desyrel OR Molipaxin OR Trittico OR Thombran OR "Trazodon Hexal" OR "Trazodon Neuraxpharm" OR Trazon OR Triazolam OR "U 33,030" OR "U33,030" OR Halcion OR Trilam OR "Apo Triazo" OR Trifluoperazine OR Trifluoroperazine OR Trifluperazine OR Eskazine OR Flupazine OR Terfluzine OR Triftazin OR Stelazine OR Trimipramine OR Trimeprimine OR Herphonal OR Trimineurin OR NovoTripramine OR Rhotrimine OR Stangyl OR Surmontil OR Trimidura OR Trimineurin OR Trimipramin OR "Apo Trimip" OR ApoTrimip OR Eldoral OR Venlafaxine OR "Wy 45030" OR Wy45030 OR "Wy 45,030" OR "Wy45,030" OR Effexor OR Trevilor OR Vandral OR Efexor OR Dobupal OR Vigabatrin OR "Gamma Vinyl GABA" OR "Gamma Vinyl Gamma Aminobutyric Acid" OR Sabril OR Sabrilex OR Vortioxetine OR Brintellix OR "Lu AA21004" OR LuAA21004 OR Zaleplon OR "SKP 1041" OR Sonata OR Zelepion OR Starnoc OR "CL 284,846" OR "CL284,846" OR "CL 284846" OR "L 846" OR Ziprasidone OR Ziprazidone OR "CP 88,059" OR "CP 88059" OR Zolpidem OR Amsic OR Bikalm OR Dalparan OR "SL 80.0750" OR "SL 800750 23 N" OR Stilnoct OR Stilnox OR Zodormdura OR Zoldem OR Zolirin OR "Zolpi Lich" OR Zolpinox OR Zolpimist OR Ambien OR Zopiclone OR Zop OR Zopicalma OR Zopiclodura OR Zopiclon OR Zopitan OR Zorclone OR Imovane OR Ximovan OR Zimovane OR Limovan OR Optidorm OR Rhovane OR "RP 27 267" OR Siaten OR Somnosan OR Zileze OR Zimoclone OR "Zopi Puren" OR Zopicalm OR Zotepine OR Zoleptil OR Nipolept OR Zuclopenthixol OR Zuclopentixol OR Clopixol OR Zuclopenthixole OR Acuphase).ti,ab.
3. "Depression (Emotion)"/ or Major Depression/ or Dysthymic Disorder/ or Reactive Depression/ or Recurrent Depression/ or Treatment Resistant Depression/ or Atypical Depression/ or Beck Depression Inventory/ or Zungs Self Rating Depression Scale/ or Affective Disorders/ or Affective Psychosis/ or Cyclothymic Disorder/ or ("Affective Disorder" or "Affective Disorders" or "Affective Psychoses" or "Affective Psychosis" or Cyclothymic or Depress* or Dysthym* or "Mood Disorder" or "Mood Disorders").ti,ab.
4. meta-analysis.pt. or meta-analysis/ or systematic review/ or meta-analysis as topic/ or "meta analysis (topic)"/ or "systematic review (topic)"/ or (((systematic* adj3 (review* or overview*)) or (methodologic* adj3 (review* or overview*))) or ((quantitative adj3 (review* or overview* or synthes*)) or (research adj3 (integrati* or overview*))) or ((integrative adj3 (review* or overview*)) or (collaborative adj3 (review* or overview*)) or (pool* adj3 analy*)) or (data synthes* or data extraction* or data abstraction*) or (handsearch* or hand search*) or (mantel haenszel or peto or der simonian or dersimonian or fixed effect* or latin square*) or (met analy* or metanaly* or technology assessment* or HTA or HTAs or technology overview* or technology appraisal*) or (meta regression* or metaregression*) or (comparative adj3 (efficacy or effectiveness)) or (outcomes research or relative effectiveness) or ((indirect or indirect treatment or mixed-treatment) adj comparison*)).ti,ab. or (meta-analy* or metaanaly* or systematic review* or biomedical technology assessment* or bio-medical technology assessment*).mp,hw. or (medline or cochrane or pubmed or medlars or embase or cinahl).ti,ab,hw. Or (cochrane or (health adj2 technology assessment) or evidence report).jw. or (meta-analysis or systematic review).md.
5. 1 AND 2 AND 3 AND 4

**Full Search Strategy for the Systematic Review**

**A. CINAHL**

Advanced Search

Limitters - Exclude MEDLINE records

( ( (MH "Brain Injuries") OR (MH "Brain Concussion") OR (MH "Left Hemisphere Injuries") OR (MH "Right Hemisphere Injuries") OR (MH "Pneumocephalus") OR (MH "Head Injuries") ) OR TI ( ((Brain OR Cerebr* OR Crani* OR "Crushing Skull" OR "Diffuse Axonal" OR Head OR Hemisphere*) N1 (Injur* OR Trauma*)) OR ((Cerebr* OR Crani* OR Head) N1 (Lesion* OR Wound*)) OR ((Posttraumatic OR Traumatic) N1 (Encephalopath*)) OR ((Traumatic) N1 (Brain OR Cerebr*)) OR Concuss* OR DAI OR DAIs OR Pneumocephalus OR TBI OR TBIs ) OR AB ( ((Brain OR Cerebr* OR Crani* OR "Crushing Skull" OR "Diffuse Axonal" OR Head OR Hemisphere*) N1 (Injur* OR Trauma*)) OR ((Cerebr* OR Crani* OR Head) N1 (Lesion* OR Wound*)) OR ((Posttraumatic OR Traumatic) N1 (Encephalopath*)) OR ((Traumatic) N1 (Brain OR Cerebr*)) OR Concuss* OR DAI OR DAIs OR Pneumocephalus OR TBI OR TBIs ) ) AND ( ( [MH "Adrenergic Beta-Antagonists"] OR [MH "Antianxiety Agents"] OR [MH Anticonvulsants] OR [MH "Antidepressive Agents"] OR [MH "Antipsychotic Agents"] OR [MH Atomoxetine] OR [MH "Antianxiety Agents, Benzodiazepine"] OR [MH "Clorazepate Dipotassium"] OR [MH "Desvenlafaxine Succinate"] OR [MH "Duloxetine Hydrochloride"] OR [MH "Lithium Carbonate"] OR [MH Paliperidone] OR [MH Quetiapine] OR [MH Venlafaxine] OR [MH Alprazolam] OR [MH Amitriptyline] OR [MH Amoxapine] OR [MH Aripiprazole] OR [MH Atenolol] OR [MH Benztropine] OR [MH Bupropion] OR [MH Buspirone] OR [MH Carbamazepine] OR [MH Chlordiazepoxide] OR [MH Chlorpromazine] OR [MH Citalopram] OR [MH Clomipramine] OR [MH Clonazepam] OR [MH Clozapine] OR [MH Desipramine] OR [MH Dextroamphetamine] OR [MH Diazepam] OR [MH Doxepin] OR [MH Droperidol] OR [MH Estazolam] OR [MH Eszopiclone] OR [MH Flunitrazepam] OR [MH Fluoxetine] OR [MH Fluphenazine] OR [MH "Fluvoxamine Maleate"] OR [MH Haloperidol] OR [MH Imipramine] OR [MH Lithium] OR [MH Lorazepam] OR [MH Loxapine] OR [MH Methylphenidate] OR [MH Midazolam] OR [MH "Molindone Hydrochloride"] OR [MH Nortriptyline] OR [MH Oxazepam] OR [MH Paroxetine] OR [MH Phenobarbital] OR [MH Pindolol] OR [MH Pregabalin] OR [MH Prochlorperazine] OR [MH Promethazine] OR [MH Propranolol] OR [MH Risperidone] OR [MH Selegiline] OR [MH "Sertraline Hydrochloride"] OR [MH Temazepam] OR [MH Thioridazine] OR [MH Thiothixene] OR [MH Trazodone] OR [MH Triazolam] OR [MH "Trifluoperazine Hydrochloride"] OR [MH "Valproic Acid"] ) OR TI ( ((Adrenergic) N1 Beta N2 (Antagonist OR Block*)) OR Anti Anxiety OR Anti Convuls* OR Anti Depress* OR Anti Epilep* OR Anti Psychotic* OR Antianxiety OR Anticonvuls* OR Antidepress* OR Antiepilep* OR Antipsychotic* OR Anxiolytic* OR Benzodiazepine* OR (Beta N1 Block*) OR (Beta N1 Adrenergic N2 Block*) OR Thymoanaleptic* OR Thymoleptic* OR Agomelatine OR "S 20098" OR S20098 OR Thymanax OR Valdoxan OR "AGO 178" OR AGO178 OR Alprazolam OR Alprazolan OR "Apo Alpraz" OR ApoAlpraz OR Cassadan OR "D 65MT" OR D65MT OR Xanax OR Tafil OR Trankimazin OR "Novo Alprazol" OR NovoAlprazol OR "Nu Alpraz" OR NuAlpraz OR Ralozam OR "U-31,889" OR "U31,889" OR Alprox OR Esparon OR Kalma OR Amisulpride OR Sultopride OR Barnetil OR "DAN 2163" OR Solian OR "LIN 1418" OR Amitriptyline OR Amineurin OR Amitrip OR Amitriptylin OR Amitrol OR Tryptine OR ApoAmitriptyline OR Damilen OR Domical OR Laroxyl OR Endep OR Lentizol OR Novoprotect OR Saroten OR Sarotex OR Syneudon OR Triptafen OR Tryptizol OR Tryptanol OR Elavil OR Anapsique OR Amoxapine OR Desmethylloxapine OR "CL 67,772" OR "CL67,772" OR Demolox OR Asendin OR Defanyl OR Asendis OR Aripiprazole OR Aripiprazol OR "OPC 14597" OR Abilify OR Asenapine OR Saphris OR "OR G 5222" OR Atenolol OR Tenormine OR Tenormin OR "ICI 66082" OR ICI66082 OR Atomoxetine OR Tomoxetine OR Strattera OR "LY 139603" OR Benztropine OR Benzatropine OR Bensylate OR PMSBenztropine OR Cogentin OR Cogentinol OR Methylbenztropine OR ApoBenztropine OR Brexpiprazole OR Bromazepam OR BromaLich OR "Bromaz 1A Pharma" OR Bromazanil OR "Bromazep von CT" OR Durazanil OR Lexotan OR Lexotanil OR Lexatin OR Lexomil OR "Ro 5-3350" OR "Ro 53350" OR Anxyrex OR Bupropion OR Amfebutamone OR Zyntabac OR Quomen OR Wellbutrin OR Zyban OR Buspirone OR "MJ 9022 1" OR MJ90221 OR Neurosine OR Busp OR Anxut OR Buspar OR Bespar OR Carbamazepine OR Tegretol OR Carbazepin OR Epitol OR Finlepsin OR Neurotol OR Amizepine OR Cariprazine OR "RGH 188" OR Chlordiazepoxide OR Methaminodiazepoxide OR Librium OR Chlozepid OR Elenium OR Chlorpromazine OR Thorazine OR Aminazine OR Largactil OR Chlordelazine OR Contomin OR Fenactil OR Propaphenin OR Chlorazine OR Citalopram OR Cytalopram OR "Lu 10 171" OR Lu10171 OR Escitalopram OR Lexapro OR Clobazam OR "HR 376" OR Onfi OR "LM 2717" OR Frisium OR Urbanyl OR Clomipramine OR Chlomipramine OR Chlorimipramine OR Hydiphen OR Anafranil OR Clonazepam OR "Ro 5 4023" OR "Ro 54023" OR Antelepsin OR Rivotril OR Clopenthixol OR Zuclopenthixol OR Cisordinol OR Clorazepate OR Chlorazepate OR Tranxene OR Tranxilium OR "4306 CB" OR Clozapine OR Clozaril OR Leponex OR Desipramine OR Desmethylimipramine OR Demethylimipramine OR Norpramin OR Pertofrane OR Pertrofran OR Pertofran OR Petylyl OR Desvenlafaxine OR "O Desmethylvenlafaxine" OR "WY 45,233" OR "WY 45,233" OR "WY45,233" OR "WY 45233" OR WY45233 OR Pristiq OR Dextroamphetamine OR Dexamphetamine OR Dexamfetamine OR "Dextro Amphetamine" OR "D Amphetamine" OR Dexedrine OR DextroStat OR Oxydess OR Diazepam OR Diazemuls OR Faustan OR Valium OR Seduxen OR Sibazon OR Stesolid OR Apaurin OR Relanium OR "Valproic Acid" OR Divalproex OR "Propylisopropylacetic Acid" OR "2 Propylpentanoic Acid" OR Convulsofin OR Depakene OR Depakine OR Depakote OR Vupral OR Valproate OR Ergenyl OR "Dipropyl Acetate" OR Domperidone OR Domperidon OR Domidon OR Gastrocure OR Motilium OR Nauzelin OR Peridys OR "R 33,812" OR "R33,812" OR "R 33812" OR R33812 OR Dothiepin OR Dosulepin OR Prothiaden OR Doxepin OR Deptran OR Desidox OR Doneurin OR Doxepia OR Espadox OR Mareen OR Prudoxin OR Quitaxon OR Sinequan OR Sinquan OR Zonalon OR Xepin OR Aponal OR ApoDoxepin OR Droperidol OR Inapsine OR Dehidrobenzperidol OR Dehydrobenzperidol OR Droleptan OR Duloxetine OR "LY 248686" OR LY248686 OR "LY 227942" OR LY227942 OR Cymbalta OR Estazolam OR Tasedan OR ProSom OR "D 40TA" OR D40TA OR Nuctalon OR Eszopiclone OR Lunesta OR Estorra OR Flunitrazepam OR Fluridrazepam OR Flunibeta OR Flunimerck OR Fluninoc OR Rohypnol OR Rohipnol OR Narcozep OR "Flunizep von CT" OR "RO 5 4200" OR RO54200 OR Fluoxetine OR Fluoxetin OR "Lilly 110140" OR Lilly110140 OR Sarafem OR Prozac OR Flupenthixol OR Flupentixol OR Emergil OR Fluanxol OR Fluphenazine OR Flufenazin OR Lyogen OR Prolixin OR Fluvoxamine OR Fluvoxadura OR Fluvoxamin OR Fluvoxamina OR Luvox OR Fevarin OR Floxyfral OR Dumirox OR Faverin OR Desiflu OR "DU 23000" OR DU23000 OR Guanfacine OR Tenex OR Lon798 OR "BS 100 141" OR BS100141 OR Estulic OR Haloperidol OR Haldol OR Iloperidone OR Zomaril OR Fanapt OR "HP 873" OR Imipramine OR Imizin OR Norchlorimipramine OR Imidobenzyle OR Tofranil OR Melipramine OR Pryleugan OR Janimine OR Isocarboxazid OR Lamotrigine OR Crisomet OR Lamictal OR Lamiktal OR "BW 430C" OR Labileno OR Methotrimeprazine OR Levomepromazine OR Levopromazine OR Levomeprazin OR Tisercin OR Tizercine OR Tizertsin OR Lithium OR Dilithium OR Lithane OR Lithobid OR Lithonate OR "CP-15,467 61" OR "CP15,46761" OR Micalith OR "NSC 16895" OR NSC16895 OR Priadel OR "Quilinorm Retard" OR Quilinormretard OR Eskalith OR Lithotabs OR Lorazepam OR Ativan OR Temesta OR "Orfidal Wyeth" OR Donix OR Duralozam OR Durazolam OR Idalprem OR Laubeel OR "Lorazep von CT" OR "Novo Lorazem" OR NovoLorazem OR "Nu Loraz" OR NuLoraz OR Sedicepan OR Sinestron OR Somagerol OR Tolid OR "WY 4036" OR WY4036 OR ApoLorazepam OR Loxapine OR Cloxazepine OR Oxilapine OR Loxitane OR Loxipine OR Loxapinsuccinate OR "CL 71,563" OR "CL71,563" OR Lurasidone OR "SM 13496" OR SM13496 OR "SM-13,496" OR "SM13,496" OR Latuda OR Methylphenidate OR Metadate OR Equasym OR Methylin OR Concerta OR Phenidylate OR Ritalin OR Ritaline OR Tsentedrin OR Centedrin OR Daytrana OR Mianserin OR Tolvon OR Lerivon OR Org GB 94 OR Midazolam OR Dormicum OR Versed OR "Ro 21 3981" OR "Ro 213981" OR Milnacipran OR Midalcipran OR Levomilnacipran OR Savella OR "F 2207" OR Ixel OR Mirtazapine OR "6 Azamianserin" OR Esmirtazapine OR Remeron OR Remergil OR Zispin OR Norset OR Rexer OR "Org 50081" OR " OR G 3770" OR Moclobemide OR Moclobamide OR Arima OR Aurorix OR Manerix OR Moclamine OR Aurorex OR Deprenorm OR Feraken OR Moclobemid OR Moclobeta OR Moclodura OR Moclonorm OR Rimoc OR "Ro 11 1163" OR Modafinil OR Benzhydrylsulfinylacetamide OR "CRL 40476" OR Vigil OR Provigil OR Sparlon OR Alertec OR Modiodal OR Molindone OR Moban OR Nefazodone OR Rulivan OR Serzone OR Dutonin OR Nefadar OR Menfazona OR Nitrazepam OR Nitrodiazepam OR "Dormo Puren" OR Eatan OR Imadorm OR Imeson OR Mogadon OR Nitrazadon OR Nitrazep OR Novanox OR Radedorm OR Remnos OR Serenade OR Somnite OR Alodorm OR Dormalon OR Nortriptyline OR Desmethylamitriptylin OR Desitriptyline OR Aventyl OR Paxtibi OR Allegron OR Norfenazin OR Pamelor OR Nortrilen OR Olanzapine OR Zolafren OR "LY 170052" OR Zyprexa OR "LY 170053" OR Oxazepam OR Serax OR Tazepam OR Adumbran OR Oxcarbazepine OR Timox OR Trileptal OR "GP 47680" OR Paliperidone OR "9 OH Risperidone" OR "9 Hydroxy Risperidone" OR "9 Hydroxyrisperidone" OR Invega OR "R 76477" OR R76477 OR Paroxetine OR "BRL 29060" OR BRL29060 OR "FG 7051" OR FG7051 OR Seroxat OR Paxil OR Aropax OR Periciazine OR Propericiazine OR Pericyazine OR Neuleptil OR Neuleptyl OR Aolept OR Phenelzine OR "Beta Phenylethylhydrazine" OR "2 Phenethylhydrazine" OR Fenelzin OR Phenethylhydrazine OR Nardelzine OR Nardil OR Phenobarbital OR Phenobarbitone OR "Phenylethylbarbituric Acid" OR Phenemal OR Phenylbarbital OR Hysteps OR Luminal OR Gardenal OR Pindolol OR Prindolol OR Visken OR "LB 46" OR LB46 OR Prazepam OR Lysanxia OR Reapam OR Centrax OR Demetrin OR Pregabalin OR "3 Isobutyl GABA" OR Lyrica OR "CI 1008" OR CI1008 OR Prochlorperazine OR Compazine OR Promazine OR Sparine OR Sinophenin OR Protactyl OR Promethazine OR Prometazin OR Proazamine OR Rumergan OR Diprazin OR Phenergan OR Phenargan OR Phensedyl OR Pipolfen OR Pipolphen OR Promet OR Prothazin OR Pyrethia OR Remsed OR Atosil OR Diphergan OR Propranolol OR Propanolol OR Inderal OR Avlocardyl OR "AY 20694" OR AY20694 OR Rexigen OR Dexpropranolol OR Dociton OR Obsidan OR Obzidan OR Anaprilin OR Anapriline OR Betadren OR Protriptyline OR Vivactil OR Quetiapine OR "ICI 204,636" OR "ICI 204636" OR ICI204636 OR Seroquel OR Reboxetine OR Vestra OR Remoxipride OR "FLA 731" OR FLA731 OR Risperidone OR Risperdal OR Risperidal OR "R 64,766" OR "R64,766" OR "R 64766" OR R64766 OR Selegiline OR Selegyline OR "L Deprenyl" OR "E 250" OR E250 OR Eldepryl OR Emsam OR Zelapar OR Deprenil OR Deprenalin OR Yumex OR Jumex OR Humex OR Deprenyl OR Sertindole OR Serlect OR "Lu 23 174" OR Serdolect OR Sertraline OR Zoloft OR Altruline OR Lustral OR Aremis OR Besitran OR Sealdin OR Gladem OR Sulpiride OR Sulperide OR Arminol OR Deponerton OR Meresa OR Desisulpid OR Digton OR Dogmatil OR Dolmatil OR Eglonyl OR Ekilid OR Guastil OR Lebopride OR Neogama OR Pontiride OR Psicocen OR Sulp OR Sulpitil OR Sulpivert OR Sulpor OR Synedil OR Tepavil OR Aiglonyl OR Temazepam OR Hydroxydiazepam OR Methyloxazepam OR Signopam OR Tenox OR "WY 3917" OR WY3917 OR Dasuen OR Euhypnos OR Levanxol OR "Norkotral Tema" OR Normison OR Nocturne OR Temtabs OR Normitab OR Nortem OR Planum OR "Pronervon T" OR Remestan OR Restoril OR "Ro 5 5345" OR Ro55345 OR "SaH 47 603" OR "SaH 47603" OR Temaze OR "Temazep von CT" OR Thioridazine OR ApoThioridazine OR Meleril OR Melleril OR Melleryl OR Mellaril OR Melleretten OR Melzine OR Thiozine OR Sonapax OR Thioridazineneurazpharm OR Aldazine OR Rideril OR Thiothixene OR Tiotixene OR Navane OR Topiramate OR USL255 OR "McN 4853" OR Topamax OR Epitomax OR Tranylcypromine OR "Trans 2 Phenylcyclopropylamine" OR Jatrosom OR Transamine OR Parnate OR Trazodone OR Tradozone OR "AF 1161" OR AF1161 OR Deprax OR Desyrel OR Molipaxin OR Trittico OR Thombran OR "Trazodon Hexal" OR "Trazodon Neuraxpharm" OR Trazon OR Triazolam OR "U 33,030" OR "U33,030" OR Halcion OR Trilam OR "Apo Triazo" OR Trifluoperazine OR Trifluoroperazine OR Trifluperazine OR Eskazine OR Flupazine OR Terfluzine OR Triftazin OR Stelazine OR Trimipramine OR Trimeprimine OR Herphonal OR Trimineurin OR NovoTripramine OR Rhotrimine OR Stangyl OR Surmontil OR Trimidura OR Trimineurin OR Trimipramin OR "Apo Trimip" OR ApoTrimip OR Eldoral OR Venlafaxine OR "Wy 45030" OR Wy45030 OR "Wy 45,030" OR "Wy45,030" OR Effexor OR Trevilor OR Vandral OR Efexor OR Dobupal OR Vigabatrin OR "Gamma Vinyl GABA" OR "Gamma Vinyl Gamma Aminobutyric Acid" OR Sabril OR Sabrilex OR Vortioxetine OR Brintellix OR "Lu AA21004" OR LuAA21004 OR Zaleplon OR "SKP 1041" OR Sonata OR Zelepion OR Starnoc OR "CL 284,846" OR "CL284,846" OR "CL 284846" OR "L 846" OR Ziprasidone OR Ziprazidone OR "CP 88,059" OR "CP 88059" OR Zolpidem OR Amsic OR Bikalm OR Dalparan OR "SL 80.0750" OR "SL 800750 23 N" OR Stilnoct OR Stilnox OR Zodormdura OR Zoldem OR Zolirin OR "Zolpi Lich" OR Zolpinox OR Zolpimist OR Ambien OR Zopiclone OR Zop OR Zopicalma OR Zopiclodura OR Zopiclon OR Zopitan OR Zorclone OR Imovane OR Ximovan OR Zimovane OR Limovan OR Optidorm OR Rhovane OR "RP 27 267" OR Siaten OR Somnosan OR Zileze OR Zimoclone OR "Zopi Puren" OR Zopicalm OR Zotepine OR Zoleptil OR Nipolept OR Zuclopenthixol OR Zuclopentixol OR Clopixol OR Zuclopenthixole OR Acuphase ) OR AB ( ((Adrenergic) N1 Beta N2 (Antagonist OR Block*)) OR Anti Anxiety OR Anti Convuls* OR Anti Depress* OR Anti Epilep* OR Anti Psychotic* OR Antianxiety OR Anticonvuls* OR Antidepress* OR Antiepilep* OR Antipsychotic* OR Anxiolytic* OR Benzodiazepine* OR (Beta N1 Block*) OR (Beta N1 Adrenergic N2 Block*) OR Thymoanaleptic* OR Thymoleptic* OR Agomelatine OR "S 20098" OR S20098 OR Thymanax OR Valdoxan OR "AGO 178" OR AGO178 OR Alprazolam OR Alprazolan OR "Apo Alpraz" OR ApoAlpraz OR Cassadan OR "D 65MT" OR D65MT OR Xanax OR Tafil OR Trankimazin OR "Novo Alprazol" OR NovoAlprazol OR "Nu Alpraz" OR NuAlpraz OR Ralozam OR "U-31,889" OR "U31,889" OR Alprox OR Esparon OR Kalma OR Amisulpride OR Sultopride OR Barnetil OR "DAN 2163" OR Solian OR "LIN 1418" OR Amitriptyline OR Amineurin OR Amitrip OR Amitriptylin OR Amitrol OR Tryptine OR ApoAmitriptyline OR Damilen OR Domical OR Laroxyl OR Endep OR Lentizol OR Novoprotect OR Saroten OR Sarotex OR Syneudon OR Triptafen OR Tryptizol OR Tryptanol OR Elavil OR Anapsique OR Amoxapine OR Desmethylloxapine OR "CL 67,772" OR "CL67,772" OR Demolox OR Asendin OR Defanyl OR Asendis OR Aripiprazole OR Aripiprazol OR "OPC 14597" OR Abilify OR Asenapine OR Saphris OR "OR G 5222" OR Atenolol OR Tenormine OR Tenormin OR "ICI 66082" OR ICI66082 OR Atomoxetine OR Tomoxetine OR Strattera OR "LY 139603" OR Benztropine OR Benzatropine OR Bensylate OR PMSBenztropine OR Cogentin OR Cogentinol OR Methylbenztropine OR ApoBenztropine OR Brexpiprazole OR Bromazepam OR BromaLich OR "Bromaz 1A Pharma" OR Bromazanil OR "Bromazep von CT" OR Durazanil OR Lexotan OR Lexotanil OR Lexatin OR Lexomil OR "Ro 5-3350" OR "Ro 53350" OR Anxyrex OR Bupropion OR Amfebutamone OR Zyntabac OR Quomen OR Wellbutrin OR Zyban OR Buspirone OR "MJ 9022 1" OR MJ90221 OR Neurosine OR Busp OR Anxut OR Buspar OR Bespar OR Carbamazepine OR Tegretol OR Carbazepin OR Epitol OR Finlepsin OR Neurotol OR Amizepine OR Cariprazine OR "RGH 188" OR Chlordiazepoxide OR Methaminodiazepoxide OR Librium OR Chlozepid OR Elenium OR Chlorpromazine OR Thorazine OR Aminazine OR Largactil OR Chlordelazine OR Contomin OR Fenactil OR Propaphenin OR Chlorazine OR Citalopram OR Cytalopram OR "Lu 10 171" OR Lu10171 OR Escitalopram OR Lexapro OR Clobazam OR "HR 376" OR Onfi OR "LM 2717" OR Frisium OR Urbanyl OR Clomipramine OR Chlomipramine OR Chlorimipramine OR Hydiphen OR Anafranil OR Clonazepam OR "Ro 5 4023" OR "Ro 54023" OR Antelepsin OR Rivotril OR Clopenthixol OR Zuclopenthixol OR Cisordinol OR Clorazepate OR Chlorazepate OR Tranxene OR Tranxilium OR "4306 CB" OR Clozapine OR Clozaril OR Leponex OR Desipramine OR Desmethylimipramine OR Demethylimipramine OR Norpramin OR Pertofrane OR Pertrofran OR Pertofran OR Petylyl OR Desvenlafaxine OR "O Desmethylvenlafaxine" OR "WY 45,233" OR "WY 45,233" OR "WY45,233" OR "WY 45233" OR WY45233 OR Pristiq OR Dextroamphetamine OR Dexamphetamine OR Dexamfetamine OR "Dextro Amphetamine" OR "D Amphetamine" OR Dexedrine OR DextroStat OR Oxydess OR Diazepam OR Diazemuls OR Faustan OR Valium OR Seduxen OR Sibazon OR Stesolid OR Apaurin OR Relanium OR "Valproic Acid" OR Divalproex OR "Propylisopropylacetic Acid" OR "2 Propylpentanoic Acid" OR Convulsofin OR Depakene OR Depakine OR Depakote OR Vupral OR Valproate OR Ergenyl OR "Dipropyl Acetate" OR Domperidone OR Domperidon OR Domidon OR Gastrocure OR Motilium OR Nauzelin OR Peridys OR "R 33,812" OR "R33,812" OR "R 33812" OR R33812 OR Dothiepin OR Dosulepin OR Prothiaden OR Doxepin OR Deptran OR Desidox OR Doneurin OR Doxepia OR Espadox OR Mareen OR Prudoxin OR Quitaxon OR Sinequan OR Sinquan OR Zonalon OR Xepin OR Aponal OR ApoDoxepin OR Droperidol OR Inapsine OR Dehidrobenzperidol OR Dehydrobenzperidol OR Droleptan OR Duloxetine OR "LY 248686" OR LY248686 OR "LY 227942" OR LY227942 OR Cymbalta OR Estazolam OR Tasedan OR ProSom OR "D 40TA" OR D40TA OR Nuctalon OR Eszopiclone OR Lunesta OR Estorra OR Flunitrazepam OR Fluridrazepam OR Flunibeta OR Flunimerck OR Fluninoc OR Rohypnol OR Rohipnol OR Narcozep OR "Flunizep von CT" OR "RO 5 4200" OR RO54200 OR Fluoxetine OR Fluoxetin OR "Lilly 110140" OR Lilly110140 OR Sarafem OR Prozac OR Flupenthixol OR Flupentixol OR Emergil OR Fluanxol OR Fluphenazine OR Flufenazin OR Lyogen OR Prolixin OR Fluvoxamine OR Fluvoxadura OR Fluvoxamin OR Fluvoxamina OR Luvox OR Fevarin OR Floxyfral OR Dumirox OR Faverin OR Desiflu OR "DU 23000" OR DU23000 OR Guanfacine OR Tenex OR Lon798 OR "BS 100 141" OR BS100141 OR Estulic OR Haloperidol OR Haldol OR Iloperidone OR Zomaril OR Fanapt OR "HP 873" OR Imipramine OR Imizin OR Norchlorimipramine OR Imidobenzyle OR Tofranil OR Melipramine OR Pryleugan OR Janimine OR Isocarboxazid OR Lamotrigine OR Crisomet OR Lamictal OR Lamiktal OR "BW 430C" OR Labileno OR Methotrimeprazine OR Levomepromazine OR Levopromazine OR Levomeprazin OR Tisercin OR Tizercine OR Tizertsin OR Lithium OR Dilithium OR Lithane OR Lithobid OR Lithonate OR "CP-15,467 61" OR "CP15,46761" OR Micalith OR "NSC 16895" OR NSC16895 OR Priadel OR "Quilinorm Retard" OR Quilinormretard OR Eskalith OR Lithotabs OR Lorazepam OR Ativan OR Temesta OR "Orfidal Wyeth" OR Donix OR Duralozam OR Durazolam OR Idalprem OR Laubeel OR "Lorazep von CT" OR "Novo Lorazem" OR NovoLorazem OR "Nu Loraz" OR NuLoraz OR Sedicepan OR Sinestron OR Somagerol OR Tolid OR "WY 4036" OR WY4036 OR ApoLorazepam OR Loxapine OR Cloxazepine OR Oxilapine OR Loxitane OR Loxipine OR Loxapinsuccinate OR "CL 71,563" OR "CL71,563" OR Lurasidone OR "SM 13496" OR SM13496 OR "SM-13,496" OR "SM13,496" OR Latuda OR Methylphenidate OR Metadate OR Equasym OR Methylin OR Concerta OR Phenidylate OR Ritalin OR Ritaline OR Tsentedrin OR Centedrin OR Daytrana OR Mianserin OR Tolvon OR Lerivon OR Org GB 94 OR Midazolam OR Dormicum OR Versed OR "Ro 21 3981" OR "Ro 213981" OR Milnacipran OR Midalcipran OR Levomilnacipran OR Savella OR "F 2207" OR Ixel OR Mirtazapine OR "6 Azamianserin" OR Esmirtazapine OR Remeron OR Remergil OR Zispin OR Norset OR Rexer OR "Org 50081" OR " OR G 3770" OR Moclobemide OR Moclobamide OR Arima OR Aurorix OR Manerix OR Moclamine OR Aurorex OR Deprenorm OR Feraken OR Moclobemid OR Moclobeta OR Moclodura OR Moclonorm OR Rimoc OR "Ro 11 1163" OR Modafinil OR Benzhydrylsulfinylacetamide OR "CRL 40476" OR Vigil OR Provigil OR Sparlon OR Alertec OR Modiodal OR Molindone OR Moban OR Nefazodone OR Rulivan OR Serzone OR Dutonin OR Nefadar OR Menfazona OR Nitrazepam OR Nitrodiazepam OR "Dormo Puren" OR Eatan OR Imadorm OR Imeson OR Mogadon OR Nitrazadon OR Nitrazep OR Novanox OR Radedorm OR Remnos OR Serenade OR Somnite OR Alodorm OR Dormalon OR Nortriptyline OR Desmethylamitriptylin OR Desitriptyline OR Aventyl OR Paxtibi OR Allegron OR Norfenazin OR Pamelor OR Nortrilen OR Olanzapine OR Zolafren OR "LY 170052" OR Zyprexa OR "LY 170053" OR Oxazepam OR Serax OR Tazepam OR Adumbran OR Oxcarbazepine OR Timox OR Trileptal OR "GP 47680" OR Paliperidone OR "9 OH Risperidone" OR "9 Hydroxy Risperidone" OR "9 Hydroxyrisperidone" OR Invega OR "R 76477" OR R76477 OR Paroxetine OR "BRL 29060" OR BRL29060 OR "FG 7051" OR FG7051 OR Seroxat OR Paxil OR Aropax OR Periciazine OR Propericiazine OR Pericyazine OR Neuleptil OR Neuleptyl OR Aolept OR Phenelzine OR "Beta Phenylethylhydrazine" OR "2 Phenethylhydrazine" OR Fenelzin OR Phenethylhydrazine OR Nardelzine OR Nardil OR Phenobarbital OR Phenobarbitone OR "Phenylethylbarbituric Acid" OR Phenemal OR Phenylbarbital OR Hysteps OR Luminal OR Gardenal OR Pindolol OR Prindolol OR Visken OR "LB 46" OR LB46 OR Prazepam OR Lysanxia OR Reapam OR Centrax OR Demetrin OR Pregabalin OR "3 Isobutyl GABA" OR Lyrica OR "CI 1008" OR CI1008 OR Prochlorperazine OR Compazine OR Promazine OR Sparine OR Sinophenin OR Protactyl OR Promethazine OR Prometazin OR Proazamine OR Rumergan OR Diprazin OR Phenergan OR Phenargan OR Phensedyl OR Pipolfen OR Pipolphen OR Promet OR Prothazin OR Pyrethia OR Remsed OR Atosil OR Diphergan OR Propranolol OR Propanolol OR Inderal OR Avlocardyl OR "AY 20694" OR AY20694 OR Rexigen OR Dexpropranolol OR Dociton OR Obsidan OR Obzidan OR Anaprilin OR Anapriline OR Betadren OR Protriptyline OR Vivactil OR Quetiapine OR "ICI 204,636" OR "ICI 204636" OR ICI204636 OR Seroquel OR Reboxetine OR Vestra OR Remoxipride OR "FLA 731" OR FLA731 OR Risperidone OR Risperdal OR Risperidal OR "R 64,766" OR "R64,766" OR "R 64766" OR R64766 OR Selegiline OR Selegyline OR "L Deprenyl" OR "E 250" OR E250 OR Eldepryl OR Emsam OR Zelapar OR Deprenil OR Deprenalin OR Yumex OR Jumex OR Humex OR Deprenyl OR Sertindole OR Serlect OR "Lu 23 174" OR Serdolect OR Sertraline OR Zoloft OR Altruline OR Lustral OR Aremis OR Besitran OR Sealdin OR Gladem OR Sulpiride OR Sulperide OR Arminol OR Deponerton OR Meresa OR Desisulpid OR Digton OR Dogmatil OR Dolmatil OR Eglonyl OR Ekilid OR Guastil OR Lebopride OR Neogama OR Pontiride OR Psicocen OR Sulp OR Sulpitil OR Sulpivert OR Sulpor OR Synedil OR Tepavil OR Aiglonyl OR Temazepam OR Hydroxydiazepam OR Methyloxazepam OR Signopam OR Tenox OR "WY 3917" OR WY3917 OR Dasuen OR Euhypnos OR Levanxol OR "Norkotral Tema" OR Normison OR Nocturne OR Temtabs OR Normitab OR Nortem OR Planum OR "Pronervon T" OR Remestan OR Restoril OR "Ro 5 5345" OR Ro55345 OR "SaH 47 603" OR "SaH 47603" OR Temaze OR "Temazep von CT" OR Thioridazine OR ApoThioridazine OR Meleril OR Melleril OR Melleryl OR Mellaril OR Melleretten OR Melzine OR Thiozine OR Sonapax OR Thioridazineneurazpharm OR Aldazine OR Rideril OR Thiothixene OR Tiotixene OR Navane OR Topiramate OR USL255 OR "McN 4853" OR Topamax OR Epitomax OR Tranylcypromine OR "Trans 2 Phenylcyclopropylamine" OR Jatrosom OR Transamine OR Parnate OR Trazodone OR Tradozone OR "AF 1161" OR AF1161 OR Deprax OR Desyrel OR Molipaxin OR Trittico OR Thombran OR "Trazodon Hexal" OR "Trazodon Neuraxpharm" OR Trazon OR Triazolam OR "U 33,030" OR "U33,030" OR Halcion OR Trilam OR "Apo Triazo" OR Trifluoperazine OR Trifluoroperazine OR Trifluperazine OR Eskazine OR Flupazine OR Terfluzine OR Triftazin OR Stelazine OR Trimipramine OR Trimeprimine OR Herphonal OR Trimineurin OR NovoTripramine OR Rhotrimine OR Stangyl OR Surmontil OR Trimidura OR Trimineurin OR Trimipramin OR "Apo Trimip" OR ApoTrimip OR Eldoral OR Venlafaxine OR "Wy 45030" OR Wy45030 OR "Wy 45,030" OR "Wy45,030" OR Effexor OR Trevilor OR Vandral OR Efexor OR Dobupal OR Vigabatrin OR "Gamma Vinyl GABA" OR "Gamma Vinyl Gamma Aminobutyric Acid" OR Sabril OR Sabrilex OR Vortioxetine OR Brintellix OR "Lu AA21004" OR LuAA21004 OR Zaleplon OR "SKP 1041" OR Sonata OR Zelepion OR Starnoc OR "CL 284,846" OR "CL284,846" OR "CL 284846" OR "L 846" OR Ziprasidone OR Ziprazidone OR "CP 88,059" OR "CP 88059" OR Zolpidem OR Amsic OR Bikalm OR Dalparan OR "SL 80.0750" OR "SL 800750 23 N" OR Stilnoct OR Stilnox OR Zodormdura OR Zoldem OR Zolirin OR "Zolpi Lich" OR Zolpinox OR Zolpimist OR Ambien OR Zopiclone OR Zop OR Zopicalma OR Zopiclodura OR Zopiclon OR Zopitan OR Zorclone OR Imovane OR Ximovan OR Zimovane OR Limovan OR Optidorm OR Rhovane OR "RP 27 267" OR Siaten OR Somnosan OR Zileze OR Zimoclone OR "Zopi Puren" OR Zopicalm OR Zotepine OR Zoleptil OR Nipolept OR Zuclopenthixol OR Zuclopentixol OR Clopixol OR Zuclopenthixole OR Acuphase ) ) AND ( ( (MH "Depression") OR (MH "Depression, Reactive") OR (MH "Dysthymic Disorder") OR (MH "Self-Rating Depression Scale") OR (MH "Beck Depression Inventory, Revised Edition") OR (MH "Center for Epidemiological Studies Depression Scale") OR (MH "Hamilton Rating Scale for Depression") OR (MH "Dysthymic Disorder") OR (MH "Cyclothymic Disorder") OR (MH "Affective Disorders") OR (MH "Affective Disorders, Psychotic") ) OR TI ( "Affective Disorder" or "Affective Disorders" or "Affective Psychoses" or "Affective Psychosis" or Cyclothymic or Depress* or Dysthym* or "Mood Disorder" or "Mood Disorders" ) OR AB ( "Affective Disorder" or "Affective Disorders" or "Affective Psychoses" or "Affective Psychosis" or Cyclothymic or Depress* or Dysthym* or "Mood Disorder" or "Mood Disorders" ) ) AND EM 201803-

**B. ClinicalTrials.Gov**

Advanced Search

Condition or disease: (Brain OR Cerebr* OR Cranial OR Cranio* OR "Diffuse Axonal" OR Head OR Hemisphere* OR Trauma* OR Posttrauma* OR Concuss*) AND (Affective OR Cyclothymic OR Depression OR Depressive OR Dysthymic OR Mood)

Study type: Interventional Studies (Clinical Trials)

First Posted: From 03/01/2018 to (MM/DD/YYYY)

**C. CENTRAL**

([mh "Brain Hemorrhage, Traumatic"] OR [mh ^"Brain Injuries"] OR [mh ^"Brain Injury, Chronic"] OR [mh ^"Cerebral Hemorrhage, Traumatic"] OR [mh ^"Cerebrovascular Trauma"] OR [mh ^"Craniocerebral Trauma"] OR [mh ^"Diffuse Axonal Injury"] OR [mh "Head Injuries, Closed"] OR [mh ^"Head Injuries, Penetrating"] OR [mh "Intracranial Hemorrhage, Traumatic"] OR [mh Pneumocephalus] OR (((Brain OR Cerebr* OR Crani* OR "Crushing Skull" OR "Diffuse Axonal" OR Head OR Hemisphere*) NEAR/1 (Injur* OR Trauma*)) OR ((Cerebr* OR Crani* OR Head) NEAR/1 (Lesion* OR Wound*)) OR ((Posttraumatic OR Traumatic) NEAR/1 Encephalopath*) OR (Traumatic NEAR/1 (Brain OR Cerebr*)) OR Concuss* OR DAI OR DAIs OR Pneumocephalus OR TBI OR TBIs):ti,ab) AND ([mh "Adrenergic Beta-Antagonists"] or [mh "Anti-Anxiety Agents"] or [mh Anticonvulsants] or [mh "Antidepressive Agents"] or [mh "Antipsychotic Agents"] or [mh "Atomoxetine Hydrochloride"] or [mh Benzodiazepines] or [mh "Clorazepate Dipotassium"] or [mh "Desvenlafaxine Succinate"] or [mh "Duloxetine Hydrochloride"] or [mh "Lithium Carbonate"] or [mh "Lurasidone Hydrochloride"] or [mh "Paliperidone Palmitate"] or [mh "Quetiapine Fumarate"] or [mh "Venlafaxine Hydrochloride"] or [mh Alprazolam] or [mh Amitriptyline] or [mh Amoxapine] or [mh Aripiprazole] or [mh Atenolol] or [mh Benztropine] or [mh Bromazepam] or [mh Bupropion] or [mh Buspirone] or [mh Carbamazepine] or [mh Chlordiazepoxide] or [mh Chlorpromazine] or [mh Citalopram] or [mh Clomipramine] or [mh Clonazepam] or [mh Clopenthixol] or [mh Clozapine] or [mh Desipramine] or [mh Dextroamphetamine] or [mh Diazepam] or [mh Domperidone] or [mh Dothiepin] or [mh Doxepin] or [mh Droperidol] or [mh Estazolam] or [mh Eszopiclone] or [mh Flunitrazepam] or [mh Fluoxetine] or [mh Flupenthixol] or [mh Fluphenazine] or [mh Fluvoxamine] or [mh Guanfacine] or [mh Haloperidol] or [mh Imipramine] or [mh Isocarboxazid] or [mh Lithium] or [mh Lorazepam] or [mh Loxapine] or [mh Methotrimeprazine] or [mh Methylphenidate] or [mh Mianserin] or [mh Midazolam] or [mh Moclobemide] or [mh Molindone] or [mh Nitrazepam] or [mh Nortriptyline] or [mh Oxazepam] or [mh Paroxetine] or [mh Phenelzine] or [mh Phenobarbital] or [mh Pindolol] or [mh Prazepam] or [mh Pregabalin] or [mh Prochlorperazine] or [mh Promazine] or [mh Promethazine] or [mh Propranolol] or [mh Protriptyline] or [mh Remoxipride] or [mh Risperidone] or [mh Selegiline] or [mh Sertraline] or [mh Sulpiride] or [mh Temazepam] or [mh Thioridazine] or [mh Thiothixene] or [mh Tranylcypromine] or [mh Trazodone] or [mh Triazolam] or [mh Trifluoperazine] or [mh Trimipramine] or [mh "Valproic Acid"] or [mh Vigabatrin] OR ((Adrenergic NEAR/1 Beta NEAR/2 (Antagonist OR Block*)) OR Anti Anxiety OR Anti Convuls* OR Anti Depress* OR Anti Epilep* OR Anti Psychotic* OR Antianxiety OR Anticonvuls* OR Antidepress* OR Antiepilep* OR Antipsychotic* OR Anxiolytic* OR Benzodiazepine* OR (Beta NEAR/1 Block*) OR (Beta NEAR/1 Adrenergic NEAR/2 Block*) OR Thymoanaleptic* OR Thymoleptic* OR Agomelatine OR "S 20098" OR S20098 OR Thymanax OR Valdoxan OR "AGO 178" OR AGO178 OR Alprazolam OR Alprazolan OR "Apo Alpraz" OR ApoAlpraz OR Cassadan OR "D 65MT" OR D65MT OR Xanax OR Tafil OR Trankimazin OR "Novo Alprazol" OR NovoAlprazol OR "Nu Alpraz" OR NuAlpraz OR Ralozam OR "U-31,889" OR "U31,889" OR Alprox OR Esparon OR Kalma OR Amisulpride OR Sultopride OR Barnetil OR "DAN 2163" OR Solian OR "LIN 1418" OR Amitriptyline OR Amineurin OR Amitrip OR Amitriptylin OR Amitrol OR Tryptine OR ApoAmitriptyline OR Damilen OR Domical OR Laroxyl OR Endep OR Lentizol OR Novoprotect OR Saroten OR Sarotex OR Syneudon OR Triptafen OR Tryptizol OR Tryptanol OR Elavil OR Anapsique OR Amoxapine OR Desmethylloxapine OR "CL 67,772" OR "CL67,772" OR Demolox OR Asendin OR Defanyl OR Asendis OR Aripiprazole OR Aripiprazol OR "OPC 14597" OR Abilify OR Asenapine OR Saphris OR "OR G 5222" OR Atenolol OR Tenormine OR Tenormin OR "ICI 66082" OR ICI66082 OR Atomoxetine OR Tomoxetine OR Strattera OR "LY 139603" OR Benztropine OR Benzatropine OR Bensylate OR PMSBenztropine OR Cogentin OR Cogentinol OR Methylbenztropine OR ApoBenztropine OR Brexpiprazole OR Bromazepam OR BromaLich OR "Bromaz 1A Pharma" OR Bromazanil OR "Bromazep von CT" OR Durazanil OR Lexotan OR Lexotanil OR Lexatin OR Lexomil OR "Ro 5-3350" OR "Ro 53350" OR Anxyrex OR Bupropion OR Amfebutamone OR Zyntabac OR Quomen OR Wellbutrin OR Zyban OR Buspirone OR "MJ 9022 1" OR MJ90221 OR Neurosine OR Busp OR Anxut OR Buspar OR Bespar OR Carbamazepine OR Tegretol OR Carbazepin OR Epitol OR Finlepsin OR Neurotol OR Amizepine OR Cariprazine OR "RGH 188" OR Chlordiazepoxide OR Methaminodiazepoxide OR Librium OR Chlozepid OR Elenium OR Chlorpromazine OR Thorazine OR Aminazine OR Largactil OR Chlordelazine OR Contomin OR Fenactil OR Propaphenin OR Chlorazine OR Citalopram OR Cytalopram OR "Lu 10 171" OR Lu10171 OR Escitalopram OR Lexapro OR Clobazam OR "HR 376" OR Onfi OR "LM 2717" OR Frisium OR Urbanyl OR Clomipramine OR Chlomipramine OR Chlorimipramine OR Hydiphen OR Anafranil OR Clonazepam OR "Ro 5 4023" OR "Ro 54023" OR Antelepsin OR Rivotril OR Clopenthixol OR Zuclopenthixol OR Cisordinol OR Clorazepate OR Chlorazepate OR Tranxene OR Tranxilium OR "4306 CB" OR Clozapine OR Clozaril OR Leponex OR Desipramine OR Desmethylimipramine OR Demethylimipramine OR Norpramin OR Pertofrane OR Pertrofran OR Pertofran OR Petylyl OR Desvenlafaxine OR "O Desmethylvenlafaxine" OR "WY 45,233" OR "WY 45,233" OR "WY45,233" OR "WY 45233" OR WY45233 OR Pristiq OR Dextroamphetamine OR Dexamphetamine OR Dexamfetamine OR "Dextro Amphetamine" OR "D Amphetamine" OR Dexedrine OR DextroStat OR Oxydess OR Diazepam OR Diazemuls OR Faustan OR Valium OR Seduxen OR Sibazon OR Stesolid OR Apaurin OR Relanium OR "Valproic Acid" OR Divalproex OR "Propylisopropylacetic Acid" OR "2 Propylpentanoic Acid" OR Convulsofin OR Depakene OR Depakine OR Depakote OR Vupral OR Valproate OR Ergenyl OR "Dipropyl Acetate" OR Domperidone OR Domperidon OR Domidon OR Gastrocure OR Motilium OR Nauzelin OR Peridys OR "R 33,812" OR "R33,812" OR "R 33812" OR R33812 OR Dothiepin OR Dosulepin OR Prothiaden OR Doxepin OR Deptran OR Desidox OR Doneurin OR Doxepia OR Espadox OR Mareen OR Prudoxin OR Quitaxon OR Sinequan OR Sinquan OR Zonalon OR Xepin OR Aponal OR ApoDoxepin OR Droperidol OR Inapsine OR Dehidrobenzperidol OR Dehydrobenzperidol OR Droleptan OR Duloxetine OR "LY 248686" OR LY248686 OR "LY 227942" OR LY227942 OR Cymbalta OR Estazolam OR Tasedan OR ProSom OR "D 40TA" OR D40TA OR Nuctalon OR Eszopiclone OR Lunesta OR Estorra OR Flunitrazepam OR Fluridrazepam OR Flunibeta OR Flunimerck OR Fluninoc OR Rohypnol OR Rohipnol OR Narcozep OR "Flunizep von CT" OR "RO 5 4200" OR RO54200 OR Fluoxetine OR Fluoxetin OR "Lilly 110140" OR Lilly110140 OR Sarafem OR Prozac OR Flupenthixol OR Flupentixol OR Emergil OR Fluanxol OR Fluphenazine OR Flufenazin OR Lyogen OR Prolixin OR Fluvoxamine OR Fluvoxadura OR Fluvoxamin OR Fluvoxamina OR Luvox OR Fevarin OR Floxyfral OR Dumirox OR Faverin OR Desiflu OR "DU 23000" OR DU23000 OR Guanfacine OR Tenex OR Lon798 OR "BS 100 141" OR BS100141 OR Estulic OR Haloperidol OR Haldol OR Iloperidone OR Zomaril OR Fanapt OR "HP 873" OR Imipramine OR Imizin OR Norchlorimipramine OR Imidobenzyle OR Tofranil OR Melipramine OR Pryleugan OR Janimine OR Isocarboxazid OR Lamotrigine OR Crisomet OR Lamictal OR Lamiktal OR "BW 430C" OR Labileno OR Methotrimeprazine OR Levomepromazine OR Levopromazine OR Levomeprazin OR Tisercin OR Tizercine OR Tizertsin OR Lithium OR Dilithium OR Lithane OR Lithobid OR Lithonate OR "CP-15,467 61" OR "CP15,46761" OR Micalith OR "NSC 16895" OR NSC16895 OR Priadel OR "Quilinorm Retard" OR Quilinormretard OR Eskalith OR Lithotabs OR Lorazepam OR Ativan OR Temesta OR "Orfidal Wyeth" OR Donix OR Duralozam OR Durazolam OR Idalprem OR Laubeel OR "Lorazep von CT" OR "Novo Lorazem" OR NovoLorazem OR "Nu Loraz" OR NuLoraz OR Sedicepan OR Sinestron OR Somagerol OR Tolid OR "WY 4036" OR WY4036 OR ApoLorazepam OR Loxapine OR Cloxazepine OR Oxilapine OR Loxitane OR Loxipine OR Loxapinsuccinate OR "CL 71,563" OR "CL71,563" OR Lurasidone OR "SM 13496" OR SM13496 OR "SM-13,496" OR "SM13,496" OR Latuda OR Methylphenidate OR Metadate OR Equasym OR Methylin OR Concerta OR Phenidylate OR Ritalin OR Ritaline OR Tsentedrin OR Centedrin OR Daytrana OR Mianserin OR Tolvon OR Lerivon OR Org GB 94 OR Midazolam OR Dormicum OR Versed OR "Ro 21 3981" OR "Ro 213981" OR Milnacipran OR Midalcipran OR Levomilnacipran OR Savella OR "F 2207" OR Ixel OR Mirtazapine OR "6 Azamianserin" OR Esmirtazapine OR Remeron OR Remergil OR Zispin OR Norset OR Rexer OR "Org 50081" OR " OR G 3770" OR Moclobemide OR Moclobamide OR Arima OR Aurorix OR Manerix OR Moclamine OR Aurorex OR Deprenorm OR Feraken OR Moclobemid OR Moclobeta OR Moclodura OR Moclonorm OR Rimoc OR "Ro 11 1163" OR Modafinil OR Benzhydrylsulfinylacetamide OR "CRL 40476" OR Vigil OR Provigil OR Sparlon OR Alertec OR Modiodal OR Molindone OR Moban OR Nefazodone OR Rulivan OR Serzone OR Dutonin OR Nefadar OR Menfazona OR Nitrazepam OR Nitrodiazepam OR "Dormo Puren" OR Eatan OR Imadorm OR Imeson OR Mogadon OR Nitrazadon OR Nitrazep OR Novanox OR Radedorm OR Remnos OR Serenade OR Somnite OR Alodorm OR Dormalon OR Nortriptyline OR Desmethylamitriptylin OR Desitriptyline OR Aventyl OR Paxtibi OR Allegron OR Norfenazin OR Pamelor OR Nortrilen OR Olanzapine OR Zolafren OR "LY 170052" OR Zyprexa OR "LY 170053" OR Oxazepam OR Serax OR Tazepam OR Adumbran OR Oxcarbazepine OR Timox OR Trileptal OR "GP 47680" OR Paliperidone OR "9 OH Risperidone" OR "9 Hydroxy Risperidone" OR "9 Hydroxyrisperidone" OR Invega OR "R 76477" OR R76477 OR Paroxetine OR "BRL 29060" OR BRL29060 OR "FG 7051" OR FG7051 OR Seroxat OR Paxil OR Aropax OR Periciazine OR Propericiazine OR Pericyazine OR Neuleptil OR Neuleptyl OR Aolept OR Phenelzine OR "Beta Phenylethylhydrazine" OR "2 Phenethylhydrazine" OR Fenelzin OR Phenethylhydrazine OR Nardelzine OR Nardil OR Phenobarbital OR Phenobarbitone OR "Phenylethylbarbituric Acid" OR Phenemal OR Phenylbarbital OR Hysteps OR Luminal OR Gardenal OR Pindolol OR Prindolol OR Visken OR "LB 46" OR LB46 OR Prazepam OR Lysanxia OR Reapam OR Centrax OR Demetrin OR Pregabalin OR "3 Isobutyl GABA" OR Lyrica OR "CI 1008" OR CI1008 OR Prochlorperazine OR Compazine OR Promazine OR Sparine OR Sinophenin OR Protactyl OR Promethazine OR Prometazin OR Proazamine OR Rumergan OR Diprazin OR Phenergan OR Phenargan OR Phensedyl OR Pipolfen OR Pipolphen OR Promet OR Prothazin OR Pyrethia OR Remsed OR Atosil OR Diphergan OR Propranolol OR Propanolol OR Inderal OR Avlocardyl OR "AY 20694" OR AY20694 OR Rexigen OR Dexpropranolol OR Dociton OR Obsidan OR Obzidan OR Anaprilin OR Anapriline OR Betadren OR Protriptyline OR Vivactil OR Quetiapine OR "ICI 204,636" OR "ICI 204636" OR ICI204636 OR Seroquel OR Reboxetine OR Vestra OR Remoxipride OR "FLA 731" OR FLA731 OR Risperidone OR Risperdal OR Risperidal OR "R 64,766" OR "R64,766" OR "R 64766" OR R64766 OR Selegiline OR Selegyline OR "L Deprenyl" OR "E 250" OR E250 OR Eldepryl OR Emsam OR Zelapar OR Deprenil OR Deprenalin OR Yumex OR Jumex OR Humex OR Deprenyl OR Sertindole OR Serlect OR "Lu 23 174" OR Serdolect OR Sertraline OR Zoloft OR Altruline OR Lustral OR Aremis OR Besitran OR Sealdin OR Gladem OR Sulpiride OR Sulperide OR Arminol OR Deponerton OR Meresa OR Desisulpid OR Digton OR Dogmatil OR Dolmatil OR Eglonyl OR Ekilid OR Guastil OR Lebopride OR Neogama OR Pontiride OR Psicocen OR Sulp OR Sulpitil OR Sulpivert OR Sulpor OR Synedil OR Tepavil OR Aiglonyl OR Temazepam OR Hydroxydiazepam OR Methyloxazepam OR Signopam OR Tenox OR "WY 3917" OR WY3917 OR Dasuen OR Euhypnos OR Levanxol OR "Norkotral Tema" OR Normison OR Nocturne OR Temtabs OR Normitab OR Nortem OR Planum OR "Pronervon T" OR Remestan OR Restoril OR "Ro 5 5345" OR Ro55345 OR "SaH 47 603" OR "SaH 47603" OR Temaze OR "Temazep von CT" OR Thioridazine OR ApoThioridazine OR Meleril OR Melleril OR Melleryl OR Mellaril OR Melleretten OR Melzine OR Thiozine OR Sonapax OR Thioridazineneurazpharm OR Aldazine OR Rideril OR Thiothixene OR Tiotixene OR Navane OR Topiramate OR USL255 OR "McN 4853" OR Topamax OR Epitomax OR Tranylcypromine OR "Trans 2 Phenylcyclopropylamine" OR Jatrosom OR Transamine OR Parnate OR Trazodone OR Tradozone OR "AF 1161" OR AF1161 OR Deprax OR Desyrel OR Molipaxin OR Trittico OR Thombran OR "Trazodon Hexal" OR "Trazodon Neuraxpharm" OR Trazon OR Triazolam OR "U 33,030" OR "U33,030" OR Halcion OR Trilam OR "Apo Triazo" OR Trifluoperazine OR Trifluoroperazine OR Trifluperazine OR Eskazine OR Flupazine OR Terfluzine OR Triftazin OR Stelazine OR Trimipramine OR Trimeprimine OR Herphonal OR Trimineurin OR NovoTripramine OR Rhotrimine OR Stangyl OR Surmontil OR Trimidura OR Trimineurin OR Trimipramin OR "Apo Trimip" OR ApoTrimip OR Eldoral OR Venlafaxine OR "Wy 45030" OR Wy45030 OR "Wy 45,030" OR "Wy45,030" OR Effexor OR Trevilor OR Vandral OR Efexor OR Dobupal OR Vigabatrin OR "Gamma Vinyl GABA" OR "Gamma Vinyl Gamma Aminobutyric Acid" OR Sabril OR Sabrilex OR Vortioxetine OR Brintellix OR "Lu AA21004" OR LuAA21004 OR Zaleplon OR "SKP 1041" OR Sonata OR Zelepion OR Starnoc OR "CL 284,846" OR "CL284,846" OR "CL 284846" OR "L 846" OR Ziprasidone OR Ziprazidone OR "CP 88,059" OR "CP 88059" OR Zolpidem OR Amsic OR Bikalm OR Dalparan OR "SL 80.0750" OR "SL 800750 23 N" OR Stilnoct OR Stilnox OR Zodormdura OR Zoldem OR Zolirin OR "Zolpi Lich" OR Zolpinox OR Zolpimist OR Ambien OR Zopiclone OR Zop OR Zopicalma OR Zopiclodura OR Zopiclon OR Zopitan OR Zorclone OR Imovane OR Ximovan OR Zimovane OR Limovan OR Optidorm OR Rhovane OR "RP 27 267" OR Siaten OR Somnosan OR Zileze OR Zimoclone OR "Zopi Puren" OR Zopicalm OR Zotepine OR Zoleptil OR Nipolept OR Zuclopenthixol OR Zuclopentixol OR Clopixol OR Zuclopenthixole OR Acuphase):ti,ab) AND ([mh Depression] or [mh ^"Mood Disorders"] or [mh ^"Depressive Disorder"] or [mh "Depressive Disorder, Major"] or [mh "Dysthymic Disorder"] or [mh "Depressive Disorder, Treatment‐Resistant"] or [mh "Affective Disorders, Psychotic"] or [mh "Cyclothymic Disorder"] or ("Affective Disorder" or "Affective Disorders" or "Affective Psychoses" or "Affective Psychosis" or Cyclothymic or Depress* or Dysthym* or "Mood Disorder" or "Mood Disorders"):ti,ab)

with Publication Year from 2018 to present, in Trials

**D. Embase 1980 to 2020 Week 18**

1. Brain Concussion/ OR Brain Injury/ OR Diffuse Axonal Injury/ OR Head Injury/ OR Pneumocephalus/ OR Traumatic Brain Injury/ OR (((Brain OR Cerebr$ OR Crani$ OR Crushing Skull OR Diffuse Axonal OR Head OR Hemisphere?) adj1 (Injur$ OR Trauma$)) OR ((Cerebr$ OR Crani$ OR Head) adj (Lesion? OR Wound?)) OR ((Posttraumatic OR Traumatic) adj Encephalopath$) OR (Traumatic adj (Brain OR Cerebr$)) OR Concuss$ OR DAI OR DAIs OR Pneumocephalus OR TBI OR TBIs).ti,ab.
2. Anxiolytic Agent/ OR Anticonvulsive Agent/ OR Antidepressant Agent/ OR Neuroleptic Agent/ OR Benzodiazepine Derivative/ OR Beta Adrenergic Receptor Blocking Agent/ OR Agomelatine/ OR Alprazolam/ OR Amisulpride/ OR Amitriptyline/ OR Amoxapine/ OR Aripiprazole/ OR Atenolol/ OR Atomoxetine/ OR Benztropine/ OR Brexpiprazole/ OR Bromazepam/ OR Bupropion/ OR Buspirone/ OR Carbamazepine/ OR Cariprazine/ OR Chlordiazepoxide/ OR Chlorpromazine/ OR Citalopram/ OR Clobazam/ OR Clomipramine/ OR Clonazepam/ OR Clopenthixol/ OR Clorazepate/ OR Clozapine/ OR Desipramine/ OR Desvenlafaxine/ OR Dexamphetamine/ OR Diazepam/ OR Domperidone/ OR Dosulepin/ OR Doxepin/ OR Droperidol/ OR Duloxetine/ OR Estazolam/ OR Eszopiclone/ OR Flunitrazepam/ OR Fluoxetine/ OR Flupentixol/ OR Fluphenazine/ OR Fluvoxamine/ OR Guanfacine/ OR Haloperidol/ OR Iloperidone/ OR Imipramine/ OR Isocarboxazid/ OR Lamotrigine/ OR Levomepromazine/ OR Lithium/ OR Lithium Carbonate/ OR Lorazepam/ OR Loxapine/ OR Lurasidone/ OR Methylphenidate/ OR Mianserin/ OR Midazolam/ OR Milnacipran/ OR Mirtazapine/ OR Moclobemide/ OR Modafinil/ OR Molindone/ OR Nefazodone/ OR Nitrazepam/ OR Nortriptyline/ OR Olanzapine/ OR Oxazepam/ OR Oxcarbazepine/ OR Paliperidone/ OR Paroxetine/ OR Periciazine/ OR Phenelzine/ OR Phenobarbital/ OR Pindolol/ OR Prazepam/ OR Pregabalin/ OR Prochlorperazine/ OR Promazine/ OR Promethazine/ OR Propranolol/ OR Protriptyline/ OR Quetiapine/ OR Reboxetine/ OR Remoxipride/ OR Risperidone/ OR Selegiline/ OR Sertindole/ OR Sertraline/ OR Sulpiride/ OR Temazepam/ OR Thioridazine/ OR Tiotixene/ OR Topiramate/ OR Tranylcypromine/ OR Trazodone/ OR Triazolam/ OR Trifluoperazine/ OR Trimipramine/ OR Valproic Acid/ OR Venlafaxine/ OR Vigabatrin/ OR Vortioxetine/ OR Zaleplon/ OR Ziprasidone/ OR Zolpidem/ OR Zopiclone/ OR Zotepine/ OR Zuclopenthixol/ OR Zuclopenthixol Acetate/ OR Zuclopenthixol Decanoate/ OR ((Adrenergic adj Beta adj2 (Antagonist OR Block$)) OR Anti Anxiety OR Anti Convuls$ OR Anti Depress$ OR Anti Epilep$ OR Anti Psychotic? OR Antianxiety OR Anticonvuls$ OR Antidepress$ OR Antiepilep$ OR Antipsychotic$ OR Anxiolytic$ OR Benzodiazepine$ OR (Beta adj Block$) OR (Beta adj1 Adrenergic adj2 Block$) OR Thymoanaleptic$ OR Thymoleptic$ OR Agomelatine OR "S 20098" OR S20098 OR Thymanax OR Valdoxan OR "AGO 178" OR AGO178 OR Alprazolam OR Alprazolan OR "Apo Alpraz" OR ApoAlpraz OR Cassadan OR "D 65MT" OR D65MT OR Xanax OR Tafil OR Trankimazin OR "Novo Alprazol" OR NovoAlprazol OR "Nu Alpraz" OR NuAlpraz OR Ralozam OR "U-31,889" OR "U31,889" OR Alprox OR Esparon OR Kalma OR Amisulpride OR Sultopride OR Barnetil OR "DAN 2163" OR Solian OR "LIN 1418" OR Amitriptyline OR Amineurin OR Amitrip OR Amitriptylin OR Amitrol OR Tryptine OR ApoAmitriptyline OR Damilen OR Domical OR Laroxyl OR Endep OR Lentizol OR Novoprotect OR Saroten OR Sarotex OR Syneudon OR Triptafen OR Tryptizol OR Tryptanol OR Elavil OR Anapsique OR Amoxapine OR Desmethylloxapine OR "CL 67,772" OR "CL67,772" OR Demolox OR Asendin OR Defanyl OR Asendis OR Aripiprazole OR Aripiprazol OR "OPC 14597" OR Abilify OR Asenapine OR Saphris OR "OR G 5222" OR Atenolol OR Tenormine OR Tenormin OR "ICI 66082" OR ICI66082 OR Atomoxetine OR Tomoxetine OR Strattera OR "LY 139603" OR Benztropine OR Benzatropine OR Bensylate OR PMSBenztropine OR Cogentin OR Cogentinol OR Methylbenztropine OR ApoBenztropine OR Brexpiprazole OR Bromazepam OR BromaLich OR "Bromaz 1A Pharma" OR Bromazanil OR "Bromazep von CT" OR Durazanil OR Lexotan OR Lexotanil OR Lexatin OR Lexomil OR "Ro 5-3350" OR "Ro 53350" OR Anxyrex OR Bupropion OR Amfebutamone OR Zyntabac OR Quomen OR Wellbutrin OR Zyban OR Buspirone OR "MJ 9022 1" OR MJ90221 OR Neurosine OR Busp OR Anxut OR Buspar OR Bespar OR Carbamazepine OR Tegretol OR Carbazepin OR Epitol OR Finlepsin OR Neurotol OR Amizepine OR Cariprazine OR "RGH 188" OR Chlordiazepoxide OR Methaminodiazepoxide OR Librium OR Chlozepid OR Elenium OR Chlorpromazine OR Thorazine OR Aminazine OR Largactil OR Chlordelazine OR Contomin OR Fenactil OR Propaphenin OR Chlorazine OR Citalopram OR Cytalopram OR "Lu 10 171" OR Lu10171 OR Escitalopram OR Lexapro OR Clobazam OR "HR 376" OR Onfi OR "LM 2717" OR Frisium OR Urbanyl OR Clomipramine OR Chlomipramine OR Chlorimipramine OR Hydiphen OR Anafranil OR Clonazepam OR "Ro 5 4023" OR "Ro 54023" OR Antelepsin OR Rivotril OR Clopenthixol OR Zuclopenthixol OR Cisordinol OR Clorazepate OR Chlorazepate OR Tranxene OR Tranxilium OR "4306 CB" OR Clozapine OR Clozaril OR Leponex OR Desipramine OR Desmethylimipramine OR Demethylimipramine OR Norpramin OR Pertofrane OR Pertrofran OR Pertofran OR Petylyl OR Desvenlafaxine OR "O Desmethylvenlafaxine" OR "WY 45,233" OR "WY 45,233" OR "WY45,233" OR "WY 45233" OR WY45233 OR Pristiq OR Dextroamphetamine OR Dexamphetamine OR Dexamfetamine OR "Dextro Amphetamine" OR "D Amphetamine" OR Dexedrine OR DextroStat OR Oxydess OR Diazepam OR Diazemuls OR Faustan OR Valium OR Seduxen OR Sibazon OR Stesolid OR Apaurin OR Relanium OR "Valproic Acid" OR Divalproex OR "Propylisopropylacetic Acid" OR "2 Propylpentanoic Acid" OR Convulsofin OR Depakene OR Depakine OR Depakote OR Vupral OR Valproate OR Ergenyl OR "Dipropyl Acetate" OR Domperidone OR Domperidon OR Domidon OR Gastrocure OR Motilium OR Nauzelin OR Peridys OR "R 33,812" OR "R33,812" OR "R 33812" OR R33812 OR Dothiepin OR Dosulepin OR Prothiaden OR Doxepin OR Deptran OR Desidox OR Doneurin OR Doxepia OR Espadox OR Mareen OR Prudoxin OR Quitaxon OR Sinequan OR Sinquan OR Zonalon OR Xepin OR Aponal OR ApoDoxepin OR Droperidol OR Inapsine OR Dehidrobenzperidol OR Dehydrobenzperidol OR Droleptan OR Duloxetine OR "LY 248686" OR LY248686 OR "LY 227942" OR LY227942 OR Cymbalta OR Estazolam OR Tasedan OR ProSom OR "D 40TA" OR D40TA OR Nuctalon OR Eszopiclone OR Lunesta OR Estorra OR Flunitrazepam OR Fluridrazepam OR Flunibeta OR Flunimerck OR Fluninoc OR Rohypnol OR Rohipnol OR Narcozep OR "Flunizep von CT" OR "RO 5 4200" OR RO54200 OR Fluoxetine OR Fluoxetin OR "Lilly 110140" OR Lilly110140 OR Sarafem OR Prozac OR Flupenthixol OR Flupentixol OR Emergil OR Fluanxol OR Fluphenazine OR Flufenazin OR Lyogen OR Prolixin OR Fluvoxamine OR Fluvoxadura OR Fluvoxamin OR Fluvoxamina OR Luvox OR Fevarin OR Floxyfral OR Dumirox OR Faverin OR Desiflu OR "DU 23000" OR DU23000 OR Guanfacine OR Tenex OR Lon798 OR "BS 100 141" OR BS100141 OR Estulic OR Haloperidol OR Haldol OR Iloperidone OR Zomaril OR Fanapt OR "HP 873" OR Imipramine OR Imizin OR Norchlorimipramine OR Imidobenzyle OR Tofranil OR Melipramine OR Pryleugan OR Janimine OR Isocarboxazid OR Lamotrigine OR Crisomet OR Lamictal OR Lamiktal OR "BW 430C" OR Labileno OR Methotrimeprazine OR Levomepromazine OR Levopromazine OR Levomeprazin OR Tisercin OR Tizercine OR Tizertsin OR Lithium OR Dilithium OR Lithane OR Lithobid OR Lithonate OR "CP-15,467 61" OR "CP15,46761" OR Micalith OR "NSC 16895" OR NSC16895 OR Priadel OR "Quilinorm Retard" OR Quilinormretard OR Eskalith OR Lithotabs OR Lorazepam OR Ativan OR Temesta OR "Orfidal Wyeth" OR Donix OR Duralozam OR Durazolam OR Idalprem OR Laubeel OR "Lorazep von CT" OR "Novo Lorazem" OR NovoLorazem OR "Nu Loraz" OR NuLoraz OR Sedicepan OR Sinestron OR Somagerol OR Tolid OR "WY 4036" OR WY4036 OR ApoLorazepam OR Loxapine OR Cloxazepine OR Oxilapine OR Loxitane OR Loxipine OR Loxapinsuccinate OR "CL 71,563" OR "CL71,563" OR Lurasidone OR "SM 13496" OR SM13496 OR "SM-13,496" OR "SM13,496" OR Latuda OR Methylphenidate OR Metadate OR Equasym OR Methylin OR Concerta OR Phenidylate OR Ritalin OR Ritaline OR Tsentedrin OR Centedrin OR Daytrana OR Mianserin OR Tolvon OR Lerivon OR Org GB 94 OR Midazolam OR Dormicum OR Versed OR "Ro 21 3981" OR "Ro 213981" OR Milnacipran OR Midalcipran OR Levomilnacipran OR Savella OR "F 2207" OR Ixel OR Mirtazapine OR "6 Azamianserin" OR Esmirtazapine OR Remeron OR Remergil OR Zispin OR Norset OR Rexer OR "Org 50081" OR " OR G 3770" OR Moclobemide OR Moclobamide OR Arima OR Aurorix OR Manerix OR Moclamine OR Aurorex OR Deprenorm OR Feraken OR Moclobemid OR Moclobeta OR Moclodura OR Moclonorm OR Rimoc OR "Ro 11 1163" OR Modafinil OR Benzhydrylsulfinylacetamide OR "CRL 40476" OR Vigil OR Provigil OR Sparlon OR Alertec OR Modiodal OR Molindone OR Moban OR Nefazodone OR Rulivan OR Serzone OR Dutonin OR Nefadar OR Menfazona OR Nitrazepam OR Nitrodiazepam OR "Dormo Puren" OR Eatan OR Imadorm OR Imeson OR Mogadon OR Nitrazadon OR Nitrazep OR Novanox OR Radedorm OR Remnos OR Serenade OR Somnite OR Alodorm OR Dormalon OR Nortriptyline OR Desmethylamitriptylin OR Desitriptyline OR Aventyl OR Paxtibi OR Allegron OR Norfenazin OR Pamelor OR Nortrilen OR Olanzapine OR Zolafren OR "LY 170052" OR Zyprexa OR "LY 170053" OR Oxazepam OR Serax OR Tazepam OR Adumbran OR Oxcarbazepine OR Timox OR Trileptal OR "GP 47680" OR Paliperidone OR "9 OH Risperidone" OR "9 Hydroxy Risperidone" OR "9 Hydroxyrisperidone" OR Invega OR "R 76477" OR R76477 OR Paroxetine OR "BRL 29060" OR BRL29060 OR "FG 7051" OR FG7051 OR Seroxat OR Paxil OR Aropax OR Periciazine OR Propericiazine OR Pericyazine OR Neuleptil OR Neuleptyl OR Aolept OR Phenelzine OR "Beta Phenylethylhydrazine" OR "2 Phenethylhydrazine" OR Fenelzin OR Phenethylhydrazine OR Nardelzine OR Nardil OR Phenobarbital OR Phenobarbitone OR "Phenylethylbarbituric Acid" OR Phenemal OR Phenylbarbital OR Hysteps OR Luminal OR Gardenal OR Pindolol OR Prindolol OR Visken OR "LB 46" OR LB46 OR Prazepam OR Lysanxia OR Reapam OR Centrax OR Demetrin OR Pregabalin OR "3 Isobutyl GABA" OR Lyrica OR "CI 1008" OR CI1008 OR Prochlorperazine OR Compazine OR Promazine OR Sparine OR Sinophenin OR Protactyl OR Promethazine OR Prometazin OR Proazamine OR Rumergan OR Diprazin OR Phenergan OR Phenargan OR Phensedyl OR Pipolfen OR Pipolphen OR Promet OR Prothazin OR Pyrethia OR Remsed OR Atosil OR Diphergan OR Propranolol OR Propanolol OR Inderal OR Avlocardyl OR "AY 20694" OR AY20694 OR Rexigen OR Dexpropranolol OR Dociton OR Obsidan OR Obzidan OR Anaprilin OR Anapriline OR Betadren OR Protriptyline OR Vivactil OR Quetiapine OR "ICI 204,636" OR "ICI 204636" OR ICI204636 OR Seroquel OR Reboxetine OR Vestra OR Remoxipride OR "FLA 731" OR FLA731 OR Risperidone OR Risperdal OR Risperidal OR "R 64,766" OR "R64,766" OR "R 64766" OR R64766 OR Selegiline OR Selegyline OR "L Deprenyl" OR "E 250" OR E250 OR Eldepryl OR Emsam OR Zelapar OR Deprenil OR Deprenalin OR Yumex OR Jumex OR Humex OR Deprenyl OR Sertindole OR Serlect OR "Lu 23 174" OR Serdolect OR Sertraline OR Zoloft OR Altruline OR Lustral OR Aremis OR Besitran OR Sealdin OR Gladem OR Sulpiride OR Sulperide OR Arminol OR Deponerton OR Meresa OR Desisulpid OR Digton OR Dogmatil OR Dolmatil OR Eglonyl OR Ekilid OR Guastil OR Lebopride OR Neogama OR Pontiride OR Psicocen OR Sulp OR Sulpitil OR Sulpivert OR Sulpor OR Synedil OR Tepavil OR Aiglonyl OR Temazepam OR Hydroxydiazepam OR Methyloxazepam OR Signopam OR Tenox OR "WY 3917" OR WY3917 OR Dasuen OR Euhypnos OR Levanxol OR "Norkotral Tema" OR Normison OR Nocturne OR Temtabs OR Normitab OR Nortem OR Planum OR "Pronervon T" OR Remestan OR Restoril OR "Ro 5 5345" OR Ro55345 OR "SaH 47 603" OR "SaH 47603" OR Temaze OR "Temazep von CT" OR Thioridazine OR ApoThioridazine OR Meleril OR Melleril OR Melleryl OR Mellaril OR Melleretten OR Melzine OR Thiozine OR Sonapax OR Thioridazineneurazpharm OR Aldazine OR Rideril OR Thiothixene OR Tiotixene OR Navane OR Topiramate OR USL255 OR "McN 4853" OR Topamax OR Epitomax OR Tranylcypromine OR "Trans 2 Phenylcyclopropylamine" OR Jatrosom OR Transamine OR Parnate OR Trazodone OR Tradozone OR "AF 1161" OR AF1161 OR Deprax OR Desyrel OR Molipaxin OR Trittico OR Thombran OR "Trazodon Hexal" OR "Trazodon Neuraxpharm" OR Trazon OR Triazolam OR "U 33,030" OR "U33,030" OR Halcion OR Trilam OR "Apo Triazo" OR Trifluoperazine OR Trifluoroperazine OR Trifluperazine OR Eskazine OR Flupazine OR Terfluzine OR Triftazin OR Stelazine OR Trimipramine OR Trimeprimine OR Herphonal OR Trimineurin OR NovoTripramine OR Rhotrimine OR Stangyl OR Surmontil OR Trimidura OR Trimineurin OR Trimipramin OR "Apo Trimip" OR ApoTrimip OR Eldoral OR Venlafaxine OR "Wy 45030" OR Wy45030 OR "Wy 45,030" OR "Wy45,030" OR Effexor OR Trevilor OR Vandral OR Efexor OR Dobupal OR Vigabatrin OR "Gamma Vinyl GABA" OR "Gamma Vinyl Gamma Aminobutyric Acid" OR Sabril OR Sabrilex OR Vortioxetine OR Brintellix OR "Lu AA21004" OR LuAA21004 OR Zaleplon OR "SKP 1041" OR Sonata OR Zelepion OR Starnoc OR "CL 284,846" OR "CL284,846" OR "CL 284846" OR "L 846" OR Ziprasidone OR Ziprazidone OR "CP 88,059" OR "CP 88059" OR Zolpidem OR Amsic OR Bikalm OR Dalparan OR "SL 80.0750" OR "SL 800750 23 N" OR Stilnoct OR Stilnox OR Zodormdura OR Zoldem OR Zolirin OR "Zolpi Lich" OR Zolpinox OR Zolpimist OR Ambien OR Zopiclone OR Zop OR Zopicalma OR Zopiclodura OR Zopiclon OR Zopitan OR Zorclone OR Imovane OR Ximovan OR Zimovane OR Limovan OR Optidorm OR Rhovane OR "RP 27 267" OR Siaten OR Somnosan OR Zileze OR Zimoclone OR "Zopi Puren" OR Zopicalm OR Zotepine OR Zoleptil OR Nipolept OR Zuclopenthixol OR Zuclopentixol OR Clopixol OR Zuclopenthixole OR Acuphase).ti,ab.
3. 1 AND 2
4. Exp Animals/ OR Exp Invertebrate/ OR Animal Experiment/ OR Animal Model/ OR Animal Tissue/ OR Animal Cell/ OR Nonhuman/
5. Human/ OR Normal Human/ OR Human Cell/
6. 4 AND 5
7. 4 NOT 6
8. 3 NOT 7
9. Limit 8 to MEDLINE
10. 8 NOT 9
11. Limit 10 to English Language
12. Depression/ or Adolescent Depression/ or Agitated Depression/ or Atypical Depression/ or Chronic Depression/ or Depressive Psychosis/ or Dysthymia/ or Major Depression/ or Minor Depression/ or "Mixed Anxiety and Depression"/ or "Mixed Depression and Dementia"/ or "Mixed Mania and Depression"/ or Reactive Depression/ or Recurrent Brief Depression/ or Subsyndromal Depression/ or Treatment Resistant Depression/ or Cyclothymia/ or Exp Depression Assessment/ or Mood Disorder/ or Affective Psychosis/ or Major Affective Disorder/ or Minor Affective Disorder/ or ("Affective Disorder" or "Affective Disorders" or "Affective Psychoses" or "Affective Psychosis" or Cyclothymic or Depress* or Dysthym* or "Mood Disorder" or "Mood Disorders").ti,ab.
13. (201803* or 201804* or 201805* or 201806* or 201807* or 201808* or 201809* or 201810* or 201811* or 201812* or 2019* or 202*).ce,cs,dc,dd. or (Mar 2018 or Apr 2018 or May 2018 or Jun 2018 or Jul 2018 or Aug 2018 or Sep 2018 or Oct 2018 or Nov 2018 or Dec 2018 or 2018* or Jan 2019 or Feb 2019 or Mar 2019 or Apr 2019 or May 2019 or Jun 2019 or Jul 2019 or Aug 2019 or Sep 2019 or Oct 2019 or Nov 2019 or Dec 2019 or 2019* or Jan 202* or Feb 202* or Mar 202* or Apr 202* or May 202* or 202*).dp.
14. 11 AND 12 AND 13

**E. Ovid MEDLINE(R) ALL 1946 to May 01, 2020**

1. Exp Brain Hemorrhage, Traumatic/ OR Brain Injuries/ OR Brain Injury, Chronic/ OR Cerebral Hemorrhage, Traumatic/ OR Cerebrovascular Trauma/ OR Craniocerebral Trauma/ OR Diffuse Axonal Injury/ OR Exp Head Injuries, Closed/ OR Head Injuries, Penetrating/ OR Exp Intracranial Hemorrhage, Traumatic/ OR Exp Pneumocephalus/ OR (((Brain OR Cerebr$ OR Crani$ OR Crushing Skull OR Diffuse Axonal OR Head OR Hemisphere?) adj1 (Injur$ OR Trauma$)) OR ((Cerebr$ OR Crani$ OR Head) adj (Lesion? OR Wound?)) OR ((Posttraumatic OR Traumatic) adj Encephalopath$) OR (Traumatic adj (Brain OR Cerebr$)) OR Concuss$ OR DAI OR DAIs OR Pneumocephalus OR TBI OR TBIs).ti,ab.
2. "Anti-Anxiety Agents"/ OR "Anticonvulsants"/ OR "Antidepressive Agents"/ OR "Antipsychotic Agents"/ OR "Benzodiazepines"/ OR "Adrenergic Beta-Antagonists"/ OR Alprazolam/ OR Amitriptyline/ OR Amoxapine/ OR Aripiprazole/ OR Atenolol/ OR Atomoxetine Hydrochloride/ OR Benztropine/ OR Bromazepam/ OR Bupropion/ OR Buspirone/ OR Carbamazepine/ OR Chlordiazepoxide/ OR Chlorpromazine/ OR Citalopram/ OR Clomipramine/ OR Clonazepam/ OR Clopenthixol/ OR Clorazepate Dipotassium/ OR Clozapine/ OR Desipramine/ OR Desvenlafaxine Succinate/ OR Dextroamphetamine/ OR Diazepam/ OR Valproic Acid/ OR Domperidone/ OR Dothiepin/ OR Doxepin/ OR Droperidol/ OR Duloxetine Hydrochloride/ OR Estazolam/ OR Eszopiclone/ OR Flunitrazepam/ OR Fluoxetine/ OR Flupenthixol/ OR Fluphenazine/ OR Fluvoxamine/ OR Guanfacine/ OR Haloperidol/ OR Imipramine/ OR Isocarboxazid/ OR Methotrimeprazine/ OR Lithium/ OR Lithium Carbonate/ OR Lorazepam/ OR Loxapine/ OR Lurasidone Hydrochloride/ OR Methylphenidate/ OR Mianserin/ OR Midazolam/ OR Moclobemide/ OR Molindone/ OR Nitrazepam/ OR Nortriptyline/ OR Oxazepam/ OR Paliperidone Palmitate/ OR Paroxetine/ OR Phenelzine/ OR Phenobarbital/ OR Pindolol/ OR Prazepam/ OR Pregabalin/ OR Prochlorperazine/ OR Promazine/ OR Promethazine/ OR Propranolol/ OR Protriptyline/ OR Quetiapine Fumarate/ OR Remoxipride/ OR Risperidone/ OR Selegiline/ OR Sertraline/ OR Sulpiride/ OR Temazepam/ OR Thioridazine/ OR Thiothixene/ OR Tranylcypromine/ OR Trazodone/ OR Triazolam/ OR Trifluoperazine/ OR Trimipramine/ OR Venlafaxine Hydrochloride/ OR Vigabatrin/ OR ((Adrenergic adj Beta adj2 (Antagonist OR Block$)) OR Anti Anxiety OR Anti Convuls$ OR Anti Depress$ OR Anti Epilep$ OR Anti Psychotic? OR Antianxiety OR Anticonvuls$ OR Antidepress$ OR Antiepilep$ OR Antipsychotic$ OR Anxiolytic$ OR Benzodiazepine$ OR (Beta adj Block$) OR (Beta adj1 Adrenergic adj2 Block$) OR Thymoanaleptic$ OR Thymoleptic$ OR Agomelatine OR "S 20098" OR S20098 OR Thymanax OR Valdoxan OR "AGO 178" OR AGO178 OR Alprazolam OR Alprazolan OR "Apo Alpraz" OR ApoAlpraz OR Cassadan OR "D 65MT" OR D65MT OR Xanax OR Tafil OR Trankimazin OR "Novo Alprazol" OR NovoAlprazol OR "Nu Alpraz" OR NuAlpraz OR Ralozam OR "U-31,889" OR "U31,889" OR Alprox OR Esparon OR Kalma OR Amisulpride OR Sultopride OR Barnetil OR "DAN 2163" OR Solian OR "LIN 1418" OR Amitriptyline OR Amineurin OR Amitrip OR Amitriptylin OR Amitrol OR Tryptine OR ApoAmitriptyline OR Damilen OR Domical OR Laroxyl OR Endep OR Lentizol OR Novoprotect OR Saroten OR Sarotex OR Syneudon OR Triptafen OR Tryptizol OR Tryptanol OR Elavil OR Anapsique OR Amoxapine OR Desmethylloxapine OR "CL 67,772" OR "CL67,772" OR Demolox OR Asendin OR Defanyl OR Asendis OR Aripiprazole OR Aripiprazol OR "OPC 14597" OR Abilify OR Asenapine OR Saphris OR "OR G 5222" OR Atenolol OR Tenormine OR Tenormin OR "ICI 66082" OR ICI66082 OR Atomoxetine OR Tomoxetine OR Strattera OR "LY 139603" OR Benztropine OR Benzatropine OR Bensylate OR PMSBenztropine OR Cogentin OR Cogentinol OR Methylbenztropine OR ApoBenztropine OR Brexpiprazole OR Bromazepam OR BromaLich OR "Bromaz 1A Pharma" OR Bromazanil OR "Bromazep von CT" OR Durazanil OR Lexotan OR Lexotanil OR Lexatin OR Lexomil OR "Ro 5-3350" OR "Ro 53350" OR Anxyrex OR Bupropion OR Amfebutamone OR Zyntabac OR Quomen OR Wellbutrin OR Zyban OR Buspirone OR "MJ 9022 1" OR MJ90221 OR Neurosine OR Busp OR Anxut OR Buspar OR Bespar OR Carbamazepine OR Tegretol OR Carbazepin OR Epitol OR Finlepsin OR Neurotol OR Amizepine OR Cariprazine OR "RGH 188" OR Chlordiazepoxide OR Methaminodiazepoxide OR Librium OR Chlozepid OR Elenium OR Chlorpromazine OR Thorazine OR Aminazine OR Largactil OR Chlordelazine OR Contomin OR Fenactil OR Propaphenin OR Chlorazine OR Citalopram OR Cytalopram OR "Lu 10 171" OR Lu10171 OR Escitalopram OR Lexapro OR Clobazam OR "HR 376" OR Onfi OR "LM 2717" OR Frisium OR Urbanyl OR Clomipramine OR Chlomipramine OR Chlorimipramine OR Hydiphen OR Anafranil OR Clonazepam OR "Ro 5 4023" OR "Ro 54023" OR Antelepsin OR Rivotril OR Clopenthixol OR Zuclopenthixol OR Cisordinol OR Clorazepate OR Chlorazepate OR Tranxene OR Tranxilium OR "4306 CB" OR Clozapine OR Clozaril OR Leponex OR Desipramine OR Desmethylimipramine OR Demethylimipramine OR Norpramin OR Pertofrane OR Pertrofran OR Pertofran OR Petylyl OR Desvenlafaxine OR "O Desmethylvenlafaxine" OR "WY 45,233" OR "WY 45,233" OR "WY45,233" OR "WY 45233" OR WY45233 OR Pristiq OR Dextroamphetamine OR Dexamphetamine OR Dexamfetamine OR "Dextro Amphetamine" OR "D Amphetamine" OR Dexedrine OR DextroStat OR Oxydess OR Diazepam OR Diazemuls OR Faustan OR Valium OR Seduxen OR Sibazon OR Stesolid OR Apaurin OR Relanium OR "Valproic Acid" OR Divalproex OR "Propylisopropylacetic Acid" OR "2 Propylpentanoic Acid" OR Convulsofin OR Depakene OR Depakine OR Depakote OR Vupral OR Valproate OR Ergenyl OR "Dipropyl Acetate" OR Domperidone OR Domperidon OR Domidon OR Gastrocure OR Motilium OR Nauzelin OR Peridys OR "R 33,812" OR "R33,812" OR "R 33812" OR R33812 OR Dothiepin OR Dosulepin OR Prothiaden OR Doxepin OR Deptran OR Desidox OR Doneurin OR Doxepia OR Espadox OR Mareen OR Prudoxin OR Quitaxon OR Sinequan OR Sinquan OR Zonalon OR Xepin OR Aponal OR ApoDoxepin OR Droperidol OR Inapsine OR Dehidrobenzperidol OR Dehydrobenzperidol OR Droleptan OR Duloxetine OR "LY 248686" OR LY248686 OR "LY 227942" OR LY227942 OR Cymbalta OR Estazolam OR Tasedan OR ProSom OR "D 40TA" OR D40TA OR Nuctalon OR Eszopiclone OR Lunesta OR Estorra OR Flunitrazepam OR Fluridrazepam OR Flunibeta OR Flunimerck OR Fluninoc OR Rohypnol OR Rohipnol OR Narcozep OR "Flunizep von CT" OR "RO 5 4200" OR RO54200 OR Fluoxetine OR Fluoxetin OR "Lilly 110140" OR Lilly110140 OR Sarafem OR Prozac OR Flupenthixol OR Flupentixol OR Emergil OR Fluanxol OR Fluphenazine OR Flufenazin OR Lyogen OR Prolixin OR Fluvoxamine OR Fluvoxadura OR Fluvoxamin OR Fluvoxamina OR Luvox OR Fevarin OR Floxyfral OR Dumirox OR Faverin OR Desiflu OR "DU 23000" OR DU23000 OR Guanfacine OR Tenex OR Lon798 OR "BS 100 141" OR BS100141 OR Estulic OR Haloperidol OR Haldol OR Iloperidone OR Zomaril OR Fanapt OR "HP 873" OR Imipramine OR Imizin OR Norchlorimipramine OR Imidobenzyle OR Tofranil OR Melipramine OR Pryleugan OR Janimine OR Isocarboxazid OR Lamotrigine OR Crisomet OR Lamictal OR Lamiktal OR "BW 430C" OR Labileno OR Methotrimeprazine OR Levomepromazine OR Levopromazine OR Levomeprazin OR Tisercin OR Tizercine OR Tizertsin OR Lithium OR Dilithium OR Lithane OR Lithobid OR Lithonate OR "CP-15,467 61" OR "CP15,46761" OR Micalith OR "NSC 16895" OR NSC16895 OR Priadel OR "Quilinorm Retard" OR Quilinormretard OR Eskalith OR Lithotabs OR Lorazepam OR Ativan OR Temesta OR "Orfidal Wyeth" OR Donix OR Duralozam OR Durazolam OR Idalprem OR Laubeel OR "Lorazep von CT" OR "Novo Lorazem" OR NovoLorazem OR "Nu Loraz" OR NuLoraz OR Sedicepan OR Sinestron OR Somagerol OR Tolid OR "WY 4036" OR WY4036 OR ApoLorazepam OR Loxapine OR Cloxazepine OR Oxilapine OR Loxitane OR Loxipine OR Loxapinsuccinate OR "CL 71,563" OR "CL71,563" OR Lurasidone OR "SM 13496" OR SM13496 OR "SM-13,496" OR "SM13,496" OR Latuda OR Methylphenidate OR Metadate OR Equasym OR Methylin OR Concerta OR Phenidylate OR Ritalin OR Ritaline OR Tsentedrin OR Centedrin OR Daytrana OR Mianserin OR Tolvon OR Lerivon OR "Org GB 94" OR Midazolam OR Dormicum OR Versed OR "Ro 21 3981" OR "Ro 213981" OR Milnacipran OR Midalcipran OR Levomilnacipran OR Savella OR "F 2207" OR Ixel OR Mirtazapine OR "6 Azamianserin" OR Esmirtazapine OR Remeron OR Remergil OR Zispin OR Norset OR Rexer OR "Org 50081" OR " OR G 3770" OR Moclobemide OR Moclobamide OR Arima OR Aurorix OR Manerix OR Moclamine OR Aurorex OR Deprenorm OR Feraken OR Moclobemid OR Moclobeta OR Moclodura OR Moclonorm OR Rimoc OR "Ro 11 1163" OR Modafinil OR Benzhydrylsulfinylacetamide OR "CRL 40476" OR Vigil OR Provigil OR Sparlon OR Alertec OR Modiodal OR Molindone OR Moban OR Nefazodone OR Rulivan OR Serzone OR Dutonin OR Nefadar OR Menfazona OR Nitrazepam OR Nitrodiazepam OR "Dormo Puren" OR Eatan OR Imadorm OR Imeson OR Mogadon OR Nitrazadon OR Nitrazep OR Novanox OR Radedorm OR Remnos OR Serenade OR Somnite OR Alodorm OR Dormalon OR Nortriptyline OR Desmethylamitriptylin OR Desitriptyline OR Aventyl OR Paxtibi OR Allegron OR Norfenazin OR Pamelor OR Nortrilen OR Olanzapine OR Zolafren OR "LY 170052" OR Zyprexa OR "LY 170053" OR Oxazepam OR Serax OR Tazepam OR Adumbran OR Oxcarbazepine OR Timox OR Trileptal OR "GP 47680" OR Paliperidone OR "9 OH Risperidone" OR "9 Hydroxy Risperidone" OR "9 Hydroxyrisperidone" OR Invega OR "R 76477" OR R76477 OR Paroxetine OR "BRL 29060" OR BRL29060 OR "FG 7051" OR FG7051 OR Seroxat OR Paxil OR Aropax OR Periciazine OR Propericiazine OR Pericyazine OR Neuleptil OR Neuleptyl OR Aolept OR Phenelzine OR "Beta Phenylethylhydrazine" OR "2 Phenethylhydrazine" OR Fenelzin OR Phenethylhydrazine OR Nardelzine OR Nardil OR Phenobarbital OR Phenobarbitone OR "Phenylethylbarbituric Acid" OR Phenemal OR Phenylbarbital OR Hysteps OR Luminal OR Gardenal OR Pindolol OR Prindolol OR Visken OR "LB 46" OR LB46 OR Prazepam OR Lysanxia OR Reapam OR Centrax OR Demetrin OR Pregabalin OR "3 Isobutyl GABA" OR Lyrica OR "CI 1008" OR CI1008 OR Prochlorperazine OR Compazine OR Promazine OR Sparine OR Sinophenin OR Protactyl OR Promethazine OR Prometazin OR Proazamine OR Rumergan OR Diprazin OR Phenergan OR Phenargan OR Phensedyl OR Pipolfen OR Pipolphen OR Promet OR Prothazin OR Pyrethia OR Remsed OR Atosil OR Diphergan OR Propranolol OR Propanolol OR Inderal OR Avlocardyl OR "AY 20694" OR AY20694 OR Rexigen OR Dexpropranolol OR Dociton OR Obsidan OR Obzidan OR Anaprilin OR Anapriline OR Betadren OR Protriptyline OR Vivactil OR Quetiapine OR "ICI 204,636" OR "ICI 204636" OR ICI204636 OR Seroquel OR Reboxetine OR Vestra OR Remoxipride OR "FLA 731" OR FLA731 OR Risperidone OR Risperdal OR Risperidal OR "R 64,766" OR "R64,766" OR "R 64766" OR R64766 OR Selegiline OR Selegyline OR "L Deprenyl" OR "E 250" OR E250 OR Eldepryl OR Emsam OR Zelapar OR Deprenil OR Deprenalin OR Yumex OR Jumex OR Humex OR Deprenyl OR Sertindole OR Serlect OR "Lu 23 174" OR Serdolect OR Sertraline OR Zoloft OR Altruline OR Lustral OR Aremis OR Besitran OR Sealdin OR Gladem OR Sulpiride OR Sulperide OR Arminol OR Deponerton OR Meresa OR Desisulpid OR Digton OR Dogmatil OR Dolmatil OR Eglonyl OR Ekilid OR Guastil OR Lebopride OR Neogama OR Pontiride OR Psicocen OR Sulp OR Sulpitil OR Sulpivert OR Sulpor OR Synedil OR Tepavil OR Aiglonyl OR Temazepam OR Hydroxydiazepam OR Methyloxazepam OR Signopam OR Tenox OR "WY 3917" OR WY3917 OR Dasuen OR Euhypnos OR Levanxol OR "Norkotral Tema" OR Normison OR Nocturne OR Temtabs OR Normitab OR Nortem OR Planum OR "Pronervon T" OR Remestan OR Restoril OR "Ro 5 5345" OR Ro55345 OR "SaH 47 603" OR "SaH 47603" OR Temaze OR "Temazep von CT" OR Thioridazine OR ApoThioridazine OR Meleril OR Melleril OR Melleryl OR Mellaril OR Melleretten OR Melzine OR Thiozine OR Sonapax OR Thioridazineneurazpharm OR Aldazine OR Rideril OR Thiothixene OR Tiotixene OR Navane OR Topiramate OR USL255 OR "McN 4853" OR Topamax OR Epitomax OR Tranylcypromine OR "Trans 2 Phenylcyclopropylamine" OR Jatrosom OR Transamine OR Parnate OR Trazodone OR Tradozone OR "AF 1161" OR AF1161 OR Deprax OR Desyrel OR Molipaxin OR Trittico OR Thombran OR "Trazodon Hexal" OR "Trazodon Neuraxpharm" OR Trazon OR Triazolam OR "U 33,030" OR "U33,030" OR Halcion OR Trilam OR "Apo Triazo" OR Trifluoperazine OR Trifluoroperazine OR Trifluperazine OR Eskazine OR Flupazine OR Terfluzine OR Triftazin OR Stelazine OR Trimipramine OR Trimeprimine OR Herphonal OR Trimineurin OR NovoTripramine OR Rhotrimine OR Stangyl OR Surmontil OR Trimidura OR Trimineurin OR Trimipramin OR "Apo Trimip" OR ApoTrimip OR Eldoral OR Venlafaxine OR "Wy 45030" OR Wy45030 OR "Wy 45,030" OR "Wy45,030" OR Effexor OR Trevilor OR Vandral OR Efexor OR Dobupal OR Vigabatrin OR "Gamma Vinyl GABA" OR "Gamma Vinyl Gamma Aminobutyric Acid" OR Sabril OR Sabrilex OR Vortioxetine OR Brintellix OR "Lu AA21004" OR LuAA21004 OR Zaleplon OR "SKP 1041" OR Sonata OR Zelepion OR Starnoc OR "CL 284,846" OR "CL284,846" OR "CL 284846" OR "L 846" OR Ziprasidone OR Ziprazidone OR "CP 88,059" OR "CP 88059" OR Zolpidem OR Amsic OR Bikalm OR Dalparan OR "SL 80.0750" OR "SL 800750 23 N" OR Stilnoct OR Stilnox OR Zodormdura OR Zoldem OR Zolirin OR "Zolpi Lich" OR Zolpinox OR Zolpimist OR Ambien OR Zopiclone OR Zop OR Zopicalma OR Zopiclodura OR Zopiclon OR Zopitan OR Zorclone OR Imovane OR Ximovan OR Zimovane OR Limovan OR Optidorm OR Rhovane OR "RP 27 267" OR Siaten OR Somnosan OR Zileze OR Zimoclone OR "Zopi Puren" OR Zopicalm OR Zotepine OR Zoleptil OR Nipolept OR Zuclopenthixol OR Zuclopentixol OR Clopixol OR Zuclopenthixole OR Acuphase).ti,ab.
3. (1 AND 2) NOT (Animals NOT (Humans NOT Animals)).sh.
4. Limit 3 to English
5. Depression/ or Mood Disorders/ or Depressive Disorder/ or "Depressive Disorder, Major"/ or Dysthymic Disorder/ or "Depressive Disorder, Treatment‐Resistant"/ or "Affective Disorders, Psychotic"/ or Cyclothymic Disorder/ or ("Affective Disorder" or "Affective Disorders" or "Affective Psychoses" or "Affective Psychosis" or Cyclothymic or Depress* or Dysthym* or "Mood Disorder" or "Mood Disorders").ti,ab.
6. (201803* or 201804* or 201805* or 201806* or 201807* or 201808* or 201809* or 201810* or 201811* or 201812* or 2019* or 202*).dc,dp,dt,ed,ep,ez,yr.
7. 4 and 5 and 6

**F. APA PsycInfo 1806 to April Week 4 2020**

1. Exp Head Injuries/ OR Exp Traumatic Brain Injury/ OR (((Brain OR Cerebr$ OR Crani$ OR Crushing Skull OR Diffuse Axonal OR Head OR Hemisphere?) adj1 (Injur$ OR Trauma$)) OR ((Cerebr$ OR Crani$ OR Head) adj (Lesion? OR Wound?)) OR ((Posttraumatic OR Traumatic) adj Encephalopath$) OR (Traumatic adj (Brain OR Cerebr$)) OR Concuss$ OR DAI OR DAIs OR Pneumocephalus OR TBI OR TBIs).ti,ab.
2. Tranquilizing Drugs/ OR Anticonvulsive Drugs/ OR Antidepressant Drugs/ OR Neuroleptic Drugs/ OR Benzodiazepines/ OR Adrenergic Blocking Drugs/ OR Alprazolam/ OR Amitriptyline/ OR Aripiprazole/ OR Atomoxetine/ OR Bupropion/ OR Buspirone/ OR Carbamazepine/ OR Chlordiazepoxide/ OR Chlorpromazine/ OR Citalopram/ OR Chlorimipramine/ OR Clonazepam/ OR Clozapine/ OR Desipramine/ OR Dextroamphetamine/ OR Diazepam/ OR Doxepin/ OR Flunitrazepam/ OR Fluoxetine/ OR Fluphenazine/ OR Fluvoxamine/ OR Haloperidol/ OR Imipramine/ OR Isocarboxazid/ OR Lithium/ OR Lithium Carbonate/ OR Lorazepam/ OR Loxapine/ OR Methylphenidate/ OR Mianserin/ OR Midazolam/ OR Moclobemide/ OR Molindone/ OR Nitrazepam/ OR Nortriptyline/ OR Olanzapine/ OR Oxazepam/ OR Paroxetine/ OR Phenelzine/ OR Phenobarbital/ OR Pregabalin/ OR Prochlorperazine/ OR Promazine/ OR Promethazine/ OR Propranolol/ OR Quetiapine/ OR Risperidone/ OR Sertraline/ OR Sulpiride/ OR Thioridazine/ OR Thiothixene/ OR Tranylcypromine/ OR Trazodone/ OR Triazolam/ OR Trifluoperazine/ OR Valproic Acid/ OR Venlafaxine/ OR ((Adrenergic adj Beta adj2 (Antagonist OR Block$)) OR Anti Anxiety OR Anti Convuls$ OR Anti Depress$ OR Anti Epilep$ OR Anti Psychotic? OR Antianxiety OR Anticonvuls$ OR Antidepress$ OR Antiepilep$ OR Antipsychotic$ OR Anxiolytic$ OR Benzodiazepine$ OR (Beta adj Block$) OR (Beta adj1 Adrenergic adj2 Block$) OR Thymoanaleptic$ OR Thymoleptic$ OR Agomelatine OR "S 20098" OR S20098 OR Thymanax OR Valdoxan OR "AGO 178" OR AGO178 OR Alprazolam OR Alprazolan OR "Apo Alpraz" OR ApoAlpraz OR Cassadan OR "D 65MT" OR D65MT OR Xanax OR Tafil OR Trankimazin OR "Novo Alprazol" OR NovoAlprazol OR "Nu Alpraz" OR NuAlpraz OR Ralozam OR "U-31,889" OR "U31,889" OR Alprox OR Esparon OR Kalma OR Amisulpride OR Sultopride OR Barnetil OR "DAN 2163" OR Solian OR "LIN 1418" OR Amitriptyline OR Amineurin OR Amitrip OR Amitriptylin OR Amitrol OR Tryptine OR ApoAmitriptyline OR Damilen OR Domical OR Laroxyl OR Endep OR Lentizol OR Novoprotect OR Saroten OR Sarotex OR Syneudon OR Triptafen OR Tryptizol OR Tryptanol OR Elavil OR Anapsique OR Amoxapine OR Desmethylloxapine OR "CL 67,772" OR "CL67,772" OR Demolox OR Asendin OR Defanyl OR Asendis OR Aripiprazole OR Aripiprazol OR "OPC 14597" OR Abilify OR Asenapine OR Saphris OR "OR G 5222" OR Atenolol OR Tenormine OR Tenormin OR "ICI 66082" OR ICI66082 OR Atomoxetine OR Tomoxetine OR Strattera OR "LY 139603" OR Benztropine OR Benzatropine OR Bensylate OR PMSBenztropine OR Cogentin OR Cogentinol OR Methylbenztropine OR ApoBenztropine OR Brexpiprazole OR Bromazepam OR BromaLich OR "Bromaz 1A Pharma" OR Bromazanil OR "Bromazep von CT" OR Durazanil OR Lexotan OR Lexotanil OR Lexatin OR Lexomil OR "Ro 5-3350" OR "Ro 53350" OR Anxyrex OR Bupropion OR Amfebutamone OR Zyntabac OR Quomen OR Wellbutrin OR Zyban OR Buspirone OR "MJ 9022 1" OR MJ90221 OR Neurosine OR Busp OR Anxut OR Buspar OR Bespar OR Carbamazepine OR Tegretol OR Carbazepin OR Epitol OR Finlepsin OR Neurotol OR Amizepine OR Cariprazine OR "RGH 188" OR Chlordiazepoxide OR Methaminodiazepoxide OR Librium OR Chlozepid OR Elenium OR Chlorpromazine OR Thorazine OR Aminazine OR Largactil OR Chlordelazine OR Contomin OR Fenactil OR Propaphenin OR Chlorazine OR Citalopram OR Cytalopram OR "Lu 10 171" OR Lu10171 OR Escitalopram OR Lexapro OR Clobazam OR "HR 376" OR Onfi OR "LM 2717" OR Frisium OR Urbanyl OR Clomipramine OR Chlomipramine OR Chlorimipramine OR Hydiphen OR Anafranil OR Clonazepam OR "Ro 5 4023" OR "Ro 54023" OR Antelepsin OR Rivotril OR Clopenthixol OR Zuclopenthixol OR Cisordinol OR Clorazepate OR Chlorazepate OR Tranxene OR Tranxilium OR "4306 CB" OR Clozapine OR Clozaril OR Leponex OR Desipramine OR Desmethylimipramine OR Demethylimipramine OR Norpramin OR Pertofrane OR Pertrofran OR Pertofran OR Petylyl OR Desvenlafaxine OR "O Desmethylvenlafaxine" OR "WY 45,233" OR "WY 45,233" OR "WY45,233" OR "WY 45233" OR WY45233 OR Pristiq OR Dextroamphetamine OR Dexamphetamine OR Dexamfetamine OR "Dextro Amphetamine" OR "D Amphetamine" OR Dexedrine OR DextroStat OR Oxydess OR Diazepam OR Diazemuls OR Faustan OR Valium OR Seduxen OR Sibazon OR Stesolid OR Apaurin OR Relanium OR "Valproic Acid" OR Divalproex OR "Propylisopropylacetic Acid" OR "2 Propylpentanoic Acid" OR Convulsofin OR Depakene OR Depakine OR Depakote OR Vupral OR Valproate OR Ergenyl OR "Dipropyl Acetate" OR Domperidone OR Domperidon OR Domidon OR Gastrocure OR Motilium OR Nauzelin OR Peridys OR "R 33,812" OR "R33,812" OR "R 33812" OR R33812 OR Dothiepin OR Dosulepin OR Prothiaden OR Doxepin OR Deptran OR Desidox OR Doneurin OR Doxepia OR Espadox OR Mareen OR Prudoxin OR Quitaxon OR Sinequan OR Sinquan OR Zonalon OR Xepin OR Aponal OR ApoDoxepin OR Droperidol OR Inapsine OR Dehidrobenzperidol OR Dehydrobenzperidol OR Droleptan OR Duloxetine OR "LY 248686" OR LY248686 OR "LY 227942" OR LY227942 OR Cymbalta OR Estazolam OR Tasedan OR ProSom OR "D 40TA" OR D40TA OR Nuctalon OR Eszopiclone OR Lunesta OR Estorra OR Flunitrazepam OR Fluridrazepam OR Flunibeta OR Flunimerck OR Fluninoc OR Rohypnol OR Rohipnol OR Narcozep OR "Flunizep von CT" OR "RO 5 4200" OR RO54200 OR Fluoxetine OR Fluoxetin OR "Lilly 110140" OR Lilly110140 OR Sarafem OR Prozac OR Flupenthixol OR Flupentixol OR Emergil OR Fluanxol OR Fluphenazine OR Flufenazin OR Lyogen OR Prolixin OR Fluvoxamine OR Fluvoxadura OR Fluvoxamin OR Fluvoxamina OR Luvox OR Fevarin OR Floxyfral OR Dumirox OR Faverin OR Desiflu OR "DU 23000" OR DU23000 OR Guanfacine OR Tenex OR Lon798 OR "BS 100 141" OR BS100141 OR Estulic OR Haloperidol OR Haldol OR Iloperidone OR Zomaril OR Fanapt OR "HP 873" OR Imipramine OR Imizin OR Norchlorimipramine OR Imidobenzyle OR Tofranil OR Melipramine OR Pryleugan OR Janimine OR Isocarboxazid OR Lamotrigine OR Crisomet OR Lamictal OR Lamiktal OR "BW 430C" OR Labileno OR Methotrimeprazine OR Levomepromazine OR Levopromazine OR Levomeprazin OR Tisercin OR Tizercine OR Tizertsin OR Lithium OR Dilithium OR Lithane OR Lithobid OR Lithonate OR "CP-15,467 61" OR "CP15,46761" OR Micalith OR "NSC 16895" OR NSC16895 OR Priadel OR "Quilinorm Retard" OR Quilinormretard OR Eskalith OR Lithotabs OR Lorazepam OR Ativan OR Temesta OR "Orfidal Wyeth" OR Donix OR Duralozam OR Durazolam OR Idalprem OR Laubeel OR "Lorazep von CT" OR "Novo Lorazem" OR NovoLorazem OR "Nu Loraz" OR NuLoraz OR Sedicepan OR Sinestron OR Somagerol OR Tolid OR "WY 4036" OR WY4036 OR ApoLorazepam OR Loxapine OR Cloxazepine OR Oxilapine OR Loxitane OR Loxipine OR Loxapinsuccinate OR "CL 71,563" OR "CL71,563" OR Lurasidone OR "SM 13496" OR SM13496 OR "SM-13,496" OR "SM13,496" OR Latuda OR Methylphenidate OR Metadate OR Equasym OR Methylin OR Concerta OR Phenidylate OR Ritalin OR Ritaline OR Tsentedrin OR Centedrin OR Daytrana OR Mianserin OR Tolvon OR Lerivon OR Org GB 94 OR Midazolam OR Dormicum OR Versed OR "Ro 21 3981" OR "Ro 213981" OR Milnacipran OR Midalcipran OR Levomilnacipran OR Savella OR "F 2207" OR Ixel OR Mirtazapine OR "6 Azamianserin" OR Esmirtazapine OR Remeron OR Remergil OR Zispin OR Norset OR Rexer OR "Org 50081" OR " OR G 3770" OR Moclobemide OR Moclobamide OR Arima OR Aurorix OR Manerix OR Moclamine OR Aurorex OR Deprenorm OR Feraken OR Moclobemid OR Moclobeta OR Moclodura OR Moclonorm OR Rimoc OR "Ro 11 1163" OR Modafinil OR Benzhydrylsulfinylacetamide OR "CRL 40476" OR Vigil OR Provigil OR Sparlon OR Alertec OR Modiodal OR Molindone OR Moban OR Nefazodone OR Rulivan OR Serzone OR Dutonin OR Nefadar OR Menfazona OR Nitrazepam OR Nitrodiazepam OR "Dormo Puren" OR Eatan OR Imadorm OR Imeson OR Mogadon OR Nitrazadon OR Nitrazep OR Novanox OR Radedorm OR Remnos OR Serenade OR Somnite OR Alodorm OR Dormalon OR Nortriptyline OR Desmethylamitriptylin OR Desitriptyline OR Aventyl OR Paxtibi OR Allegron OR Norfenazin OR Pamelor OR Nortrilen OR Olanzapine OR Zolafren OR "LY 170052" OR Zyprexa OR "LY 170053" OR Oxazepam OR Serax OR Tazepam OR Adumbran OR Oxcarbazepine OR Timox OR Trileptal OR "GP 47680" OR Paliperidone OR "9 OH Risperidone" OR "9 Hydroxy Risperidone" OR "9 Hydroxyrisperidone" OR Invega OR "R 76477" OR R76477 OR Paroxetine OR "BRL 29060" OR BRL29060 OR "FG 7051" OR FG7051 OR Seroxat OR Paxil OR Aropax OR Periciazine OR Propericiazine OR Pericyazine OR Neuleptil OR Neuleptyl OR Aolept OR Phenelzine OR "Beta Phenylethylhydrazine" OR "2 Phenethylhydrazine" OR Fenelzin OR Phenethylhydrazine OR Nardelzine OR Nardil OR Phenobarbital OR Phenobarbitone OR "Phenylethylbarbituric Acid" OR Phenemal OR Phenylbarbital OR Hysteps OR Luminal OR Gardenal OR Pindolol OR Prindolol OR Visken OR "LB 46" OR LB46 OR Prazepam OR Lysanxia OR Reapam OR Centrax OR Demetrin OR Pregabalin OR "3 Isobutyl GABA" OR Lyrica OR "CI 1008" OR CI1008 OR Prochlorperazine OR Compazine OR Promazine OR Sparine OR Sinophenin OR Protactyl OR Promethazine OR Prometazin OR Proazamine OR Rumergan OR Diprazin OR Phenergan OR Phenargan OR Phensedyl OR Pipolfen OR Pipolphen OR Promet OR Prothazin OR Pyrethia OR Remsed OR Atosil OR Diphergan OR Propranolol OR Propanolol OR Inderal OR Avlocardyl OR "AY 20694" OR AY20694 OR Rexigen OR Dexpropranolol OR Dociton OR Obsidan OR Obzidan OR Anaprilin OR Anapriline OR Betadren OR Protriptyline OR Vivactil OR Quetiapine OR "ICI 204,636" OR "ICI 204636" OR ICI204636 OR Seroquel OR Reboxetine OR Vestra OR Remoxipride OR "FLA 731" OR FLA731 OR Risperidone OR Risperdal OR Risperidal OR "R 64,766" OR "R64,766" OR "R 64766" OR R64766 OR Selegiline OR Selegyline OR "L Deprenyl" OR "E 250" OR E250 OR Eldepryl OR Emsam OR Zelapar OR Deprenil OR Deprenalin OR Yumex OR Jumex OR Humex OR Deprenyl OR Sertindole OR Serlect OR "Lu 23 174" OR Serdolect OR Sertraline OR Zoloft OR Altruline OR Lustral OR Aremis OR Besitran OR Sealdin OR Gladem OR Sulpiride OR Sulperide OR Arminol OR Deponerton OR Meresa OR Desisulpid OR Digton OR Dogmatil OR Dolmatil OR Eglonyl OR Ekilid OR Guastil OR Lebopride OR Neogama OR Pontiride OR Psicocen OR Sulp OR Sulpitil OR Sulpivert OR Sulpor OR Synedil OR Tepavil OR Aiglonyl OR Temazepam OR Hydroxydiazepam OR Methyloxazepam OR Signopam OR Tenox OR "WY 3917" OR WY3917 OR Dasuen OR Euhypnos OR Levanxol OR "Norkotral Tema" OR Normison OR Nocturne OR Temtabs OR Normitab OR Nortem OR Planum OR "Pronervon T" OR Remestan OR Restoril OR "Ro 5 5345" OR Ro55345 OR "SaH 47 603" OR "SaH 47603" OR Temaze OR "Temazep von CT" OR Thioridazine OR ApoThioridazine OR Meleril OR Melleril OR Melleryl OR Mellaril OR Melleretten OR Melzine OR Thiozine OR Sonapax OR Thioridazineneurazpharm OR Aldazine OR Rideril OR Thiothixene OR Tiotixene OR Navane OR Topiramate OR USL255 OR "McN 4853" OR Topamax OR Epitomax OR Tranylcypromine OR "Trans 2 Phenylcyclopropylamine" OR Jatrosom OR Transamine OR Parnate OR Trazodone OR Tradozone OR "AF 1161" OR AF1161 OR Deprax OR Desyrel OR Molipaxin OR Trittico OR Thombran OR "Trazodon Hexal" OR "Trazodon Neuraxpharm" OR Trazon OR Triazolam OR "U 33,030" OR "U33,030" OR Halcion OR Trilam OR "Apo Triazo" OR Trifluoperazine OR Trifluoroperazine OR Trifluperazine OR Eskazine OR Flupazine OR Terfluzine OR Triftazin OR Stelazine OR Trimipramine OR Trimeprimine OR Herphonal OR Trimineurin OR NovoTripramine OR Rhotrimine OR Stangyl OR Surmontil OR Trimidura OR Trimineurin OR Trimipramin OR "Apo Trimip" OR ApoTrimip OR Eldoral OR Venlafaxine OR "Wy 45030" OR Wy45030 OR "Wy 45,030" OR "Wy45,030" OR Effexor OR Trevilor OR Vandral OR Efexor OR Dobupal OR Vigabatrin OR "Gamma Vinyl GABA" OR "Gamma Vinyl Gamma Aminobutyric Acid" OR Sabril OR Sabrilex OR Vortioxetine OR Brintellix OR "Lu AA21004" OR LuAA21004 OR Zaleplon OR "SKP 1041" OR Sonata OR Zelepion OR Starnoc OR "CL 284,846" OR "CL284,846" OR "CL 284846" OR "L 846" OR Ziprasidone OR Ziprazidone OR "CP 88,059" OR "CP 88059" OR Zolpidem OR Amsic OR Bikalm OR Dalparan OR "SL 80.0750" OR "SL 800750 23 N" OR Stilnoct OR Stilnox OR Zodormdura OR Zoldem OR Zolirin OR "Zolpi Lich" OR Zolpinox OR Zolpimist OR Ambien OR Zopiclone OR Zop OR Zopicalma OR Zopiclodura OR Zopiclon OR Zopitan OR Zorclone OR Imovane OR Ximovan OR Zimovane OR Limovan OR Optidorm OR Rhovane OR "RP 27 267" OR Siaten OR Somnosan OR Zileze OR Zimoclone OR "Zopi Puren" OR Zopicalm OR Zotepine OR Zoleptil OR Nipolept OR Zuclopenthixol OR Zuclopentixol OR Clopixol OR Zuclopenthixole OR Acuphase).ti,ab.
3. 1 AND 2
4. Limit 3 to English Language
5. "Depression (Emotion)"/ or Major Depression/ or Dysthymic Disorder/ or Reactive Depression/ or Recurrent Depression/ or Treatment Resistant Depression/ or Atypical Depression/ or Beck Depression Inventory/ or Zungs Self Rating Depression Scale/ or Affective Disorders/ or Affective Psychosis/ or Cyclothymic Disorder/ or ("Affective Disorder" or "Affective Disorders" or "Affective Psychoses" or "Affective Psychosis" or Cyclothymic or Depress* or Dysthym* or "Mood Disorder" or "Mood Disorders").ti,ab.
6. (2018* or 2019* or 202*).dp.
7. 4 AND 5 AND 6
