# Supplementary material for: Effectiveness of Pharmacotherapy for Depression after Adult Traumatic Brain Injury: an Umbrella Review
Source: Neuropsychol Rev. 2022 Jun 14;33(2):393–431. doi: 10.1007/s11065-022-09543-6 (PMC10148771; doi:10.1007/s11065-022-09543-6)
Supplement: Supplementary file 2 — Supplementary file2 (DOCX 44 KB) [file 11065_2022_9543_MOESM2_ESM.docx]

**Appendix 2**

**Table 1**

*Reviews Excluded at Full Text Screening Stage for the Umbrella Review and Reason for Exclusion*

| Reason for Exclusion | Citations |
| --- | --- |
| DATABASE SEARCH | |
| Does not fulfil our criteria for a systematic review | [1-6] |
| Not written in English | [7] |
| Intervention not pharmacotherapy | [8] |
| Purpose of studies in review was not to treat depression | [9-13] |
| Other study design - protocol paper | [14]  This protocol was developed by the authors of this umbrella review. After the protocol had been published, a number of other systematic reviews on this topic were published.  [15]  Authors were e-mailed twice, with a 6-week period between e-mails. No response was received. |
| Other study design – narrative review or editorial | [16-18] |
| Other study design – conference abstract | [19-23] |
| Other study design – withdrawal notice | [24] |
| Other – review has now been updated | [24] |
| SUPPLEMENTARY SEARCH | |
| Does not fulfil our criteria for a systematic review | [25, 26] |
| Other study design – protocol paper | [27]  Lead author contacted via ResearchGate and provided details for the published review. Published review (Beedham 2020) has been included in this umbrella review. |

Note: No excluded reviews were considered to be ‘near misses’ (i.e. met many but not all inclusion criteria).

Table 2. Primary studies excluded from systematic review and reasons for exclusion.

| Citation | Reason for Exclusion |
| --- | --- |
| Wrong outcomes. | [28]  This study did not examine change in depression symptoms following pharmacotherapy intervention. This study had two primary outcomes; (1) receipt of any medication for depression and (2) receipt of psychotherapy.  [29]  This study did not examine change in depression symptoms following pharmacotherapy intervention. The primary outcome for this study was diagnosis of epilepsy. |
| Ongoing clinical trial | [30] |
| Insufficient information regarding intervention | [31]  No drug class specified. |
| Study included in systematic reviews that form the umbrella review | [32] |

Note: No excluded studies were considered to be ‘near misses’ (i.e. met many but not all inclusion criteria).

1. Bhalerao, S.U., et al., *Understanding the neuropsychiatric consequences associated with significant traumatic brain injury.* Brain injury, 2013. **27**(7-8): p. 767-774.

2. Anghinah, R., et al., *Traumatic brain injury pharmacological treatment: recommendations.* Arq Neuropsiquiatr, 2018. **76**(2): p. 100-103.

3. Bernardo, C.G., V. Singh, and P.M. Thompson, *Safety and efficacy of psychopharmacological agents used to treat the psychiatric sequelae of common neurological disorders.* Expert opinion on drug safety, 2008. **7**(4): p. 435-445.

4. Kraus, M.F., *Neuropsychiatric sequelae of stroke and traumatic brain injury: the role of psychostimulants.* The International Journal of Psychiatry in Medicine, 1995. **25**(1): p. 39-51.

5. Rosenthal, M., B.K. Christensen, and T.P. Ross, *Depression following traumatic brain injury.* Archives of physical medicine and rehabilitation, 1998. **79**(1): p. 90-103.

6. Chew, E. and R.D. Zafonte, *Pharmacological management of neurobehavioral disorders following traumatic brain injury--a state-of-the-art review.* J Rehabil Res Dev, 2009. **46**(6): p. 851-79.

7. Richard, I., et al. *Pharmacological treatment of post-traumatic behavioural disorders*. in *Annales de readaptation et de medecine physique: revue scientifique de la Societe francaise de reeducation fonctionnelle de readaptation et de medecine physique*. 2003.

8. Matarazzo, B.B., et al., *Evidence-based intervention strategies for veterans and military personnel with traumatic brain injury and co-morbid mental health conditions: a systematic review.* Brain Impairment, 2013. **14**(1): p. 42.

9. Jones, M., L. Acion, and R.E. Jorge, *What are the complications and emerging strategies for preventing depression following traumatic brain injury?* Expert review of neurotherapeutics, 2017. **17**(6): p. 631-640.

10. de Sousa, C.N.S., et al., *Alpha-lipoic acid in the treatment of psychiatric and neurological disorders: a systematic review.* Metabolic brain disease, 2019. **34**(1): p. 39-52.

11. Sami, M.B. and R. Faruqui, *The effectiveness of dopamine agonists for treatment of neuropsychiatric symptoms post brain injury and stroke.* Acta neuropsychiatrica, 2015. **27**(6): p. 317-326.

12. Slattery, J., et al., *Clinical trials of N-acetylcysteine in psychiatry and neurology: a systematic review.* Neuroscience & Biobehavioral Reviews, 2015. **55**: p. 294-321.

13. Rothschild, L., et al., *Mood and anxiety in concussion and mild traumatic brain injury (mTBI): a systematic review.* Critical Reviews™ in Physical and Rehabilitation Medicine, 2015. **27**(2-4).

14. Clay, F.J., et al., *Safety and effectiveness of pharmacotherapy for depression in adults who have sustained a traumatic brain injury: a systematic review protocol.* JBI Evidence Synthesis, 2017. **15**(9): p. 2270-2286.

15. Vattakatuchery, J., et al., *Pharmacological interventions for depression in people with traumatic brain injury: systematic review.* Journal of neurology, neurosurgery, and psychiatry, 2014. **85**: p. e3.

16. Tsaousides, T., T.A. Ashman, and W.A. Gordon, *Diagnosis and treatment of depression following traumatic brain injury.* Brain Impairment, 2013. **14**(1): p. 63-76.

17. Rapoport, M.J., *Do antidepressants improve recovery from neurologic illness?* The American Journal of Geriatric Psychiatry, 2011. **19**(12): p. 985-988.

18. Silverberg, N.D. and W.J. Panenka, *Antidepressants for depression after concussion and traumatic brain injury are still best practice.* BMC psychiatry, 2019. **19**(1): p. 100.

19. Teasell, R., et al., *Poster 17: Treatment of Affective Disorders and Challenging Behavior Following Brain Injury.* Archives of Physical Medicine and Rehabilitation, 2010. **91**(10): p. e9-e10.

20. Rice, D.B., et al., *Interventions for Depression: Combining the Stroke and Acquired Brain Injury Rehabilitation Literature.* Archives of Physical Medicine and Rehabilitation, 2014. **95**(10): p. e17-e18.

21. Kreitzer, N.A., R.; McCullumsmith, C.; Kurowski, B.; Foreman, B.; Adeoye, O., *Pharmacotherapy for the treatment of depression following traumatic brain injury: A meta-analysis.* 2017, Neurocritical Care. p. S89.

22. Salter, K., *Poster 43 Pharmacotherapy for Depression Post Traumatic Brain Injury: A Meta-Analysis.* Archives of Physical Medicine and Rehabilitation, 2012. **93**(10): p. e26.

23. Plantier, D., Luaute, J., Wiart, L., Stefan, A., Hamonet, J., Arnould, A., Aubert, S., Beis, J. M., Blais, L., & Cazals, M. C. , *Support disorders after traumatic brain injury, guidelines: Medications. .* Annals of Physical and Rehabilitation Medicine, 2013. **Supplement 1**: p. e95.

24. Gill, D. and S. Hatcher, *Antidepressants for depression in medical illness.* Cochrane Database of Systematic Reviews, 2000(3).

25. Bhatnagar, S., M.A. Iaccarino, and R. Zafonte, *Pharmacotherapy in rehabilitation of post-acute traumatic brain injury.* Brain Res, 2016. **1640**(Pt A): p. 164-179.

26. Rabinowitz, A.R. and T.K. Watanabe, *Pharmacotherapy for Treatment of Cognitive and Neuropsychiatric Symptoms After mTBI.* The Journal of head trauma rehabilitation, 2020. **35**(1): p. 76-83.

27. Beedham, W., Upthegrove, R., Belli, A. , *The management of depression following traumatic brain injury: a systematic review with meta-analysis. .* PROSPERO, 2019. **CRD42019122600**.

28. Albrecht, J.S., et al., *Receipt of Treatment for Depression Following Traumatic Brain Injury.* The Journal of Head Trauma Rehabilitation, 2020.

29. Christensen, J., et al., *Selective serotonin reuptake inhibitors and risk of epilepsy after traumatic brain injury–A population based cohort study.* PloS one, 2019. **14**(7): p. e0219137.

30. *Allopregnanolone in chronic complex traumatic brain injury.*

31. Kruse, R.C., et al., *Pharmacologic Treatment for Depression at Injury Is Associated With Fewer Clinician Visits for Persistent Symptoms After Mild Traumatic Brain Injury: A Medical Record Review Study.* PM&R, 2018. **10**(9): p. 898-902.

32. Fann, J.R., et al., *Sertraline for Major Depression During the Year Following Traumatic Brain Injury: A Randomized Controlled Trial.* J Head Trauma Rehabil, 2017. **32**(5): p. 332-342.
