# Supplementary material for: Effectiveness of Pharmacotherapy for Depression after Adult Traumatic Brain Injury: an Umbrella Review
Source: Neuropsychol Rev. 2022 Jun 14;33(2):393–431. doi: 10.1007/s11065-022-09543-6 (PMC10148771; doi:10.1007/s11065-022-09543-6)
Supplement: Supplementary file 3 — Supplementary file3 (DOCX 28 KB) [file 11065_2022_9543_MOESM3_ESM.docx]

**Appendix 3**

**Data Extraction Template used for the Umbrella Review**

| **Reviewer Full Name** |  |
| --- | --- |
| **Date Completed** |  |
| **Author-Year** |  |

**Key Notes**

- Only extract information from studies with a pharmacological intervention.
- Only extract information from studies with an intervention that specially aimed to treat depression, do not include studies for which depression was a secondary outcome.
- If detail is missing from a systematic review please record ‘Not Reported’ or ‘NR’.

**Review Details**

| **Financial Support** | Yes / No / Not Stated  If Yes:  Pharmaceutical Company / Other financial support |
| --- | --- |
| **Declared Conflicts of Interest** | Stated / Not Stated  If stated, were there conflicts: Yes / NO  If yes, please provide brief details: |
| **Overall Analysis** | meta-analysis  narrative summary |
| **Primary Studies Included**  List all primary studies of relevance to this umbrella review (Author, Year)  *List the author and year for all primary studies or relevance to this umbrella review* |  |

| **Study Objective** | **Date review last assessed as up-to-date** | **Inclusion criteria for primary studies** | **Exclusion criteria for primary studies** | **Number of studies included & study designs** *Record the number of each study design (e.g. 4 x RCTs, 5 x cohort etc.)* | **Number of participants included**   - # TBI - # other ABI - # healthy controls |
| --- | --- | --- | --- | --- | --- |
|  |  |  |  |  | **TBI:**  **Other ABI:**  **Healthy Controls:** |

**Search Strategy**

| **Number of databases searched** | **Names of database searched** | **Restrictions imposed for database search** | **Search strategy for one database provided as an appendices?** | **Supplementary searching completed** | **Details of supplementary searching** | **Search for grey literature completed** | **Details of search for grey literature** |
| --- | --- | --- | --- | --- | --- | --- | --- |
|  |  | Yes – Language  Yes – Publication Year  No restrictions |  |  |  |  |  |

**Participants**

| **Author-Year (Primary Study)** | **Gender**  (# male) | **Age**  (Mean and Range) | **Sample Size**   - Report % Sample TBI - If sample includes other ABI, please note whether the data for TBI was presented separately | **TBI**   - Medically confirmed/ self-report - Severity - Time post injury | **Depression Entry Criteria**   - Measure - Cut-off |
| --- | --- | --- | --- | --- | --- |
|  |  |  |  |  |  |
|  |  |  |  |  |  |

**Risk of Bias & Quality**

| **Author-Year (Primary Study)** | **Risk of Bias Instrument** | **Risk of Bias Rating** | **Quality Instrument** | **Quality Rating** |
| --- | --- | --- | --- | --- |
|  |  |  |  |  |
|  |  |  |  |  |
|  |  |  |  |  |

**Outcomes**

| **Author-Year (Primary Study)** | **Depression measurement Tool** |
| --- | --- |
|  |  |
|  |  |
|  |  |

**Heterogeneity**

**Provide details of the analysis of heterogeneity or record ‘N/A’ if this was not done.**

|  |
| --- |

**Findings – Depression**

| **Author – Year**  **Primary Study** | **Intervention**   - Drug name - Drug class - Dose - Frequency - Duration - Comparator | **Full Sample/ Sub-group** Record type of sample (i.e. full sample or sub-group) and size | **Depression Measure** | **Follow-up Point(s)** | **Statistical findings**  (i.e. odds ratios, relative risks etc) | **Significance/ Direction** (i.e. in favour of drug or placebo) | **Conclusions**  (of SR authors) |
| --- | --- | --- | --- | --- | --- | --- | --- |
|  |  |  |  |  |  |  |  |
|  |  |  |  |  |  |  |  |
|  |  |  |  |  |  |  |  |

**Findings – Harms**

| **Author – Year**  **(Primary Study)** | **Intervention** Drug name   - Drug class - Dose - Frequency - Duration - Comparator | **Full Sample/ Sub-group** Record type of sample (i.e. full sample or sub-group) and size | **Harm** | **Follow-up Point(s)** | **Statistical findings** | **Conclusions**  (of SR authors) |
| --- | --- | --- | --- | --- | --- | --- |
|  |  |  |  |  |  |  |
|  |  |  |  |  |  |  |
|  |  |  |  |  |  |  |

**FINAL COMMENTS**

Please document any final comments such as the congruence between the systematic review results and conclusions, limitations of the systematic review and methodological differences between the included systematic reviews.

|  |
| --- |
