# Supplementary material for: Effectiveness of Pharmacotherapy for Depression after Adult Traumatic Brain Injury: an Umbrella Review
Source: Neuropsychol Rev. 2022 Jun 14;33(2):393–431. doi: 10.1007/s11065-022-09543-6 (PMC10148771; doi:10.1007/s11065-022-09543-6)
Supplement: Supplementary file 4 — Supplementary file4 (DOCX 16 KB) [file 11065_2022_9543_MOESM4_ESM.docx]

**Appendix 4**

**Methodology for the Systematic Review**

**Inclusion criteria**

Primary studies were selected for inclusion in the systematic review according to our pre-defined inclusion criteria. The inclusion criteria for the systematic review were identical to that of the umbrella review, with the exception of study design.

**Studies**

The following study designs were considered for inclusion, regardless of sample size and study setting: RCTs, controlled non-randomized clinical trials, quasi-randomized controlled trials, controlled before and after studies, interrupted time series with a control group, interrupted time series without a parallel concurrent control group, analytical observational studies (including cohort and case–control studies), case series with pre-test and post-test outcomes and single arm studies. The following study types were excluded from this review: case reports, qualitative research, editorials and opinion pieces, methodological papers, secondary studies including narrative reviews, systematic reviews and meta-analyses.

**Search strategy**

The design of the search strategy was the same as for the umbrella review.

**Information sources**

The databases searched were MEDLINE (Ovid SP; March 2018 – May 2020), EMBASE (Excerpta Medica Database; Ovid SP; March 2018 – May 2020), PsycINFO (Ovid SP; March 2018 – April 2020), CINAHL (Cumulative Index to Nursing and Allied Health Literature; EBSCO Host; March 2018 – May 2020) and CENTRAL (Cochrane Library; March 2018 – May 2020). We also searched two clinical trial websites (clinicaltrials.gov, Australia New Zealand Clinical Trials Registry; anzctr.org.au), two medical regulatory websites (European Medicines Agency, US Food and Drug Administration), ResearchGate, Google Scholar, TRIP Medical Database (search dates: March 2018 - July 2020). Three key journals were searched online (Journal of Neurotrauma March 2018 – July 2020; Brain Impairment March 2018 – July 2020; Journal of Head Trauma Rehabilitation March 2018 – July 2020). We contacted two key experts in the field to inquire about ongoing or future trials; both responded to confirm no ongoing or future trials of pharmacotherapy for post TBI depression. This systematic review was last assessed as up-to-date in July 2020.

**Study selection**

The study selection process followed the steps outlined above for the umbrella review. As no primary studies were identified for inclusion, data extraction and assessment of methodological quality did not take place.
