# Supplementary material for: Effectiveness of Pharmacotherapy for Depression after Adult Traumatic Brain Injury: an Umbrella Review
Source: Neuropsychol Rev. 2022 Jun 14;33(2):393–431. doi: 10.1007/s11065-022-09543-6 (PMC10148771; doi:10.1007/s11065-022-09543-6)
Supplement: Supplementary file 5 — Supplementary file5 (DOCX 44 KB) [file 11065_2022_9543_MOESM5_ESM.docx]

**Appendix 5**

**Fig.1**

*PRISMA Flow Diagram for the Systematic Review Detailing the Results of the Literature Search, Article Screening and Study Selection Process*

Records identified from:

Websites (k=25)

Clinical trial registries (k=38)

Journal search (k=73)

**Identification of new studies via databases and registers**

**Identification of new studies via other methods**

Records removed before screening:

Duplicate records (k=76)

Records identified from:

Databases (k=576)

**Identification**

**Included**

**Screening**

Reports excluded:

Wrong outcomes (k=2)

Ongoing clinical trial (k=1)

Insufficient information regarding intervention (k=1)

Study included in systematic reviews in the umbrella review (k=1)

Studies included in review (k=0)

Reports not retrieved

(k=0)

Records excluded

(k=495)

Reports assessed for eligibility

(k=5)

Reports sought for retrieval

(k=5)

Records screened

(k=500)

Reports sought for retrieval

(k=0)
