# Supplementary material for: Effectiveness of Pharmacotherapy for Depression after Adult Traumatic Brain Injury: an Umbrella Review
Source: Neuropsychol Rev. 2022 Jun 14;33(2):393–431. doi: 10.1007/s11065-022-09543-6 (PMC10148771; doi:10.1007/s11065-022-09543-6)
Supplement: Supplementary file 6 — Supplementary file6 (DOCX 20 KB) [file 11065_2022_9543_MOESM6_ESM.docx]

**Appendix 6**

**Citations for the 22 Reviews included in the Umbrella Review (listed chronologically)**

1. Deb, S., & Crownshaw, T. (2004). Review of subject The role of pharmacotherapy in the management of behaviour disorders in traumatic brain injury patients. Brain Injury. 18(1), 1-31.
2. Comper, P., Bisschop, S. M., Carnide, N., & Tricco, A. (2005). A systematic review of treatments for mild traumatic brain injury. Brain injury. 19(11), 863-880.
3. Warden, D. L., Gordon, B., McAllister, T. W., Silver, J. M., Barth, J. T., Bruns, J., ... & Kraus, J. (2006). Guidelines for the pharmacologic treatment of neurobehavioral sequelae of traumatic brain injury. Journal of Neurotrauma. 23(10), 1468-1501.
4. Hardy, S. (2009). Methylphenidate for the Treatment of Depressive Symptoms, Including Fatigue and Apathy, in Medically III Older Adults and Terminally III Adults. The American Journal of Geriatric Pharmacotherapy. 7(1), 34-59.
5. Fann, J. R., Hart, T., & Schomer, K. G. (2009). Treatment for depression after traumatic brain injury: a systematic review. Journal of Neurotrauma. 26(12), 2383-2402.
6. Rayner, L., Price, A., Evans, A., Valsraj, K., Higginson, I. J., & Hotopf, M. (2010). Antidepressants for depression in physically ill people. Cochrane Database of Systematic Reviews. (3).
7. Guillamondegui, O. D., Montgomery, S. A., Phibbs, F. T., McPheeters, M. L., Alexander, P. T., Jerome, R. N., ... & Salomon, R. M. (2011). Traumatic Brain Injury and Depression. (Report No. 25). Retrieved from <https://www.ahrq.gov/>.
8. Price, A., Rayner, L., Okon-Rocha, E., Evans, A., Valsraj, K., Higginson, I. J., & Hotopf, M. (2011). Antidepressants for the treatment of depression in neurological disorders: a systematic review and meta-analysis of randomised controlled trials. Journal of Neurology, Neurosurgery & Psychiatry. 82(8), 914-923.
9. Wheaton, P., Mathias, J. L., & Vink, R. (2011). Impact of pharmacological treatments on cognitive and behavioral outcome in the postacute stages of adult traumatic brain injury: a meta-analysis. Journal of Clinical Psychopharmacology. 31(6), 745-757.
10. Barker-Collo, S., Starkey, N., & Theadom, A. (2013). Treatment for depression following mild traumatic brain injury in adults: a meta-analysis. Brain Injury. 27(10), 1124-1133.
11. Plantier, D., & Luauté, J. (2016). Drugs for behavior disorders after traumatic brain injury: systematic review and expert consensus leading to French recommendations for good practice. Annals of Physical and Rehabilitation Medicine. 59(1), 42-57.
12. Salter, K. L., McClure, J. A., Foley, N. C., Sequeira, K., & Teasell, R. W. (2016). Pharmacotherapy for depression posttraumatic brain injury: a meta-analysis. Journal of Head Trauma Rehabilitation. 31(4), E21-E32.
13. Maksimowski, M., & Tampi, R. (2016). Efficacy of stimulants for psychiatric symptoms in individuals with traumatic brain injury. Annals of Clinical Psychiatry. 28(3), 156-166.
14. Paraschakis, A., & Katsanos, A. H. (2017). Antidepressants for depression associated with traumatic brain injury: a meta-analytical study of randomised controlled trials. East Asian Archives of Psychiatry. 27(4), 142.
15. Yue, J. K., Burke, J. F., Upadhyayula, P. S., Winkler, E. A., Deng, H., Robinson, C. K., ... & Ngwenya, L. B. (2017). Selective serotonin reuptake inhibitors for treating neurocognitive and neuropsychiatric disorders following traumatic brain injury: an evaluation of current evidence. Brain sciences. 7(8), 93.
16. Gao, C., Fu, Q., Chen, B., Liu, Z., Zhou, Q., & Jiang, Z. (2019). The influence of sertraline on depressive disorder after traumatic brain injury: A meta-analysis of randomized controlled studies. The American Journal of Emergency Medicine. 37(9), 1778-1783.
17. Kreitzer, N., Ancona, R., McCullumsmith, C., Kurowski, B. G., Foreman, B., Ngwenya, L. B., & Adeoye, O. (2019). The effect of antidepressants on depression after traumatic brain injury: a meta-analysis. The Journal of Head Trauma Rehabilitation. 34(3), E47-E54.
18. Liu, Q., Li, R., Qu, W., Li, B., Yang, W., & Cui, R. (2019). Pharmacological and non-pharmacological interventions of depression after traumatic brain injury: a systematic review. European Journal of Pharmacology. 865, 172775.
19. Reyes, N. G. D., Espiritu, A. I., & Anlacan, V. M. M. (2019). Efficacy of sertraline in post-traumatic brain injury (post-TBI) depression and quality of life: a systematic review and meta-analysis of randomized controlled trials. Clinical Neurology and Neurosurgery. 181, 104-111.
20. Slowinski, A., Coetzer, R., & Byrne, C. (2019). Pharmacotherapy effectiveness in treating depression after traumatic brain injury: a meta-analysis. The Journal of Neuropsychiatry and Clinical Neurosciences. 31(3), 220-227.
21. Peppel, L. D., Ribbers, G. M., & Heijenbrok-Kal, M. H. (2020). Pharmacological and Non-Pharmacological Interventions for Depression after Moderate-to-Severe Traumatic Brain Injury: A Systematic Review and Meta-Analysis. Journal of Neurotrauma. 37(14), 1587-1596.
22. Beedham, W., Belli, A., Ingaralingam, S., Haque, S., & Upthegrove, R. (2020). The management of depression following traumatic brain injury: A systematic review with meta-analysis. Brain Injury. 34(10), 1287-1304.
