# Supplementary material for: Effectiveness of Pharmacotherapy for Depression after Adult Traumatic Brain Injury: an Umbrella Review
Source: Neuropsychol Rev. 2022 Jun 14;33(2):393–431. doi: 10.1007/s11065-022-09543-6 (PMC10148771; doi:10.1007/s11065-022-09543-6)
Supplement: Supplementary file 7 — Supplementary file7 (DOCX 27 KB) [file 11065_2022_9543_MOESM7_ESM.docx]

**Appendix 7**

**Table 1**

*Searches Undertaken by the 22 Systematic Reviews Included in the Umbrella Review*

| Citation | Number of databases searched | Names of database searched | Language restrictions | Date Restrictions |  | Search strategy provided^[[1]](#footnote-1)^ | Details of supplementary searching | Search for unpublished literature conducted |
| --- | --- | --- | --- | --- | --- | --- | --- | --- |
| Beedham (2020) | 7 | CENTRAL, CINAHL, Cochrane Library, EMBASE, Medline, PsycInfo, Web of Science | No | Database inception – Jan 2019 |  | Yes | - Clinical trials websites  - Reference lists  - Consultation with experts  - Digital Dissertations Database  - Conference proceedings | Yes |
| Peppel (2020) | 5 | CENTRAL, EMBASE, Medline^[[2]](#footnote-2)^, PsycInfo, Web of Science | Yes – English | Database inception – Sept 2018 |  | Yes | - Google Scholar  - Reference lists | Yes |
| Gao (2019) | 5 | Cochrane Library, EMBASE, EBSCO, PubMed, Web of Science | NR | 1980 – Feb 2019 |  | No | NR | No |
| Kreitzer (2019) | 2 | Cochrane Library, - PubMed | Yes – English | NR – Sept 2017 |  | Yes | - Clinical trials websites  - Reference lists | Yes |
| Liu (2019) | NR | NR | NR | NR |  | No | NR | NR |
| Reyes (2019) | 5 | CENTRAL, CINAHL, LILACS  PubMed, SCOPUS | Yes – English | 1980 – NR |  | Yes | - Clinical trials websites  - Reference lists | No |
| Slowinski (2019) | 3 | CENTRAL, PubMed, National Institute of Health and Care Excellence Healthcare Database | Yes – English | 1980 – NR |  | Yes | - Google Scholar | No |
| Paraschakis (2017) | 3 | CENTRAL, Medline, SCOPUS | No | 1990 – Aug 2017 |  | Yes | - Reference lists | No |
| Yue (2017) | 2 | CENTRAL, PubMed | Yes – English | NR – Sept 2016 |  | Yes | NR | No |
| Maksimowski (2016) | 5 | Cochrane Library, EMBASE, Medline, PsycInfo, PubMed | Yes – English | Database inception – December 2015 |  | Yes | - Reference lists | No |
| Plantier (2016) | 1^[[3]](#footnote-3)^ | Medline | Yes – Unclear which language | 1990 – June 2015 |  | Yes | Unclear if this was performed | NR |
| Salter (2016) | 5 | CINAHL, Cochrane Library, EMBASE, PsycInfo, PubMed | Yes – English | Database inception –Oct 2014 |  | Yes | - Reference lists | No |
| Barker-Collo (2013) | 4 | Medline, PubMed, PsycInfo, Web of Science | Yes – English | 1980 – NR |  | Yes | - Reference lists  - Digital Dissertations Database | Yes |
| Guillamondegui (2011) | 5 | CINAHL, EMBASE, Medline, PILOTS, PsycInfo | Yes – English | Jan 1966 – May 2010 |  | Yes | - BIOSIS Previews  - Clinical trials websites  - Google search engine  - Reference lists | Yes |
| Price (2011) | 4 | Cochrane Depression Anxiety and Neurosis Trials Register, EMBASE, Medline, PsycInfo | Unclear | NR – Aug 2009 |  | Yes | - Clinical trials websites  - Reference lists  - Pharmaceutical registries | Yes |
| Wheaton (2011) | 2 | PsycInfo, PubMed | Yes – English | Jan 1980 – April 2010 |  | Yes | - Reference lists | No |
| Rayner (2010) | 5 | CENTRAL, Cochrane Depression Anxiety and Neurosis Trials Register, EMBASE, Medline, PsycInfo | No | Cochrane Depression Anxiety and Neurosis Trials Register:  NR – Aug 2008.  All other databases: 2008-2009. |  | Yes | - Clinical trials websites  - Reference lists  - Pharmaceutical registries | Yes |
| Fann (2009) | 5 | CINAHL, ProQuest, PsycInfo, PubMed, Web of Science | Yes – English | 1980 – NR. |  | Yes | - Google Scholar  - External expert reviewers | Yes |
| Hardy (2009) | 4 | Cochrane Library, International Pharmaceutical Abstracts, Medline, PsycInfo | Yes – English | Database inception – July 2008 |  | Yes | - Reference lists | No |
| Warden (2006) | 1 | Medline | Yes – English | 1960 – Oct 2004 |  | Yes | - Reference lists  - Study authors personal files | No |
| Comper (2005) | 6 | CINAHL, EMBASE, Evidence Based Medical Reviews, HealthSTAR, Medline, PsycInfo | Yes – English | 1980 – 2003 |  | Yes | - Hand searching key journals  - Reference lists  - Web of Science  - Consultation with experts | No |
| Deb (2004) | 6 | Cochrane Library, EMBASE, Medline, PreMedline, PsycInfo, PsycLit | Yes – English | 1990 – Jan 2003  PsycInfo: 2001 – Jan 2003 |  | Yes | - Reference lists  - Hand searching a key journal | No |

Abbreviations: CENTRAL – The Cochrane Central Register of Controlled Trials; CINAHL – Cumulative Index to Nursing and Allied Health Literature; EBSCO – Elton B. Stephens Co. Information Services; EMBASE – Excerpta Medica Database; LILACS – Latin American and Caribbean Health Sciences Literature; MedLine – U.S. National Library of Medicine Database; NR – Not Reported; PILOTS – Published International Literature on Traumatic Stress Database; PsycInfo – Database produced by the American Psychological Association; PsycLit – A CD-ROM version of the American Psychological Association database; SCOPUS – Elsevier’s Abstract and Citation Database

1. A comprehensive list of search terms was accepted as provision of a search strategy. [↑](#footnote-ref-1)
2. Abstract states PubMed in place of Medline, which is listed in the ‘Search strategy’ section. [↑](#footnote-ref-2)
3. Medline stated to be the ‘main database’, unclear whether other databases were used. [↑](#footnote-ref-3)
