# Supplementary material for: Effectiveness of Pharmacotherapy for Depression after Adult Traumatic Brain Injury: an Umbrella Review
Source: Neuropsychol Rev. 2022 Jun 14;33(2):393–431. doi: 10.1007/s11065-022-09543-6 (PMC10148771; doi:10.1007/s11065-022-09543-6)
Supplement: Supplementary file 8 — Supplementary file8 (DOCX 29 KB) [file 11065_2022_9543_MOESM8_ESM.docx]

**Appendix 8**

**Table 1**

*Summary of Primary Study Findings – Drugs Not Included in Meta-Analyses^[[1]](#footnote-1)^*

|  | Drug and Drug Class | Sample Size | Injury Severity | Time Post Injury | Depression – Findings Favouring Drug | Depression – Findings Not Showing Effect of Drug | Harms | Included in which reviews? | Included in meta-analysis across drug classes? |
| --- | --- | --- | --- | --- | --- | --- | --- | --- | --- |
| Saran 1985^[[2]](#footnote-2)^ | **Phenelzine (MAOI)**  45-90mg/day; 4wks | 22 | “All these subjects claimed unconsciousness of less than 20 minutes and were awake on admission to hospital.” | “Most subjects were examined for baseline clinical and laboratory evaluations within 4 to 11 months.” |  | ***HAM-D***  *Pre-Post*  -Change in HAM-D scores at 1, 2, 3 and 4 weeks: ns | NR | n=5  [3, 7-10] | Yes; n=2  [9, 10] |
| Wroblewski 1996 | **Desipramine (TCA)**  150-300mg/day; 1.5-3mths^[[3]](#footnote-3)^ | 10 | “All participants had a minimal period of post-traumatic amnesia of 1 week and were in the range of cognitive function levels 4 to 6 on the Rancho Los Amigos Head-Injury Scale.” | M 1.5yrs (R 0.5-2.5yrs) | ***DSM-III-R***  *Pre-Post*  - 6/7 demonstrated “resolution of depression and depressed mood.”^[[4]](#footnote-4)^  ***Affect/Mood Scale***  - TG (n=6) had significantly greater improvement on the affect/mood scale than CG (n=4) (p=0.001) |  | -Two dropouts: seizures (n=1), mania (n=1_  -Two other AE: mild seizures (n=1), action tremors (n=1; resolved by lowering dose) | n=7  [2, 3, 6, 8-11] | Yes; n=2  [9, 10] |
| Newburn 1999 | **Moclobemide (MAOI)**  450-600mg/day; 6wks | 26 | NR | NR | ***HAM-D***  *Pre-Post*  -23/26 “responders^[[5]](#footnote-5)^”;  mean reduction 80.79% |  | -24 AEs reported by 14 subjects  - Five dropouts due to AEs^[[6]](#footnote-6)^ | n=7  [1-6,10] | No |
| Perino 2001 | **Citalopram (SSRI)**  10-20mg/day; 12wks & **Carbamazepine (Anti-convulsant)**  100-600mg/day; 12wks | 20 | “Injury severity was defined as a GCS of 8 or less in the first 24 hours after hospital admission.” | Participants divided into 2 groups.  A: M 4.7mths (SD 1.8)  B: M 34.6 mths (SD 6.7) | ***BPRS***  *Pre-Post*  -Baseline: M 62.3 (17.6)  -12 weeks:  M 51.7 (12.8)  p < 0.05 |  | NR | n=8  [2-6, 8, 9, 13] | Yes; n=1  [9] |
| Kanetani 2003 | **Milnacipran (SNRI)**  30-150mg/day; 6wks | 10 | “With regards to the type of TBI, four patients experienced either traumatic subarachnoid hemorrhage or cerebral contusion; three experienced concussion; two experienced acute subdural hematoma; and one experienced acute epidural hematoma. Computed tomography (CT) revealed organic brain lesions in seven patients.” | M 152.8 days (R 21 – 510) | ***HAM-D***  *Pre-Post*  - Response rate^[[7]](#footnote-7)^: 66.7%  - Remission rate^[[8]](#footnote-8)^: 44.4%  - Significant improvement after 6 weeks; p= 0.0002 |  | - One subject dropped out after one week due to nausea. | n=8  [2-4, 8-12] | Yes; n=3  [4, 9, 10] |
| Rao 2013 | **Escitalopram (SSRI)**  10-20mg/day; 12wks | 14 | NR | NR | ***MADRS^[[9]](#footnote-9)^***  *Control-Comparison*  - Baseline:  CG: M 29.5 (3.9)  TG: M 33.6 (6.9)  12 Weeks:  CG: M 11.2 (9.5)  TG: M 7 (5.7) |  | - No AEs experienced in the CG or TG. | n=1  [1] | Yes; n=1  [1] |

Abbreviations: AEs – adverse events; BPRS – Brief Psychiatric Rating Scale; CG – control group; GCS – Glasgow Coma Scale; HAM-D – Hamilton Depression Scale; M – mean; MADRS – Montgomery Asberg Depression Rating Scale; mths – months; ns – not significant SNRI – serotonin norepinephrine reuptake inhibitor; SSRI – selective serotonin reuptake inhibitor; TG – treatment group; wks – weeks

1. Kreitzer, N., et al., *The effect of antidepressants on depression after traumatic brain injury: a meta-analysis.* The Journal of head trauma rehabilitation, 2019. **34**(3): p. E47-E54.

2. Plantier, D., J. Luaute, and S. group, *Drugs for behavior disorders after traumatic brain injury: Systematic review and expert consensus leading to French recommendations for good practice.* Ann Phys Rehabil Med, 2016. **59**(1): p. 42-57.

3. Fann, J.R., T. Hart, and K.G. Schomer, *Treatment for depression after traumatic brain injury: a systematic review.* Journal of neurotrauma, 2009. **26**(12): p. 2383-2402.

4. Beedham, W., et al., *The management of depression following traumatic brain injury: A systematic review with meta-analysis.* Brain injury, 2020: p. 1-18.

5. Deb, S. and T. Crownshaw, *Review of subject The role of pharmacotherapy in the management of behaviour disorders in traumatic brain injury patients.* Brain Injury, 2004. **18**(1): p. 1-31.

6. Neurobehavioral Guidelines Working, G., et al., *Guidelines for the pharmacologic treatment of neurobehavioral sequelae of traumatic brain injury.* J Neurotrauma, 2006. **23**(10): p. 1468-501.

7. Comper, P., et al., *A systematic review of treatments for mild traumatic brain injury.* Brain injury, 2005. **19**(11): p. 863-880.

8. Wheaton, P., J.L. Mathias, and R. Vink, *Impact of pharmacological treatments on cognitive and behavioral outcome in the postacute stages of adult traumatic brain injury: a meta-analysis.* J Clin Psychopharmacol, 2011. **31**(6): p. 745-57.

9. Slowinski, A., R. Coetzer, and C. Byrne, *Pharmacotherapy effectiveness in treating depression after traumatic brain injury: a meta-analysis.* The Journal of neuropsychiatry and clinical neurosciences, 2019. **31**(3): p. 220-227.

10. Salter, K.L., et al., *Pharmacotherapy for depression posttraumatic brain injury: a meta-analysis.* Journal of head trauma rehabilitation, 2016. **31**(4): p. E21-E32.

11. Liu, Q., et al., *Pharmacological and non-pharmacological interventions of depression after traumatic brain injury: a systematic review.* European journal of pharmacology, 2019: p. 172775.

12. Barker-Collo, S., N. Starkey, and A. Theadom, *Treatment for depression following mild traumatic brain injury in adults: a meta-analysis.* Brain Injury, 2013. **27**(10): p. 1124-1133.

13. Yue, J.K., et al., *Selective Serotonin Reuptake Inhibitors for Treating Neurocognitive and Neuropsychiatric Disorders Following Traumatic Brain Injury: An Evaluation of Current Evidence.* Brain Sci, 2017. **7**(8): p. 93.

1. The primary studies in this table examine drugs that were either not included in any meta-analyses or drugs that were only included in meta-analysis that pooled findings across different drug classes. [↑](#footnote-ref-1)
2. Saran 1985 also included an intervention with amitriptyline. The amitriptyline intervention was included in numerous meta-analyses and so is not included in this table. [↑](#footnote-ref-2)
3. 6/10 participants were started on this dose. The remaining 4/10 were given a placebo for one month. As there was no significant improvement noted for any of these participants, they were all blindly crossed over to desipramine treatment. [↑](#footnote-ref-3)
4. The remaining 3 participants that were initially included in this study did not provide post treatment outcome data. [↑](#footnote-ref-4)
5. Responders defined as: those who achieved a total HAM-D score of less than 10 or a reduction of greater than 50% of the baseline score. [↑](#footnote-ref-5)
6. There were seven total dropouts. The study still reports 23/26 participants as responders. This may be because they responded before dropping out. Authors note that majority “responded” within the first 3 days. [↑](#footnote-ref-6)
7. Response rate defined as: decrease in final HAM-D score of more than 50%. [↑](#footnote-ref-7)
8. Remission rate defined as: decrease in final HAM-D score of below 7 and no longer meeting criteria for major or minor depression. [↑](#footnote-ref-8)
9. We have put the findings for Rao (2013) in the column for ‘Favours Drug’ as the findings did suggest the drug had a positive effect. However, the study provides no details on whether or not their findings were significant, or any overall conclusions about the efficacy of the intervention. [↑](#footnote-ref-9)
