# Supplementary material for: Effectiveness of Pharmacotherapy for Depression after Adult Traumatic Brain Injury: an Umbrella Review
Source: Neuropsychol Rev. 2022 Jun 14;33(2):393–431. doi: 10.1007/s11065-022-09543-6 (PMC10148771; doi:10.1007/s11065-022-09543-6)
Supplement: Supplementary file 9 — Supplementary file9 (DOCX 88 KB) [file 11065_2022_9543_MOESM9_ESM.docx]

**Appendix 9**

**Table 1**

*Risk of Bias Judgement and Rationale for the 22 Systematic Reviews Included in the Umbrella Review*

| Criteria | Judgement (Yes/No/Unclear) | | Rationale | |
| --- | --- | --- | --- | --- |
| Review: Beedham (2020) | | | | |
| Overall Score: Y=9; Y*=2; N=0; U=0 | | | | |
| Is the review question clearly and explicitly stated? | | Yes | | A clear and explicit objective was stated. Some elements of the PICO were addressed in the study objective, however, further detail was required to fully define the scope of the review.  Published protocol (PROSPERO CRD42019122600) includes two clearly articulated review questions. |
| Were the inclusion criteria appropriate for the review question? | | Yes | | The inclusion criteria seemed appropriate to the review objective. Included studies were clearly eligible when matched against the inclusion criteria. |
| Was the search strategy appropriate? | | Yes | | Articles were evaluated for inclusion by multiple authors.  The search strategy was provided as an appendix. Keywords were logical and relevant, and addressed some PICO elements. Controlled vocabulary was used. Databases were searched from inception. No language restrictions were applied. |
| Were the sources and resources used to search for studies adequate? | | Yes | | Multiple databases (≥3) were searched.  An attempt was made to search for grey literature.  Some supplementary searching was also conducted. |
| Were the criteria for appraising studies appropriate? | | Yes | | The authors used a tool based on the Cochrane Collaboration Tool that they modified to separately assess RCT studies and quasi-experimental studies. Both tools were piloted.  Further information about how the tool was modified would have been of interest. |
| Was critical appraisal conducted by two or more reviewers independently? | | Yes | | The critical appraisal was conducted independently and in duplicate by two authors. The authors discussed discrepancies, and a third author was involved when needed to reach a consensus decision. |
| Were there methods to minimize errors in data extraction? | | Yes* | | Data was only extracted by the primary reviewer, and it did not appear any independent verification was made.  There were, however, other efforts made to ensure the accuracy of data extraction including use of a customized and pre-piloted tool, use of software to extract numerical data from figures, use of experienced translators, contacting authors for incomplete data, imputation of incomplete data from comparable studies. |
| Were the methods used to combine studies appropriate? | | Yes* | | Multiple meta-analyses were conducted.  Studies were separated into prevention and treatment for the analysis and a few studies from each were ultimately excluded from the meta-analysis due to methodological issues. Sensitivity analyses were conducted to examine the impact of removing certain studies from the meta-analysis.  Where heterogeneity was detected, a random-effects model was used. Where heterogeneity was low (I^2^=0), the authors used a fixed-effects model. However, use of the fixed-effects model should only be used when it is assumed that the underlying true effect size is identical across studies. There are likely to be many clinical and methodological differences across the two studies (e.g. different medication dosage) that would necessitate use of a random-effects model. |
| Was the likelihood of publication bias assessed? | | Yes | | Publication bias was assessed using a funnel plot. The impact of publication bias may have been somewhat alleviated by the comprehensive search strategy. |
| Were recommendations for policy and/or practice supported by the reported data? | | Yes | | No strong recommendations were made. This is in line with the lack of significant findings from RCTs. Even for Methylphenidate where there were significant findings – the authors urge caution given the lack of studies. In terms of antidepressants, they note that SSRIs are likely the best, though again they do not explicitly endorse them. The authors do recommend SSRIs and Sertraline in part because of their limited side effect profile, but they do not analyse the side effect profile in the paper. |
| Were the specific directives for new research appropriate? | | Yes | | Recommendations were appropriate and focused on the need for further research into the relationship between treatment response and the severity/classification or TBI and/or depression. |
| Review: Peppel (2020) | | | | |
| Overall Score: Y=8; Y*=2; N=1; U=0 | | | | |
| Is the review question clearly and explicitly stated? | Yes | | A clear and explicit aim was stated at the end of the introduction. Some elements of the PICO were addressed in the study objective, however, further detail was required to fully define the scope of the review.  There was no published protocol for this review. | |
| Were the inclusion criteria appropriate for the review question? | Yes* | | The inclusion criteria seemed appropriate to answer the review aim.  Given that the objective of the review was to examine only ‘moderate to severe’ TBI (and this statement is included in the title), inclusion of studies with 80% mild TBI (e.g. Lee 2005), for which results for mild TBI and moderate TBI were not reported separately, is concerning. | |
| Was the search strategy appropriate? | Yes | | An information specialist was consulted to develop the search. The search strategy was provided in supplementary materials. Keywords were logical and relevant, and addressed some PICO elements. Subject headings/ indexing/controlled vocabulary were used.  Databases were searched from inception. English language restriction was used – the possible impact of this on findings was not discussed in the systematic review. | |
| Were the sources and resources used to search for studies adequate? | Yes | | Multiple databases (≥3) were searched.  An attempt was made to search for grey literature. Some supplementary searching was also conducted. | |
| Were the criteria for appraising studies appropriate? | Yes | | An appropriate critical appraisal tool was used – the Cochrane Collaboration Tool. The results of this were translated into a quality rating. The authors provided detail of the items used to assess the included studies. | |
| Was critical appraisal conducted by two or more reviewers independently? | Yes | | The critical appraisal was conducted independently and in duplicate by two authors. | |
| Were there methods to minimize errors in data extraction? | Yes | | The data extraction was conducted independently and in duplicate by two authors using a standardized tool. There was no evidence provided of training or piloting of the data extraction tool. | |
| Were the methods used to combine studies appropriate? | Yes | | Appropriate meta-analyses were conducted. Authors used standardized mean differences to combine studies using different outcome measures.  Sub-group analyses were conducted to compare each individual intervention, each intervention with each reported outcome measure, and to compare the findings of low- and high-quality studies.  Heterogeneity was explored and a ‘leave one study out’ of the analysis was conducted to test the influence of a single study on the overall effect size. | |
| Was the likelihood of publication bias assessed? | No | | The impact of publication bias may have been somewhat alleviated by the comprehensive search strategy, however, no statistical tests for publication bias were conducted. | |
| Were recommendations for policy and/or practice supported by the reported data? | Yes* | | The authors’ overall conclusions are well supported by their findings and they note the limitations in the evidence that prohibit specific recommendations being made. There were, however, a few recommendations made that weren’t directly connected to the findings of the studies reported, including that treating post- traumatic depression may improve compliance in post-acute rehabilitation and even long-term outcome. | |
| Were the specific directives for new research appropriate? | Yes | | Research recommendations were appropriate, e.g. more research, more and better defined RCTs, well-defined outcome measures, more homogenous patient population. | |
| Review: Gao (2019) | | | | |
| Overall Score: Y=8; Y*=0; N=3; U=0 | | | | |
| Is the review question clearly and explicitly stated? | | Yes | | A clear and explicit objective was stated. Some elements of the PICO were addressed in the study objective, however, further detail was required to fully define the scope of the review.  There was no published protocol for this review. |
| Were the inclusion criteria appropriate for the review question? | | No | | The eligibility criteria did not include reference to depression. |
| Was the search strategy appropriate? | | No | | No search strategy provided. |
| Were the sources and resources used to search for studies adequate? | | Yes | | Multiple databases (≥3) were searched.  No attempt was made to search for grey literature and there was no supplementary searching. |
| Were the criteria for appraising studies appropriate? | | Yes | | An appropriate critical appraisal tool was used – the Jadad Scale. The authors provided detail of the items used to assess the included studies. |
| Was critical appraisal conducted by two or more reviewers independently? | | Yes | | The critical appraisal was conducted independently and in duplicate by two authors. |
| Were there methods to minimize errors in data extraction? | | Yes | | The data extraction was conducted independently and in duplicate by two authors. There was no detail provided about the specific data extraction tool used, and no evidence provided of training or piloting of the data extraction tool. |
| Were the methods used to combine studies appropriate? | | Yes | | Appropriate meta-analyses were conducted. Heterogeneity statistics were calculated. |
| Was the likelihood of publication bias assessed? | | No | | The impact of publication bias may have been alleviated somewhat by the comprehensive search strategy, however, no statistical tests for publication bias were conducted. The authors stated this was due to the limited number (<10) of studies. |
| Were recommendations for policy and/or practice supported by the reported data? | | Yes | | The conclusion that sertraline “has no obvious benefit” to depression after TBI was in keeping with the data. |
| Were the specific directives for new research appropriate? | | Yes | | Directions for future research were appropriate (e.g. large sample sizes, standardized pharmacotherapy doses and regimes to allow for better comparison across studies). |
| Review: Kreitzer (2019) | | | | |
| Overall Score: Y=8; Y*=0; N=2; U=1 | | | | |
| Is the review question clearly and explicitly stated? | | Yes | | A clear and explicit aim was stated. Some elements of the PICO were addressed in the study aim, however, further detail was required to fully define the scope of the review.  There was no published protocol for this review. |
| Were the inclusion criteria appropriate for the review question? | | Unclear | | The inclusion criteria seemed appropriate to answer the review aim. However, the statements made with respect to exclusions for ‘prior anti-depressant use’ in the legend of Figure 1 were unclear (see below). |
| Was the search strategy appropriate? | | Yes | | The search strategy was provided in the Methods. Keywords were logical and relevant – albeit limited, and addressed some PICO elements. There was no evidence that subject headings/controlled vocabulary were used.  Unclear if date restrictions were used. English language restriction was used – the possible impact of this on findings was not stated in the systematic review. |
| Were the sources and resources used to search for studies adequate? | | No | | Only two databases were searched.  An attempt was made to search for grey literature by examining clinical trial websites.  Supplementary searching was conducted. A secondary search was undertaken 8 months after the first. |
| Were the criteria for appraising studies appropriate? | | Yes | | An appropriate critical appraisal tool was used – the Cochrane Collaboration Tool.  The authors listed the items used to assess the included studies. |
| Was critical appraisal conducted by two or more reviewers independently? | | Yes | | The critical appraisal was conducted independently and in duplicate by two authors. The authors discussed discrepancies between critical appraisals, and a third author was involved when needed to reach a consensus decision. |
| Were there methods to minimize errors in data extraction? | | Yes | | The data extraction was conducted independently and in duplicate by two authors using a standardized tool. There was no evidence provided of training or piloting of the data extraction tool. All extracted data was checked by a third-party research assistant. |
| Were the methods used to combine studies appropriate? | | Yes | | Appropriate meta-analyses were conducted. Given the objective of the study was to examine the effectiveness of anti-depressants in general, it was appropriate to pool across different drug classes. Heterogeneity statistics were calculated. The authors provided some explanation for the heterogeneity and discussed how this may impact findings/ conclusions. |
| Was the likelihood of publication bias assessed? | | No | | Quote: “Publication bias was not examined because of the lack of power for adequate asymmetry assessment.” |
| Were recommendations for policy and/or practice supported by the reported data? | | Yes | | No clear recommendations were made. This is in line with the non-significant findings in the treatment vs placebo meta-analysis. |
| Were the specific directives for new research appropriate? | | Yes | | Research recommendations were appropriate, e.g. better reporting of underlying statistics (demographics) and covariates, less heterogeneity in patient populations, better control and accounting for possible comorbidities, larger studies, better/alternative measures to HAM-D that better account for the uniqueness of post-TBI depression. |
| Review: Liu (2019) | | | | |
| Overall Score: Y=2; Y*=1; N=8; U=0 | | | | |
| Is the review question clearly and explicitly stated? | | No | | A purpose for the review was broadly provided in the introduction, however, it is not explicit enough to clearly define the scope of the review.  There was no published protocol for this review. |
| Were the inclusion criteria appropriate for the review question? | | No | | No inclusion criteria are provided. |
| Was the search strategy appropriate? | | No | | No search strategy provided. |
| Were the sources and resources used to search for studies adequate? | | No | | The sources and resources used to search for studies was not provided. |
| Were the criteria for appraising studies appropriate? | | No | | A critical appraisal was not conducted. |
| Was critical appraisal conducted by two or more reviewers independently? | | No | | A critical appraisal was not conducted. |
| Were there methods to minimize errors in data extraction? | | No | | Details of data extraction are not provided. |
| Were the methods used to combine studies appropriate? | | Yes* | | A narrative synthesis seems an appropriate approach to discussing the disparate primary studies. The synthesized findings mostly represented the data extracted from the primary studies adequately. However, there were some notable discrepancies between the data extracted into evidence tables and how the study was summarized in their narrative synthesis. |
| Was the likelihood of publication bias assessed? | | No | | No statistical tests for publication bias were conducted, and the search strategy was not limited. |
| Were recommendations for policy and/or practice supported by the reported data? | | Yes | | Limited but appropriate recommendations were made for each drug class. |
| Were the specific directives for new research appropriate? | | Yes | | The authors make appropriate and insightful suggestions including studies with larger sample sizes, taking better account of the pathology of post-TBI depression, and more personalized and flexible treatment of post-TBI depression. |
| Review: Reyes (2019) | | | | |
| Overall Score: Y=9; Y*=1; N=1; U=0 | | | | |
| Is the review question clearly and explicitly stated? | | Yes | | A clear and explicit aim was stated. Some elements of the PICO were addressed in the study aim, however, further detail was required to fully define the scope of the review.  There was no published protocol for this review. |
| Were the inclusion criteria appropriate for the review question? | | Yes | | The inclusion criteria seemed appropriate to answer the review aim. The authors provided extensive inclusion criteria for the participants (e.g. exclusions for comorbid psychiatric diagnosis, currently taking anti-depressants). |
| Was the search strategy appropriate? | | Yes | | The search strategy was provided in the Methods section. Keywords were logical and relevant – albeit limited, and addressed some PICO elements.  No evidence that subject headings/ indexing/controlled vocabulary were used.  Databases were searched from 1980 with a rationale provided. English language restriction was used – the possible impact of this on findings was not stated in the systematic review. |
| Were the sources and resources used to search for studies adequate? | | Yes | | Multiple databases (≥3) were searched.  An attempt was made to search for grey literature by examining clinical trial websites.  Some supplementary searching was also conducted by reviewing reference lists of included studies. |
| Were the criteria for appraising studies appropriate? | | Yes | | An appropriate critical appraisal tool was used – the Cochrane Collaboration Tool. There was an impressive level of detail provided regarding how the risk of bias was conducted and the assessment of each study. |
| Was critical appraisal conducted by two or more reviewers independently? | | Yes | | The critical appraisal was conducted independently and in duplicate by two authors. |
| Were there methods to minimize errors in data extraction? | | Yes | | The data extraction was conducted independently and in duplicate by two authors. Differences between the two sets of data extraction were reconciled by consensus, and involvement of a third author when needed. There was no detail provided about the data extraction tool and  no evidence provided of training or piloting of the data extraction tool. |
| Were the methods used to combine studies appropriate? | | Yes* | | Given the studies were qualitatively homogenous it seems appropriate to combine them in a meta-analysis. However, more discussion and emphasis should have been made of the high statistical heterogeneity. |
| Was the likelihood of publication bias assessed? | | No | | The impact of publication bias may have been somewhat alleviated by the comprehensive search strategy, however, no statistical tests for publication bias were conducted. |
| Were recommendations for policy and/or practice supported by the reported data? | | Yes | | The varied results of the included studies and the high heterogeneity between studies should have been considered in making conclusions from the review (i.e. how this may have impacted the pooled estimates reported).  However, as the authors did not use their findings to caution against sertraline use, we conclude that their overall conclusions were largely appropriate. |
| Were the specific directives for new research appropriate? | | Yes | | Directions for future research were appropriate, e.g. more studies are needed, with uniform criteria for and measures of depression (pre- and post-treatment), and better consistency across studies with respect to things such as time since injury, severity of injury and treatment duration. |
| Review: Slowinski (2019) | | | | |
| Overall Score: Y=7; Y*=1; N=3; U=0 | | | | |
| Is the review question clearly and explicitly stated? | | Yes | | A clear and explicit aim was stated. Some elements of the PICO were addressed in the study aim; however, further detail was required to fully define the scope of the review.  There was no published protocol for this review. |
| Were the inclusion criteria appropriate for the review question? | | Yes* | | The inclusion criteria seemed appropriate to answer the review aim, and included studies were clearly eligible when matched against the inclusion criteria. We do query the decision to expand the inclusion criteria to include studies that examined depression as a secondary outcome. The aim of these studies is not to treat depression and so it is likely that included participants would not have had clinically significant depressive symptoms at the beginning of the trial. Inclusion of these studies may impact the findings. |
| Was the search strategy appropriate? | | Yes | | The search was conducted by only one author (with oversight by another) as opposed to two independent authors. The search strategy was provided in the Methods. Keywords were logical and relevant – albeit limited and addressed some PICO elements. No evidence that subject headings/ indexing/controlled vocabulary were used.  Databases were searched from 1980 - no rationale was explicitly provided for this. English language restriction was used – the possible impact of this on findings was not stated in the systematic review. |
| Were the sources and resources used to search for studies adequate? | | Yes | | Multiple databases (≥3) were searched.  An attempt was made to search for grey literature through supplementary searching in Google Scholar. |
| Were the criteria for appraising studies appropriate? | | No | | A critical appraisal was not conducted. |
| Was critical appraisal conducted by two or more reviewers independently? | | No | | A critical appraisal was not conducted. |
| Were there methods to minimize errors in data extraction? | | No | | Details of data extraction are not provided and was only conducted by one author. |
| Were the methods used to combine studies appropriate? | | Yes | | A number of meta-analyses were conducted.  The main meta-analysis combined both pre-post single group studies with controlled trials. The validity of this approach is not clear. Heterogeneity statistics were calculated. The authors provided some explanation for the heterogeneity and discussed how this may impact findings/ conclusions. |
| Was the likelihood of publication bias assessed? | | Yes | | Publication bias was assessed using a funnel plot analysis. The impact of publication bias may have been somewhat alleviated by the comprehensive search strategy. |
| Were recommendations for policy and/or practice supported by the reported data? | | Yes | | The authors were cautious in their recommendations. This is appropriate given the lack of strength in their findings. |
| Were the specific directives for new research appropriate? | | Yes | | The author’s main recommendation is more studies on the interplay between pharmacological and non-pharmacological interventions. |
| Review: Paraschakis (2017) | | | | |
| Overall Score: Y=7; Y*=0; N=4; U=0 | | | | |
| Is the review question clearly and explicitly stated? | | Yes | | A clear and explicit objective was stated. Some elements of the PICO were addressed in the study objective; however, further detail was required to fully define the scope of the review.  There was no published protocol for this review. |
| Were the inclusion criteria appropriate for the review question? | | Yes | | The inclusion criteria seemed appropriate to answer the review objective, and included studies were clearly eligible when matched against the inclusion criteria. |
| Was the search strategy appropriate? | | Yes | | The search strategy was provided in the Methods section. Keywords were logical and relevant, and addressed some PICO elements.  No evidence that subject headings/ indexing/controlled vocabulary were used.  Databases were searched from 1990 - no rationale was explicitly provided for this.  No language restrictions were imposed. |
| Were the sources and resources used to search for studies adequate? | | No | | Multiple databases (≥3) were searched.  No attempt was made to search for grey literature. Some supplementary searching was conducted by reviewing reference lists of included studies. |
| Were the criteria for appraising studies appropriate? | | Yes | | An appropriate critical appraisal tool was used – the Cochrane Collaboration Tool.  The authors provided comprehensive detail how this tool was applied to the studies included in the review. |
| Was critical appraisal conducted by two or more reviewers independently? | | Yes | | The critical appraisal was conducted independently and in duplicate by two authors, with any discrepancies between critical appraisals settled by consensus. |
| Were there methods to minimize errors in data extraction? | | No | | The authors note that all statistical analysis was done using review manager version 5.3; however, no clear detail is given about the manner in which the data for analysis was extracted. |
| Were the methods used to combine studies appropriate? | | Yes | | Appropriate meta-analyses were conducted.  Odds ratios were calculated for 3 of the 4 studies, and standard mean differences were calculated for 2 of the 4. Heterogeneity was tested for in all analyses. |
| Was the likelihood of publication bias assessed? | | No | | No statistical tests for publication bias conducted, and the search strategy was limited. The lack of assessment for publication bias was commented on in the discussion with the absence of a grey literature search noted. |
| Were recommendations for policy and/or practice supported by the reported data? | | No | | In their conclusion, the authors state that their results “should not be considered as evidenced-based treatment guidelines.” However, early in their discussion they did make some recommendations . They advocated for a “start low, go slow titration scheme” for Sertraline. Basing this on its positive – though not significant findings – and its “favorable side-effect profile” which they did not evaluate critically in this review. Beyond Sertraline, the authors also made suggestions about the other drug classes in the space (SNRIs, monoamine oxidase inhibitors, tricyclics). These suggestions seem to be based on review of the literature more than their own findings. |
| Were the specific directives for new research appropriate? | | Yes | | Appropriate directives are suggested for more RCTs with better uniformity in their design, identification of at-risk sub-groups and the possibility of multi-modal interventions (i.e. pharmacotherapy and psychotherapy). |
| Review: Yue (2017) | | | | |
| Overall Score: Y=4; Y*=1; N=6; U=0 | | | | |
| Is the review question clearly and explicitly stated? | | Yes | | A clear and explicit objective was stated. Some elements of the PICO were addressed in the study objective; however, further detail was required to fully define the scope of the review.  There was no published protocol for this review. |
| Were the inclusion criteria appropriate for the review question? | | No | | No clear inclusion criteria were provided. |
| Was the search strategy appropriate? | | Yes | | The search strategy was provided in the Methods section. Keywords were logical and relevant – albeit limited, and addressed some PICO elements. Controlled vocabulary was used.  Unclear if date restrictions were used. English language restriction was used – the possible impact of this on findings was not stated in the systematic review. |
| Were the sources and resources used to search for studies adequate? | | No | | Only two databases were searched. No attempt was made to search for grey literature and there was no supplementary searching. |
| Were the criteria for appraising studies appropriate? | | No | | A critical appraisal was not conducted. |
| Was critical appraisal conducted by two or more reviewers independently? | | No | | A critical appraisal was not conducted. |
| Were there methods to minimize errors in data extraction? | | No | | Details of data extraction are not provided. |
| Were the methods used to combine studies appropriate? | | Yes* | | A narrative synthesis was provided that covered the majority of included papers. A meta-analysis was conducted only for the subset of papers for which it was appropriate (same intervention and common endpoint). Heterogeneity statistics were not provided. |
| Was the likelihood of publication bias assessed? | | No | | There were no statistical tests for publication bias conducted, and the search strategy was not comprehensive. |
| Were recommendations for policy and/or practice supported by the reported data? | | Yes | | The authors concluded that SSRIs represent more of a “potential option” than an “effective treatment” seem appropriate given the findings of this review. |
| Were the specific directives for new research appropriate? | | Yes | | Comprehensive recommendations are given including better study design, larger sample sizes, more homogenous populations. The overall final recommendation was  “We recommend a placebo-controlled longitudinal study with an extended follow-up period at multiple time points, along with concurrent biomarker, neuroimaging and behavioural data to delineate the true pharmacological effect of SSRIs in the TBI population.” |
| Review: Maksimowski (2016) | | | | |
| Overall Score: Y=6; Y*=0; N=5; U=0 | | | | |
| Is the review question clearly and explicitly stated? | | No | | A study objective was stated. The objective was stated in the abstract and the method section. In the abstract, the outcome is referred to as ‘psychiatric symptoms’, whereas in the method the outcome is just referred to as ‘symptoms’. Many of the included studies do not examine psychiatric symptoms - but rather cognitive symptoms of functional abilities. As such, the objective of the study with respect to ‘outcomes’ is not clearly articulated.  There was no published protocol for this review. |
| Were the inclusion criteria appropriate for the review question? | | No | | The inclusion/exclusion criteria are not provided in sufficient detail to properly ascertain their level of appropriateness.  Furthermore, the objective of the review (as stated in the abstract) is to examine the outcome of psychiatric symptoms. There are, however, a number of included studies that do not examine psychiatric symptoms, rather the outcomes of these studies are cognition or functional ability. |
| Was the search strategy appropriate? | | Yes | | The search strategy was provided in the Methods. Keywords were logical and relevant – albeit limited, and addressed some PICO elements. No evidence that subject headings/ indexing/controlled vocabulary were used.  Databases were searched from inception.  English language restriction was used – the possible impact of this on findings was not stated in the systematic review. |
| Were the sources and resources used to search for studies adequate? | | Yes | | Multiple databases (≥3) were searched.  No attempt was made to search the grey literature. Some supplementary searching was also conducted by reviewing reference lists of included studies. |
| Were the criteria for appraising studies appropriate? | | Yes | | They used the criteria developed by the Centre for Evidence Based Medicine, which is appropriate for RCTs. The authors provided detail of the items used to assess the included studies. |
| Was critical appraisal conducted by two or more reviewers independently? | | No | | It was not reported whether the critical appraisal was conducted by two or more authors independently. |
| Were there methods to minimize errors in data extraction? | | No | | Details of data extraction are not provided. |
| Were the methods used to combine studies appropriate? | | Yes | | The results of the studies are presented in a narrative format. The authors state that “the data could not be pooled due to heterogeneity between studies”; however, no further explanation is provided. |
| Was the likelihood of publication bias assessed? | | No | | The impact of publication bias may have been somewhat alleviated by the comprehensive search strategy, however, no statistical tests for publication bias were conducted. The authors did acknowledge the possibility of publication bias in the discussion: “there is the pos­sibility of publication bias in that some studies, which did not show significance, were not published.” |
| Were recommendations for policy and/or practice supported by the reported data? | | Yes | | The authors appropriately conclude that there is insufficient evidence to recommend stimulants as a “standard treatment” for individuals with TBI. |
| Were the specific directives for new research appropriate? | | Yes | | The authors make appropriate recommendations, e.g. well-conducted, larger, longer-term RCTs. They also encourage further research into other non-stimulant pharmacological treatments for TBI. |
| Review: Plantier (2016) | | | | |
| Overall Score: Y=4; Y*=0; N=6; U=1 | | | | |
| Is the review question clearly and explicitly stated? | | Yes | | A broad objective for the review is provided in the introduction. Most elements of the PICO are well addressed.  There was no published protocol for this review. |
| Were the inclusion criteria appropriate for the review question? | | No | | The inclusion criteria were not clearly stated. Some are listed in the body of the text; others appear in figures detailing their search. Those detailed in the body of the text are clear and appropriate. Those listed in the figures are not clear. For example, a few studies are listed as being excluded for including ‘mild TBI’, however, other studies with sample of mild TBI have been included in the review. Studies are also excluded due to “language” and “pediatrics” with no further elaboration on what this means. |
| Was the search strategy appropriate? | | Yes | | The search was conducted by an 8-person committee (1 librarian, 6 physicians, 1 professor). The search strategy was provided in the Methods. Keywords were logical and relevant and addressed some PICO elements. Controlled vocabulary was used.  Databases were searched from 1990 - no rationale was explicitly provided for this. Language restriction was used (according to information provided in Figure 1) but it was unclear to which language studies were restricted to and the possible impact of this on findings was not stated in the systematic review. |
| Were the sources and resources used to search for studies adequate? | | No | | The article references using the MEDLINE database as the “main” database but lists no other resources in addition to it. It is unclear to what extent their search went beyond this single database. |
| Were the criteria for appraising studies appropriate? | | No | | A critical appraisal was not conducted. The French High Authority for Health (HAS) methodology was used to grade the level of evidence. This method largely separates studies based on study design and does not consider risk of bias. For example, an RCT is obviously preferable to a case series, but that does not mean it is necessarily well conducted or free of bias. |
| Was critical appraisal conducted by two or more reviewers independently? | | No | | A critical appraisal was not conducted. |
| Were there methods to minimize errors in data extraction? | | Unclear | | There is a statement that ‘… analysis of the scientific literature and elaboration of a scientific rationale [was made] by the project managers’. However, it is not explicitly stated how the data was extracted and whether multiple people participated in data extraction. Further, the data extracted in to the evidence tables is inconsistent, raising the possibility that independent data extraction using a standardized tool may not have been used. |
| Were the methods used to combine studies appropriate? | | Yes | | A narrative summary is provided and that seems appropriate given the disparate study designs included and their varying methodological quality.  As the review considered pharmacological interventions against all behavioral sequalae of TBI, not just depression, , the effects of drugs are often discussed in terms of their pooled effects across a range of issues, including depression but also apathy, agitation, anxiety and aggression. This drug specific approach, rather than a more behavior-specific approach make it difficult to discern the evidence for each behavior type. |
| Was the likelihood of publication bias assessed? | | No | | There were no statistical tests for publication bias conducted, and the search strategy was limited. |
| Were recommendations for policy and/or practice supported by the reported data? | | Yes | | The authors provide appropriate recommendations with clear caveats about the lack of evidence and reliance on expert consensus. |
| Were the specific directives for new research appropriate? | | No | | No clear directives for further research are given. |
| Review: Salter (2016) | | | | |
| Overall Score: Y=9; Y*=2; N=0; U=0 | | | | |
| Is the review question clearly and explicitly stated? | | Yes | | A clear and explicit objective was stated. Some elements of the PICO were addressed in the study objective, however, further detail was required to fully define the scope of the review.  There was no published protocol for this review. |
| Were the inclusion criteria appropriate for the review question? | | Yes | | The inclusion criteria seemed appropriate to answer the review objective, and included studies were clearly eligible when matched against the inclusion criteria. |
| Was the search strategy appropriate? | | Yes | | The search strategy was provided in the Methods section. Keywords were logical and relevant – albeit limited, and addressed some PICO elements. Controlled vocabulary was used.  Databases were searched from inception. English language restriction was used – the possible impact of this on findings was not stated in the systematic review. |
| Were the sources and resources used to search for studies adequate? | | Yes | | Multiple databases (≥3) were searched. No attempt was made to search for grey literature. Some supplementary searching was also conducted by reviewing reference lists of included studies. |
| Were the criteria for appraising studies appropriate? | | Yes* | | A revised Jadad scale was used for all independent group designs. The authors provided some detail about how this tool was used, and the modification that was made.  However, no critical appraisal was undertaken for single group studies. There are various well-validated tools that can be used for single group study designs. |
| Was critical appraisal conducted by two or more reviewers independently? | | Yes | | The critical appraisal was conducted independently and in duplicate by two authors. The authors discussed discrepancies between critical appraisals, and a third author was involved when needed to reach a consensus decision. |
| Were there methods to minimize errors in data extraction? | | Yes | | The data extraction was conducted independently and in duplicate by two trained research associates whom were noted to have experience in this method of data collection. There was no detail provided about the specific data extraction tool used and no evidence provided of training or piloting of the data extraction tool. |
| Were the methods used to combine studies appropriate? | | Yes* | | A meta-analysis was conducted with hedges’ g used to calculate effect sizes. Detail was provided regarding decision-making for including studies and which follow-up point to extract outcome data.  Heterogeneity statistics were not calculated. |
| Was the likelihood of publication bias assessed? | | Yes | | Publication bias was comprehensively addressed. |
| Were recommendations for policy and/or practice supported by the reported data? | | Yes | | The authors are right to suggest cautious optimism given that their positive results are clearly undermined by methodological issues within their review and those of the included studies. |
| Were the specific directives for new research appropriate? | | Yes | | The only major recommendation was for a placebo-controlled trial with a large sample. The other recommendation of note was for studies of combination treatment with talk-based psychotherapies. Both of these recommendations were felt to be appropriate. |
| Review: Barker-Collo (2013) | | | | |
| Overall Score: Y=7; Y*=0; N=3; U=1 | | | | |
| Is the review question clearly and explicitly stated? | | No | | The aim of the review is stated over two sentences at the end of the introduction. Some elements of the PICO were addressed in the study aim, however, further detail was required to fully define the scope of the review. There was no published protocol for this review. |
| Were the inclusion criteria appropriate for the review question? | | Yes | | The inclusion criteria seemed appropriate to answer the review aim, and included studies were clearly eligible when matched against the inclusion criteria. The authors diligently ensured all study participants had mild TBI and obtained disaggregated data from primary study authors when needed. |
| Was the search strategy appropriate? | | Yes | | The search strategy was provided in the Methods section. Keywords were logical and relevant – albeit limited, and addressed some PICO elements. No evidence that subject headings/ indexing/controlled vocabulary were used.  Databases were searched from 1980, and the authors provided a rationale for this. English language restriction was used – the possible impact of this on findings was not stated in the systematic review. |
| Were the sources and resources used to search for studies adequate? | | Yes | | Multiple databases (≥3) were searched.  An attempt was made to search for grey literature through the Digital Dissertations Database. Some supplementary searching was also conducted by reviewing reference lists of included studies. |
| Were the criteria for appraising studies appropriate? | | No | | A critical appraisal was not conducted. |
| Was critical appraisal conducted by two or more reviewers independently? | | No | | A critical appraisal was not conducted. |
| Were there methods to minimize errors in data extraction? | | Unclear | | Details of data extraction are not explicitly given. It is clear, however, from the text that the authors went to great lengths to get appropriate and comparable data from each of the study authors. |
| Were the methods used to combine studies appropriate? | | Yes | | As the aim of the study was to examine the efficacy of treatments for post mTBI depression, combining pharmacotherapy and non-pharmacotherapy interventions appears appropriate. It is arguable, however, that providing separate meta-analyses for the pharmacotherapy and non-pharmacotherapy interventions may have been more useful for clinical application.  The authors provided detailed information regarding how they performed their meta-analyses and completed additional analyses to ensure their chosen correlation coefficient of 0.5 (for correlations between the pre- and post-intervention scores) was not biasing their results. Heterogeneity statistics were calculated. The authors provided some explanation for the heterogeneity and discussed how this may impact findings/ conclusions. |
| Was the likelihood of publication bias assessed? | | Yes | | Two Egger Funnel plots were conducted, one for the full sample and one for the control-comparator sub-sample. The impact of publication bias may have been somewhat alleviated by the comprehensive search strategy. |
| Were recommendations for policy and/or practice supported by the reported data? | | Yes | | The authors make no specific recommendations with respect to treatment for depression post-TBI, and this is in line with their lack of significant findings. |
| Were the specific directives for new research appropriate? | | Yes | | The authors make appropriate recommendations, e.g. more controlled trials, uniform outcome measures, uniform durations, explicit definitions of TBI severity, uniform sample data reporting (i.e. age, time since injury, etc.), larger samples, and less heterogeneity.  The authors, emphasized that future studies be RCTs or, at a minimum, control-compared studies. They qualify this by discussing the possibly misleading results of those studies using only pre-post designs. |
| Review: Guillamondegui (2011) | | | | |
| Overall Score: Y=9; Y*=0; N=1; U=1 | | | | |
| Is the review question clearly and explicitly stated? | | Yes | | The review is broken down into the assessment of six clear and explicit “key questions” of which the final three relate to treatment of post TBI depression. The key questions are formulated around the PICO for the review. However, further detail was required to fully define the scope of the review.  There was no published protocol for this review. |
| Were the inclusion criteria appropriate for the review question? | | Yes | | Given the breadth of the study, the inclusion criteria were appropriate and comprehensively detailed. With respect to assessing pharmacological treatment of TBI, the requirement that studies must have more than 50 participants likely limited the number of eligible studies. |
| Was the search strategy appropriate? | | Yes | | All search strategies were provided in an appendices. Keywords were logical and relevant, and addressed some PICO elements. Subject headings/ indexing/controlled vocabulary were used.  Databases were searched from inception. English language restriction was used – the authors made the following comments on this “We did not have translation services available to us to review non-English papers, and our TEP agreed that the vast majority if not all of the relevant literature would be published in English. Furthermore, this review is intended to inform U.S. health care, and most research in this population is published in studies. Empirical evidence on the potential for bias created by excluding non-English studies also suggests little effect.” |
| Were the sources and resources used to search for studies adequate? | | Yes | | Multiple databases (≥3) were searched.  An attempt was made to search for grey literature using Google and clinical trial websites.  Some supplementary searching was also conducted through BIOSIS Previews and examining the reference lists of included studies. |
| Were the criteria for appraising studies appropriate? | | Unclear | | The authors appraised studies according to a self-designed instrument. The categories on which they evaluated the studies appeared appropriate and comprehensive, and their explanations for why those categories were chosen were thorough.  However, it was not clear how the appraisal ratings from the nine criteria were mapped on to the overall adjudications and furthermore what the range of possible adjudications were, i.e. unclear if studies could only be assigned ‘poor’, ‘fair’ and ‘good’. |
| Was critical appraisal conducted by two or more reviewers independently? | | Yes | | The critical appraisal was conducted independently and in duplicate by two authors. They resolved differences through discussion, review of the publications, and arrival at consensus with the team. |
| Were there methods to minimize errors in data extraction? | | Yes | | The data extraction was conducted independently and in duplicate by two authors. Differences between the two sets of data extraction were reconciled by consensus.  There was no detail provided about the specific data extraction tool used and no evidence provided of training or piloting of the data extraction tool. After initial data extraction, another member of the team reviewed the article and checked all table entries for accuracy, completeness, and consistency. |
| Were the methods used to combine studies appropriate? | | Yes | | A narrative summary was provided. This was appropriate and represented the data extracted from the primary studies adequately. |
| Was the likelihood of publication bias assessed? | | No | | The impact of publication bias was somewhat alleviated by the comprehensive search strategy, however, no statistical tests for publication bias were conducted. There was brief mention that excluding non-English studies doesn’t significantly raise the chance of publication bias. |
| Were recommendations for policy and/or practice supported by the reported data? | | Yes | | No recommendations for policy or practice were offered as the data was rightfully deemed insufficient to make such recommendations. |
| Were the specific directives for new research appropriate? | | Yes | | The authors discussion on directives for future research focuses on two key issues: methodological quality and gaps in knowledge. On both counts they offer insightful and accurate, bullet-point recommendations that are supported by the information presented elsewhere in the paper (e.g. need for operational definitions using standard approaches to diagnosing and categorizing TBI severity, the need for collaborative multi-site studies to follow up large and more representative populations). |
| Review: Price (2011) | | | | |
| Overall Score: Y=8; Y*=0; N=2; U=1 | | | | |
| Is the review question clearly and explicitly stated? | | Yes | | A clear and explicit objective was stated. Some elements of the PICO were addressed in the study objective, however, further detail was required to fully define the scope of the review.  There was no published protocol for this review. |
| Were the inclusion criteria appropriate for the review question? | | Yes | | The inclusion criteria seemed appropriate to answer the review objective, and included studies were clearly eligible when matched against the inclusion criteria. |
| Was the search strategy appropriate? | | Yes | | The search strategy was provided in the Methods section.  Keywords were logical and relevant, and addressed some PICO elements. Unclear whether subject headings/ indexing/controlled vocabulary were used.  Unclear if date and language restrictions were used. |
| Were the sources and resources used to search for studies adequate? | | Yes | | Multiple databases (≥3) were searched.  An attempt was made to search for grey literature through clinical trial websites and pharmaceutical registries.  Some supplementary searching was also conducted by reviewing reference lists of included studies. There were two updates to the search prior to publication to ensure all evidence was up-to-date. |
| Were the criteria for appraising studies appropriate? | | Yes | | Two appropriate critical appraisal tools were used – the Cochrane Collaboration Tool and the Van Tulder 11 item Quality Assessment Scale. It would have been of benefit to provide more information about the items of the Van Tulder Scale, and a study by study breakdown of the critical appraisal results. |
| Was critical appraisal conducted by two or more reviewers independently? | | Unclear | | No comment is made as to the process of appraisal. However, given that at least two individuals independently conducted both search and data extraction, it would seem likely the same rigor was applied here. |
| Were there methods to minimize errors in data extraction? | | Yes | | The data extraction was conducted independently and in duplicate by two authors using a structured format.  Differences between the two sets of data extraction were reconciled by consensus and consultation with a third reviewer. Authors were contacted in cases of non-extractable data. There was no evidence provided of training or piloting of the data extraction tool. |
| Were the methods used to combine studies appropriate? | | Yes | | The methodological conduct of the review including the statistical analyses to combine studies was very comprehensive.  However, the degree to which it is fundamentally appropriate and clinically useful to combine across neurological conditions remains unclear.  Heterogeneity statistics were calculated.  It was surprising to see the reported heterogeneity for the meta-analysis was I^2^ = 0% for both the 4-5 week analysis and the 6-8 week. Their 9-18 week analysis (which included the TBI study) and their >18 week analysis though reported I^2^ of 78% and 75% respectively. There was no discussion of this nor how the high heterogeneity may impact findings/ conclusions. |
| Was the likelihood of publication bias assessed? | | No | | Quote: “The key limitation of our review is the possibility of publication bias. Unfortunately, owing to the predominance of small trials, it did not make sense to test for publication bias using a funnel plot.” |
| Were recommendations for policy and/or practice supported by the reported data? | | Yes | | Based on their findings, the authors recommend the use of antidepressants for treating depression in individuals with neurological disorders. This is consistent with their findings; however, given that they only included one TBI study (and one epilepsy study) such a generalization may not be appropriate and this is indeed noted by the authors. |
| Were the specific directives for new research appropriate? | | No | | Though the authors make a number of remarks about the weaknesses of the included studies and how these may have impacted the results, they do not explicitly call for corrections of these in future research. |
| Review: Wheaton (2011) | | | | |
| Overall Score: Y=4; Y*=3; N=3; U=1 | | | | |
| Is the review question clearly and explicitly stated? | | Yes | | A clear and explicit objective was stated. Some elements of the PICO were addressed in the study objective, however, further detail was required to fully define the scope of the review.  There was no published protocol for this review. |
| Were the inclusion criteria appropriate for the review question? | | Yes* | | Inclusion criteria were appropriate, extensive and detailed. The authors provided extensive inclusion criteria about the participants (e.g. exclusions for pre-existing psychiatric diagnosis, previous TBI). However, it was not clear whether this level of detail was provided in the four included primary studies to allow inclusion/ exclusion of studies against these criteria.  From the published review, it was unclear how single sample repeated measures designs met inclusion criterion b (“it had treatment and control groups…”). Appendices A of the supplementary material did provide clarification “in a single sample repeated measures design, participants act as their own control”. Although pre-test data from these study designs does provide information about depression prior to any intervention, it may not be appropriate to consider this a ‘control condition’ per se – given this design cannot control for any confounding variables or control for spontaneous recovery over time. |
| Was the search strategy appropriate? | | Yes | | The search strategy was provided in the an appendices. Keywords were logical and relevant, and addressed some PICO elements.  No evidence that subject headings/ indexing/controlled vocabulary were used.  Databases were searched from 1980 - no rationale was explicitly provided for this. English language restriction was used – the possible impact of this on findings was discussed in the systematic review. |
| Were the sources and resources used to search for studies adequate? | | No | | Only two databases were searched. No attempt was made to search for grey literature. Some supplementary searching was also conducted by reviewing reference lists of included studies. |
| Were the criteria for appraising studies appropriate? | | Unclear | | It was unclear whether the methodological quality instrument was developed by the research team or if this was a validated measure. Despite this, the measure itself was very comprehensive and covered important aspects of methodological rigor and risk of bias.  Further information would have been of benefit regarding how the scores were assigned to each study. Appendix B shows that each item is awarded a maximum of 1 point, up to a total of 20. However, given the quality scores in Table B of the supplemental materials provide scores with decimal places (e.g. 6.9; 7.2), the scoring system appears incremental on a .01 scale from 0 to 1 (as opposed to a categorical assignment of either 0 or 1). It is also unclear what score range between 0 to 20 would be considered ‘poor’ or ‘good’ quality.  Finally, there is no comprehensive discussion of study quality in the narrative of the review, making it hard to get a sense of overall study quality. |
| Was critical appraisal conducted by two or more reviewers independently? | | No | | It was not reported whether the critical appraisal was conducted by two or more authors independently. |
| Were there methods to minimize errors in data extraction? | | No | | Details of data extraction are not provided. |
| Were the methods used to combine studies appropriate? | | Yes* | | The authors provide extensive detail regarding how studies were combined for the meta-analyses. Great efforts were taken to ensure combination of studies was appropriate. Heterogeneity statistics were not calculated. |
| Was the likelihood of publication bias assessed? | | Yes | | The authors provided a very clear explanation of how publication bias was assessed, and how these findings should be interpreted: “failsafe N’s (Nfs) were calculated to address the bias toward publishing studies with significant findings. This statistic indicates the number of unpublished studies with nonsignificant treatment effects that would be needed to reduce a finding to a small effect (ie. d ≤ 0.2) and, therefore, to call the finding into question.” |
| Were recommendations for policy and/or practice supported by the reported data? | | Yes* | | Broadly, the authors make appropriate recommendations.  The recommendation for methylphenidate to treat depression is questionable given this is based on one study with n=20. |
| Were the specific directives for new research appropriate? | | Yes | | The limited directives suggested for future research appear appropriate (e.g. adequately powered randomized controlled trials). |
| Review: Rayner (2010) | | | | |
| Overall Score: Y=10; Y*=1; N=0; U=0 | | | | |
| Is the review question clearly and explicitly stated? | | Yes | | A clear and explicit objective was stated. Some elements of the PICO were addressed in the study objective, however, further detail was required to fully define the scope of the review.  There was no published protocol for this review. |
| Were the inclusion criteria appropriate for the review question? | | Yes | | The inclusion criteria seemed appropriate to answer the review objective, and included studies were clearly eligible when matched against the inclusion criteria. |
| Was the search strategy appropriate? | | Yes | | Search strategies for all databases were provided in an appendix. Keywords were logical and relevant, and addressed some PICO elements. Subject headings/ indexing/controlled vocabulary were used.  Databases were searched for a finite period (provided in the review) as this review is an update to a previously published review. No language restriction was used. |
| Were the sources and resources used to search for studies adequate? | | Yes | | Multiple databases (≥3) were searched.  An attempt was made to search for grey literature through clinical trials websites.  Some supplementary searching was also conducted (e.g. clinical trial registries, medical regulatory agency websites). |
| Were the criteria for appraising studies appropriate? | | Yes | | An appropriate critical appraisal tool was used – the Cochrane Collaboration Tool. The authors provided detail of the items used to assess the included studies. |
| Was critical appraisal conducted by two or more reviewers independently? | | Yes | | The critical appraisal was conducted independently and in duplicate by two authors. The authors discussed discrepancies between critical appraisals, and a third author (and fourth author if needed) was involved when needed to reach a consensus decision. |
| Were there methods to minimize errors in data extraction? | | Yes | | The data extraction was conducted independently and in duplicate by two authors on to a specially designed form. Consultation with two other authors was conducted if needed. There was no evidence provided of training or piloting of the data extraction tool. |
| Were the methods used to combine studies appropriate? | | Yes | | Comprehensive detail was provided regarding the combination of studies in meta-analyses.  Only one study of relevance to this umbrella review was included. |
| Was the likelihood of publication bias assessed? | | Yes | | Publication bias was assessed using a funnel plot analysis. The impact of publication bias may have been somewhat alleviated by the comprehensive search strategy. |
| Were recommendations for policy and/or practice supported by the reported data? | | Yes* | | The review included studies with a treatment population covering a variety of physical illnesses. Therefore, the degree to which a reader will agree with their findings – that antidepressants are effective in the treatment of depression in physically ill people – depends on whether or not the readers agrees with their approach (i.e. to pool across medical illnesses). In particular, this statement from the discussion stands out as notable: “It is reasonable to assume that depression is similar in different physical diseases and that (with some modification) the same treatment procedures should apply.” |
| Were the specific directives for new research appropriate? | | Yes | | Recommendations were appropriate and included more studies assessing a diverse array of antidepressants so that they can better recommend individual drugs, studies assessing the role of physical illness severity, studies assessing the role of depression severity in treatment response, studies comparing antidepressants to psychological therapies, studies exploring the impact of antidepressants on physical health/QOL outcomes. |
| Review: Fann (2009) | | | | |
| Overall Score: Y=8; Y*=1; N=2; U=0 | | | | |
| Is the review question clearly and explicitly stated? | | Yes | | A clear and explicit purpose was stated. Some elements of the PICO were addressed in the study purpose, however, further detail was required to fully define the scope of the review.  There was no published protocol for this review. |
| Were the inclusion criteria appropriate for the review question? | | Yes* | | The inclusion criteria seemed appropriate to answer the review purpose. We do query the decision to expand the inclusion criteria to include studies that examined depression as a secondary outcome. The aim of these studies is not to treat depression and so it is likely that included participants would not have had clinically significant depressive symptoms at the beginning of the trial. Inclusion of these studies may cloud the findings. |
| Was the search strategy appropriate? | | Yes | | Search strategies for all databases and Google Scholar were provided in a table. Keywords were logical and relevant, and addressed some PICO elements. Subject headings were used.  Unclear if date restrictions were used. English language restriction was used – the possible impact of this on findings was not stated in the systematic review. |
| Were the sources and resources used to search for studies adequate? | | Yes | | Multiple databases (≥3) were searched.  An attempt was made to search for grey literature through Google Scholar.  Some supplementary searching was also conducted through contacting experts in the field. |
| Were the criteria for appraising studies appropriate? | | Yes | | An appropriate critical appraisal tool was used – the American Academy of Neurology Criteria for Classifying Therapeutic Studies. The authors provided some detail about how this tool was used. |
| Was critical appraisal conducted by two or more reviewers independently? | | No | | Only one author conducted each critical appraisal. Consultation between authors occurred as needed. |
| Were there methods to minimize errors in data extraction? | | Yes | | The data extraction was conducted independently and in duplicate by two authors. Differences between the two sets of data extraction were reconciled by consensus.  There was no detail provided about the specific data extraction tool used, and no evidence provided of training or piloting of the data extraction tool. |
| Were the methods used to combine studies appropriate? | | Yes | | The studies were combined in a comprehensive narrative synthesis, which adequately covered the information extracted from the primary studies. |
| Was the likelihood of publication bias assessed? | | No | | The impact of publication bias was alleviated by the comprehensive search strategy, however, no statistical tests for publication bias were conducted. |
| Were recommendations for policy and/or practice supported by the reported data? | | Yes | | The strength of findings and quality of research have been well considered in the clinical practice recommendations in this review. |
| Were the specific directives for new research appropriate? | | Yes | | Directions for future research were appropriate (e.g. need for large, appropriately controlled pharmacological, psychosocial, alternative and multi-modal prevention and treatment studies for depression that have statistical power to compare different modalities, need for outcome measures for depression that do not require verbal self-report to validly examine treatment efficacy among participants with severe cognitive or linguistic impairments). |
| Review: Hardy (2009) | | | | |
| Overall Score: Y=7; Y*=0; N=4; U=0 | | | | |
| Is the review question clearly and explicitly stated? | | Yes | | The objective of the study was provided over two sentences at the end of the introduction. Some elements of the PICO were addressed in the study objective, however, further detail was required to fully define the scope of the review.  There was no published protocol for this review. |
| Were the inclusion criteria appropriate for the review question? | | Yes | | The inclusion criteria seemed appropriate to answer the review objective and included studies were clearly eligible when matched against the inclusion criteria. |
| Was the search strategy appropriate? | | Yes | | The search strategy was provided in the Methods section. Keywords were logical and relevant – albeit limited, and addressed some PICO elements.  No evidence that subject headings/ indexing/controlled vocabulary were used.  Databases were searched from inception. English language restriction was used – the possible impact of this on findings was not stated in the systematic review. |
| Were the sources and resources used to search for studies adequate? | | Yes | | Multiple databases (≥3) were searched.  No attempt was made to search for grey literature. Some supplementary searching was also conducted by reviewing reference lists of included studies. |
| Were the criteria for appraising studies appropriate? | | No | | A critical appraisal was not conducted. |
| Was critical appraisal conducted by two or more reviewers independently? | | No | | A critical appraisal was not conducted. |
| Were there methods to minimize errors in data extraction? | | No | | Details of data extraction are not provided. |
| Were the methods used to combine studies appropriate? | | Yes | | A narrative synthesis is provided for the results given the disparate study designs and measurements. The synthesized findings represented the data extracted from the primary studies adequately. |
| Was the likelihood of publication bias assessed? | | No | | The impact of publication bias may have been somewhat alleviated by the comprehensive search strategy, however, no statistical tests for publication bias were conducted. |
| Were recommendations for policy and/or practice supported by the reported data? | | Yes | | With respect to Methylphenidate use in general, the recommendations seem appropriate. No recommendations were made with respect to TBI specifically. |
| Were the specific directives for new research appropriate? | | Yes | | Broad but appropriate recommendations are made including better designed research to determine effectiveness, tolerability, optimal dosage and duration. |
| Review: Warden (2006) | | | | |
| Overall Score: Y=6; Y*=0; N=4; U=1 | | | | |
| Is the review question clearly and explicitly stated? | | No | | A clear and explicit objective was not stated within the review.  There was no published protocol for this review. |
| Were the inclusion criteria appropriate for the review question? | | No | | The criteria used to include/exclude articles are not explicitly discussed. At various points in the review, possible criteria can be inferred, but are not clearly labelled as ‘inclusion or exclusion criteria’. |
| Was the search strategy appropriate? | | Yes | | Search strategies were provided in the Methods section.  Keywords were logical and relevant, and addressed some PICO elements. Controlled vocabulary were used.  Databases were searched from 1960 - no rationale was explicitly provided for this. English language restriction was used – the possible impact of this on findings was not stated in the systematic review. |
| Were the sources and resources used to search for studies adequate? | | No | | Only one database was searched.  No attempt was made to search for grey literature. Some supplementary searching was also conducted by reviewing reference lists of included studies and study authors reviews of their own personal files. |
| Were the criteria for appraising studies appropriate? | | Yes | | The scoring method was adapted from the Brain Trauma Foundation’s Guidelines for the Management of Severe Head Injury. The authors provided some detail about how this tool was used. |
| Was critical appraisal conducted by two or more reviewers independently? | | Yes | | The critical appraisal was conducted independently and in duplicate by two authors. The authors discussed discrepancies between critical appraisals, and a third author was involved when needed to reach a consensus decision. |
| Were there methods to minimize errors in data extraction? | | Yes | | The data extraction was conducted independently and in duplicate by two authors. There was no detail provided about the specific data extraction tool used and no  evidence provided of training or piloting of the data extraction tool. |
| Were the methods used to combine studies appropriate? | | Yes | | A narrative review was an appropriate means of discussing the included studies. The synthesized findings represented the data extracted from the primary studies adequately. |
| Was the likelihood of publication bias assessed? | | No | | There was no statistical tests for publication bias conducted, and the search strategy was limited. |
| Were recommendations for policy and/or practice supported by the reported data? | | Unclear | | Based on the evidence reviewed, the authors recommend three different drugs as “options” for the treatment of TBI depression. They conclude there is insufficient evidence to recommend a “standard” or a “guideline”.  However, it is unclear what it means to recommend a drug as an “option”, and how this should be interpreted by prescribers. |
| Were the specific directives for new research appropriate? | | Yes | | The authors offer an extensive discussion of future research, including seven key questions for future research to examine. They also make appropriate directives including better designed studies, standardized diagnostic criteria, less diversity in TBI severity among treatment populations, standardized use of validated rating scales, reasonable time frame (e.g. 2 years) for emergence of symptoms post-TBI, control for cofounders – comorbidities, substance abuse, epilepsy. |
| Review: Comper (2005) | | | | |
| Overall Score: Y=9; Y*=0; N=1; U=1 | | | | |
| Is the review question clearly and explicitly stated? | | Yes | | A clear and explicit purpose was stated. Some elements of the PICO were addressed in the study purpose, however, further detail was required to fully define the scope of the review.  There was no published protocol for this review. |
| Were the inclusion criteria appropriate for the review question? | | Yes | | Five key eligibility questions were posed for the inclusion criteria. These were appropriate to address the review purpose. |
| Was the search strategy appropriate? | | Yes | | The electronic search was conducted by three authors and overseen by a librarian. The search strategy was provided in the Methods section. Keywords were logical and relevant – albeit limited and addressed some PICO elements. Controlled vocabulary was used.  Databases were searched from 1980 - no rationale was explicitly provided for this, beyond an aim to “obtain current studies”.  English language restriction was used – the possible impact of this on findings was addressed in the systematic review. |
| Were the sources and resources used to search for studies adequate? | | Yes | | Multiple databases (≥3) were searched.  An attempt was made to search for grey literature through consultation with experts.  Comprehensive supplementary searching was also conducted (e.g. reviewing reference lists of included studies, hand searching key journals by a team of mTBI clinicians, Web of Science - to search for other works by included authors). |
| Were the criteria for appraising studies appropriate? | | Unclear | | A tool designed as part of the Public Health, Research and Education Development programme was used. From the description provided it appears to cover the relevant areas of quality assessment; however, without a proper breakdown being given it is hard to judge.  As the authors themselves discuss, there were also some limitations/concerns with their process. Time constraints meant that they could not follow up with authors to gather non-reported information, potentially rating too harshly well conducted studies. They also weighted each of the eight sections of their quality assessment tool equally, a decision which may not have been correct. |
| Was critical appraisal conducted by two or more reviewers independently? | | Yes | | The critical appraisal was conducted independently and in duplicate by two authors. If there were discrepancies between the results, the review team would engage in discussion in order to arrive at a consensus. In order to maintain consistency, a dictionary of terms and a scoring key for each section was developed. |
| Were there methods to minimize errors in data extraction? | | Yes | | The data extraction was conducted independently and in duplicate by two authors using a standard tool designed specifically for this review. There was no evidence provided of training or piloting of the data extraction tool. |
| Were the methods used to combine studies appropriate? | | Yes | | A narrative synthesis, broken down into appropriate sub-categories is provided. In their own words, “a quantitative analysis (i.e.  a meta-analysis) was not possible given that studies differed significantly with respect to treatments and outcomes.” |
| Was the likelihood of publication bias assessed? | | No | | The impact of publication bias may have been somewhat alleviated by the comprehensive search strategy, however, no statistical tests for publication bias were conducted.  The authors do acknowledge in the discussion that the lack of grey literature and the limitation to English studies may have been potential sources of bias. |
| Were recommendations for policy and/or practice supported by the reported data? | | Yes | | The authors make no strong recommendations regarding pharmacotherapy for post TBI depression. This is in line with their lack of findings. |
| Were the specific directives for new research appropriate? | | Yes | | The authors provide appropriate research recommendations including larger, better designed studies, with more uniform definitions of key entry criteria (e.g. mild TBI).  The authors also raise two key points not mentioned in many other reviews – that researchers should not overly rely on medical settings to produce their patient populations and instead try to recruit more from the general population, and that researchers should take more steps to either control for or at least report on the potential effect of co-interventions that may be occurring concurrent with their study. |
| Review: Deb (2004) | | | | |
| Overall Score: Y=6; Y*=0; N=5; U=0 | | | | |
| Is the review question clearly and explicitly stated? | | Yes | | A clear and explicit objective was stated. Some elements of the PICO were addressed in the study objective, however, further detail was required to fully define the scope of the review.  There was no published protocol for this review. |
| Were the inclusion criteria appropriate for the review question? | | Yes | | The inclusion criteria seemed appropriate to answer the review objective, and included studies were clearly eligible when matched against the inclusion criteria. |
| Was the search strategy appropriate? | | Yes | | The search strategy was provided in the Methods section. Keywords were logical and relevant – albeit limited, and addressed some PICO elements. No evidence that subject headings/ indexing/controlled vocabulary were used.  It was unclear why the search was limited to 1990 onwards, given the supplementary search of reference lists identified a number of “relevant papers” from the 1980s that were subsequently included.  English language restriction was used – the possible impact of this on findings was not stated in the systematic review. |
| Were the sources and resources used to search for studies adequate? | | Yes | | Multiple databases (≥3) were searched.  No attempt was made to search for grey literature. Some supplementary searching was also conducted by reviewing reference lists of included studies and hand searching key journals. |
| Were the criteria for appraising studies appropriate? | | No | | A critical appraisal was not conducted. |
| Was critical appraisal conducted by two or more reviewers independently? | | No | | A critical appraisal was not conducted. |
| Were there methods to minimize errors in data extraction? | | No | | Details of data extraction are not provided. |
| Were the methods used to combine studies appropriate? | | Yes | | A narrative review seems appropriate given the heterogeneity of the included studies.  Grouping the studies by drug as opposed to the neurobehavioural symptom did make the findings somewhat difficult to interpret. Further, this structure posed an issue for studies that used combination therapies, such as Perino (2001), which used a combination of an anti-convulsant and an anti-depressant. |
| Was the likelihood of publication bias assessed? | | No | | The impact of publication bias may have been somewhat alleviated by the comprehensive search strategy, however, no statistical tests for publication bias were conducted. |
| Were recommendations for policy and/or practice supported by the reported data? | | Yes | | The authors make no strong recommendations. This is in line with their findings. To the extent that they recommend anything, it is cautionary, personalized treatment. |
| Were the specific directives for new research appropriate? | | No | | The authors identify a number of common methodological flaws across primary studies; however, they do not make any clear recommendations. |

Note: mTBI – mild traumatic brain injury; TBI – traumatic brain injury; RCT – randomised controlled trial; PICO – population, intervention, comparator, outcome

Note: Y* is used to denote when a review fulfilled the criteria for an item, however, there were small caveats that may have introduced some minor bias. The explanation for each caveat is provided in the table above.

Note: The review question or objective had to be stated in the body of the review (i.e. not just in the abstract). The review question or objective provided in the abstract often differed from review question or objective provided in the body of the review. For the sake of consistency in our evaluations, we only reviewed and commented on the review question or objective provided in the main body of the review.
